# Supplementary material for: Copper(I)-nitrene platform for chemoproteomic profiling of methionine
Source: Nat Commun. 2024 May 18;15:4243. doi: 10.1038/s41467-024-48403-0 (PMC11102537; doi:10.1038/s41467-024-48403-0)
Supplement: Supplementary file 1 — Supplementary Information [file 41467_2024_48403_MOESM1_ESM.pdf]

Supplementary Information

Copper(I)-Nitrene Platform for Chemoproteomic Profiling of Methionine

Samrat Sahu[1,#], Benjamin Emenike[1,#], Christian Michel Beusch[2,3], Pritha Bagchi[4], David Ezra Gordon[2], Monika Raj[1*]

1. Department of Chemistry, Emory University, Atlanta, GA, United States
2. Department of Pathology and Laboratory Medicine, Emory University, Atlanta, GA, United States.
3. Department of Surgical Sciences, Uppsala University, Sweden
4. Department of Biochemistry, Emory University, Atlanta, GA, United States

\# These authors contributed equally.

Email: monika.raj@emory.edu

Table of Contents

Supplementary Methods. 2

Supplementary Figures. 5

Supplementary Fig 1: HOMO-LUMO gap calculation for methionine and reactive amino acid residues. 5

Supplementary Fig. 2. Optimization of reaction condition using Ph-I=N-Ts/Cu system. 6

Supplementary Fig. 3. Chemoselectivity studies on FXV-$CONH_2$. 7

Supplementary Fig. 4. Sulfimidation of methionine under ionic mechanism using 1a. 11

Supplementary Fig. 5. Chemoselectivity studies on FXV (X= Y, K). 16

Supplementary Fig. 6. Screening of halogen scavengers for the suppression of unwanted chlorination. 18

Supplementary Fig. 7. Screening of metal salts for sulfonyl sulfimidation of methionine. 19

Supplementary Fig. 8. Energetics for acetonitrile liganded copper nitrene complex. 21

Supplementary Fig. 9. Screening of bidentate ligands. 22

Supplementary Fig. 10. Evaluation of the interaction energy of the HOMO of methionine reacting with the LUMO of copper-nitrene-acetonitrile ligand. 23

Supplementary Fig. 11. Computational evaluation of chemoselectivity of methionine and reactive amino acid residues reaction with copper-nitrene-acetonitrile complex. 24

Supplementary Fig. 12. Chemoselectivity studies on Fmoc-KQYWCREHS-$CONH_2$. 24

Supplementary Fig. 13. Experimental and computational evaluation of sulfimide stability. 29

Supplementary Fig. 14. pH and temperature stability studies of the conjugated product of Fmoc-MF-OMe 31

Supplementary Fig. 15. Evaluation of electronic effect of substituents on CuNiP reaction. 31

Supplementary Fig. 16. Labeling of Myoglobin with various probes using CuNiP. 47

Supplementary Fig. 17. DFT evaluation of electronic effects on CuNiP reaction........................... 64
Supplementary Fig. 18. Labeling of methionine in Aprotinin by CuNiP (PDB: 1OA5)................. 66
Supplementary Fig. 19. Labeling of methionine in Ubiquitin by CuNiP (PDB: 1UBQ)................. 67
Supplementary Fig. 20. Labeling of methionine in Ribonuclease-A by CuNiP (PDB: 1KF5). 69
Supplementary Fig. 21. Labeling of methionine in Lysozyme Chicken Egg-White by CuNiP (PDB: 1DPX). 74
Supplementary Fig. 22. Labeling of methionine in Human Lysozyme by CuNiP (PDB: 1REX). ... 79
Supplementary Fig. 23. Labeling of methionine in α-Chymotrpsinogen A by CuNiP (PDB: 1EX3). 83
Supplementary Fig. 24. Labeling of methionine in Carbonic Anhydrase by CuNiP (PDB: 1V9E).87
Supplementary Fig. 25. Labeling of methionine in Creatine Kinase by CuNiP (PDB: 2CRK). 92
Supplementary Fig. 26: Labeling of methionine in Bovine Serum Albumin by CuNiP (PDB: 3V03). 96
Supplementary Fig. 27. Labeling of methionine in Apo-Transferrin by CuNiP (PDB: 3V8X). 98
Supplementary Fig. 28. Installation of payloads in proteins and peptides: 103
Supplementary Fig. 29. CD Spectra of the 1a Modified Myoglobin:.. 116
Supplementary Fig. 30. Installation of payloads onto bioactive peptides.................................... 117
Supplementary Fig. 31. CuNiP modification of proteins with azide fluorophore. 125
Supplementary Fig. 32. CuNiP modification of cell lysate with azide fluorophore 126
Supplementary Fig. 33. Optimization of Cell Lysate Chemoproteomics analysis using 1a 127
Supplementary Fig. 34. Dose-dependent Cell Lysate Chemoproteomics analysis using probe 1i . 128
Supplementary Fig. 35. CuNiP mediated profiling of oxidation sensitive methionine. 132
Supplementary Fig. 36. Cell viability studies. 134
Supplementary Fig. 37. CuNiP reaction on live cells 138
Supplementary Fig. 38. Confocal microscopy imaging of CuNiP labeled T47D cells. 139
Supplementary Notes. 140
Supplementary References: 148

Supplementary Methods.

All commercial materials (Sigma-Aldrich, Fluka and Novabiochem) were used without further purification. All solvents were reagent or HPLC (Fisher) grade and degassed five times through free-pump-thaw methods. All reactions for methionine labeling were performed under N_2 atmosphere. Yields refer to chromatographically pure compounds; percent conversions were obtained by comparing HPLC peak areas of products and starting materials. TLC, HPLC and

MS were used to monitor reaction progress, and product elucidation was done using MS and NMR.

Materials. Fmoc-amino acids, Rink amide resin, Hydroxybenzotriazole (HOBT) and N,N′-diisopropylcarbodiimide (DIC) were obtained from CreoSalus (Louisville, Kentucky). Piperidine and trifluoroacetic acid (TFA) were obtained from Alfa Aesar (Ward Hill, Massachusetts). *N*,*N*-dimethylformamide (DMF), dichloromethane (DCM), methanol (MeOH) and acetonitrile (MeCN) were obtained from VWR (100 Matsonford Road Radnor, Pennsylvania). Chloramine-T, CuBr and all the other transition metal salts were purchased from Sigma-Aldrich and used as received. Adrenomedullin, ribosomal L3 peptide amide, teriparatide acetate were purchased from APExBio and used as received. Tetracosactide acetate was purchased from Alpha Diagnostic International and used as received. Aviptadil was received from ChemScene and used as received. Commercially available proteins: myoglobin, lysozyme from chicken egg white, lysozyme human, apo-transferrin human, aprotinin, α-chymotrpsinogen A, creatine kinase, bovine serum albumin (BSA), carbonic anhydrase, ribonuclease-A were obtained from Sigma-Aldrich and used without further purification. Cy5 azide were obtained from Thermo Fisher Scientific. For gel analysis: 30 % acrylamide mix, 1.5 M Tris buffer (pH 8.8), 10 % SDS, 10 % ammonium persulfate and ladders were obtained from Bio-rad.

Purification. HPLC: Purification of peptide starting materials was performed using high performance liquid chromatography (HPLC) on an Agilent 1100 series HPLC equipped with a C-18 reverse phase column with a particle size of 5 µm. All separations involved a mobile phase of 0.1 % formic acid in water (solvent A) and 0.1 % formic acid in acetonitrile (solvent B). The HPLC method used a linear gradient of 0-80% solvent B over 30 min at RT with a flow rate of 1 mL min^{-1}. The eluent was monitored by absorbance at 220 nm.

NMR. ^{1}H and ^{13}C spectra were acquired at 25 °C in DMSO-d_6, $CDCl_3$ using an Bruker 400 MHz spectrometer. All ^{1}H NMR chemical shifts (δ) were referenced relative to the residual DMSO-d_6 peak at 2.50 ppm, $CDCl_3$ peak at 7.28 ppm or internal tetramethylsilane (TMS) at 0.00 ppm. ^{13}C NMR chemical shifts were referenced to DMSO-d_6 at 39.52 ppm and $CDCl_3$ at 77.2 ppm. ^{13}C NMR spectra were acquired under proton decoupled. NMR spectral data are reported as chemical shift (multiplicity, coupling constants (J), integration). Multiplicity is reported as follows: singlet (s), doublet (d), doublet of doublets (dd), doublet of doublet of doublets (ddd), doublet of triplets (td), triplet (t) and multiplet (m). Coupling constants (J) are reported in hertz (Hz).

Analytical HPLC. Analytical HPLC chromatography (HPLC) was performed on an Agilent 1200 series HPLC equipped with a 5 µm pore size C-18 reversed-phase column. All separations involved mobile phase of 0.1 % formic acid in water (solvent A) and 0.1 % formic acid in acetonitrile (solvent B) run in linear gradients with a constant flow rate of 1 mL min^{-1}. The eluent was monitored with a detection wavelength of 220 nm. **HPLC METHOD A:** Gradient:

0-80 % B over 30 min; **HPLC METHOD B:** Gradient: 0-70 % B over 30 min; **HPLC METHOD C:** Gradient: 0-60 % B over 30 min.

LC/MS. High resolution LC-MS conditions for all purified peptides: Analyses were performed on an Agilent 1290 Infinity II LC-MS series connected to a Agilent 6545 AdvanceBio quadrupole time-of-flight mass spectrometer (Q-ToF) with electrospray ionization (ESI) in the positive mode using Agilent MassHunter Workstation (version 10.0). The raw data was analyzed using Agilent Bioconfirm (version 10.0). Unless otherwise mentioned a sample (~2 μL) was injected to Agilent PLRP-S column (1000 Å pore size, 5 μM particle size) with a gradient run of solvent B (0.1% formic acid in MeCN) of 0-90% over 4 min, flow rate - 0.4 mL min^{-1}.

MS/MS Analysis: Peptide mapping was performed on ~100 μg of the modified protein after trypsin digestion using the SMART Digest™ Trypsin Kit by Thermo Scientific. The digested sample was analyzed using LC-MS (Agilent AdvanceBio Peptide Plus 2.1 x 250 mm, 2.7 μm, pore size 100 Å) using the following method: 3-40% solvent B (0.1% formic acid in MeCN) over 15 min; then 40-90% solvent B over 15-18 min; then 90% solvent B over 18-20 min, flow rate: 0.4 ml min^{-1}. Digested peptides were analyzed using Agilent Bioconfirm (v 10.0) for MS/MS analysis.

HRMS. High resolution MS data were acquired on Thermo Exactive Plus using a heated electrospray source. The solution was infused at a rate of 10-25 μL $min\text{-}^{1}$ electrospray using 3.3 kV. The typical settings were Capillary temp 320 ^{o}C. S-lens RF level was between 30-80 with an AGC setting of 1 E^6. The maximum injection time was set to 50 ms. Spectra were taken at 140,000 resolutions at m/z 200 using Tune software and analyzed with Thermo's Freestyle software.

Fmoc Solid-Phase Peptide Synthesis (Fmoc-SPPS).[1] Peptides were synthesized using standard protocols. Peptides were synthesized manually on a 0.25 mmol scale using Rink amide resin. Resin was swollen with dichloromethane for 30 min at RT. Fmoc was deprotected using 20 % piperidine–DMF for 15 min to obtain a deprotected resin. First of the sequenced Fmoc protected amino acid (1.25 mmol, 5 equiv.) was coupled using HOBT (1.25 mmol, 5 equiv.) and DIC (1.25 mmol, 5 equiv.) in DMF for 30 min at RT. Fmoc-protected amino acids (0.75 mmol, 3 equiv.) were sequentially coupled on the resin using HOBT (1.25 mmol, 5 equiv.) and DIC (1.25 mmol, 5 equiv.) in DMF for 30 min at RT. Peptides were cleaved from the resin using 4 mL of a cocktail consisting of 92.5:2.5:2.5:2.5 trifluoroacetic acid : water : triisopropylsilane (TIS): 1,3-dimethoxybenzene for 3 h. The resin was removed by filtration and the resulting solution was concentrated. Peptides were precipitated and centrifugated with cold diethyl ether (3 x 10 mL) to obtain the crude product. Crude peptides were dissolved in MeCN:H_2O and purified by HPLC.

Method for denaturing Protein: *Preparation of 2x denaturing Buffer:* 0.1 M sodium phosphate buffer (pH 7.4 , 2 mL, final concentration 20 mM), 10% SDS solution (28.6 μL,

final concentration 2 mM, 5M NaCl solution (0.3 mL, final concentration 300 mM) were mixed together and the final volume was adjusted to 10 mL. The resulting buffer was degassed 5 times through free-pump-thaw methods. *Denaturing protocol:* Proteins were dissolved in 1:1 H_2O:denaturing buffer (final concentration of protein is 100 µM). Tris(2-carboxyethyl)phosphine hydrochloride (TCEP, 0.5 M in water) was added to it (final concentration of TCEP is 5 mM). Then it was flushed through nitrogen and incubated for 1 h at 37 ^{o}C. Then it was denatured at 95 ^{o}C for 15 min. After cooled down, the denatured proteins were filtered through Amicon Ultra 3K MWCO (4 mL volume) and washed with H_2O (6×4 mL). Finally, the cloudy solution was lyophilized to get respective denatured proteins which was used without further purification.

General cell culture technique: Cells were maintained at 37 °C and 5% CO2. T47D cells were cultured in RPMI supplemented with 10% (V/V) fetal bovine serum (FBS) and 1% (V/V) penicillin/streptomycin (100 µg/mL).

General computational details: The calculations in this study utilized the Gaussian-16 software package. The geometry of all structures was optimized using the B3LYP-D3(BJ)/[6-31G(d,p)] level of theory, which combines the B3LYP density functional with Grimme's empirical dispersion-correction (D3) and Becke-Johnson (BJ) damping-correction. The split-valence 6-31G(d,p) basis sets were employed for all atoms. Frequency analyses were conducted at the same level as the geometry optimization to characterize the minimum structures and to include enthalpy and entropy corrections. The effects of the solvent were considered by incorporating bulk solvent effects using the SMD model, with water chosen as the solvent. The reported thermodynamic data were calculated at a temperature of 298.15K and a pressure of 1atm.

Supplementary Figures.

Supplementary Fig 1: HOMO-LUMO gap calculation for methionine and reactive amino acid residues.

To evaluate the intrinsic reactivity of methionine and reactive amino acid residues, we carried out geometry optimization and natural bond order (NBO) analysis of methionine and other reactive amino acid residues using density functional theory method B3LYP-D3(BJ), 6-311g++(d,p) basis set, and the solvation model density (SMD) with water as solvent. Analysis of results showed that methionine possesses the 3rd lowest HOMO-LUMO gap.

AMINO ACID RESIDUES	HOMO	LUMO	HOMO-LUMO GAP	HOMO-LUMO GAP (eV)
TRYPTOPHAN	-0.21512	-0.03002	0.1851	5.018801
TYROSINE	-0.23373	-0.02607	0.20766	5.630493
**METHIONINE**	**-0.23235**	**-0.02146**	**0.21089**	**5.738608**
HISTIDINE	-0.23683	-0.01654	0.22029	5.972943
ARGININE	-0.24192	-0.01417	0.22775	6.175214
CYSTEINE	-0.25285	-0.01930	0.23355	6.332475
ASPARTIC ACID	-0.27428	-0.03718	0.2371	6.428729
LYSINE	-0.25056	-0.01301	0.23755	6.440931
ASPARAGINE	-0.26049	-0.01819	0.2423	6.569722
SERINE	-0.26360	-0.01655	0.24705	6.698514
N-TERMINUS (GLY-OMe)	-0.26313	-0.01306	0.25007	6.780398
C-TERMINUS (NAc-GLY)	-0.27365	-0.02301	0.25064	6.795853

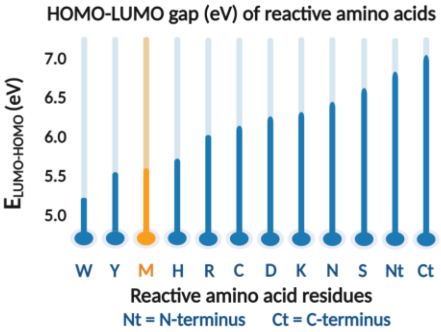

Supplementary Fig. 2. Optimization of reaction condition using Ph-I=N-Ts/Cu system.

Fmoc-MF-OMe (2 mg, 3.7 μmol, 1.0 equiv), Ph-I=N-Ts (13.8 mg, 37 μmol, 10.0 equiv), and Cu-salt (0.74 μmol, 0.2 equiv) were dissolved in either MeCN (400 μL) or MeCN:H_2O (1:1, 400 μL) and stirred at RT for 3 h. The crude reaction mixture was analyzed using HPLC (Gradient: 0-80% solvent B over 30 min, solvent B: 0.1% formic acid in MeCN) and MS.

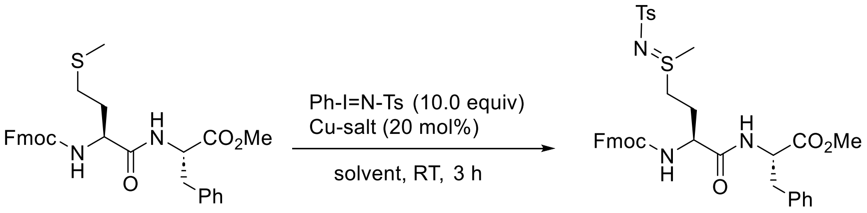

entry	Cu-salt (20 mol%)	solvent	yield (%)
1.	CuI	MeCN	95
2.	CuI	MeCN:H_2O (1:1)	75
3.	CuCl	MeCN:H_2O (1:1)	25
4.	$CuSO_4$	MeCN:H_2O (1:1)	<5
5.	$Cu(OAc)_2$	MeCN:H_2O (1:1)	18
6.	$Cu(OTf)_2$	MeCN:H_2O (1:1)	12

Fmoc-MF-OMe sulfoxide: LCMS *m/z* 549.26454 (calc. $[M+H^+]$ = 549.2054), Purity: > 95 % (HPLC analysis at 220 nm). Retention time in HPLC: 23.031 min.

Fmoc-MF-OMe sulfimide: LCMS *m/z* 702.17420 (calc. $[M+H^+]$ = 702.2302), *m/z* 724.18841 (calc. $[M+Na^+]$ = 724.2122), Purity: > 95 % (HPLC analysis at 220 nm). Retention time in HPLC: 26.916 min.

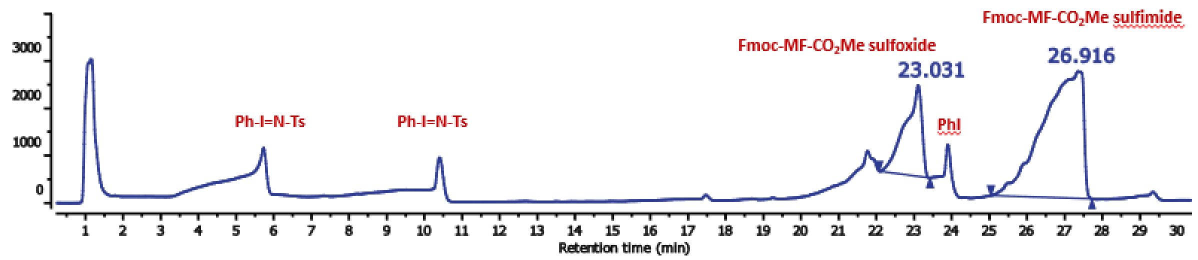

HPLC trace of reaction with Ph-I=N-Ts and CuI

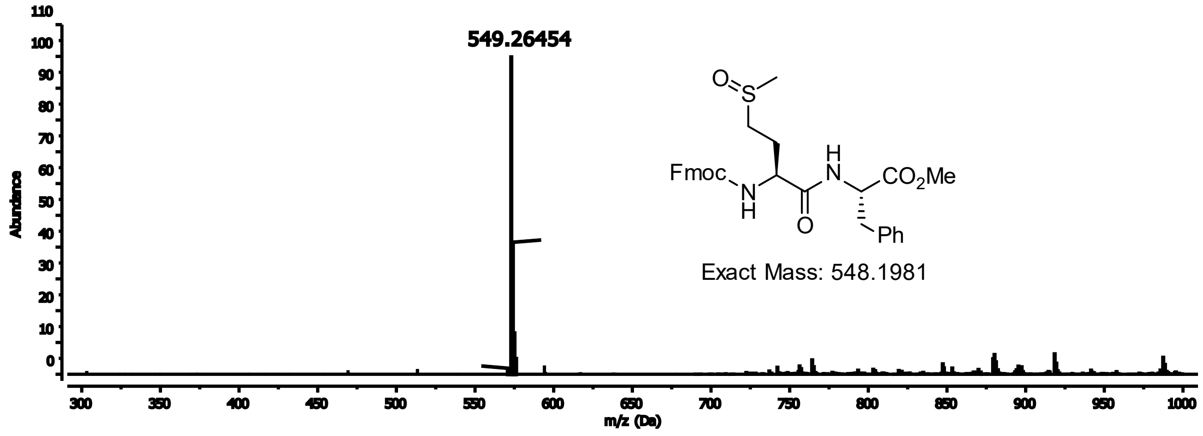

MS spectra of Fmoc-MF-OMe sulfoxide (peak 23.031)

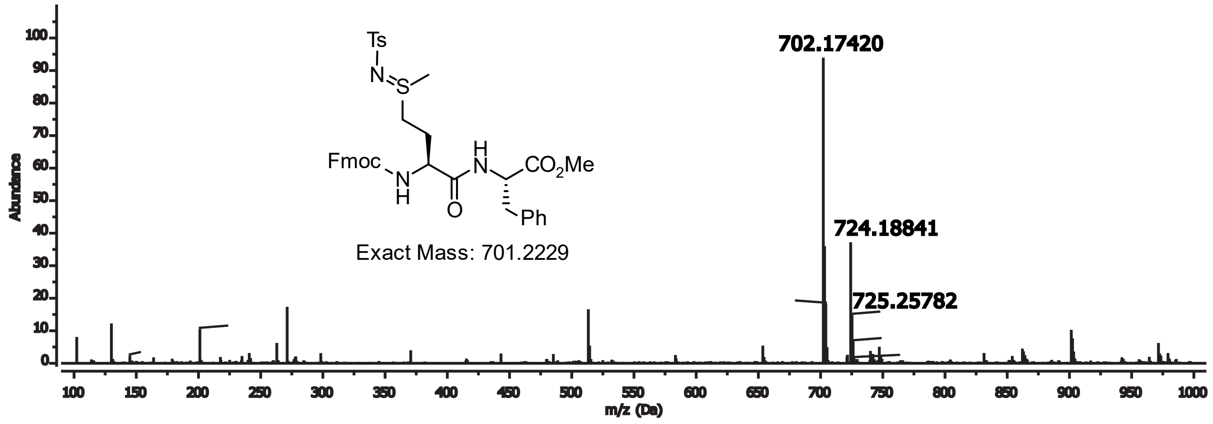

MS spectra of Fmoc-MF-OMe sulfimide (peak 26.916)

Supplementary Fig. 3. Chemoselectivity studies on FXV-$CONH_2$.

FXV-$CONH_2$ (X =Y, N, C, R, W, K, H, D, S) (3 µmol, 1.0 equiv), Ph-I=N-Ts (11 mg, 30 µmol, 10.0 equiv), and CuI (0.115 mg, 0.6 µmol, 0.2 equiv) were dissolved in MeCN:H_2O (1:1, 400 µL) and stirred at RT for 2 h. The crude reaction mixture was analyzed using HPLC (Gradient: 0-80% solvent B over 30 min, solvent B: 0.1% formic acid in MeCN) and MS.

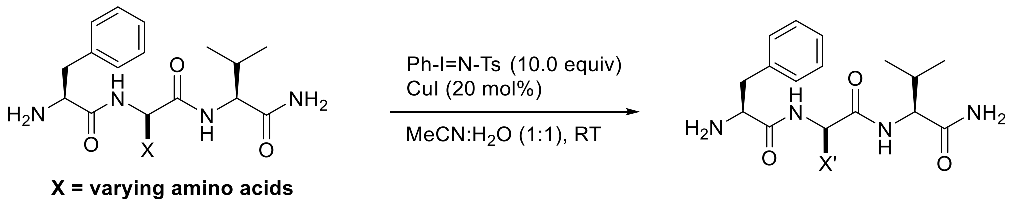

entry	tripeptide	% of recovered peptide	% of conversion (1 h)	% of conversion (2 h)
1.	FYV	<5	41 (bis-iodination)	81 (bis-iodination)
2.	FNV	>95	-	-
3.	FCV	<5	58 (cystine)	80 (cystine)
4.	FRV	>95	-	-
5.	FWV	>95	-	-
6.	FKV	>95	-	-
7.	FHV	<5	20 (mono iodination)	40 (mono iodination)
8.	FDV	>95	-	-
9.	FSV	>95	-	-

Tyrosine adducts observed with Ph-I=N-Ts:

FYV-CONH2-Iodine: LCMS *m/z* 553.11420 (calc. [M+H$^+$] = 553.1306), *m/z* 679.05978 (calc. [M+H$^+$] = 679.0273), Purity: > 95 % (HPLC analysis at 220 nm). Retention time in HPLC: 11.873 min.

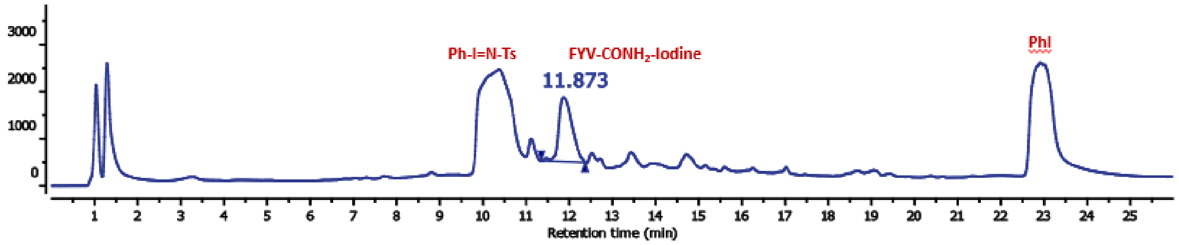

HPLC trace of tyrosine chemoselectivity studies

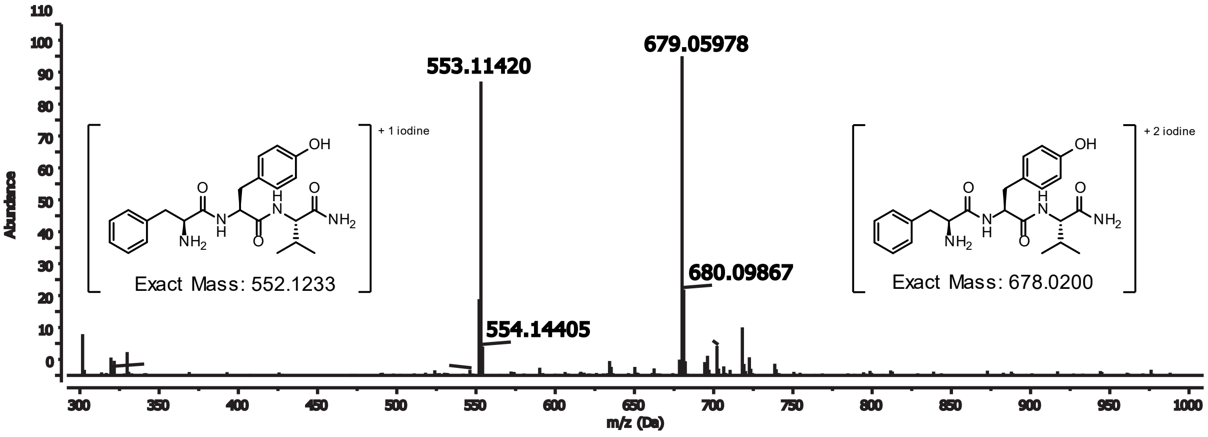

MS spectra of FYV-CONH2-Iodine (peak 11.873)

Cysteine adducts observed with Ph-I=N-Ts:

Ac-FCG-CONH2: LCMS *m/z* 367.14345 (calc. [M+H$^+$] = 367.1435), Purity: > 95 % (HPLC analysis at 220 nm). Retention time in HPLC: 6.767 min.

Ac-FCG-CONH2 disulfide: LCMS *m/z* 731.04104 (calc. [M+H$^+$] = 731.2640), *m/z* 753.12923 (calc. [M+Na$^+$] = 753.2459), *m/z* 776.10166 (calc. [M+2Na$^+$] = 776.2351), Purity: > 95 % (HPLC analysis at 220 nm). Retention time in HPLC: 12.359 min.

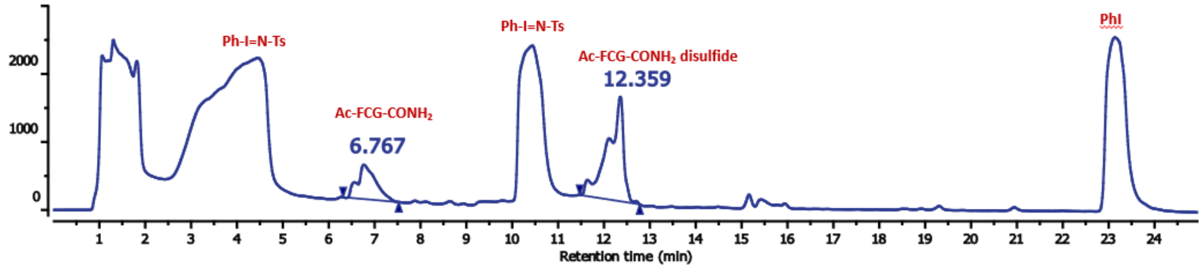

HPLC trace of cysteine chemoselectivity studies

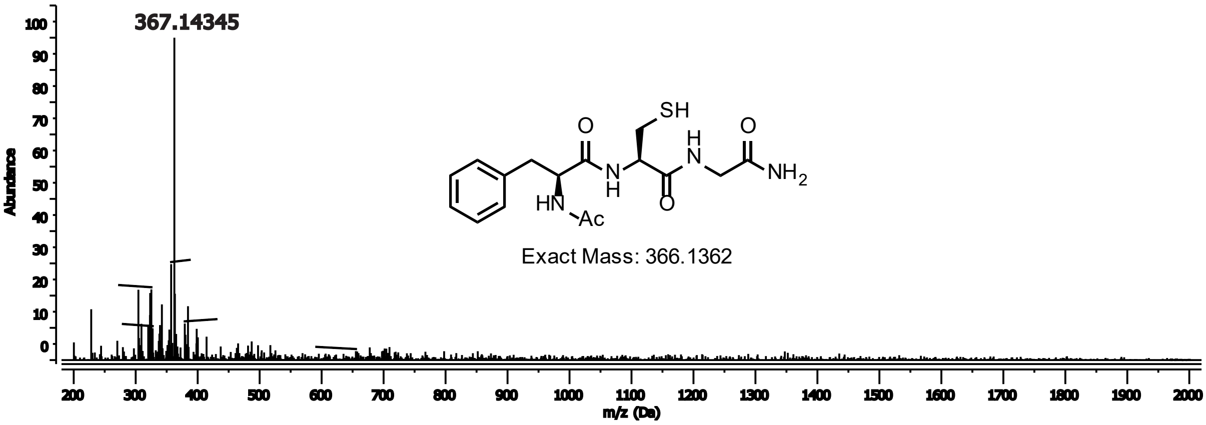

MS spectra of Ac-FCG-CONH2 (peak 6.767)

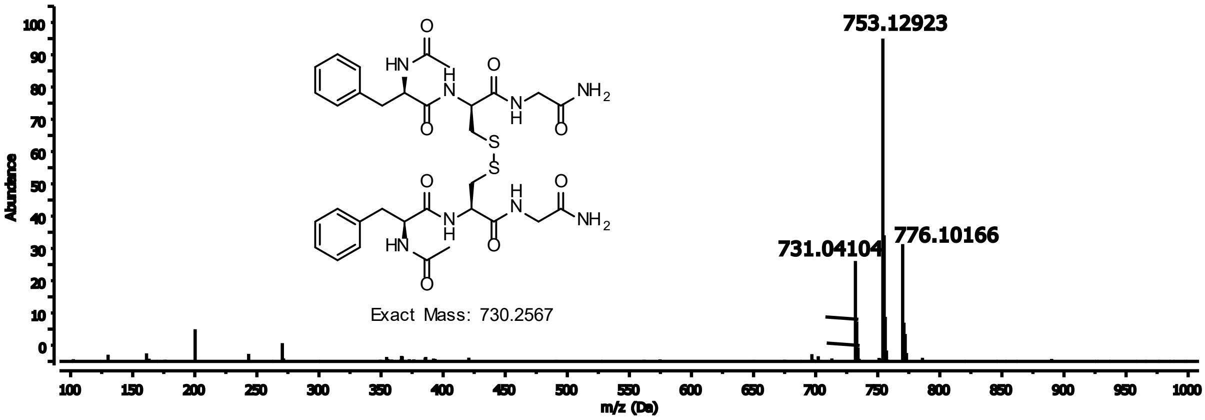

MS spectra of Ac-FCG-CONH2 disulfide (peak 12.359)

Histidine adducts observed with Ph-I=N-Ts:

FHV-CONH2 iodine: LCMS *m/z* 527.87516 (calc. [M+H$^+$] = 527.1262), Purity: > 95 % (HPLC analysis at 220 nm). Retention time in HPLC: 10.892 min.

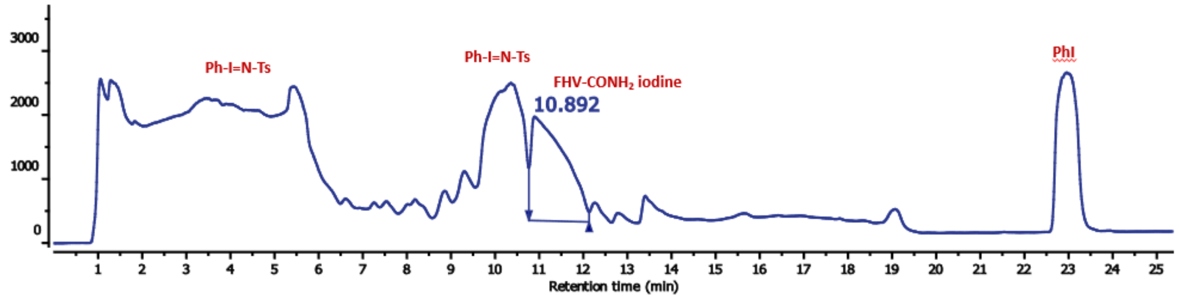

HPLC trace of histidine chemoselectivity studies

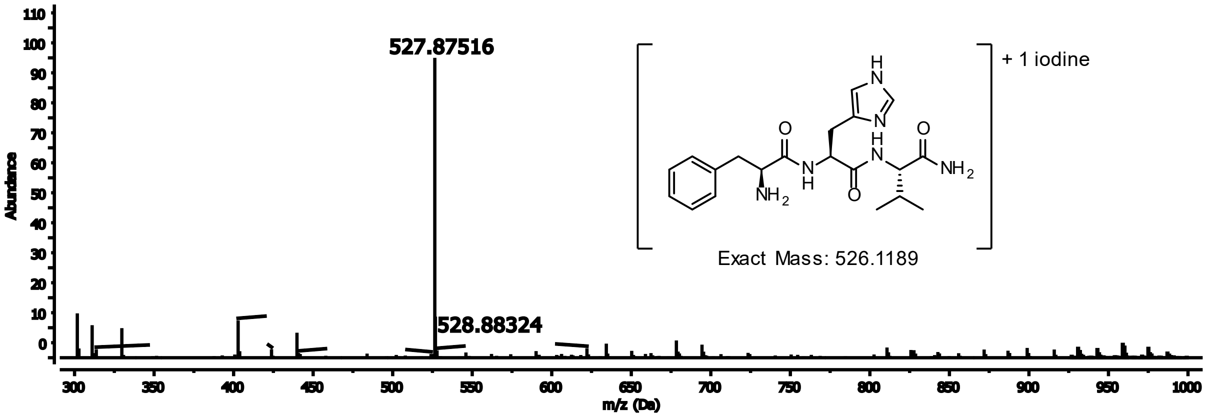

MS spectra of FHV-CONH2 (peak 10.892)

Supplementary Fig. 4. Sulfimidation of methionine under ionic mechanism using 1a.

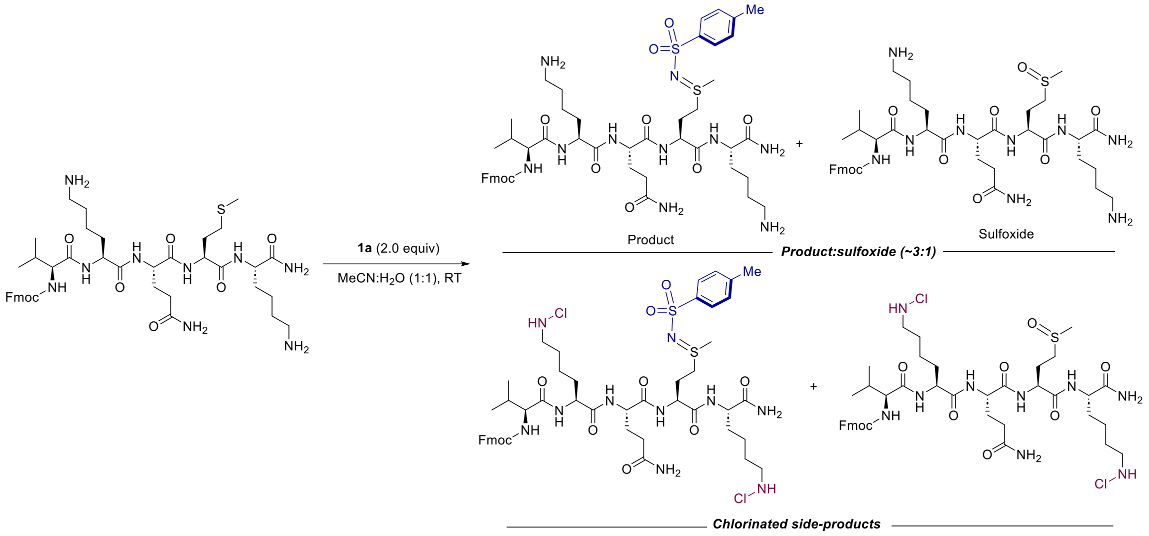

Fmoc-VKQMK-CONH$_2$ (1 mg, 1.17 µmol, 1.0 equiv) and **1a** (0.53 mg, 2.3 µmol, 2.0 equiv) were dissolved in MeCN:H$_2$O (1:1, 200 µL) and stirred at RT for 2 h. The crude reaction mixture was analyzed on HPLC (Gradient: 0-70% solvent B over 30 min, solvent B: 0.1% formic acid in MeCN) and MS. The product:sulfoxide ratio was found to be ~3:1 along with *N*-chlorination on lysine as a side product.

Fmoc-VKQM(O)K-CONH$_2$: LCMS *m/z* 870.4 (calc. [M+H$^+$] = 870.5), *m/z* 892.6 (calc. [M+Na$^+$] = 892.6), Purity: > 95 % (HPLC analysis at 220 nm). Retention time in HPLC: 11.272 min.

Fmoc-VKQM(mod)K-CONH$_2$: LCMS *m/z* 1023.5 (calc. [M+H$^+$] = 1023.5), *m/z* 1045.6 (calc. [M+Na$^+$] = 1045.6), m/z 512.4 (calc. [M+2H$^+$]$^{2+}$ = 512.4) Purity: > 95 % (HPLC analysis at 220 nm). Retention time in HPLC: 12.965 min.

Fmoc-VK(Cl)QM(O)K(Cl)-CONH$_2$: LCMS *m/z* 938.4 (calc. [M+H$^+$] = 938.4), *m/z* 960.3 (calc. [M+Na$^+$] = 960.3), Purity: > 95 % (HPLC analysis at 220 nm). Retention time in HPLC: 15.234 min.

Fmoc-VK(Cl)QM(mod)K(Cl)-CONH$_2$: LCMS *m/z* 1091.4 (calc. [M+H$^+$] = 1091.4), Purity: > 95 % (HPLC analysis at 220 nm). Retention time in HPLC: 16.605 min.

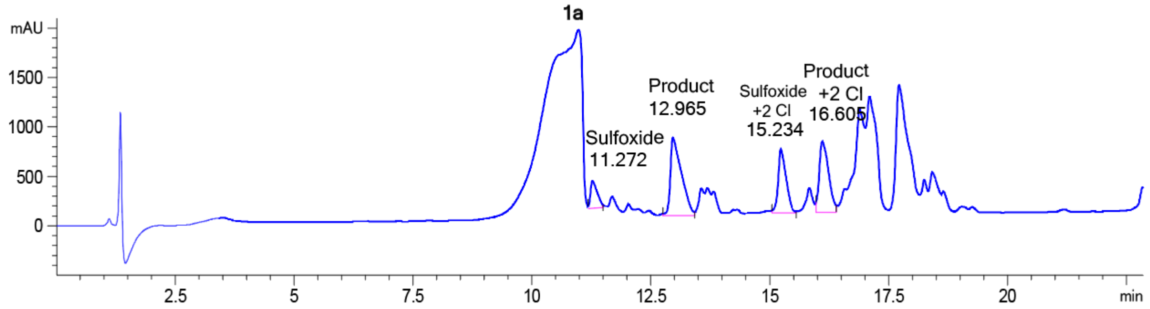

HPLC trace of the reaction mixture

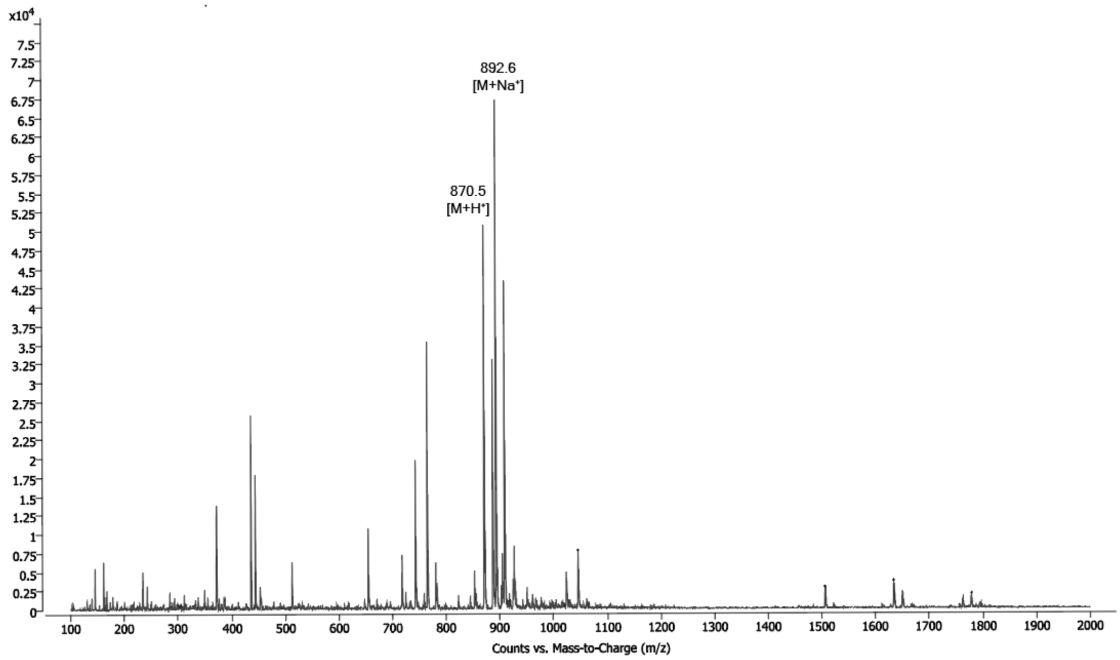

MS-Spectra of Fmoc-VKQM(O)K-$CONH_2$

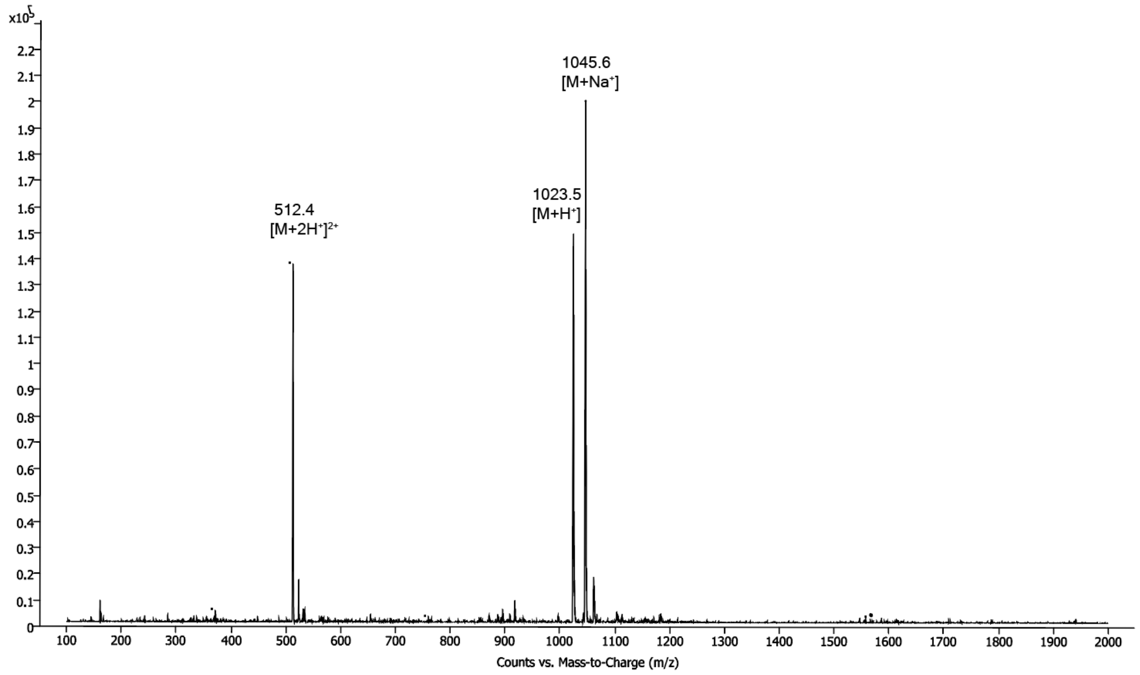

MS Spectra of Fmoc-VKQM(mod)K-$CONH_2$

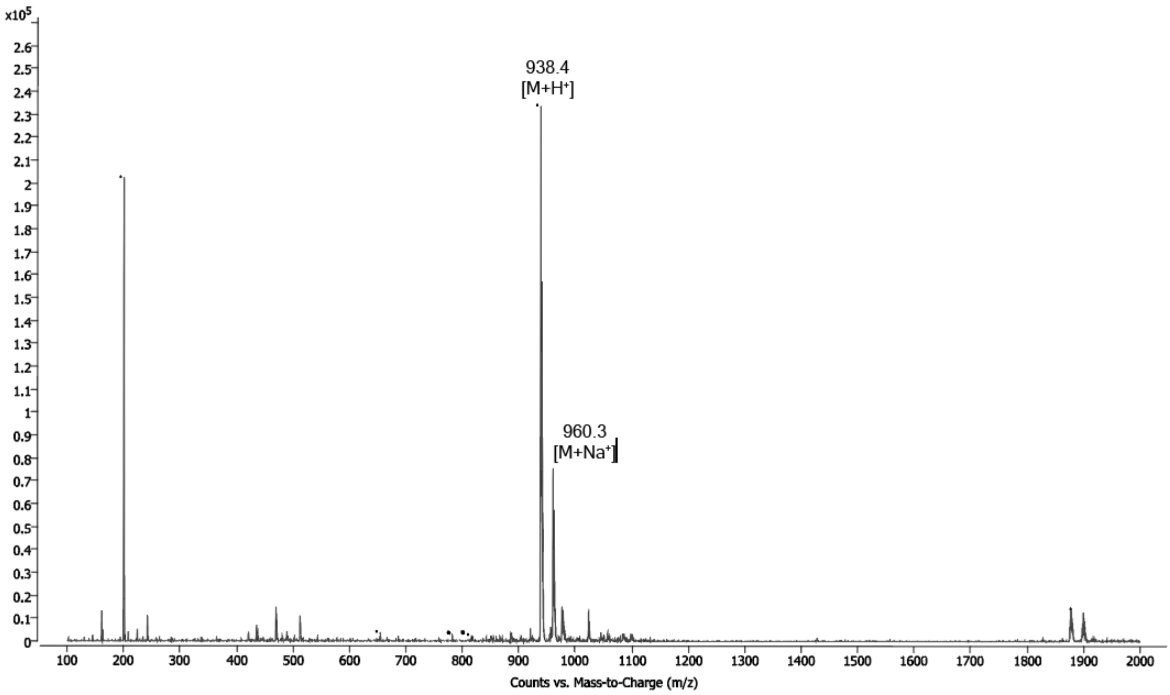

MS Spectra of Fmoc-VK(Cl)QM(O)K(Cl)-$CONH_2$

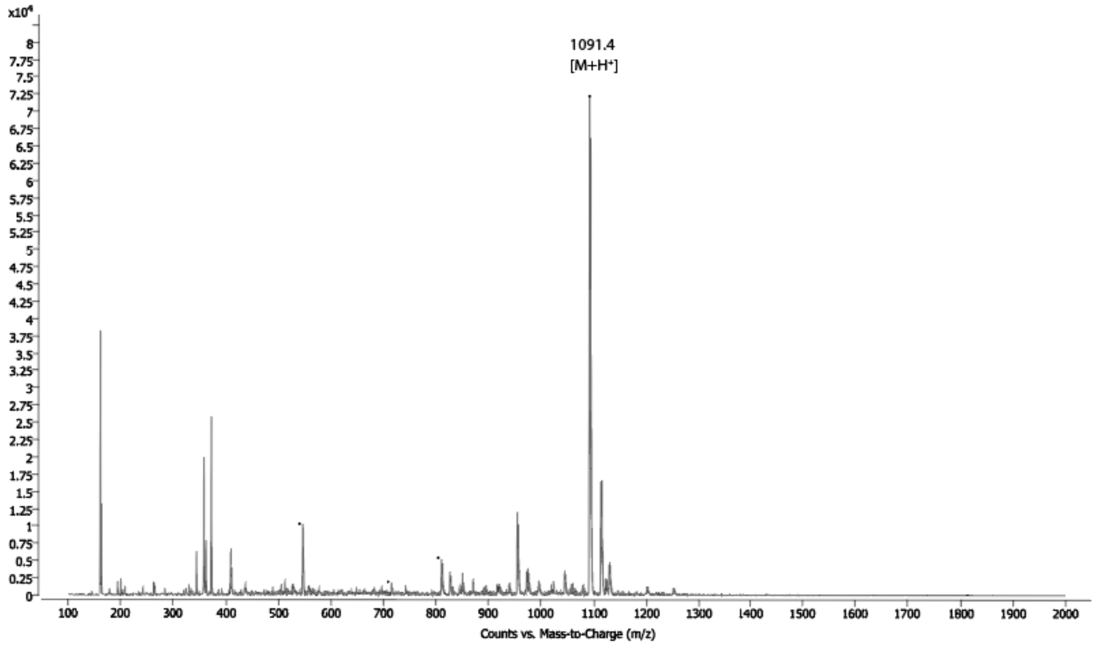

MS Spectra of Fmoc-VK(Cl)QM(mod)K(Cl)-$CONH_2$

NMR characterization of the sulfonyl sulfimide product:

Methyl (2*S*)-2-((((9*H*-fluoren-9-yl)methoxy)carbonyl)amino)-4-((*E*)-*S*-methyl-N-tosylsulfinimidoyl)butanoate

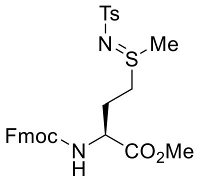

To a solution of Fmoc-Met-OMe (770 mg, 2.0 mmol) in dry MeCN (8 mL) was added chloramine-T (682 mg, 3.0 mmol, 1.5 equiv). The resulting mixture was stirred under air for 2 h. Upon completion, H_2O was added to it and extracted with ethyl acetate. The combined organic layers were dried over Na_2SO_4, concentrated, and purified on silica by using 2% MeOH in DCM to get the titled product as white solid (898 mg, 81%, 1:1 diastereomeric ratio). **^{1}H NMR** (400 MHz, $CDCl_3$) δ = 7.78 – 7.74 (m, 4H), 7.63 – 7.56 (m, 2H), 7.39 (t, *J* = 7.4 Hz, 2H), 7.32 – 7.27 (m, 2H), 7.21 (d, *J* = 8.0 Hz, 1H), 7.17 (d, *J* = 8.0 Hz, 1H), 5.82 – 5.73 (m, 1H), 4.42 – 4.34 (m, 2H), 4.22 – 4.16 (m, 1H), 3.74 (s, 3H), 3.07 – 2.95 (m, 1H), 2.93 – 2.83 (m, 1H), 2.58 (s, 3H), 2.36 (s, 3H), 2.32 – 2.22 (m, 1H), 2.12 – 1.94 (m, 1H) ppm. **^{13}C NMR** (101 MHz, $CDCl_3$, one diastereomer) δ = 171.32, 156.45, 143.85, 143.66, 142.00, 141.40, 129.45, 127.91, 127.25, 126.39, 125.18, 120.14, 67.24, 53.19, 53.09, 47.41, 47.18, 34.33, 25.68, 21.53 ppm.

^{1}H NMR of Methyl (2*S*)-2-((((9*H*-fluoren-9-yl)methoxy)carbonyl)amino)-4-((*E*)-*S*-methyl-N-tosylsulfinimidoyl)butanoate

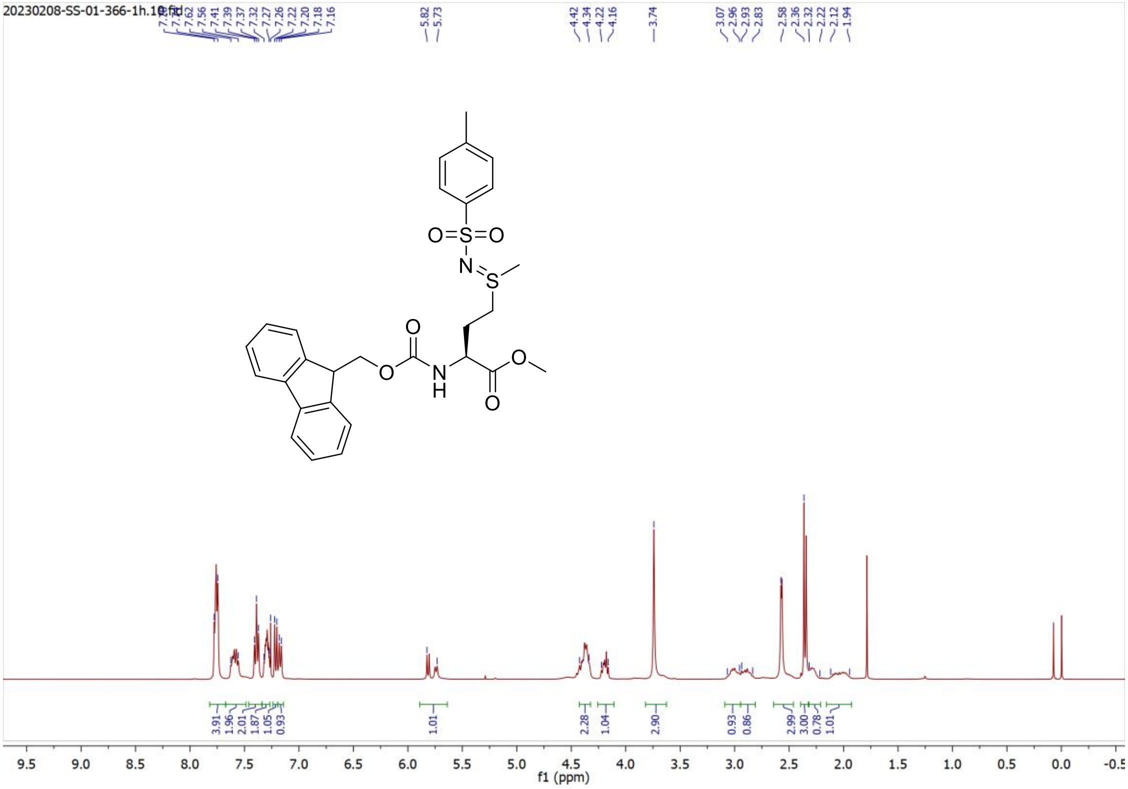

^{13}C NMR of Methyl (2*S*)-2-((((9*H*-fluoren-9-yl)methoxy)carbonyl)amino)-4-((*E*)-*S*-methyl-N-tosylsulfinimidoyl)butanoate

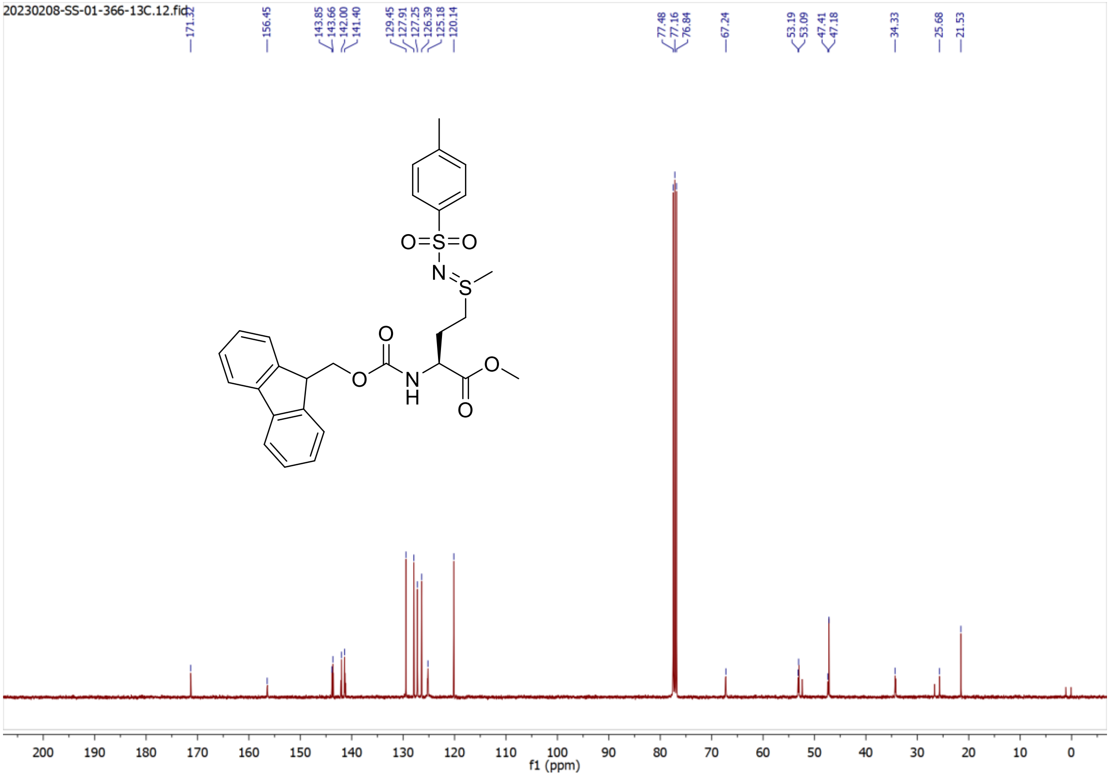

Supplementary Fig. 5. Chemoselectivity studies on FXV (X= Y, K).

F**X**V-CONH$_2$ (X = Y, K) (2.3 µmol, 1.0 equiv) and **1a** (1 mg, 4.6 µmol, 2.0 equiv) were dissolved in MeCN:H$_2$O (4:1, 400 µL) and stirred at RT for 1 h. The crude reaction mixture was analyzed on HPLC (Gradient: 0-80 % solvent B over 30 min, solvent B: 0.1% formic acid in MeCN) and MS.

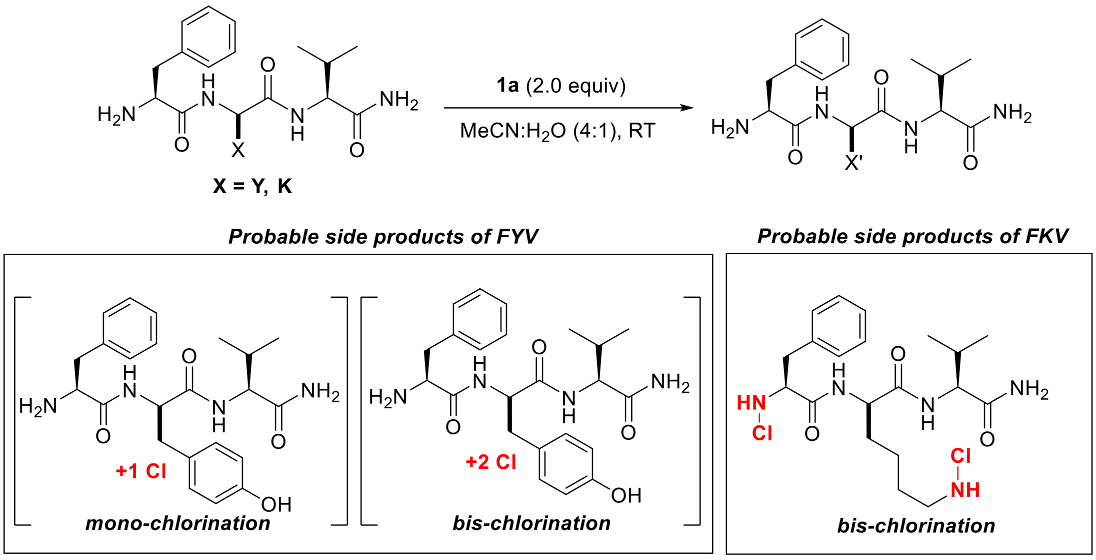

entry	tripeptide	% of recovered peptide	% of conversion (1 h)
1.	FYV	<5%	41 (+1 Cl)<br>42 (+2 Cl)
2	FKV	22%	78 (+2 Cl)

[FYV-CONH$_2$ + 1Cl]: LCMS *m/z* 461.2 (calc. [M+H$^+$] = 461.2), *m/z* 943.3 (calc. [2M+Na$^+$] = 943.4). Purity: > 95 % (HPLC analysis at 220 nm). Retention time in HPLC: 15.221 min.

[FYV-CONH$_2$ + 2Cl]: LCMS *m/z* 495.3 (calc. [M+H$^+$] = 495.3). Purity: > 95 % (HPLC analysis at 220 nm). Retention time in HPLC: 18.043 min.

[FKV-CONH$_2$ + 2Cl]: LCMS *m/z* 460.3 (calc. [M+H$^+$] = 460.3), m/z 943.5 (calc. [2M+Na$^+$] = 943.5). Purity: > 95 % (HPLC analysis at 220 nm). Retention time in HPLC: 12.341 min.

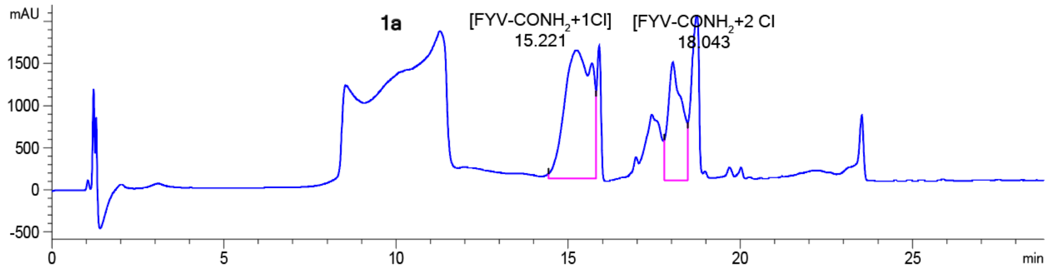

HPLC trace of the reaction mixture of FYV-CONH$_2$ with **1a**

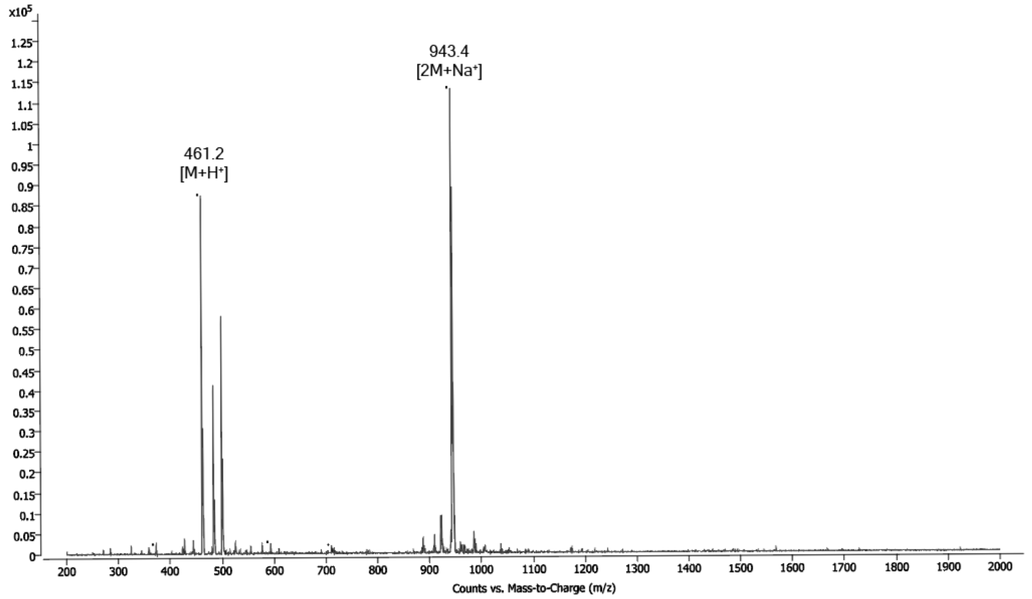

MS Spectra of [FYV-CONH$_2$ + 1Cl]

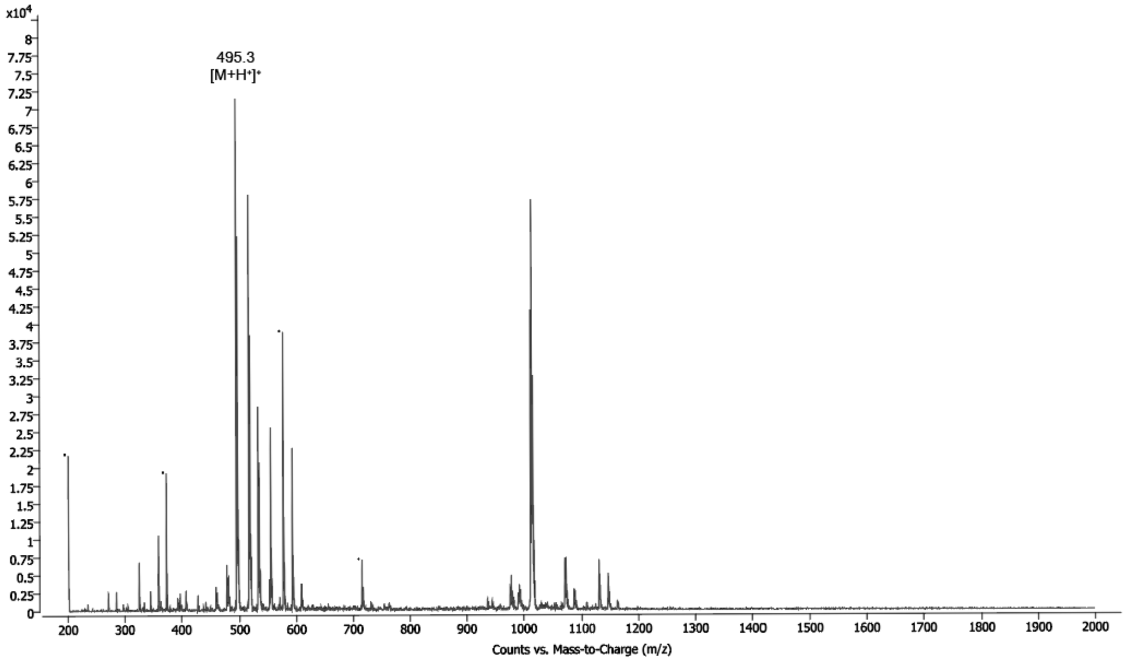

MS Spectra of [FYV-CONH2 + 2Cl]

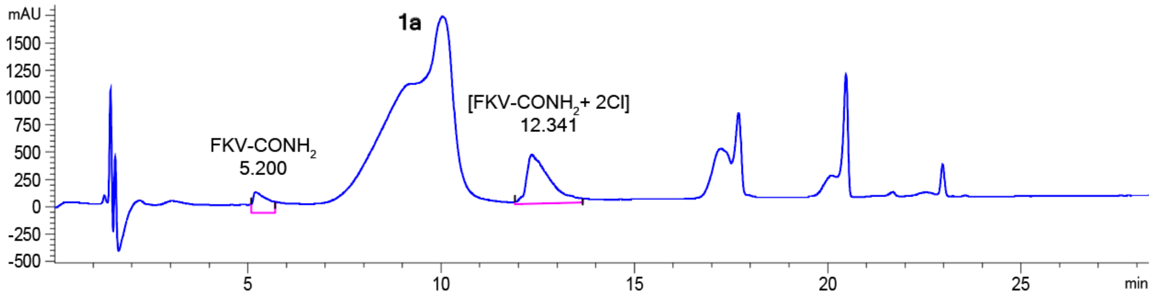

HPLC trace of the reaction mixture of FKV-CONH2 with **1a**

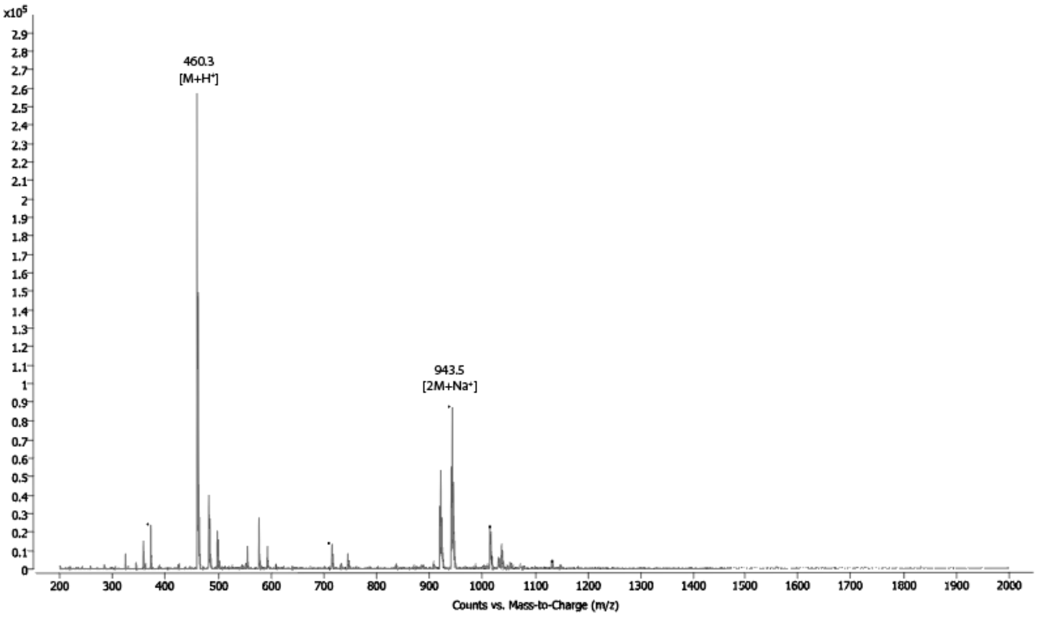

MS-Spectra of [FKV-CONH2 + 2Cl]

Supplementary Fig. 6. Screening of halogen scavengers for the suppression of unwanted chlorination.

FKV-CONH2 (2 mg, 5.0 µmol, 1.0 equiv), halogen scavenger (150 µmol, 30.0 equiv), and **1a** (2.27 mg, 10.0 µmol, 2.0 equiv) were dissolved in MeCN:H_2O (4:1, 400 µL) and stirred at RT for 1 h. The crude reaction mixture was analyzed on HPLC (Gradient: 0-70 % solvent B over 30 min, solvent B: 0.1% formic acid in MeCN) and MS.

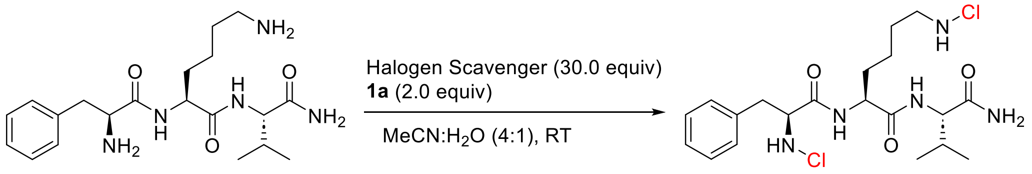

entry	Halogen Scavengers	Bis-chlorination	Recovered peptide
1.	*N*,*N*-dimethylaniline (*N*,*N*-DMA)	35%	65%
2.	1,3,5-trimethoxybenzene (1,3,5-TMB)	48%	52%
3.	mesitylene	88%	12%
4.	1,3-dimethoxybenzene (1,3-DMB)	89%	11%
5.	phenol	87%	13%
6.	indole	33%	67%
6.	pyrrole	12%	88%

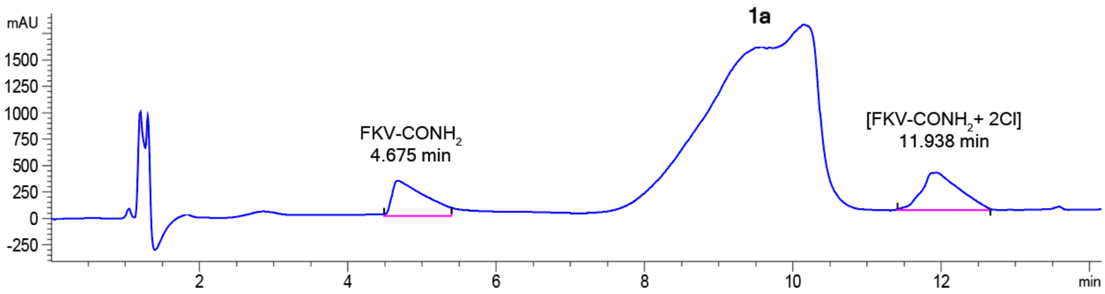

HPLC trace of the reaction mixture of FKV-CONH$_2$ and **1a** in presence of 1,3,5-trimethoxy benzene (30 equiv), showing a lower conversion to bis-chlorinated product (48%) as compared to 78% for the same reaction without the halogen scavenger.

Supplementary Fig. 7. Screening of metal salts for sulfonyl sulfimidation of methionine.

Fmoc-VKQMK-CONH$_2$ (1 mg, 1.17 µmol, 1.0 equiv), metal salts (5.8 µmol, 5.0 equiv), and **1a** (1.33 mg, 5.8 µmol, 5.0 equiv) were dissolved in MeCN:H$_2$O (1:1, 200 µL) under nitrogen atmosphere and stirred at RT for 5 h. The reaction was quenched by adding 10 µL of 0.5 M HCl and analyzed on HPLC (Gradient: 0-70% solvent B over 30 min, solvent B: 0.1% formic acid in MeCN) and MS.

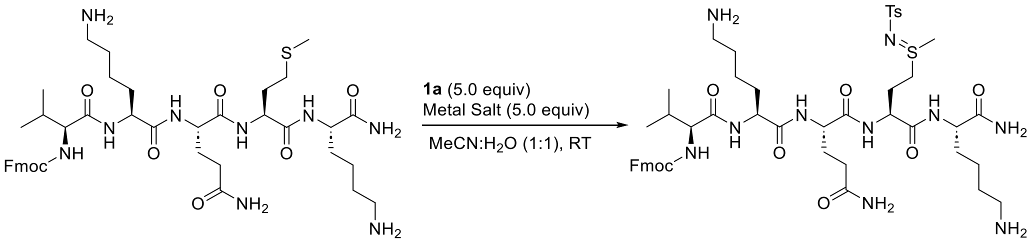

entry	Metal Salt	Product	Sulfoxide
1.	CuI	57%	43%
2.	CuBr	88%	12%
3.	CuCl	65%	35%
4.	Cu(Ph$_3$P)$_3$Br	58%	42%

5.	$Cu(MeCN)_4PF_6$	50%	50%
6.	CuCN	45%	55%
7.	$(CuOTf)_2$.Benzene	2%	2%
8.	$(CuOTf)_2$.toluene	65%	35%
9.	$Fe(OAc)_2$	44%	56%
10.	$FeCl_2$	48%	52%
11.	$Fe(acac)_3$	40%	60%
12.	$Pd(Ph_3P)_4$	2%	2%

Fmoc-VKQM(mod)K-CONH$_2$: LCMS *m/z* 1023.4793 (calc. [M+H$^+$] = 1023.4791), *m/z* 1045.4627 (calc. [M+Na$^+$] = 1045.4610), m/z 512.2437 (calc. [(M+2H$^+$)/2] = 512.2431), Purity: > 95 % (HPLC analysis at 220 nm). Retention time in HPLC: 20.952 min for product.

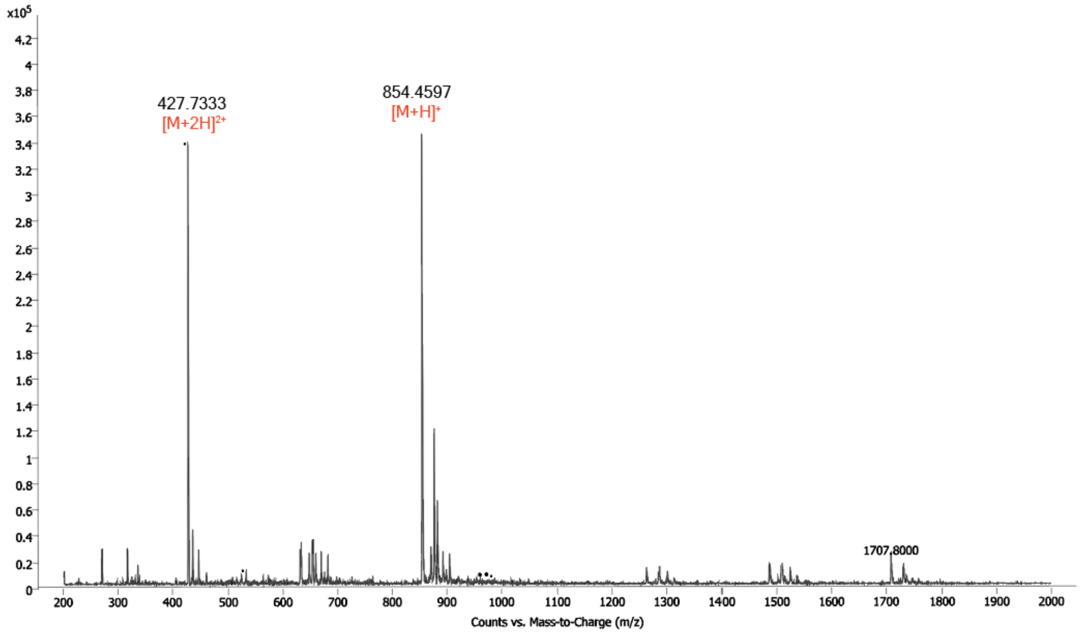

MS spectra of Fmoc-VKQMK-CONH$_2$

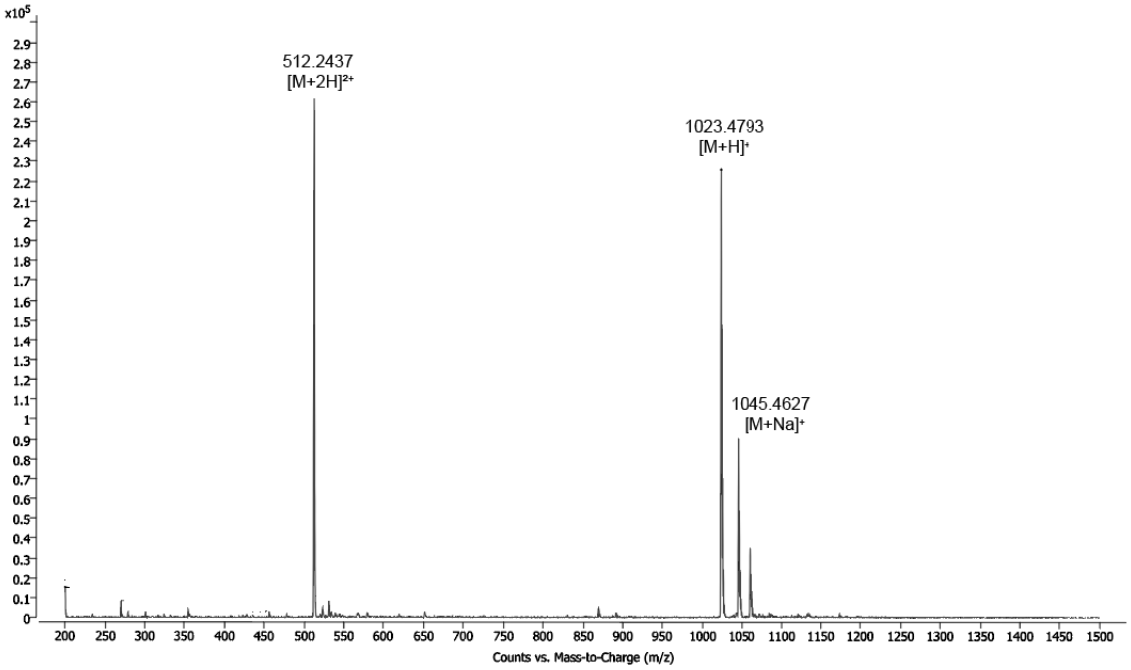

MS spectra of modified Fmoc-VKQMK-$CONH_2$

Supplementary Fig. 8. Energetics for acetonitrile liganded copper nitrene complex.

To computationally probe the effect of acetonitrile on stabilizing the copper-nitrene complex, we optimized the geometries of copper-nitrene complex without acetonitrile, and copper-nitrene-acetonitrile complex. Analysis of charge distribution and sum of electronic and thermal free energies of the system clearly identified acetonitrile liganded copper-nitrene complex to be thermodynamically stable (-2780.49) and possess higher hydrolytic stability as observed from the less positive charge density on the Cu (Q_{Cu} = +0.103). Calculations were done using the **general computational details** above.

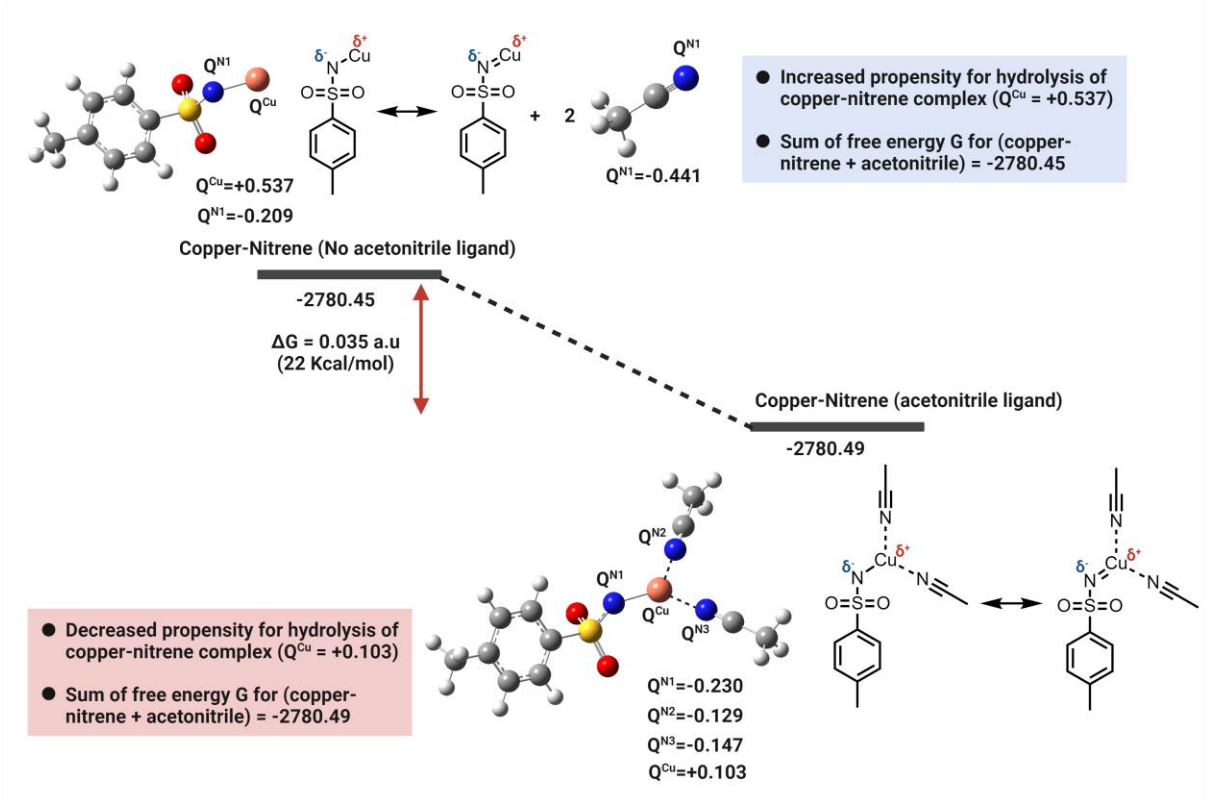

Supplementary Fig. 9. Screening of bidentate ligands.

Fmoc-VKQMK-CONH$_2$ (1 mg, 0.12 µmol, 1.0 equiv), CuBr (0.83 mg, 0.60 µmol, 5.0 equiv), ligand (0.24 µmol, 0.2 equiv) and **1a** (1.33 mg, 0.60 µmol, 5.0 equiv) were dissolved in MeCN:H$_2$O (1:1, 200 µL) under nitrogen atmosphere and stirred for 5 h at RT. The reaction was quenched by adding 10 µL of 0.5 M HCl and analyzed on HPLC (Gradient: 0-70% solvent B over 30 min, solvent B: 0.1% formic acid in MeCN) and MS.

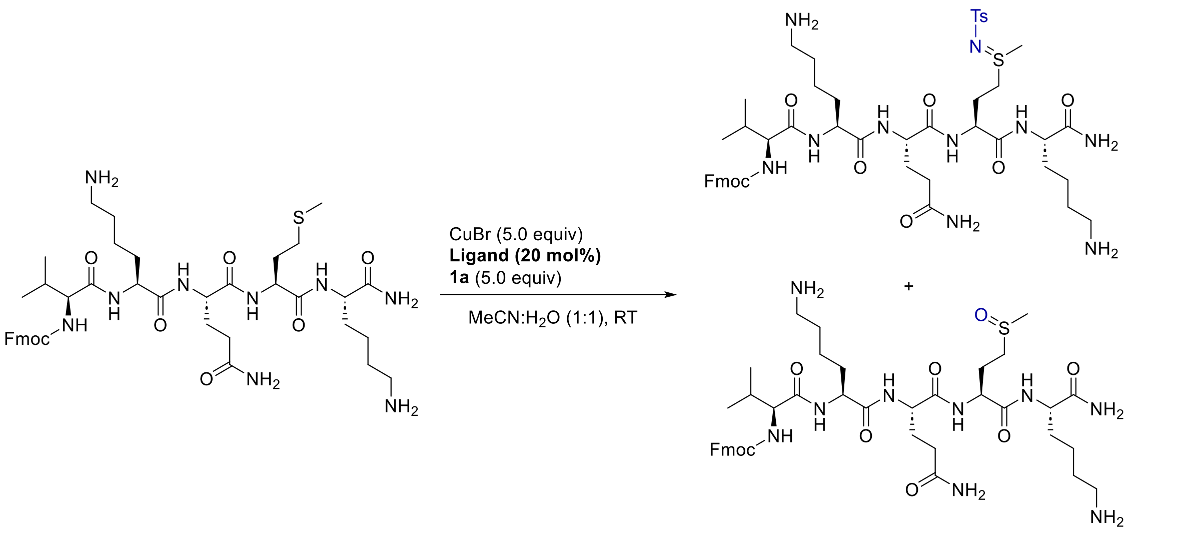

entry	Ligand	Product:Sulfoxide
1.	Pyridine	Complex mixture
2.	2,2'-Bipyridyl	Complex mixture
3.	1,10-Phenanthroline	Complex mixture

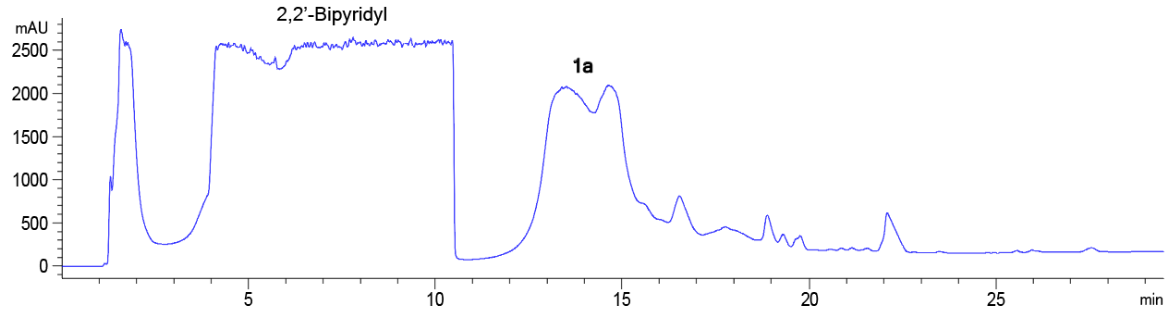

HPLC trace of the reaction mixture of Fmoc-VKQMK-$CONH_2$, **1a** (5 equiv), CuBr (5 equiv), in presence of 2,2'-Bipyridyl (40 mol%) as ligand shows complex reaction mixture.

Supplementary Fig. 10. Evaluation of the interaction energy of the HOMO of methionine reacting with the LUMO of copper-nitrene-acetonitrile ligand.

To understand the underlying mechanisms of the reaction between the copper-nitrene complex and methionine, we analyzed the interaction energy between the highest occupied molecular orbital (HOMO) of methionine and the lowest unoccupied molecular orbital (LUMO) of the copper-nitrene complex bound to the ligand. The calculated energy for this interaction was notably lower (ΔeV_1 = 5.40 eV) compared to the interaction between the HOMO of the copper-nitrene complex and the LUMO of methionine (ΔeV_2 = 5.97 eV). This outcome confirms that the electron pair in the HOMO of methionine initiates an attack on the copper's LUMO orbital in the copper-nitrene complex. Calculations were done using the **general computational details** above.

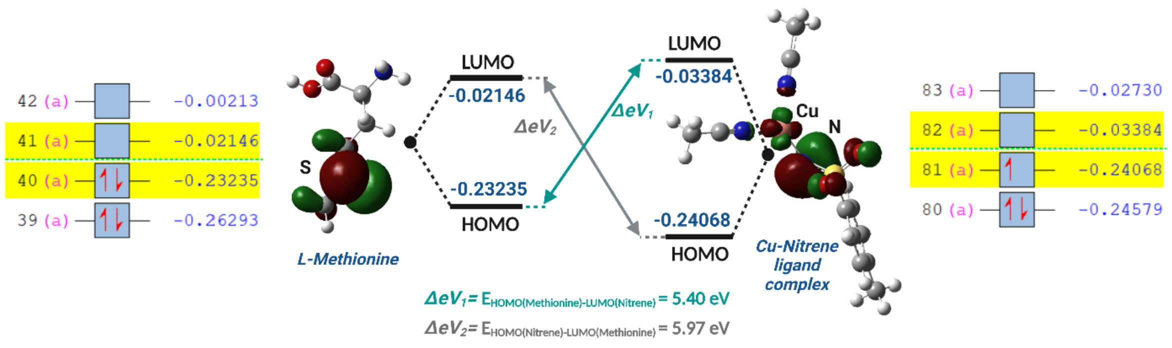

Supplementary Fig. 11. Computational evaluation of chemoselectivity of methionine and reactive amino acid residues reaction with copper-nitrene-acetonitrile complex.

To ascertain the chemoselectivity, we computationally evaluated the energetics of the reaction with other reactive amino acid residues. From our results, we observed that the energy gap between the HOMO of methionine and the LUMO of the MeCN-bound copper-nitrene complex is the lowest among all the reactive amino acid residues except for tryptophan. Calculations were done using the **general computational details** above.

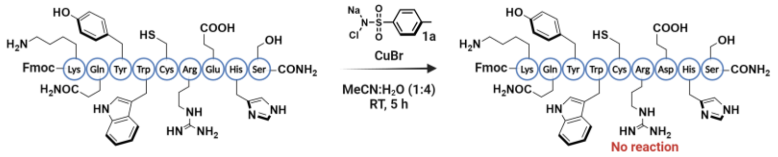

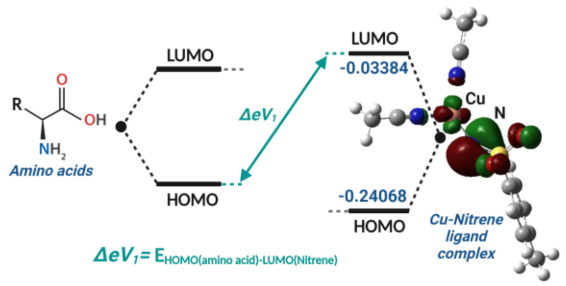

AMINO ACID RESIDUES	HOMO Amino acid	LUMO Nitrene	HOMO-LUMO GAP	HOMO-LUMO GAP (eV)
TRYPTOPHAN	-0.21512	-0.03384	0.18128	4.915226
**METHIONINE**	**-0.23235**	**-0.03384**	**0.19851**	**5.38240**
TYROSINE	-0.23373	-0.03384	0.19989	5.419817
HISTIDINE	-0.23683	-0.03384	0.20299	5.503871
ARGININE	-0.24192	-0.03384	0.20808	5.641881
CYSTEINE	-0.25285	-0.03384	0.21901	5.938237
ASPARTIC ACID	-0.27428	-0.03384	0.24044	6.519290
LYSINE	-0.25056	-0.03384	0.21672	5.876146
ASPARAGINE	-0.26049	-0.03384	0.22665	6.145388
SERINE	-0.26360	-0.03384	0.22976	6.229713
N-TERMINUS (GLY-OMe)	-0.26313	-0.03384	0.22929	6.216969
C-TERMINUS (NAc-GLY)	-0.27365	-0.03384	0.23981	6.502208

Supplementary Fig. 11 was created with BioRender.com, released under a Creative Commons Attribution-NonCommercial-NoDerivs 4.0 International license".

Supplementary Fig. 12. Chemoselectivity studies on Fmoc-KQYWCREHS-CONH2.

Negative Control:

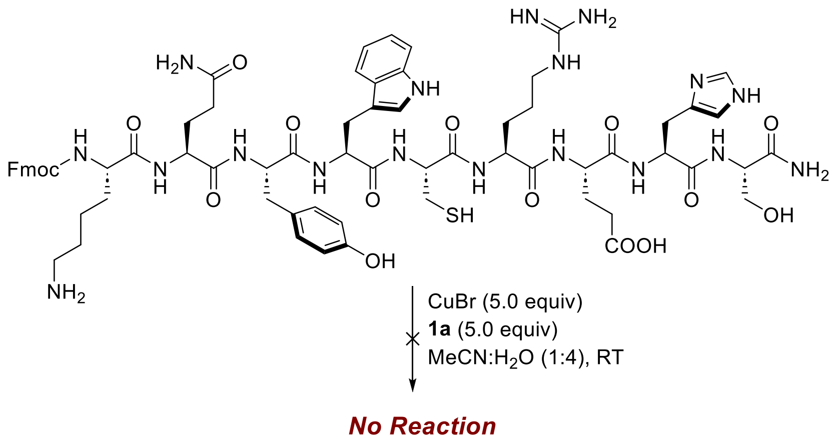

Fmoc-KQYWCREHS-CONH$_2$ (1 mg, 0.7 µmol, 1.0 equiv), CuBr (0.5 mg, 3.5 µmol, 5.0 equiv), and **1a** (0.795 mg, 3.5 µmol, 5.0 equiv) were dissolved in MeCN:H_2O (1:4, 500 µL) under nitrogen atmosphere and stirred at RT for 24 h. The reaction was quenched with 10 µL of 0.5 M HCl and analyzed on HPLC (Gradient: 0-70 % solvent B over 30 min, solvent B: 0.1% formic acid in MeCN) and MS.

Fmoc-KQYWCREHS-CONH$_2$: LCMS *m/z* 1457.6423 (calc. [M+H$^+$] = 1457.6419), *m/z* 729.3243 (calc. [(M+2H$^+$)/2] = 729.3246), *m/z* 486.5542 (calc. 486.5521). Purity: > 95 % (HPLC analysis at 220 nm). Retention time in HPLC: 6.164 min.

Recovered Fmoc-KQYWCREHS-CONH$_2$: LCMS *m/z* 1457.6437 (calc. [M+H$^+$] = 1457.6419), *m/z* 729.3237 (calc. [(M+2H$^+$)/2] = 729.3246), *m/z* 486.5557 (calc. 486.5521). Purity: > 95 % (HPLC analysis at 220 nm). Retention time in HPLC: 9.712 min.

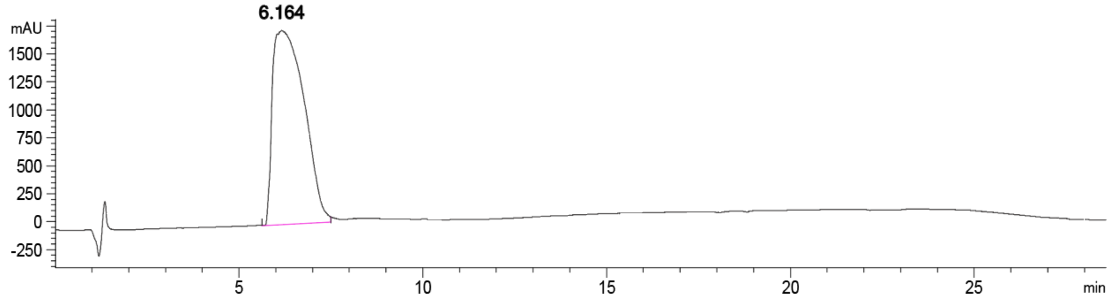

HPLC trace of Fmoc-KQYWCREHS-CONH$_2$

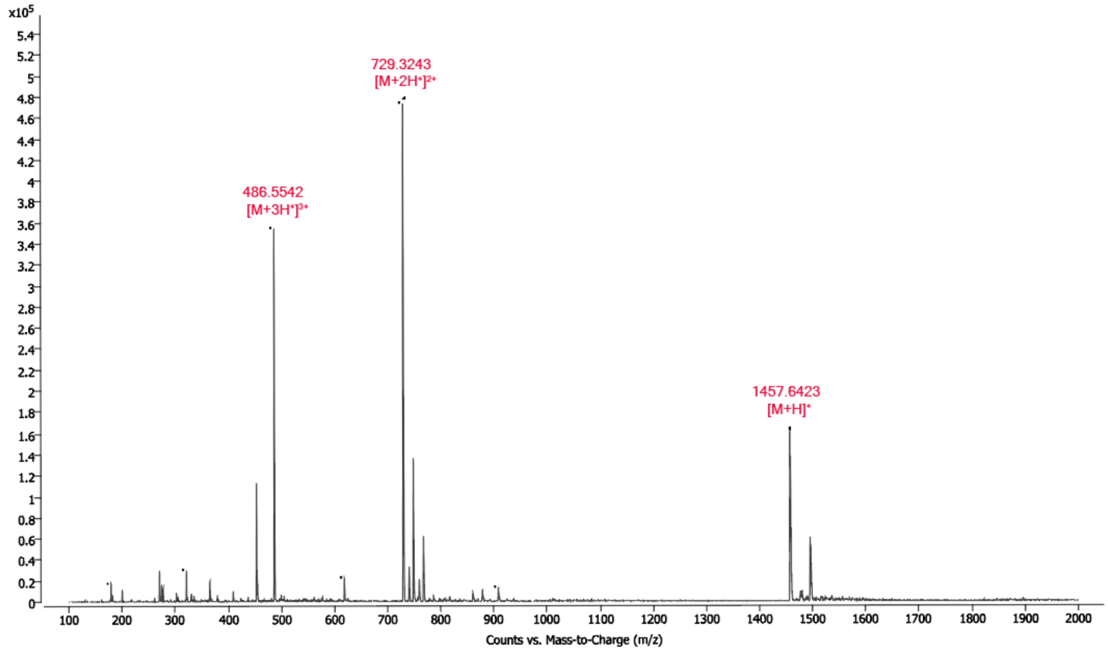

MS spectra of Fmoc-KQYWCREHS-CONH$_2$

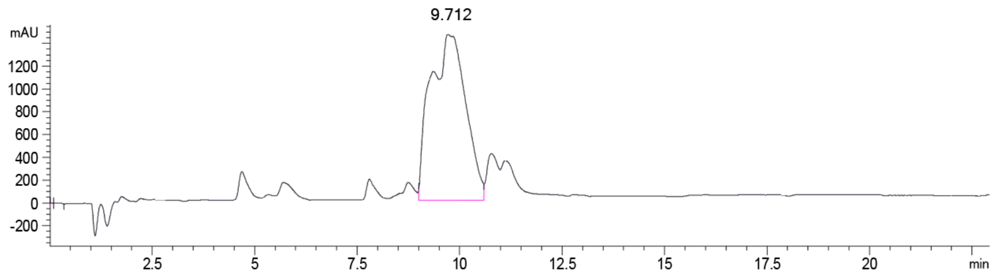

HPLC trace of the reaction mixture after 24 h

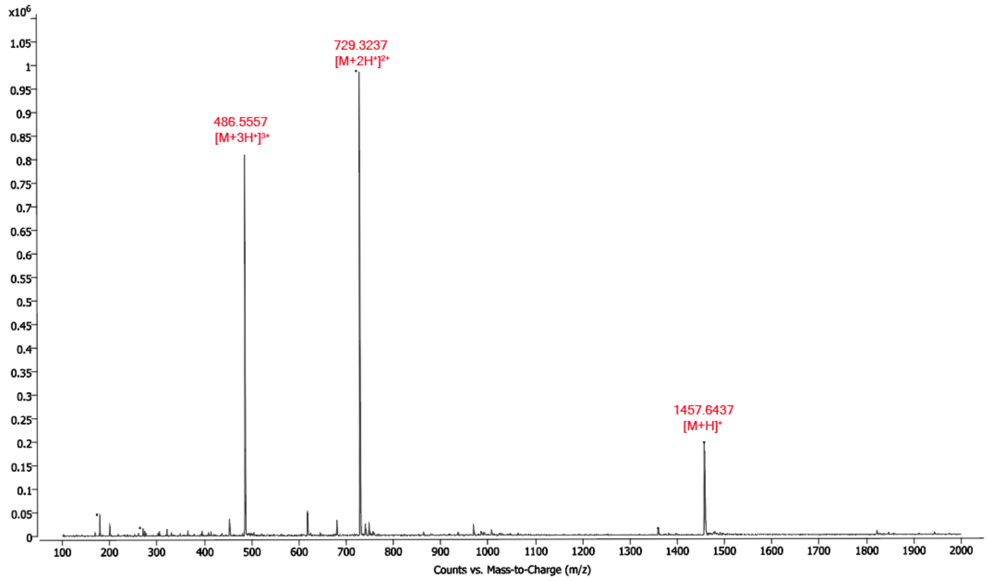

MS spectra of the recovered Fmoc-KQYWCREHS-$CONH_2$ after 24 h

Positive Control:

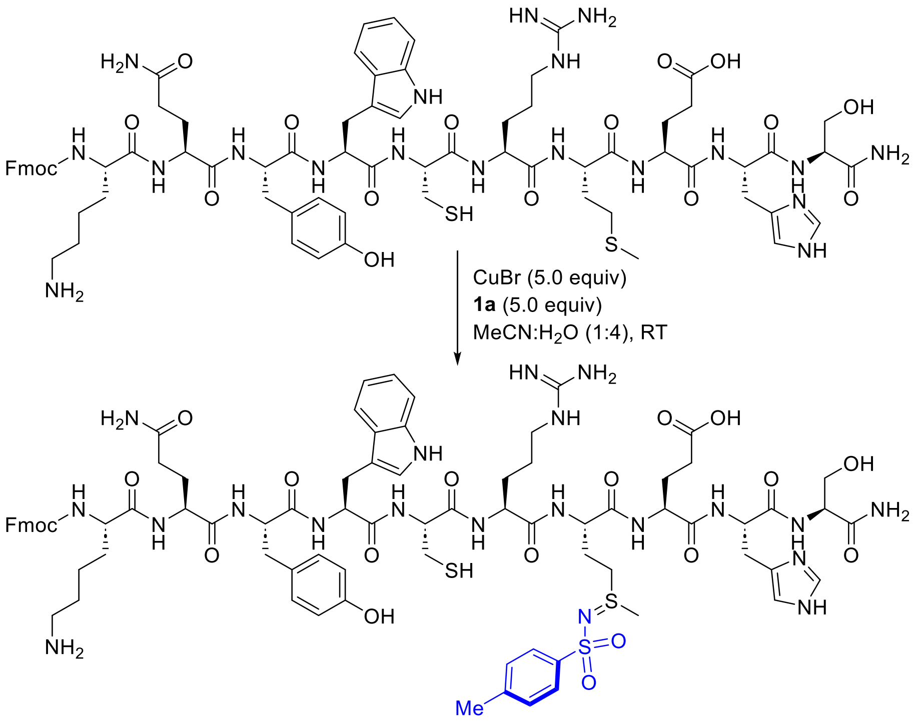

Fmoc-KQYWCRMEHS-CONH$_2$ (1 mg, 0.6 µmol, 1.0 equiv), CuBr (0.5 mg, 3.0 µmol, 5.0 equiv), and **1a** (0.75 mg, 3.0 µmol, 5.0 equiv) were dissolved in MeCN:H_2O (1:4, 500 µL) under nitrogen atmosphere and stirred at RT for 5 h. The reaction was quenched with 10 µL of 0.5 M HCl and analyzed on HPLC (Gradient: 0-70 % solvent B over 30 min, solvent B: 0.1% formic acid in MeCN) and MS. HPLC analysis shows 79% sulfimidated product.

Fmoc-KQYWCRMEHS-CONH$_2$: LCMS *m/z* 1588.6826 (calc. [M+H$^+$] = 1588.6824), *m/z* 794.8452 (calc. [(M+2H$^+$)/2] = 794.8448), *m/z* 530.2335 (calc. [(M+3H$^+$)/3] = 530.2323). Purity: > 95 % (HPLC analysis at 220 nm). Retention time in HPLC: 11.024 min.

Fmoc-KQYWCRM(mod)EHS-CONH$_2$: LCMS *m/z* 1757.7026 (calc. [M+H$^+$] = 1757.7022), *m/z* 879.3552 (calc. [(M+2H$^+$)/2] = 879.3547), *m/z* 586.5734 (calc. [(M+3H$^+$)/3] = 586.5722). Purity: > 95 % (HPLC analysis at 220 nm). Retention time in HPLC: 11.699 min.

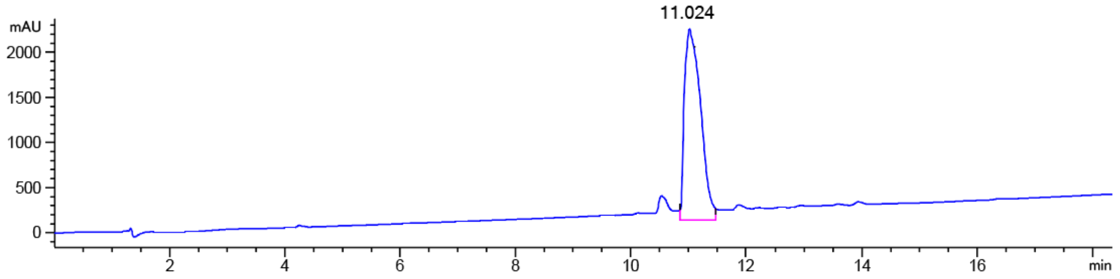

HPLC trace of Fmoc-KQYWCRMEHS-CONH$_2$

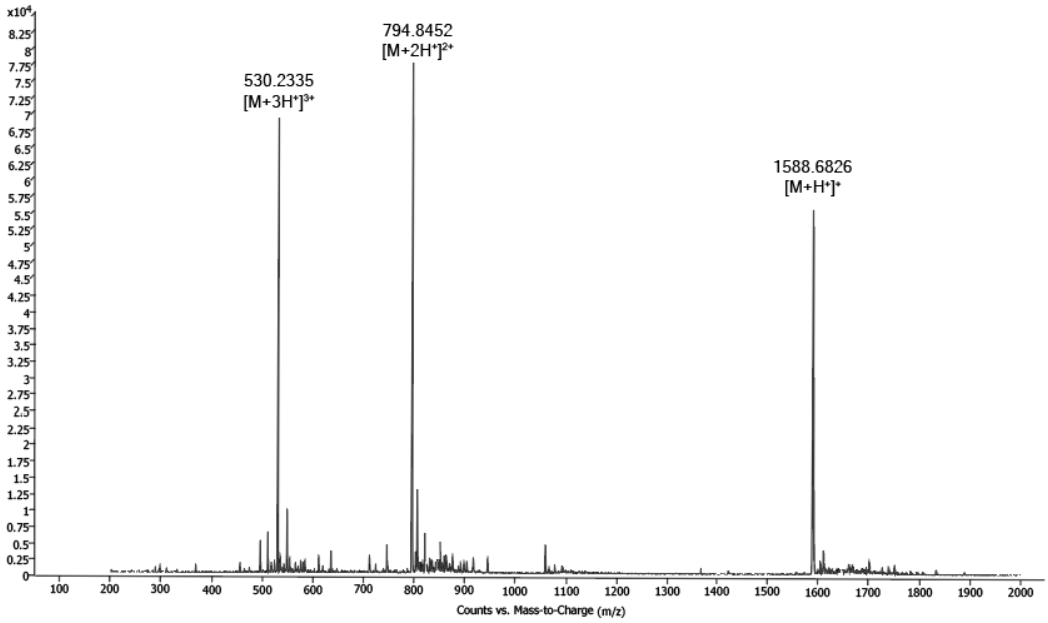

MS Spectra of Fmoc-KQYWCRMEHS-$CONH_2$

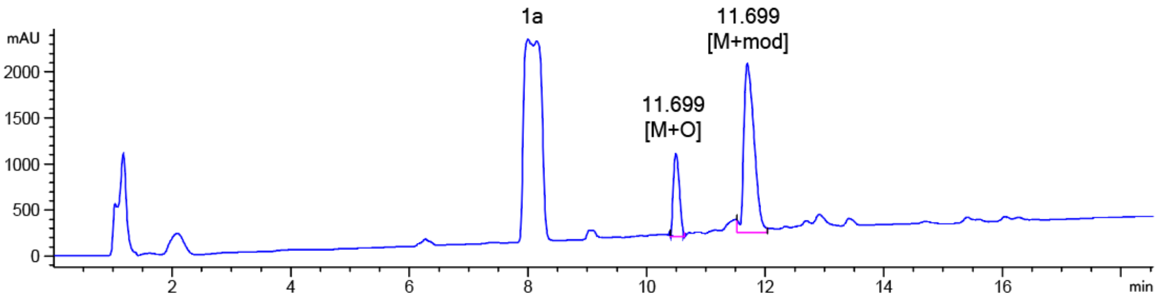

Peak #	RetTime [min]	Type	Width [min]	Area [mAU*s]	Height [mAU]	Area %
1	10.487	MM	0.1631	1.43402e4	1464.95813	21.2177
2	11.699	MM	0.2020	2.22254e4	1833.51257	78.7823

HPLC trace of **1a** modified Fmoc-KQYWCRMEHS-$CONH_2$

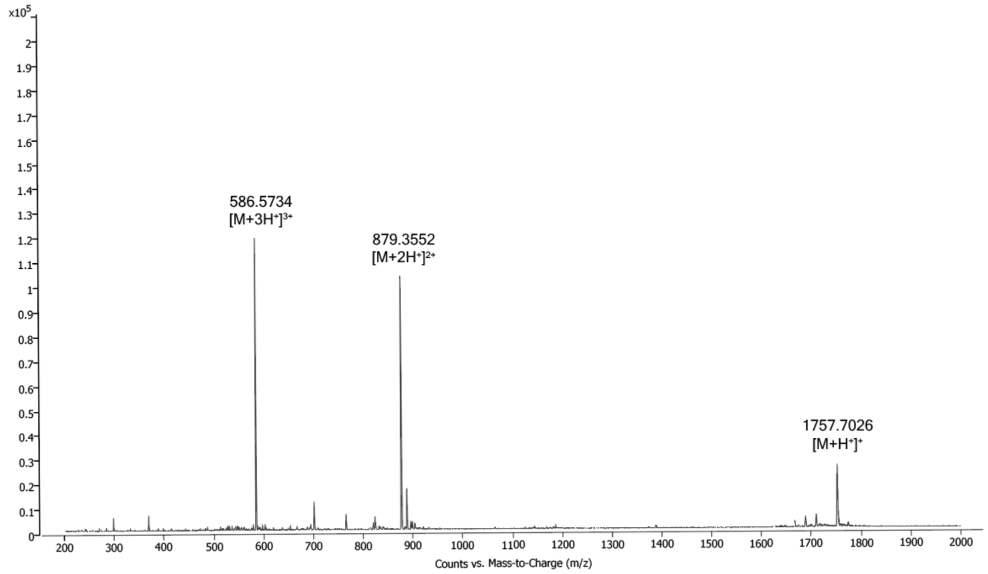

MS Spectra of **1a** modified Fmoc-KQYWCRMEHS-$CONH_2$

Plausible explanations for the lack of reactivity of nitrene with tryptophan:

Although the calculations indicate that tryptophan may potentially react with the copper-nitrene-acetonitrile complex, our hypothesis suggests that the lack of reactivity observed could be attributed to the significant resonance stabilization and dispersion of the pi electrons within the highest occupied molecular orbital (HOMO) of tryptophan. Calculations were done using the **general computational details** above.

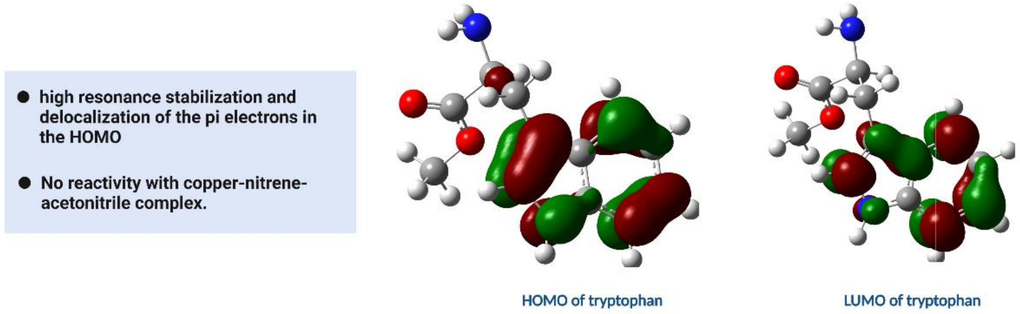

Supplementary Fig. 13. Experimental and computational evaluation of sulfimide stability.

To determine the rate of decomposition of the sulfonyl sulfimide conjugate of Ac-Met-OMe with **1a**, resulting in the formation of the corresponding sulfoxide, we incubated the sulfimide product of Ac-Met-OMe (9.26 mg, 0.025 mmol) in MeOD (0.7 mL) at a temperature of 37°C monitored for decomposition using NMR at various time intervals. The outcomes revealed the excellent stability of the sulfonyl sulfimide conjugate, as there was no detectable decomposition even after 96 hours and 7 days.

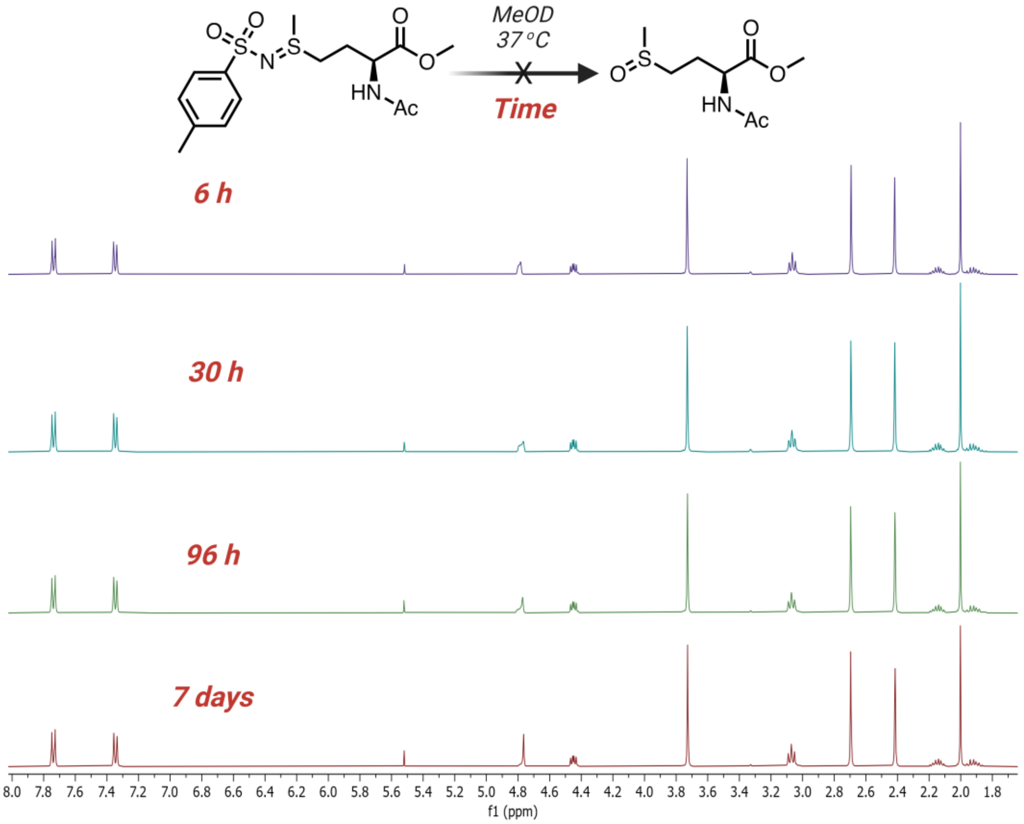

To gain more insight regarding the observed stability of sulfonyl sulfimides, we conducted DFT calculations on **1a** modified sulfonyl sulfimide of Ac-Met-OMe. The calculations showed a high electron density on the nitrogen atom of the sulfonyl sulfimide resulting in a predominantly stable S-N double bond character with reduced electrophilicity and enhanced hydrolytic stability of the sulfimide. This consequently leads to an increase in the HOMO-LUMO gap of the sulfonyl sulfimide product. Calculations were done using the **general computational details** above.

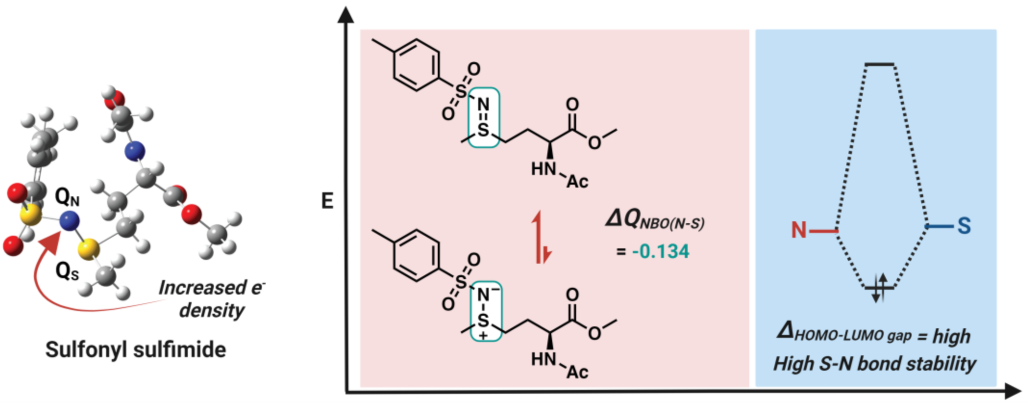

Supplementary Fig. 14. pH and temperature stability studies of the conjugated product of Fmoc-MF-OMe

1 mg of sulfonyl-sulfimide conjugate of Fmoc-MF-OMe was dissolved in MeCN:10 mM NaP buffer (1:1, 200 µL) at the following pH values: 3.0, 5.0, 9.0, and 12.0. The reactions were incubated at RT for 24 h and analyzed using LC-MS. No degradation of sulfonyl-sulfimide product was observed.

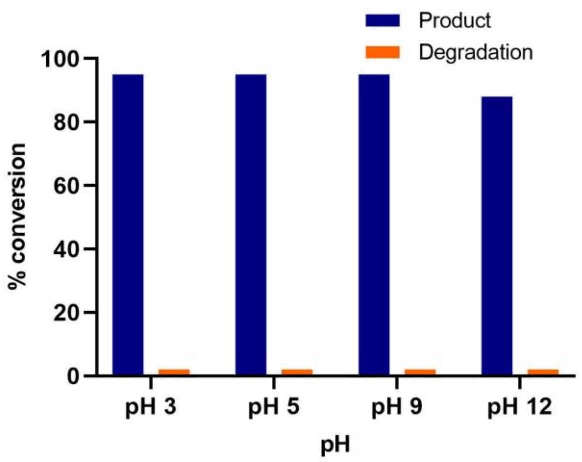

1 mg of sulfonyl-sulfimide conjugate of Fmoc-MF-OMe was dissolved in MeCN:10 mM NaP buffer pH 7 (1:1, 200 µL). The reactions were incubated at room temperature and 60 ^{O}C for 24 h and analyzed using LC-MS. No degradation of sulfonyl-sulfimide product was observed.

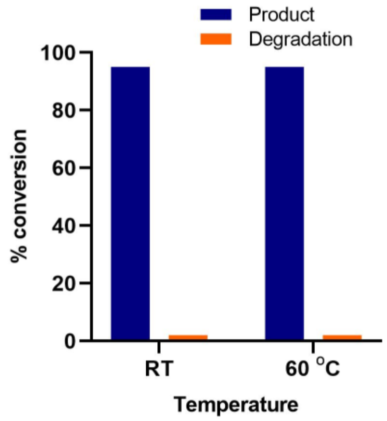

Supplementary Fig. 15. Evaluation of electronic effect of substituents on CuNiP reaction.

Synthesis of the Sulfonamides:

The following sulfonamides were purchased from Sigma-Aldrich and used as received.

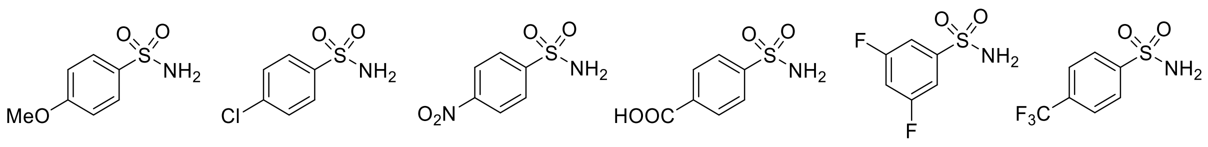

General Procedure for the Synthesis of Probes:

General Procedure I: To a solution of sulfonamide (1.0 equiv) in MeOH (0.2 M) was added trichloroisocyanuric acid (TCCA, 0.33 equiv). The reaction mixture was stirred at room temperature for 2 h and the solvent was removed under reduced pressure. Toluene was added to the resulting white precipitate and then filtered through a pad of celite. The filtrate was concentrated under reduced pressure and to the resulting liquid was subsequently added MeOH. The solution was then cooled to 0 °C and a solution of sodium hydroxide (1.0 equiv) in methanol (0.3 M) was slowly added. The reaction was allowed to stir at room temperature for 2 h. The solvent was removed under reduced pressure to afford the Chloramine-T analogues.

General Procedure II: To a solution of sulfonamide (3.0 mmol, 1.0 equiv) in H_2O (6 mL), was added crushed NaOH (120 mg, 3.0 mmol, 1.0 equiv) at 0 °C. After 5 min, once sulfonamide is dissolved then 15% NaOCl solution (2 mL, 3.15 mmol, 1.05 equiv) was dropwisely added over 10 min. The resulting reaction mixture was slowly allowed to come room temperature and stirred for 24 h. The appeared solid was filtered and washed with cold ether thrice. Finally, the resulting solid was dried under vacuum to give the Chloramine-T analogues.

Sodium chloro((4-methoxyphenyl)sulfonyl)amide (1b)

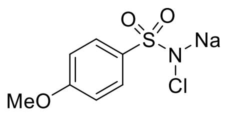

The titled compound was synthesized according to general procedure II by using 4-methoxybenzenesulfonamide (561 mg, 3.0 mmol), NaOH (120 mg, 3.0 mmol), 15% NaOCl solution (2 mL, 3.15 equiv, 1.05 equiv). The resulting product **1b** was isolated as white solid (424 mg, 58%). ^{1}**H NMR** (400 MHz, DMSO-d_6): δ = 7.56 (d, J = 7.5 Hz, 2H), 6.91 (d, J = 8.7 Hz, 2H), 3.76 (s, 3H) ppm. ^{13}**C NMR** (101 MHz, DMSO-d_6): δ = 160.00, 137.64, 128.66, 112.94, 55.26 ppm. **HRMS:** calcd. for $C_7H_7ClNNa_2O_3S$ [M+Na$^+$] 265.9625; found 265.9672.

^{1}H NMR of Compound 1b

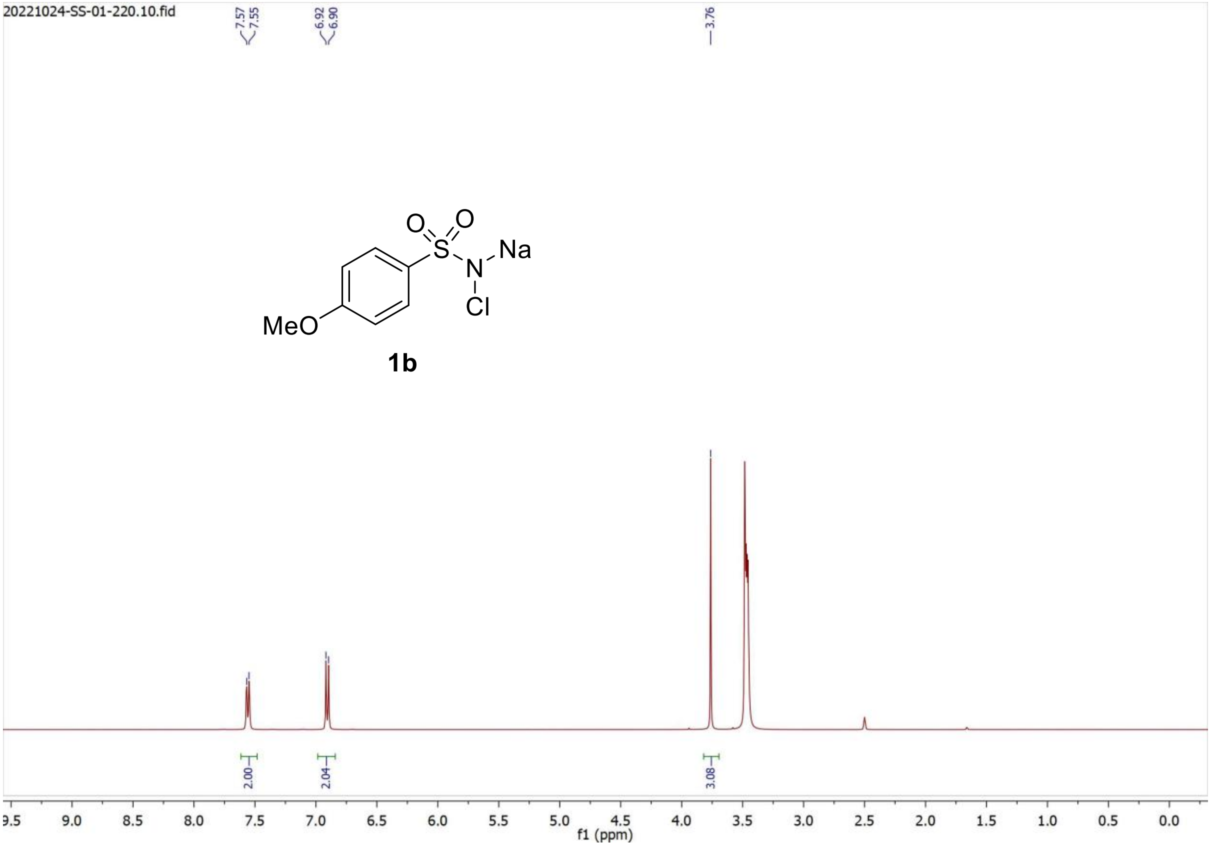

^{13}C NMR of Compound 1b

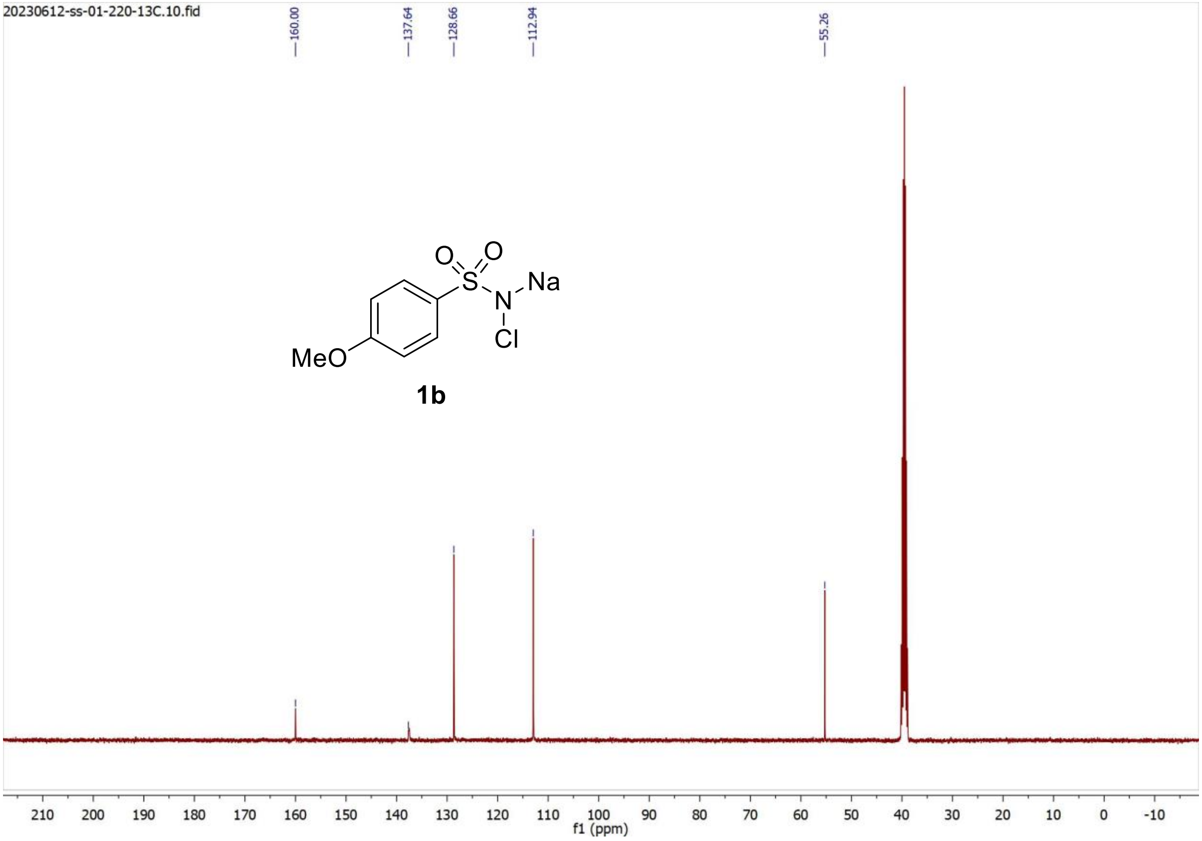

Sodium chloro((4-chlorophenyl)sulfonyl)amide (1c)

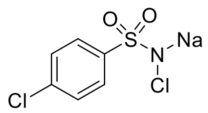

The titled compound was synthesized according to general procedure II by using 4-chlorobenzenesulfonamide (574 mg, 3.0 mmol), NaOH (120 mg, 3.0 mmol), 15% NaOCl solution (2 mL, 3.15 equiv, 1.05 equiv). The resulting product **1c** was isolated as white solid (320 mg, 43%). **^{1}H NMR** (400 MHz, DMSO-d_6): δ = 7.62 (d, J = 8.5 Hz, 2H), 7.44 (d, J = 8.5 Hz, 2H) ppm. **^{13}C NMR** (101 MHz, DMSO-d_6): δ = 144.25, 133.94, 128.78, 127.84 ppm. **HRMS:** calcd. for $C_6H_4Cl_2NNa_2O_2S$ [M+Na$^+$] 269.9130; found 269.9134.

^{1}H NMR of Compound 1c

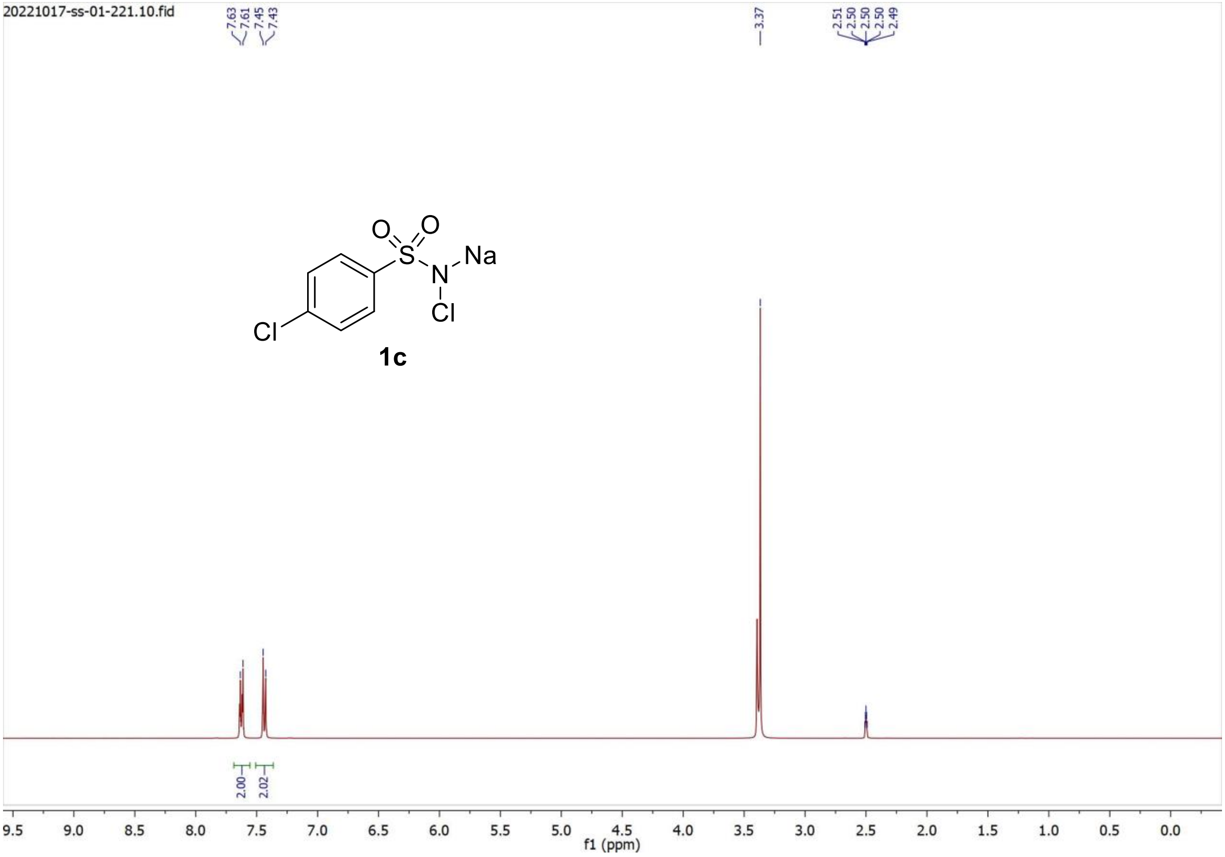

^{13}C NMR of Compound 1c

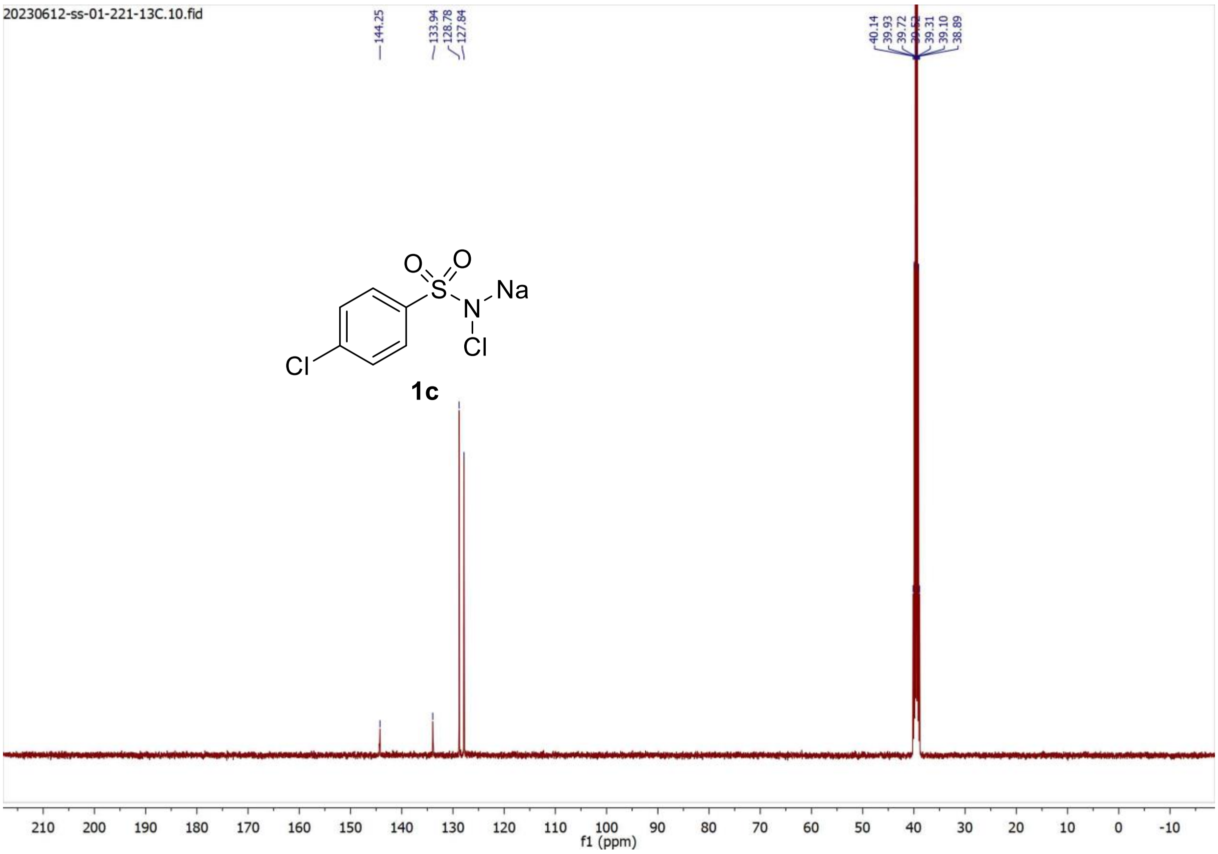

Sodium ((4-carboxylatophenyl)sulfonyl)chloroamide (1d)

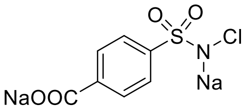

The titled compound was synthesized according to general procedure I by using 4-sulfamoylbenzoic acid (603 mg, 3.0 mmol), TCCT (232 mg, 1.0 mmol), and NaOH (240 mg, 6.0 mmol, 2.0 equiv). The resulting product **1d** was isolated as white solid (428 mg, 51% yield). **^{1}H NMR** (400 MHz, DMSO-*d*$_6$) δ = 7.90 (d, *J* = 8.2 Hz, 2H), 7.56 (d, *J* = 8.3 Hz, 2H) ppm. **^{13}C NMR** (101 MHz, DMSO-*d*$_6$) δ = 170.12, 145.85, 140.49, 128.51, 125.94 ppm. **HRMS:** calcd. for $C_7H_4ClNNa_3O_4S$ [M+Na$^+$] 301.9327; found 301.9332.

^{1}H NMR of Compound 1d

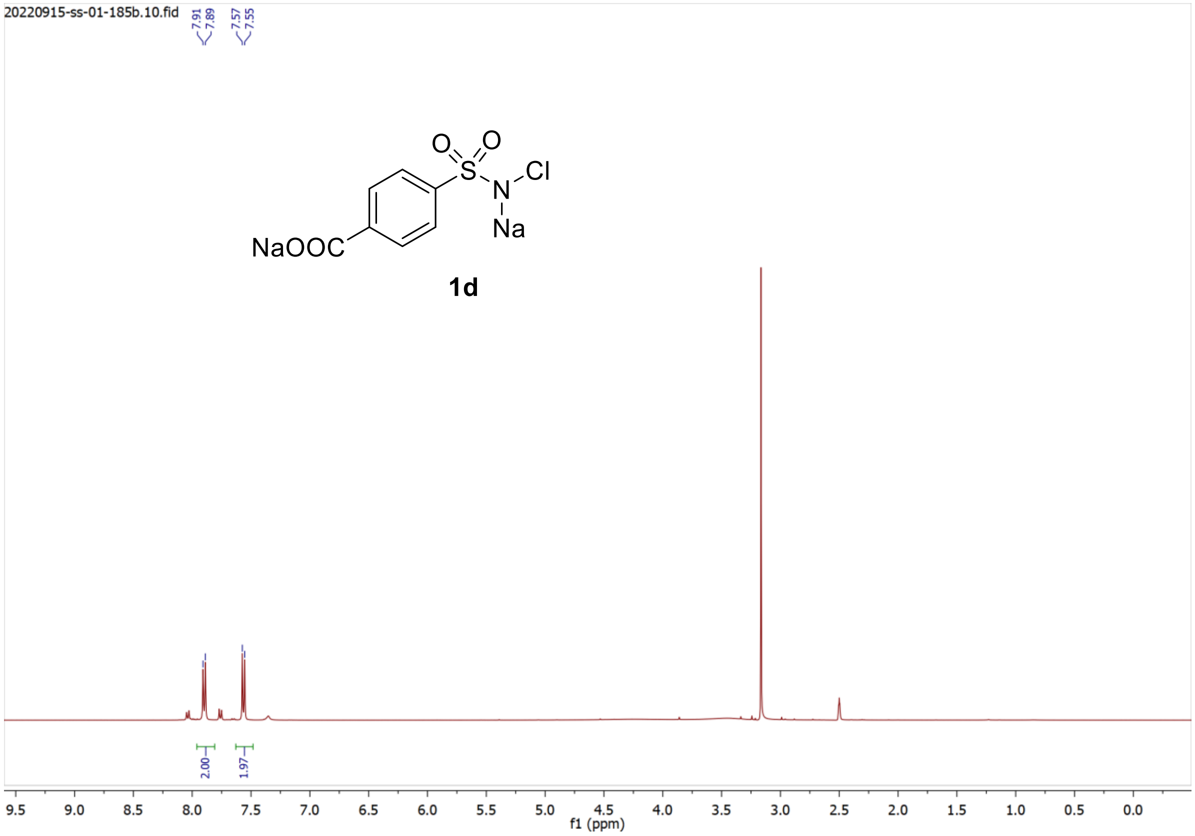

^{13}C NMR of Compound 1d

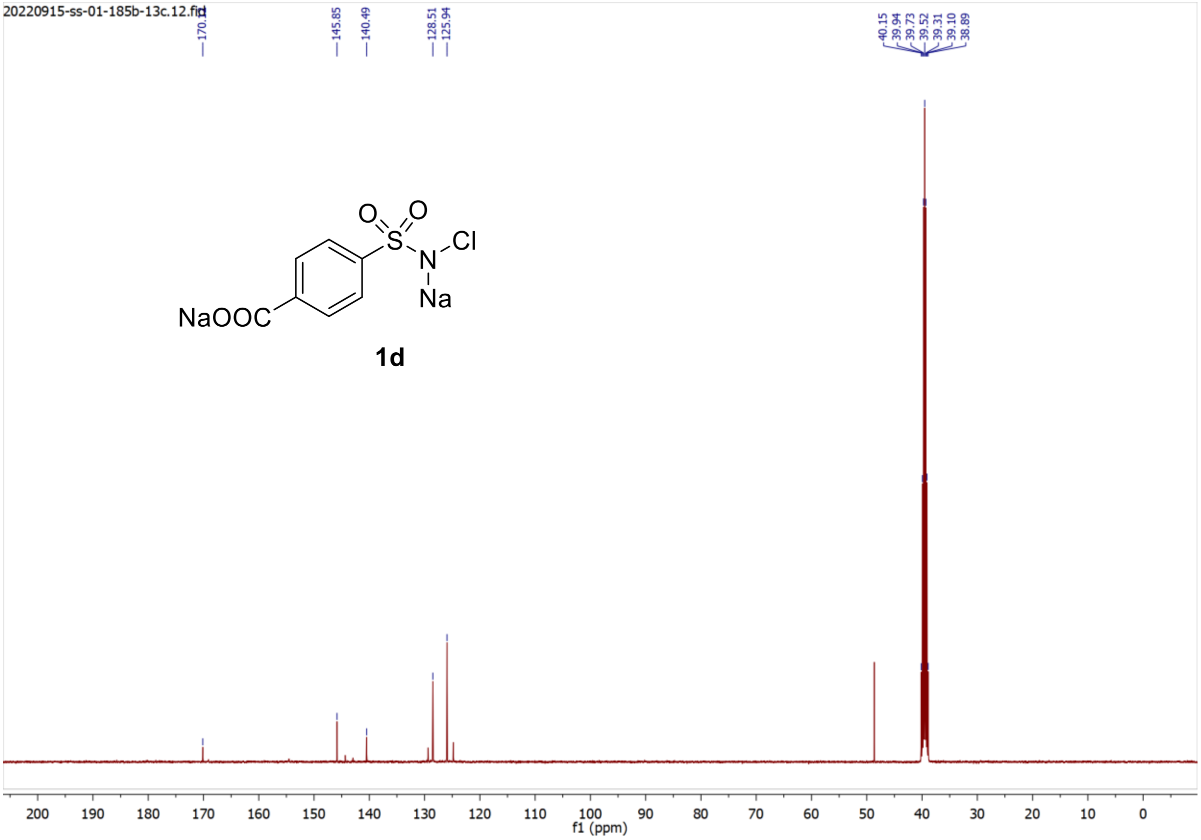

Sodium chloro((4-nitrophenyl)sulfonyl)amide (1e)

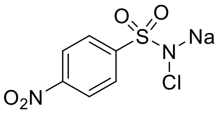

The titled compound was synthesized according to general procedure II by using 4-nitrobenzenesulfonamide (606 mg, 3.0 mmol), NaOH (120 mg, 3.0 mmol), 15% NaOCl solution (2 mL, 3.15 equiv, 1.05 equiv). The resulting product **1e** was isolated as white solid (237 mg, 31%). **^{1}H NMR** (400 MHz, DMSO-d_6): δ = 8.24 (d, J = 8.6 Hz, 2H), 7.86 (d, J = 8.8 Hz, 2H) ppm. **^{13}C NMR** (101 MHz, DMSO-d_6): δ = 151.45, 147.89, 128.22, 123.49 ppm. **HRMS:** calcd. for $C_6H_4ClN_2Na_2O_4S$ [M+Na+] 280.9370; found 280.9375.

^{1}H NMR of Compound 1e

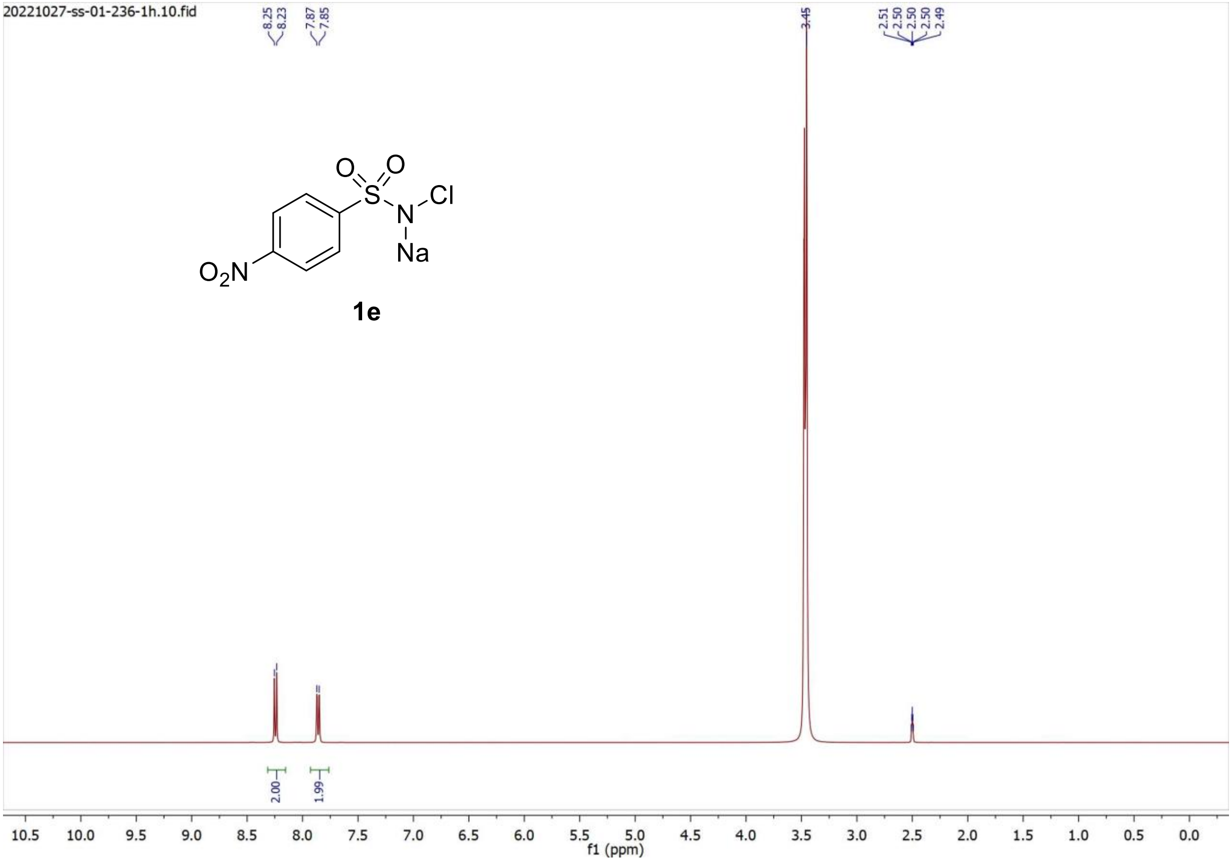

^{13}C NMR of Compound 1e

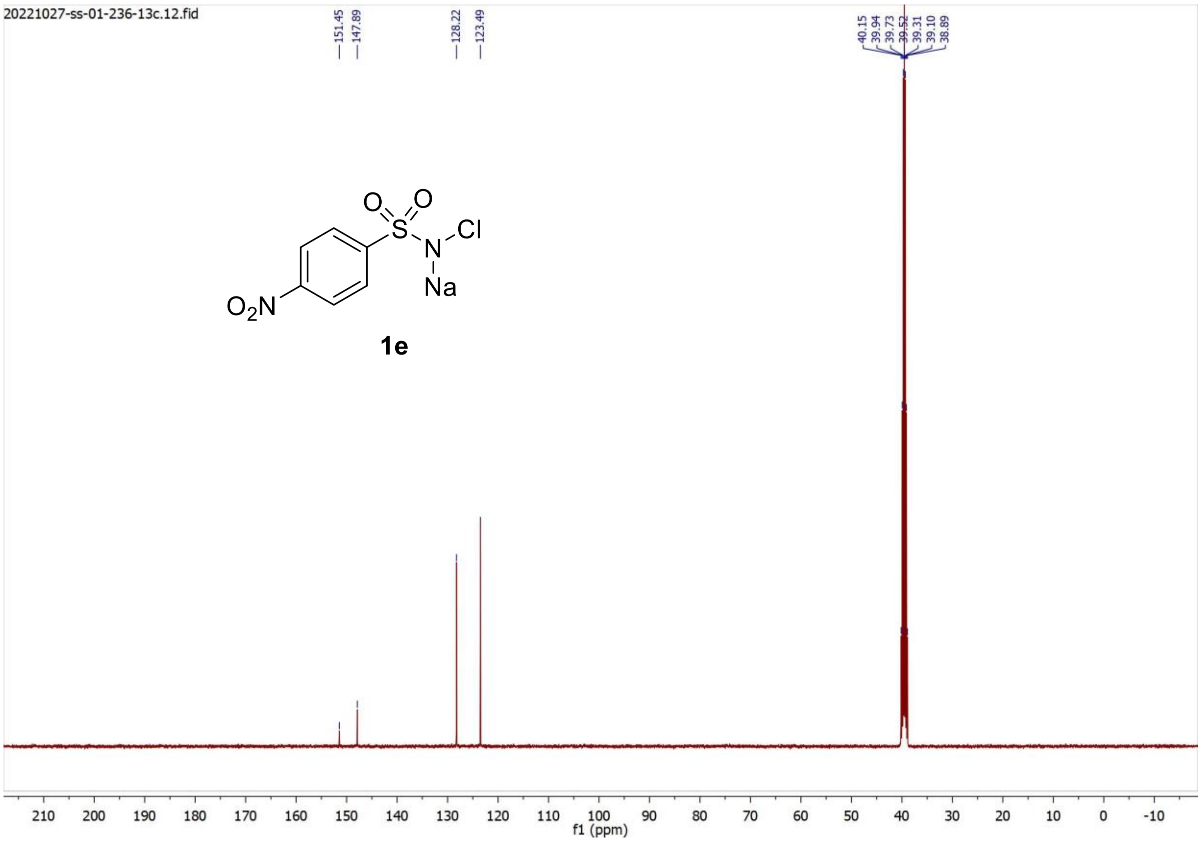

Sodium chloro((3,5-difluorophenyl)sulfonyl)amide (1f)

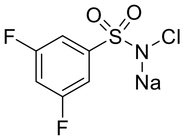

The titled compound was synthesized according to general procedure II by using 3,5-difluorobenzenesulfonamide (579 mg, 3.0 mmol), NaOH (120 mg, 3.0 mmol), 15% NaOCl solution (2 mL, 3.15 equiv, 1.05 equiv). The resulting product **1f** was isolated as white solid (345 mg, 46%). **^{1}H NMR** (400 MHz, DMSO-d_6) δ = 7.28 (tt, J = 9.3, 2.3 Hz, 1H), 7.24 – 7.20 (m, 2H) ppm. **^{13}C NMR** (101 MHz, DMSO-d_6) δ = 162.97 (d, J = 11.9 Hz), 160.50 (d, J = 12.1 Hz), 109.98 (d, J = 26.1 Hz), 104.90 (t, J = 24.7 Hz) ppm. **^{19}F NMR:** δ = -109.51 ppm. **HRMS:** calcd. for $C_6H_3ClF_2NNa_2O_2S$ [M+Na$^+$] 271.9331; found 271.9335.

^{1}H NMR of Compound 1f

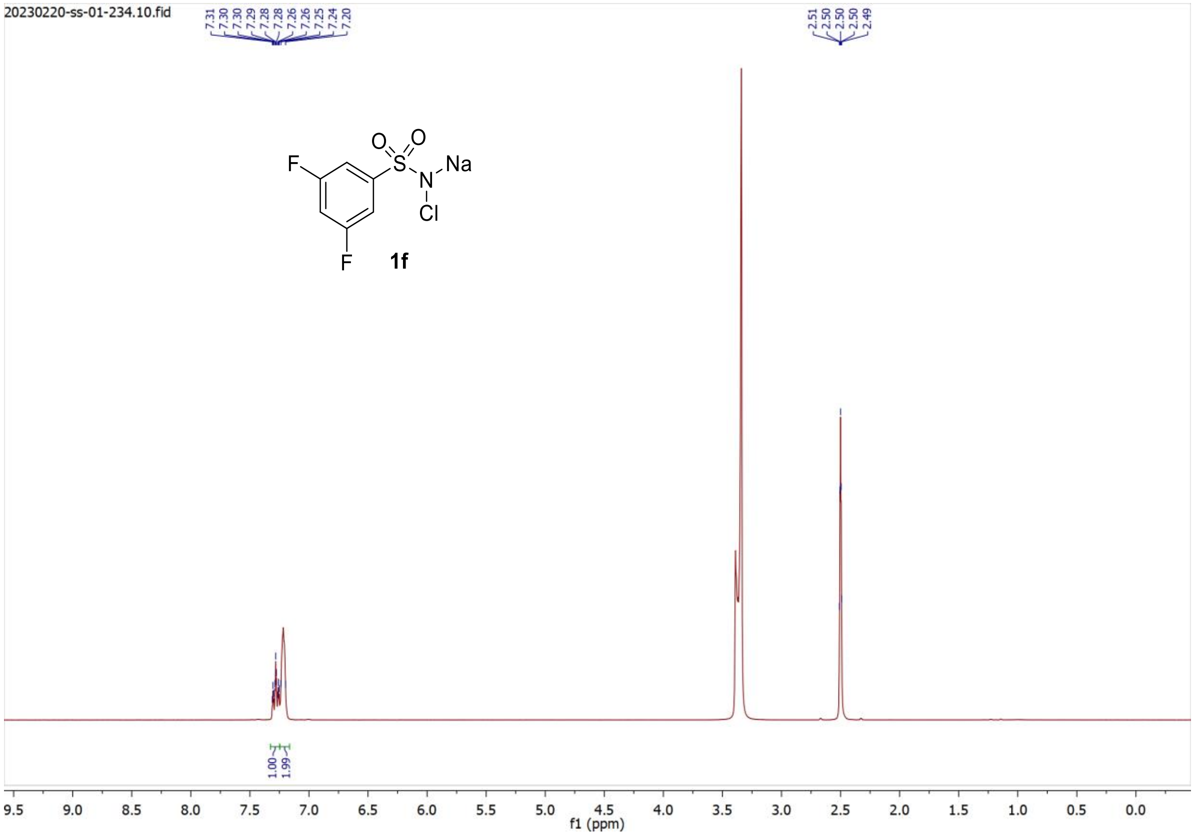

^{13}C NMR of Compound 1f

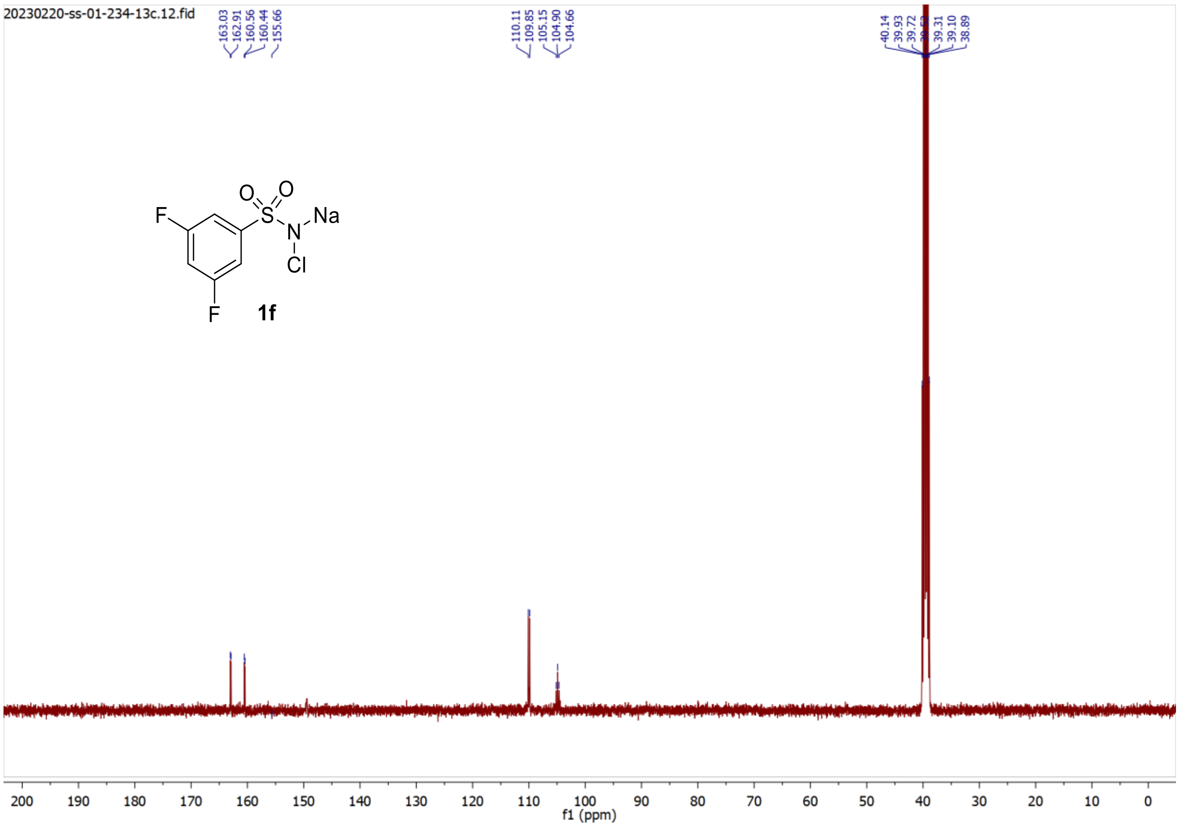

^{19}F NMR of Compound 1f

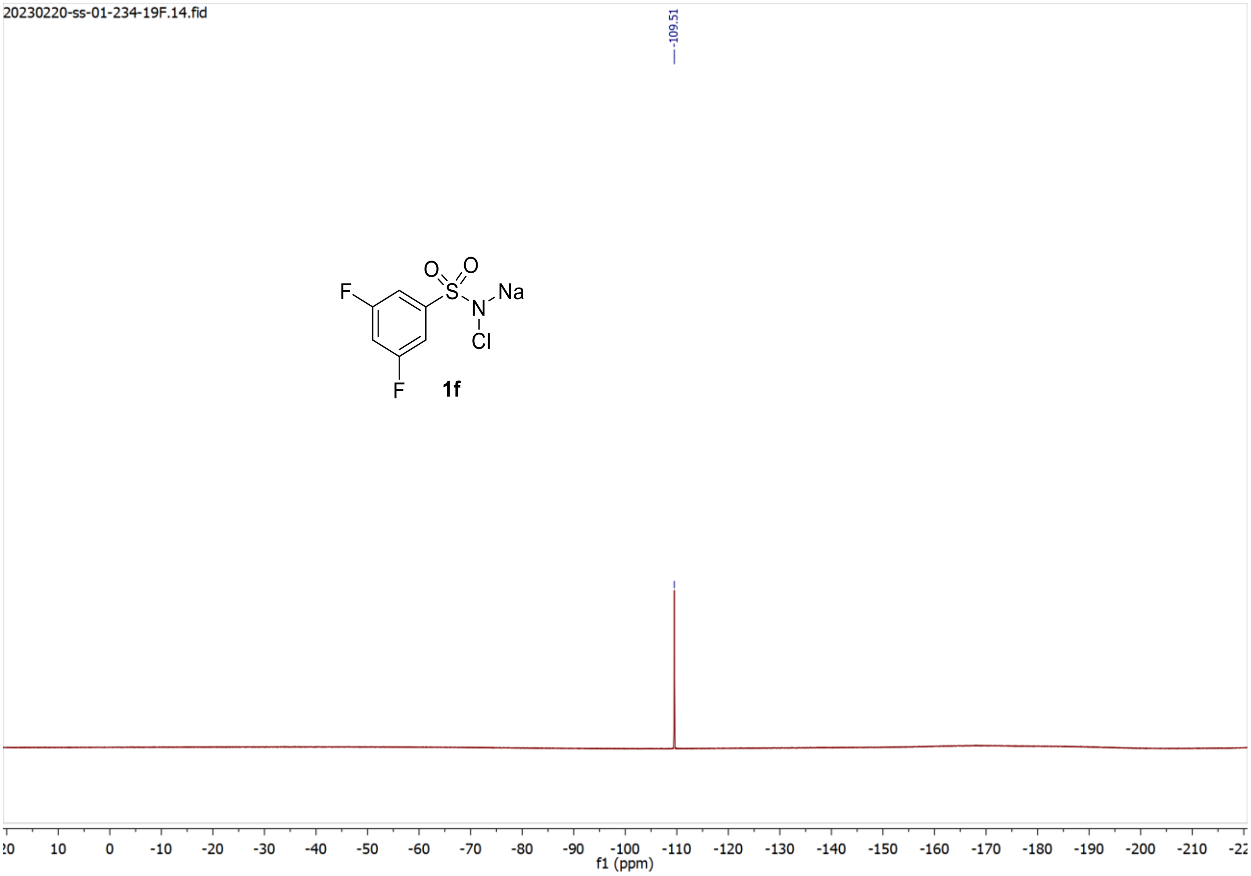

Sodium chloro((4-(trifluoromethyl)phenyl)sulfonyl)amide (1g)

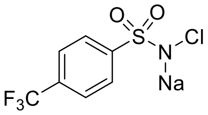

The titled compound was synthesized according to general procedure II by using 4-(trifluoromethyl)benzenesulfonamide (675 mg, 3.0 mmol), NaOH (120 mg, 3.0 mmol), 15% NaOCl solution (2 mL, 3.15 equiv, 1.05 equiv). The resulting product **1g** was isolated as white solid (540 mg, 64%). **^{1}H NMR** (400 MHz, DMSO-d_6) δ = 7.82 (d, J = 7.4 Hz, 2H), 7.76 (d, J = 7.6 Hz, 2H) ppm. **^{13}C NMR** (101 MHz, DMSO-d_6) δ = 149.35 (d, J = 13.4 Hz), 127.62, 124. 37 (q, J = 274.31 Hz), 125.01 (q, J = 3.9 Hz) ppm. ^{19}F NMR: δ = -61.06 ppm. **HRMS:** calcd. for $C_7H_4ClF_3NNa_2O_2S$ [M+Na$^+$] 303.9393; found 303.9396.

^{1}H NMR of Compound 1g

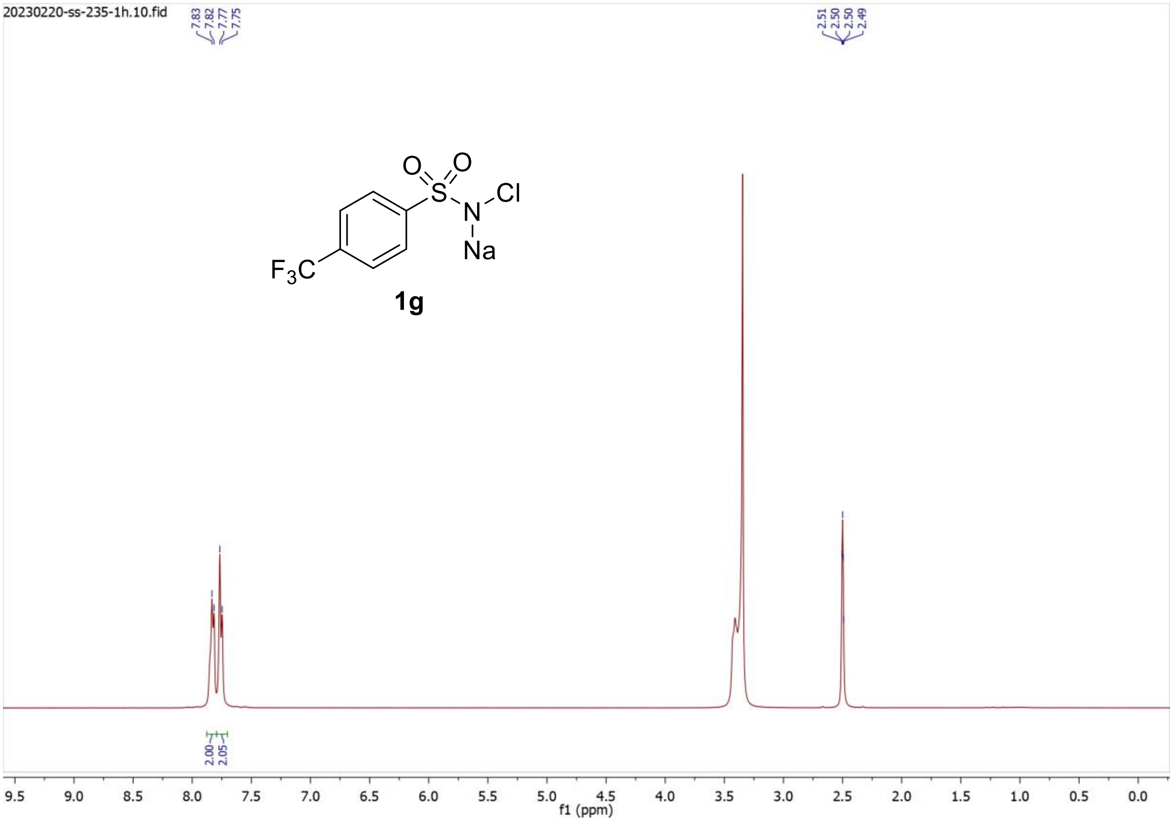

^{13}C NMR of Compound 1g

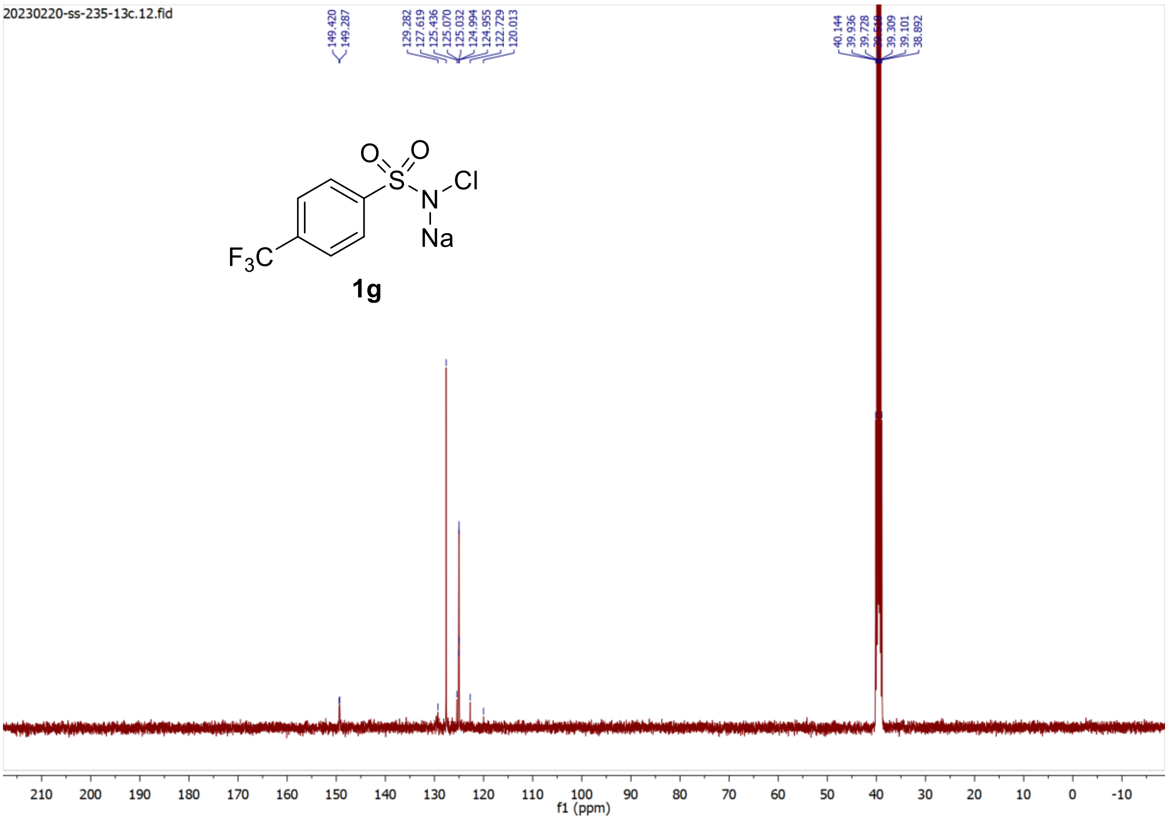

^{19}F NMR of Compound 1g

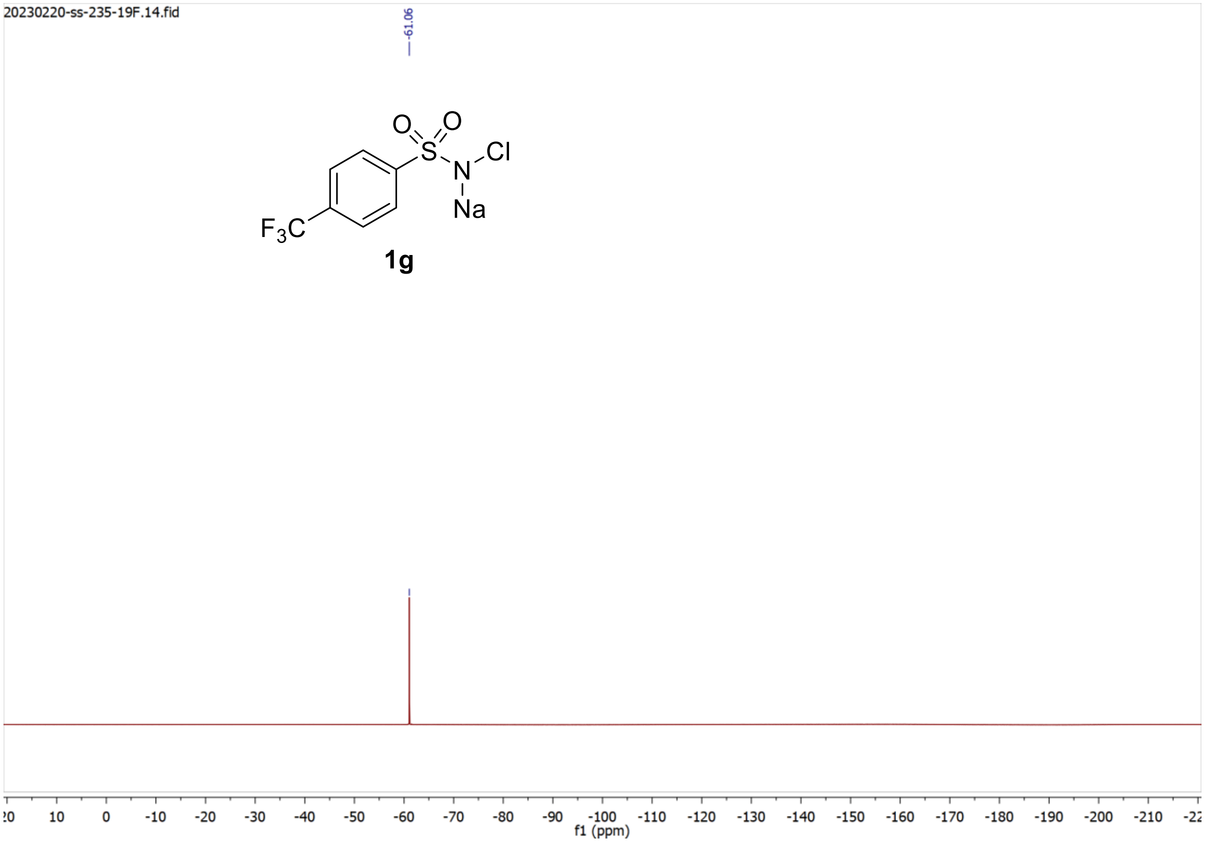

Screening of Ch-T analogs on pentapeptide

Pentapeptide Fmoc-VKQMK-NH_2 (1 mg, final conc. 2 mM) was incubated with CuBr (10 mM, 5.0 equiv) and **1a-1g** (10 mM, 5.0 equiv) at RT for 5 h under N_2. It was quenched with 20 µL 0.5 N HCl and the product:sulfoxide ratio was analyzed using HPLC and MS.

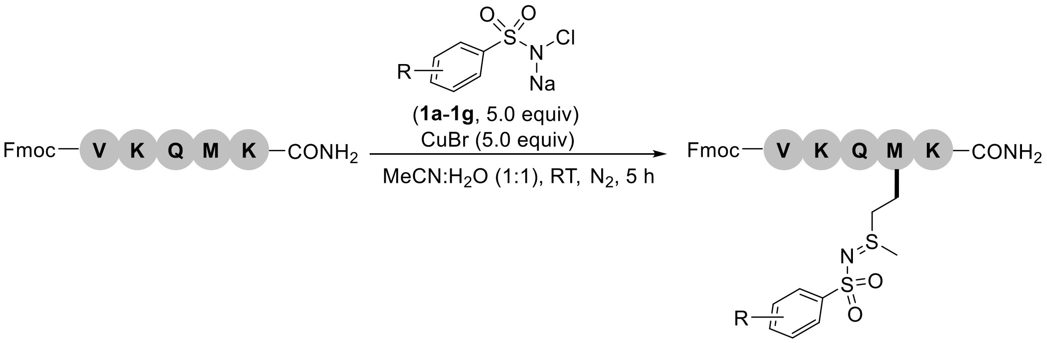

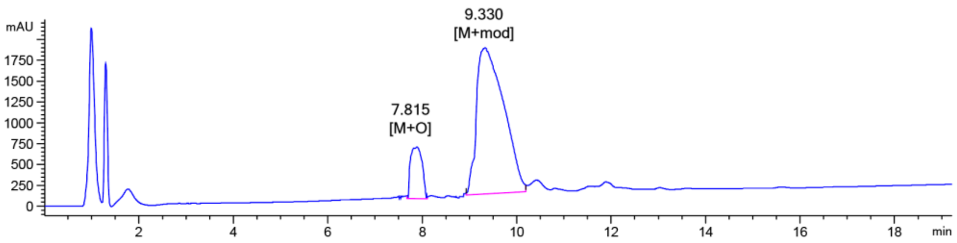

Peak #	RetTime [min]	Type	Width [min]	Area [mAU*s]	Height [mAU]	Area %
1	7.815	MM	0.6319	7.41530e4	1955.71399	13.3464
2	9.330	MM	0.6676	7.02642e4	1754.18481	86.6536

HPLC trace of **1a** modified Fmoc-VKQMK-CONH2

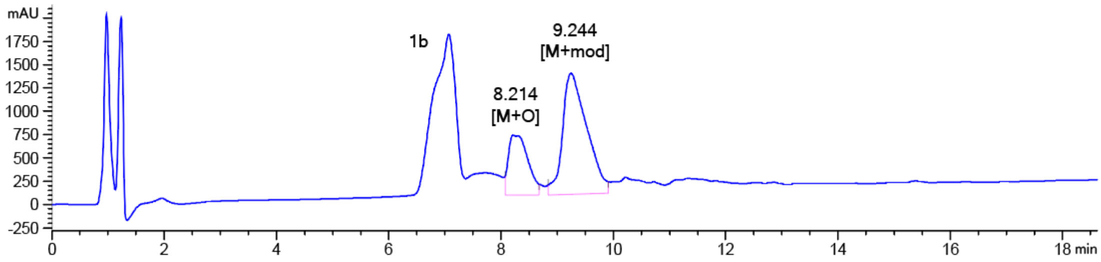

Peak #	RetTime [min]	Type	Width [min]	Area [mAU*s]	Height [mAU]	Area %
1	8.214	MM	0.4030	1.55027e4	641.07983	28.3298
2	9.244	MM	0.5031	3.92195e4	1299.27991	71.6702

HPLC trace of **1b** modified Fmoc-VKQMK-CONH2

Fmoc-VKQM(mod)K-CONH$_2$: LCMS *m/z* 1023.4745 (calc. [M+H$^+$] = 1039.4740), m/z 520.2410 (calc. [(M+2H$^+$)/2] = 520.2406), Purity: > 95 % (HPLC analysis at 220 nm). Retention time in HPLC: 9.244 min for product.

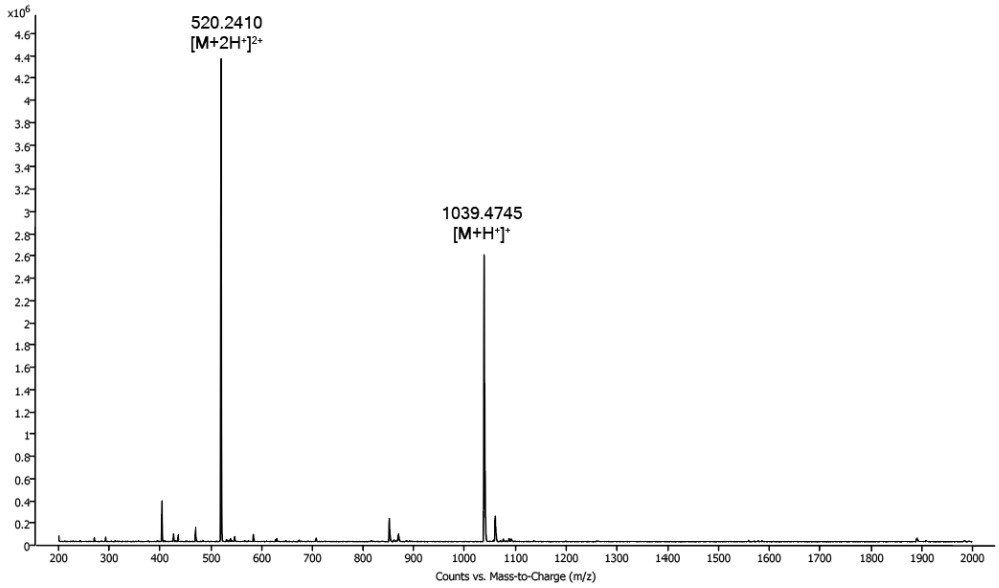

MS of **1b** modified Fmoc-VKQMK-CONH2

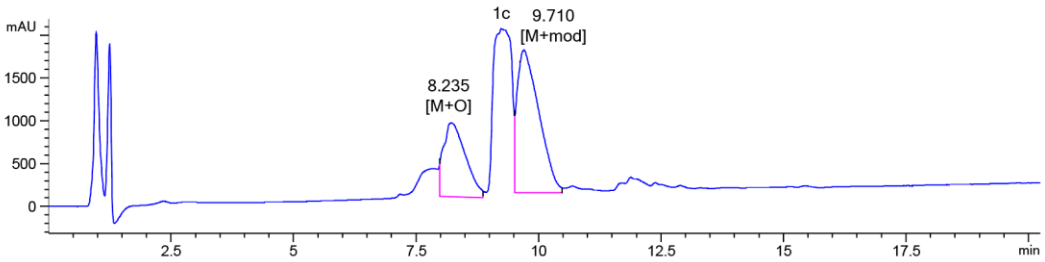

Peak #	RetTime [min]	Type	Width [min]	Area [mAU*s]	Height [mAU]	Area %
1	8.235	MM	0.5200	2.67718e4	858.06958	34.2180
2	9.710	MM	0.5143	5.14671e4	1668.02771	65.7820

HPLC trace of **1c** modified Fmoc-VKQMK-CONH2

Fmoc-VKQM(mod)K-CONH2: LCMS *m/z* 1043.4246 (calc. [M+H$^+$] = 1043.4244), m/z 522.2157 (calc. [(M+2H$^+$)/2] = 522.2158), Purity: > 95 % (HPLC analysis at 220 nm). Retention time in HPLC: 9.710 min for product.

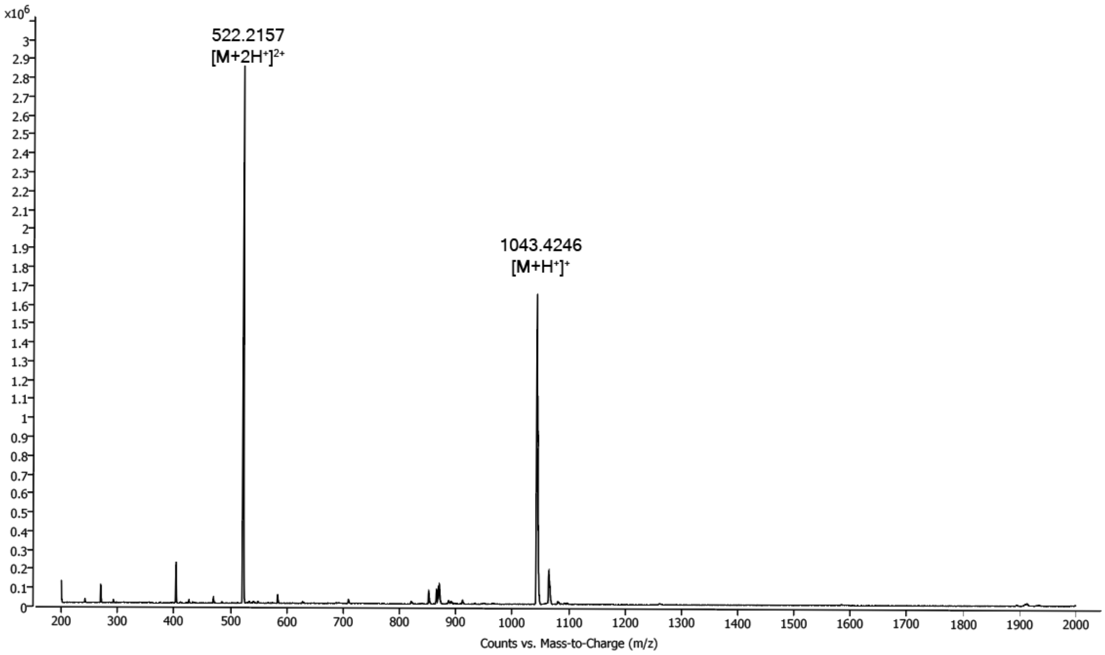

MS of **1c** modified Fmoc-VKQMK-CONH2

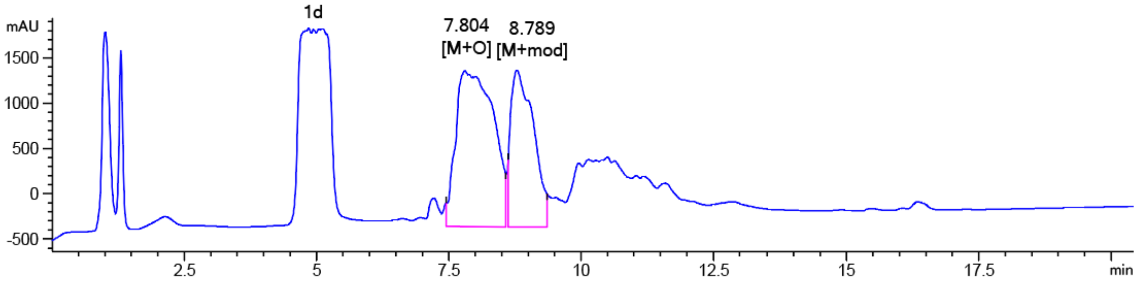

Peak #	RetTime [min]	Type	Width [min]	Area [mAU*s]	Height [mAU]	Area %
1	7.804	MM	0.8205	8.61880e4	1750.63684	62.4720
2	8.789	MM	0.4991	5.17747e4	1728.86890	37.5280

HPLC trace of **1d** modified Fmoc-VKQMK-$CONH_2$

Fmoc-VKQM(mod)K-$CONH_2$: LCMS *m/z* 1053.4536 (calc. $[M+H^+]$ = 1053.4532), m/z 527.2316 (calc. $[(M+2H^+)/2]$ = 527.2302), Purity: > 95 % (HPLC analysis at 220 nm). Retention time in HPLC: 8.789 min for product.

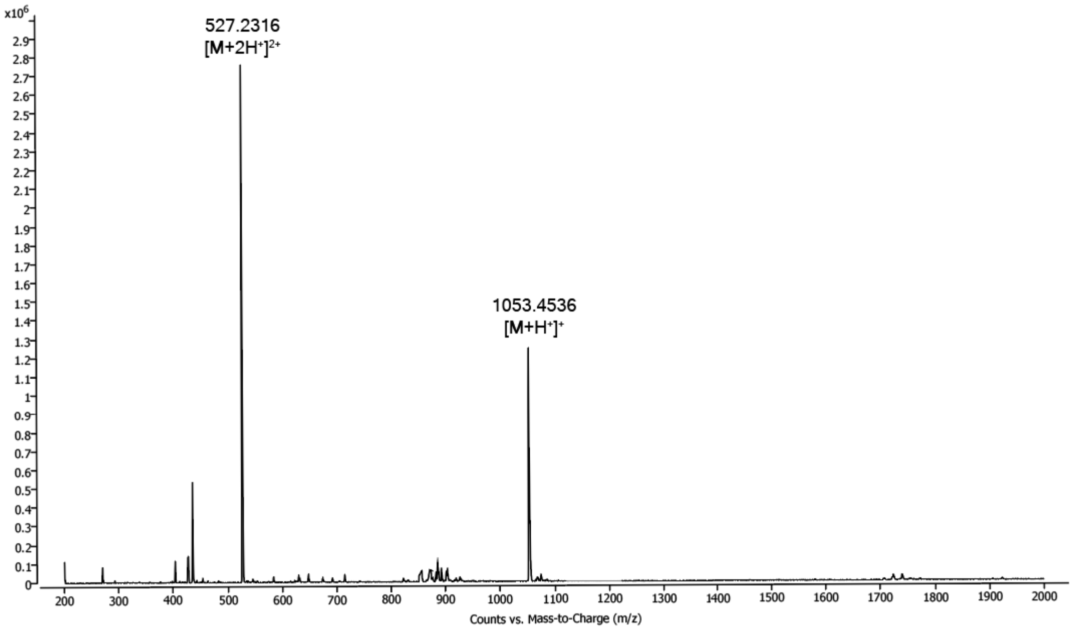

MS of **1d** modified Fmoc-VKQMK-$CONH_2$

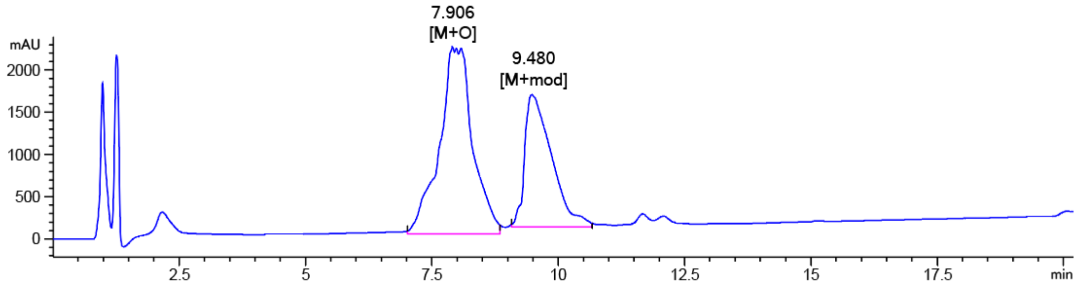

Peak #	RetTime [min]	Type	Width [min]	Area [mAU*s]	Height [mAU]	Area %
1	7.906	MM	0.7607	1.01101e5	2215.04761	63.4309
2	9.480	MM	0.6184	5.82867e4	1570.79346	36.5691

HPLC trace of **1e** modified Fmoc-VKQMK-$CONH_2$

Fmoc-VKQM(mod)K-$CONH_2$: LCMS *m/z* 1054.4479 (calc. $[M+H^+]$ = 1054.4485), m/z 527.7276 (calc. $[(M+2H^+)/2]$ = 527.7279), Purity: > 95 % (HPLC analysis at 220 nm). Retention time in HPLC: 9.480 min for product.

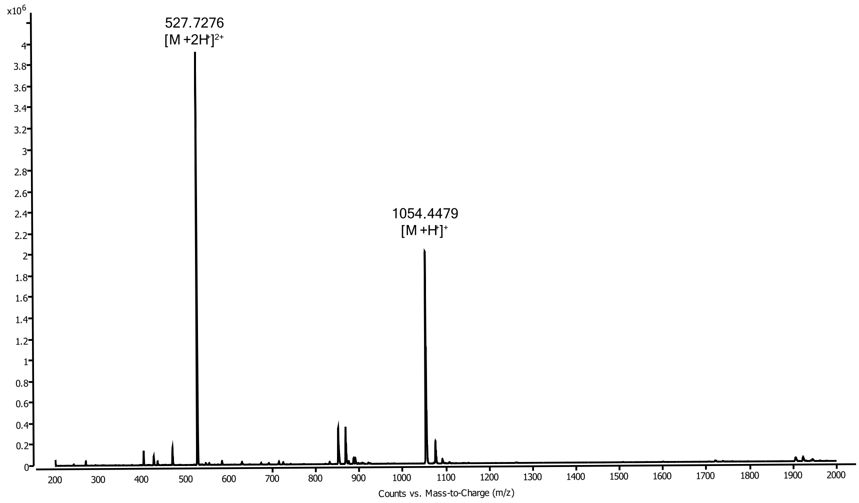

MS of **1e** modified Fmoc-VKQMK-CONH$_2$

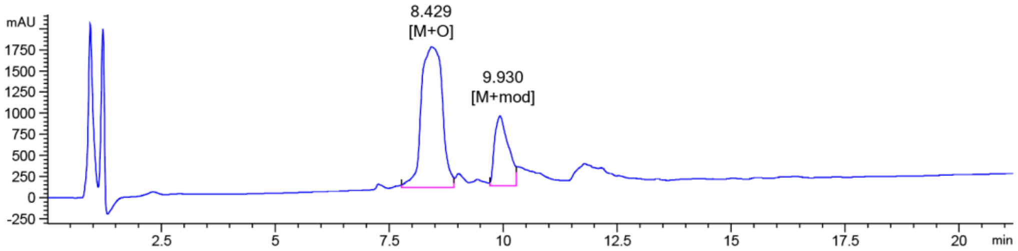

Peak #	RetTime [min]	Type	Width [min]	Area [mAU*s]	Height [mAU]	Area %
1	8.429	MM	0.5327	5.31393e4	1662.51257	75.6938
2	9.930	MM	0.3478	1.70637e4	817.75208	24.3062

HPLC trace of **1f** modified Fmoc-VKQMK-CONH$_2$

Fmoc-VKQM(mod)K-CONH$_2$: LCMS *m/z* 1045.4458 (calc. $[M+H^+]$ = 1045.4446), m/z 523.2246 (calc. $[(M+2H^+)/2]$ = 523.2259), Purity: > 95 % (HPLC analysis at 220 nm). Retention time in HPLC: 9.930 min for product.

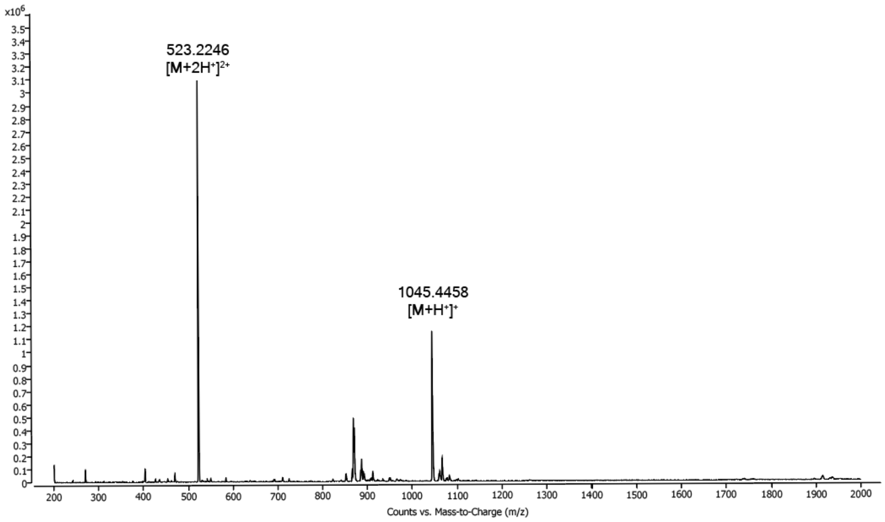

MS of **1f** modified Fmoc-VKQMK-CONH$_2$

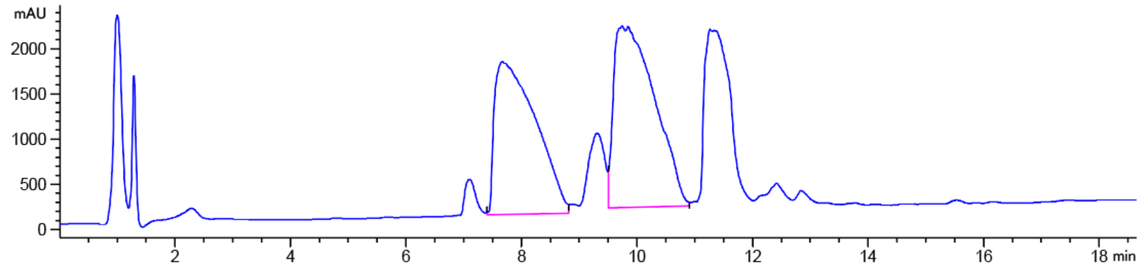

Peak #	RetTime [min]	Type	Width [min]	Area [mAU*s]	Height [mAU]	Area %
1	7.672	MM	0.8243	8.36932e4	1692.25208	45.8301
2	9.745	MM	0.8195	9.89230e4	2011.95703	54.1699

HPLC trace of **1g** modified Fmoc-VKQMK-CONH2

Fmoc-VKQM(mod)K-CONH2: LCMS *m/z* 1077.4519 (calc. $[M+H^+]$ = 1077.4508), m/z 539.2279 (calc. $[(M+2H^+)/2]$ = 539.2290), Purity: > 95 % (HPLC analysis at 220 nm). Retention time in HPLC: 9.745 min for product.

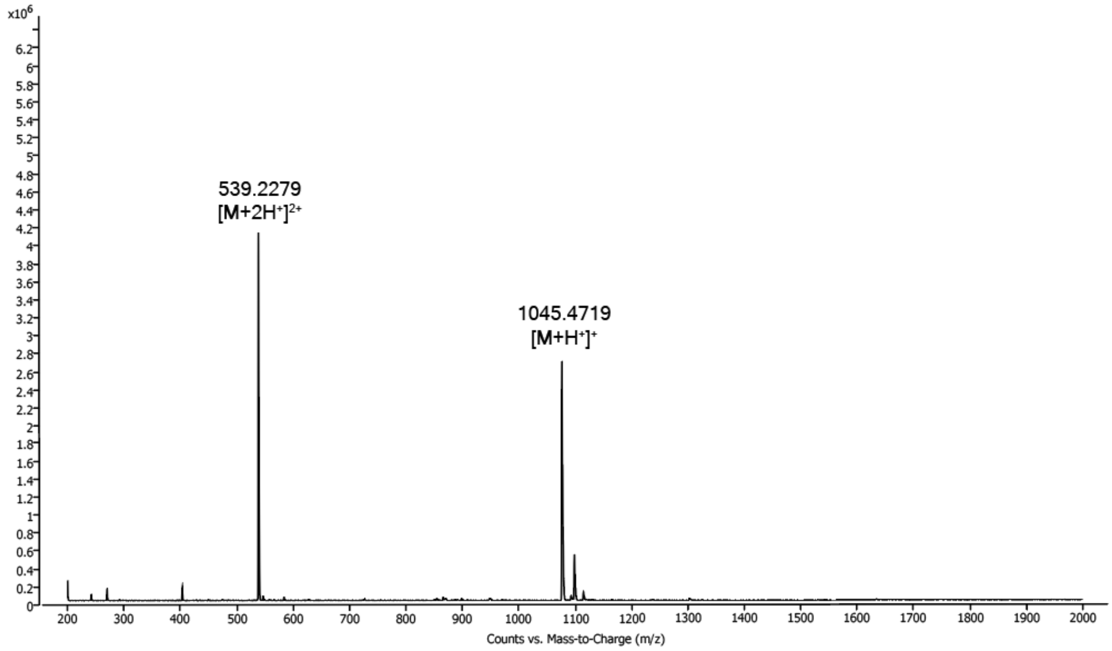

MS spectra of **1g** modified Fmoc-VKQMK-CONH2

Supplementary Fig. 16. Labeling of Myoglobin with various probes using CuNiP.

Labeling of Myoglobin with various equivalences of 1a using CuNiP

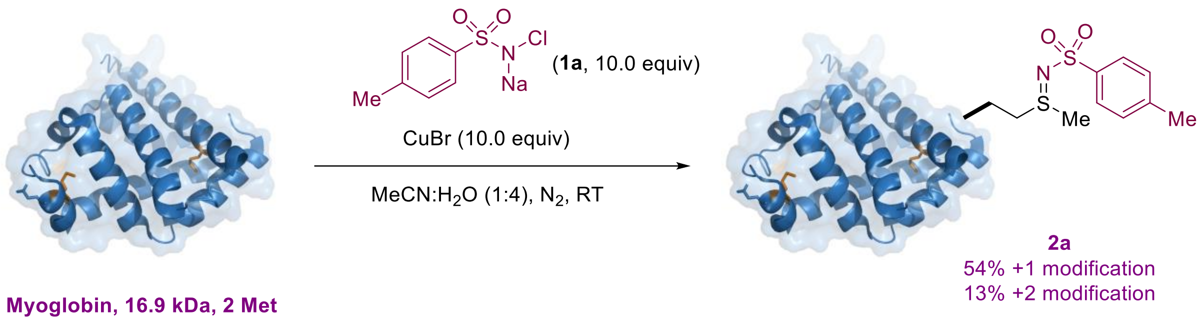

GLSDGEWQQVLNVWGKVEADIAGHGQEVLIRLFTGHPETLEKFDKFKHLKTEAEMKASEDLKKHGT
VVLTALGGILKKKGHHEAELKPLAQSHATKHKIPIKYLEFISDAIIHVLHSKHPGDFGADAQGA MTKAL
ELFRNDIAAKYKELGFQG

Myoglobin (2 mg, 0.12 µmol, 1.0 equiv) was dissolved in MeCN:H_2O (1:4, 800 µL) and CuBr (12 mM in MeCN, 100 µL, 1.2 µmol), **1a** (12 mM in H_2O, 100 µL, 1.2 µmol) were added sequentially. The reaction mixture was stirred at 25 °C for 2 h under nitrogen atmosphere followed by the addition of 10 µL of 0.5 N HCl. The crude reaction mixture was passed through Amicon Ultra 3 kDa spin-concentrator and washed with H_2O (7×0.5 mL) to remove the small molecule impurities. Labeled protein was lyophilized, redissolved in 0.1% formic acid in H_2O and analyzed using LC-MS. The conversion was found to be >95%. Intact mass analysis shows [O+1 modification] (54%) and +2 modification (13%) along with bis-sulfoxide as a side product.

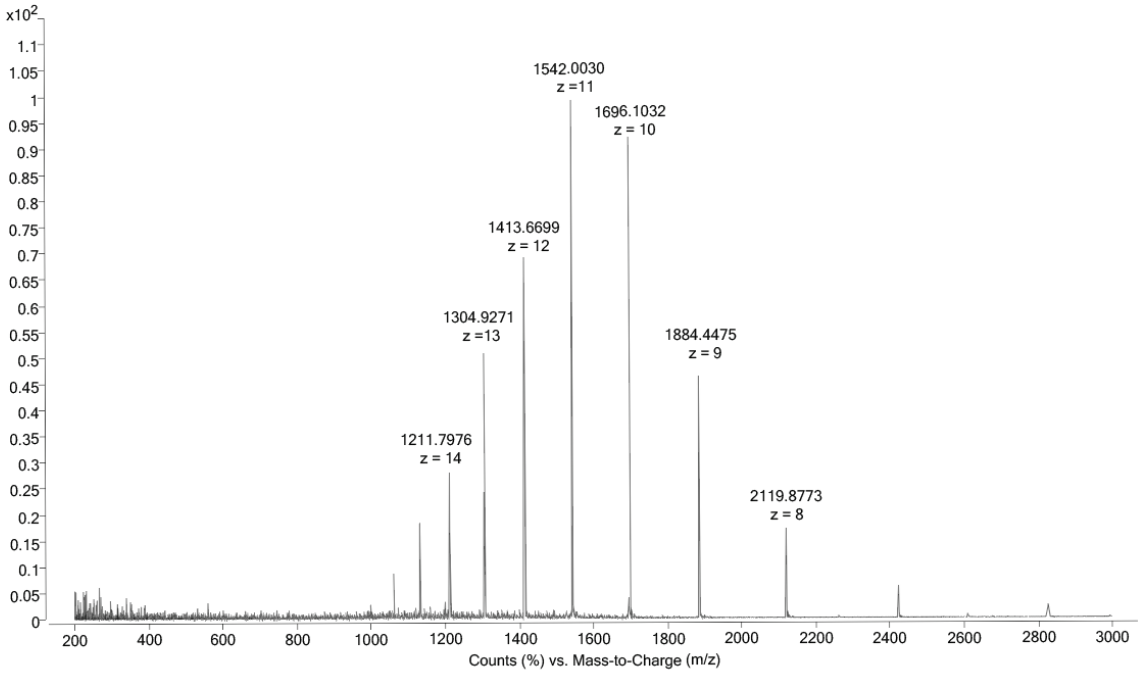

MS spectra of unmodified myoglobin

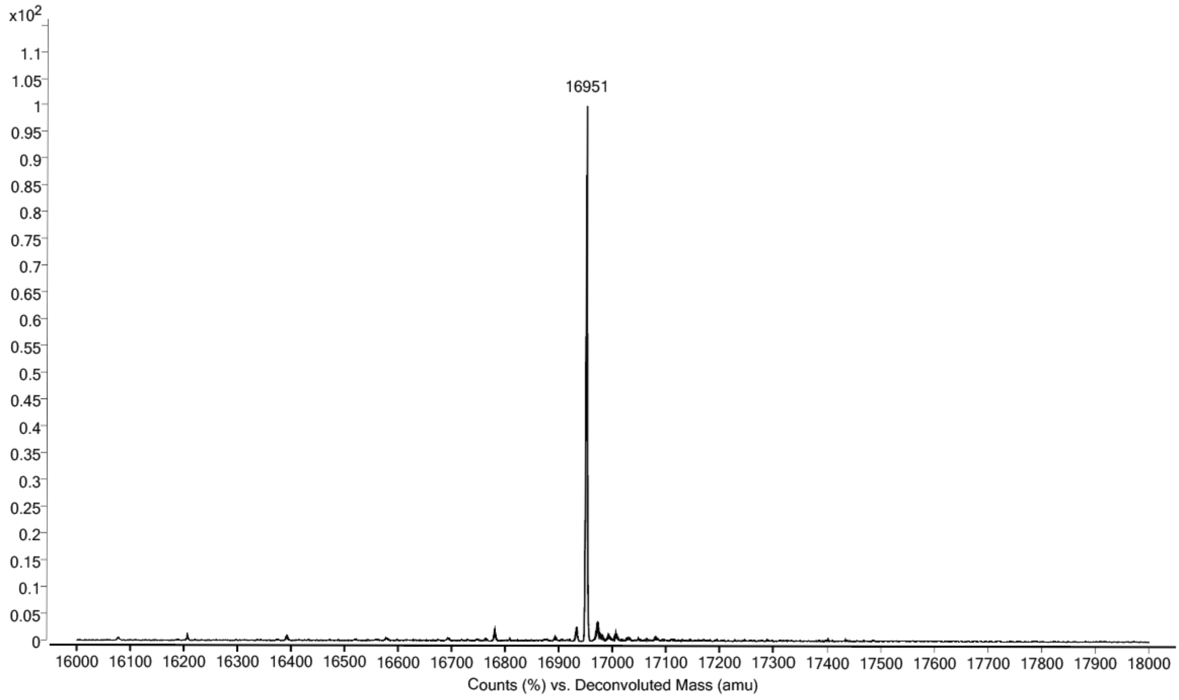

Deconvoluted MS spectra of unmodified myoglobin

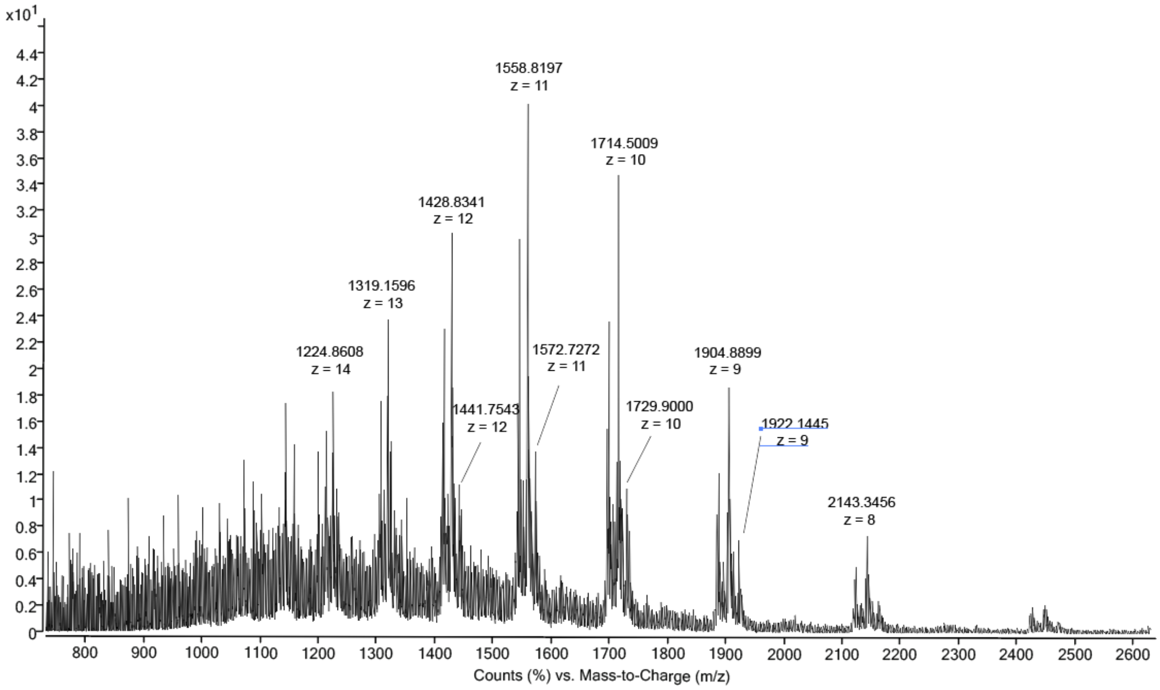

MS spectra of **1a** (10 equiv) modified myoglobin

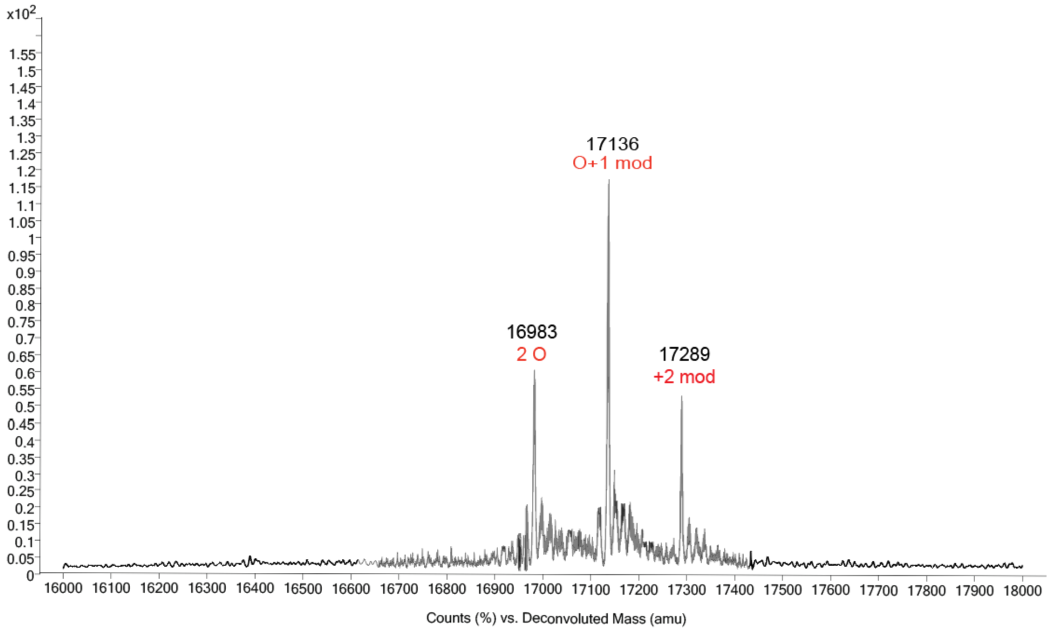

Deconvoluted MS spectra of **1a** (10 equiv) modified myoglobin

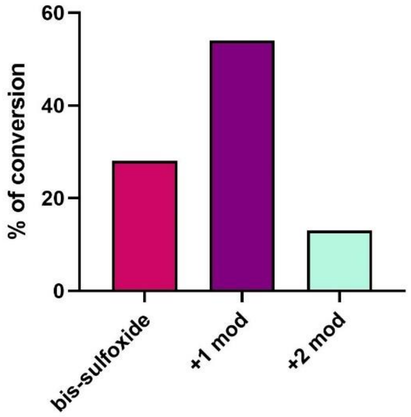

MS/MS Analysis of the Digested Myoglobin:

Biomolecule 15: HPGDFGADAQGAMTK

Biomol	Seq Loc	Rule	Pred Mods	RT	Height	Mass	Tgt Mass	Diff (ppm)
15	A(119-133)	Complete digest, Predicted modifications	Met-ChT- 13	7.504	1888565	1670.6864	1670.6817	2.77

ECC (with sample chromatogram)

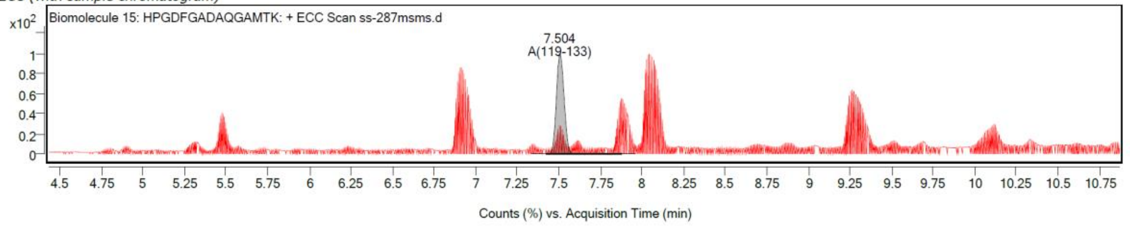

Mass Spectrum (with MFE spectrum, if available)

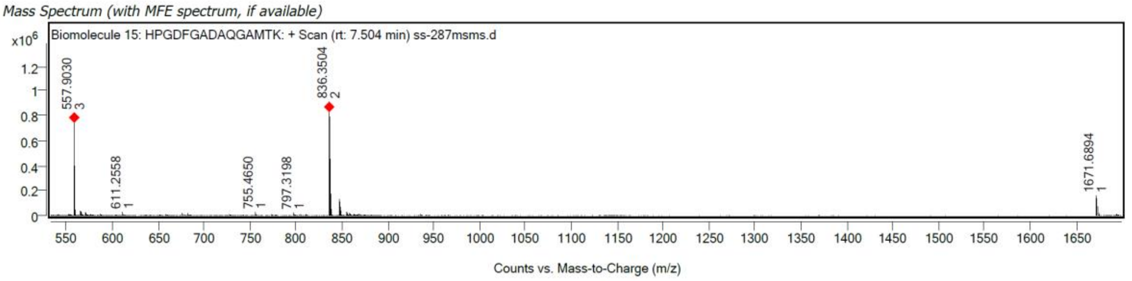

Identified Peptide Fragment: Sequence (AA119-AA133): HPGDFGADAQGA**M**TK

b^+	b^{2+}	AA			y^+	y^{2+}
138.066188	69.536732	1	H	15		
235.118952	118.063114	2	P	14	1534.630091	767.818684
292.140416	146.573846	3	G	13	1437.577328	719.292302
407.167359	204.087318	4	D	12	1380.555864	690.781570
554.235773	277.621525	5	F	11	1265.528921	633.268099
611.257237	306.132257	6	G	10	1118.460507	559.733892
682.294351	341.650813	7	A	9	1061.439043	531.223160
797.321294	399.164285	8	D	8	990.401929	495.704603
868.358407	434.682842	9	A	7	875.374986	438.191131
996.416985	498.712131	10	Q	6	804.337872	402.672574
1053.438449	527.222863	11	G	5	676.279295	338.643286
1124.475562	562.741419	12	A	4	619.257831	310.132554
1424.535797	712.771537	13	**M+mod**	3	548.220717	274.613997
1525.583476	763.295376	14	T	2	248.160483	124.583880
		15	K	1	147.112804	74.060040

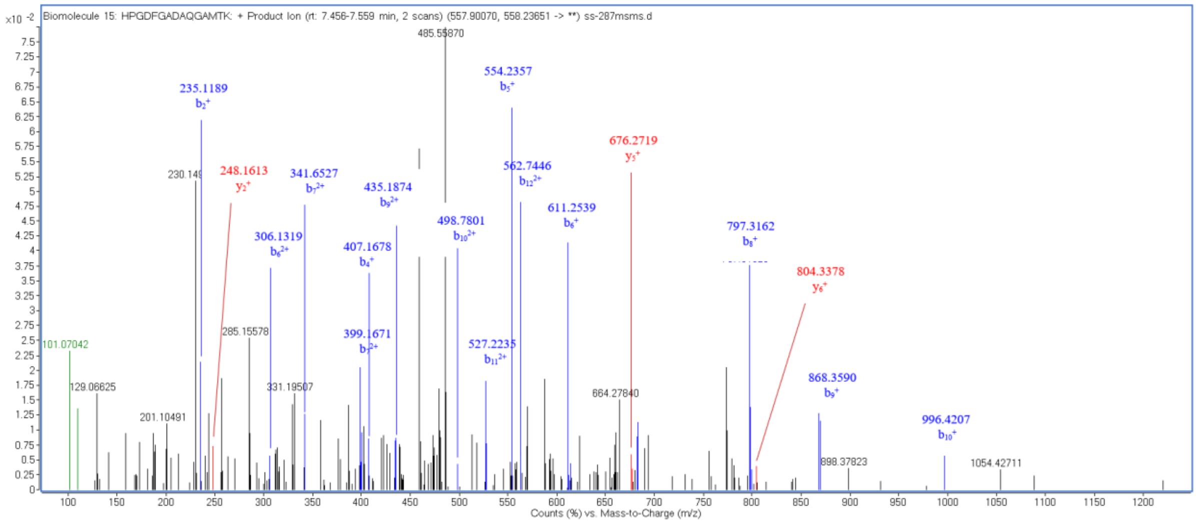

Labeling of Myoglobin with 20.0 equiv CuBr/Chloramine-T (1a).

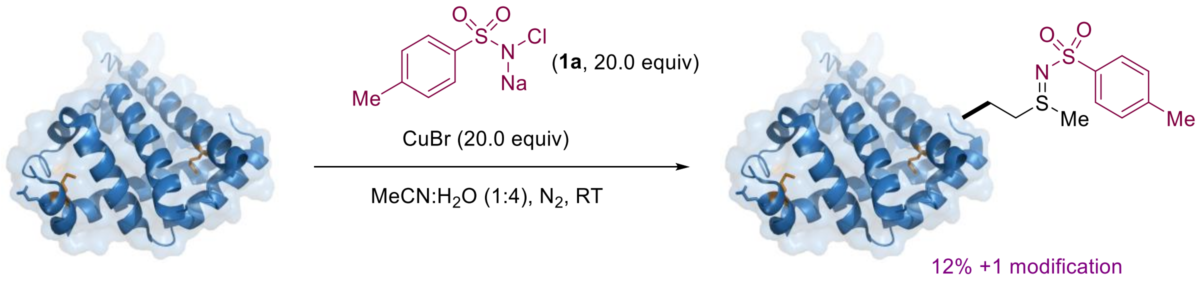

Myoglobin (2 mg, 0.12 µmol, 1.0 equiv) was dissolved in MeCN:H_2O (1:4, 800 µL) and CuBr (24 mM in MeCN, 100 µL, 2.4 µmol), **1a** (24 mM in H_2O, 100 µL, 2.4 µmol) were added sequentially. The reaction mixture was stirred at 25 °C for 2 h under nitrogen atmosphere

followed by the addition of 10 μL of 0.5 N HCl. The crude reaction mixture was passed through Amicon Ultra 3 kDa spin-concentrator and washed with H_2O (7×0.5 mL) to remove the small molecule impurities. Labeled protein was lyophilized, redissolved in 0.1% formic acid in H_2O and analyzed using LC-MS. The conversion was found to be >95%. Intact mass analysis shows [O+1 modification] (12%) and along with bis-sulfoxide as a side product.

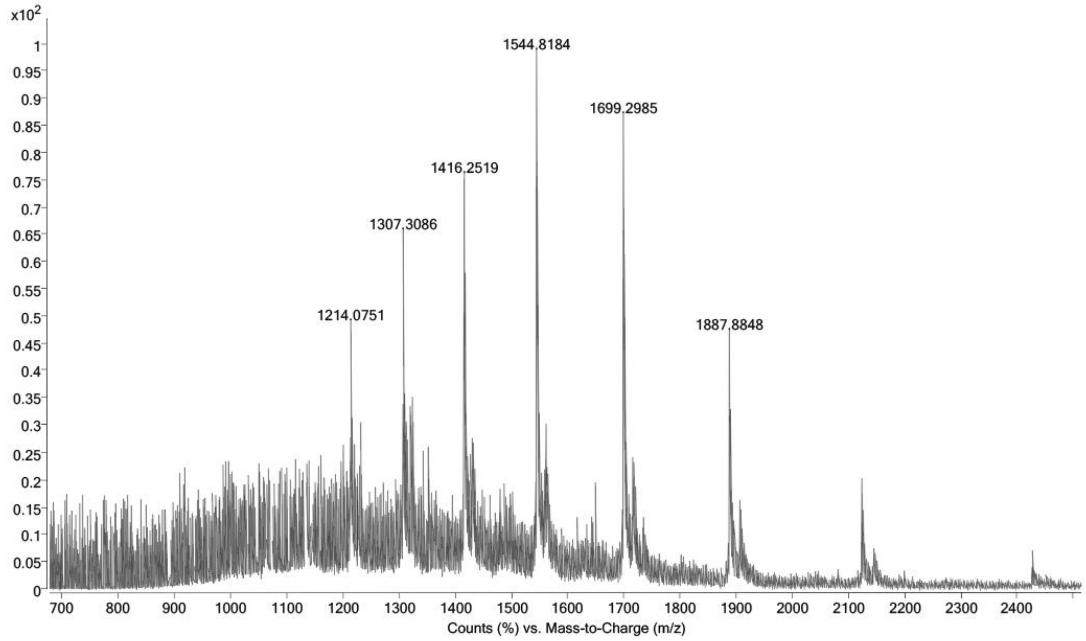

MS spectra of **1a** (20 equiv.) modified myoglobin

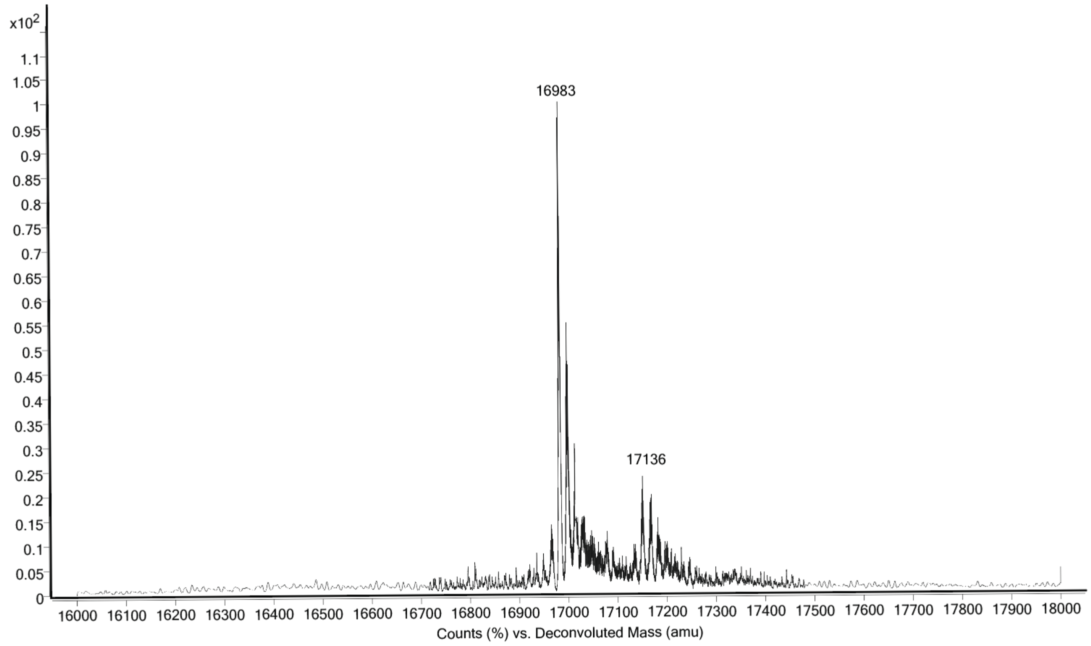

Deconvoluted MS spectra of **1a** (20 equiv.) modified myoglobin

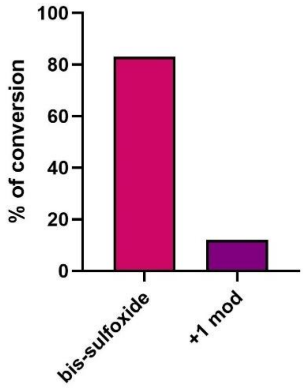

Labeling of Myoglobin with 30.0 equiv CuBr/Chloramine-T (1a).

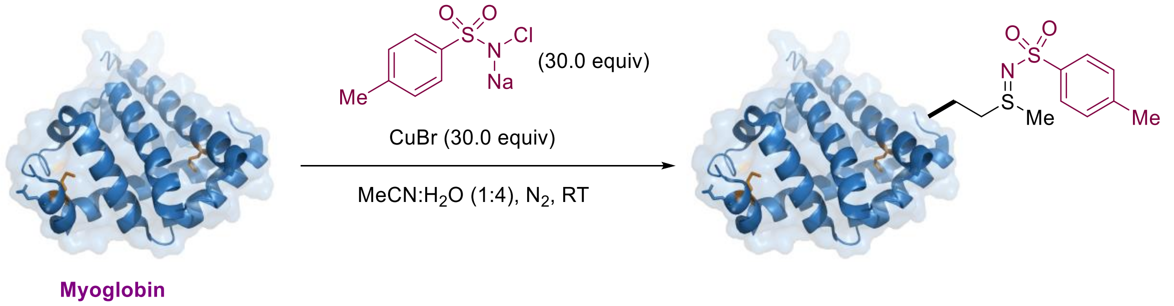

Myoglobin (2 mg, 0.12 µmol, 1.0 equiv) was dissolved in MeCN:H_2O (1:4, 800 µL) and CuBr (36 mM in MeCN, 100 µL, 1.2 µmol), **1a** (36 mM in H_2O, 100 µL, 1.2 µmol) were added sequentially. The reaction mixture was stirred at 25 °C for 2 h under nitrogen atmosphere followed by the addition of 10 µL of 0.5 N HCl. The crude reaction mixture was passed through Amicon Ultra 3 kDa spin-concentrator and washed with H_2O (7×0.5 mL) to remove the small molecule impurities. Labeled protein was lyophilized, redissolved in 0.1% formic acid in H_2O and analyzed using LC-MS. The conversion was found to be >95%. Intact mass analysis shows bis-sulfoxide as a sole product.

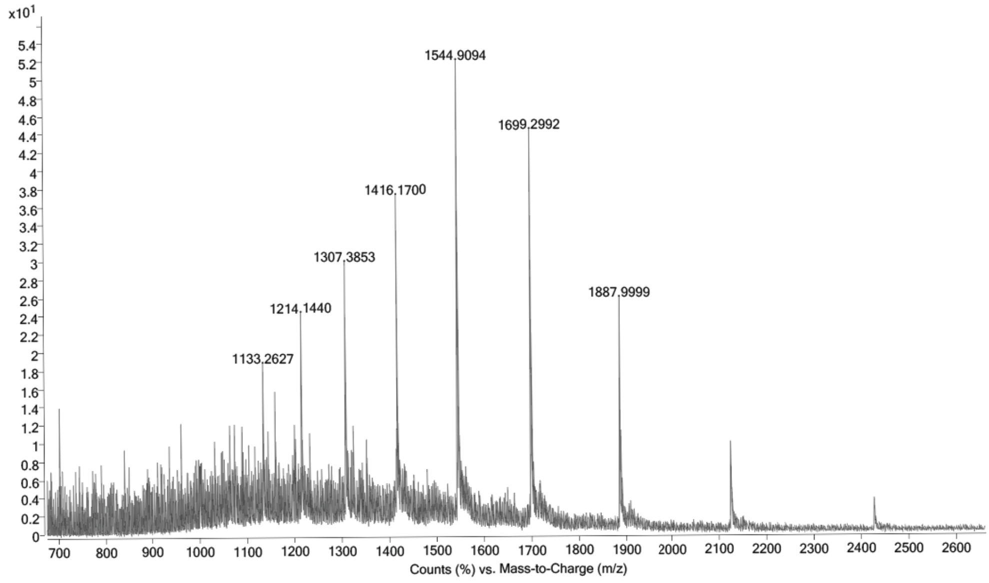

MS spectra of **1a** (30 equiv) modified myoglobin

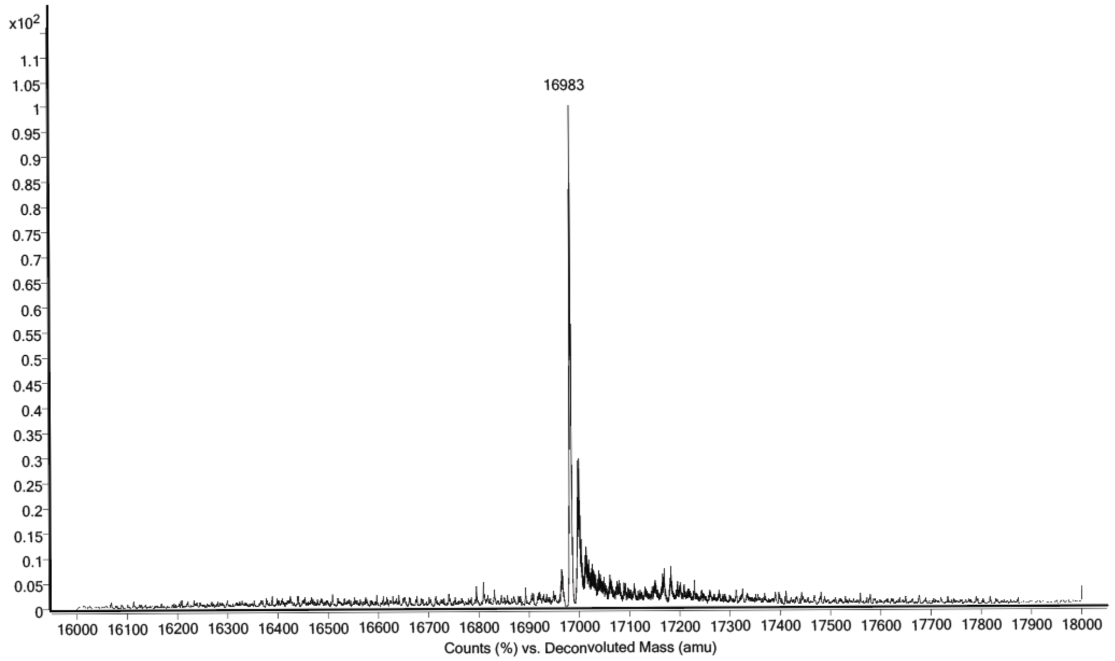

Deconvoluted MS spectra **1a** (30 equiv) modified myoglobin

Labeling of Myoglobin with 1b using CuNiP

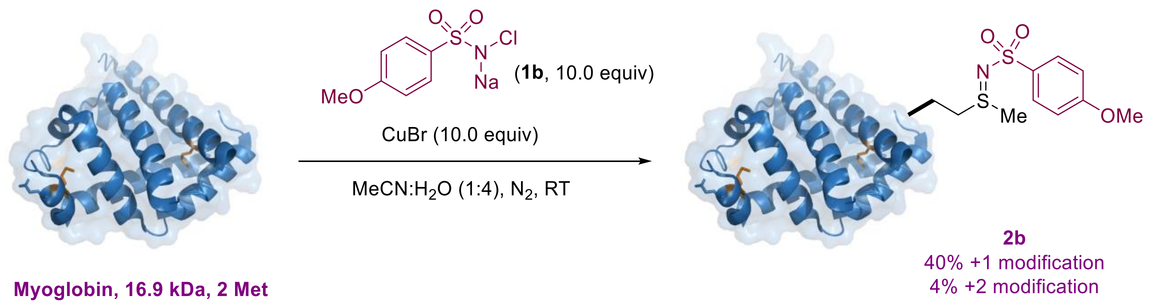

Myoglobin (2 mg, 0.12 µmol, 1.0 equiv) was dissolved in MeCN:H_2O (1:4, 800 µL) and CuBr (12 mM in MeCN, 100 µL, 1.2 µmol), **1b** (12 mM in H_2O, 100 µL, 1.2 µmol) were added sequentially. The reaction mixture was stirred at 25 °C for 2 h under nitrogen atmosphere followed by the addition of 10 µL of 0.5 N HCl. The crude reaction mixture was passed through Amicon Ultra 3 kDa spin-concentrator and washed with H_2O (7×0.5 mL) to remove the small molecule impurities. Labeled protein was lyophilized, redissolved in 0.1% formic acid in H_2O and analyzed using LC-MS. The conversion was found to be >95%. Intact mass analysis shows [O+1 mod] as 40%, +2 modification (4%) along with bis-sulfoxide as a side product.

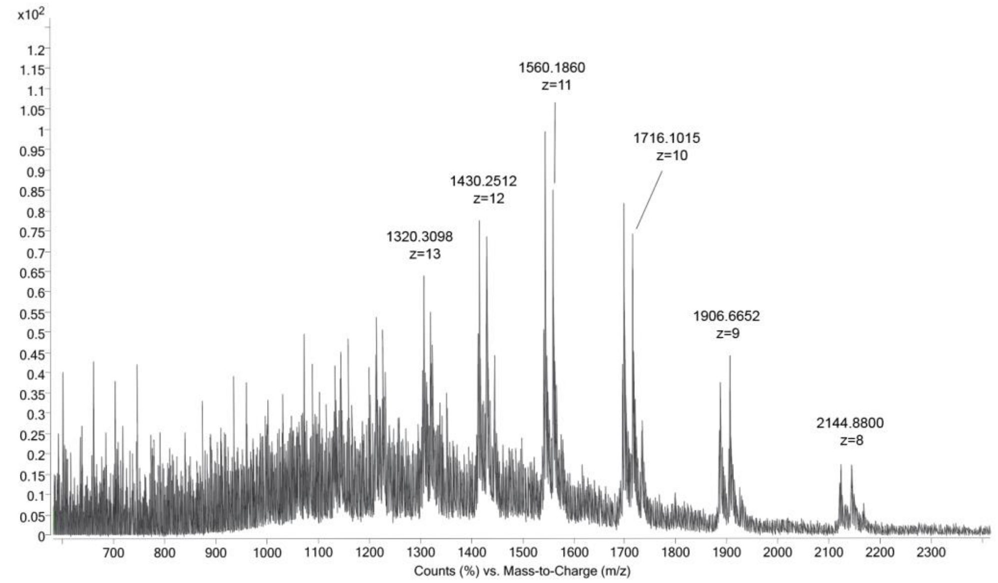

MS spectra of **1b** modified myoglobin

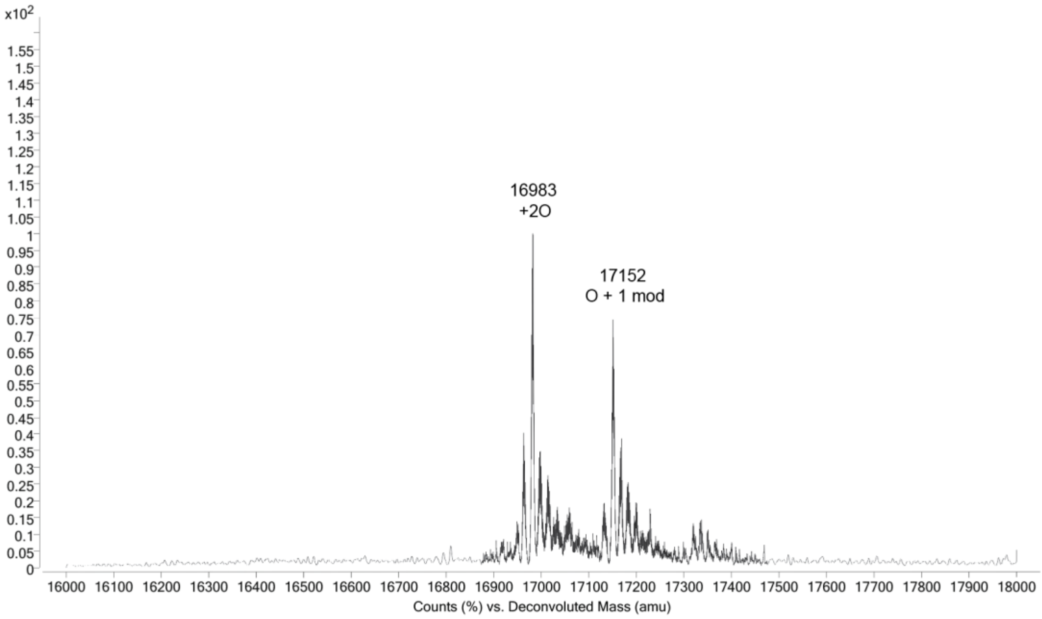

Deconvoluted MS spectra of **1b** modified myoglobin

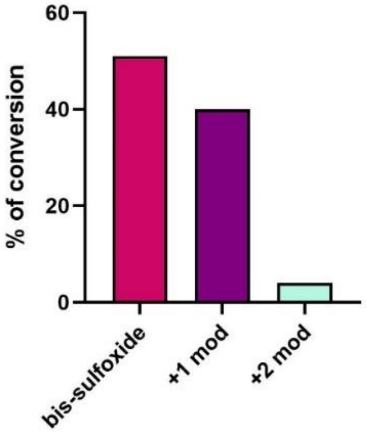

Labeling of Myoglobin with 1c using CuNiP

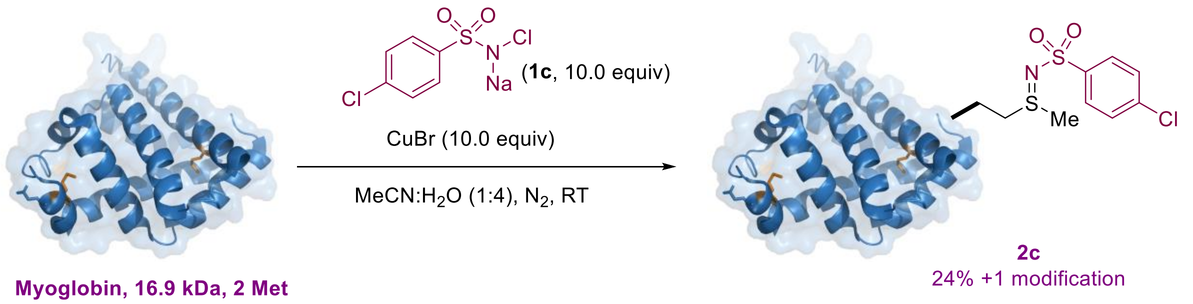

Myoglobin (2 mg, 0.12 µmol, 1.0 equiv) was dissolved in MeCN:H_2O (1:4, 800 µL) and CuBr (12 mM in MeCN, 100 µL, 1.2 µmol), **1c** (12 mM in H_2O, 100 µL, 1.2 µmol) were added sequentially. The reaction mixture was stirred at 25 °C for 2 h under nitrogen atmosphere followed by the addition of 10 µL of 0.5 N HCl. The crude reaction mixture was passed through Amicon Ultra 3 kDa spin-concentrator and washed with H_2O (7×0.5 mL) to remove the small molecule impurities. Labeled protein was lyophilized, redissolved in 0.1% formic acid in H_2O and analyzed using LC-MS. The conversion was found to be >95%. Intact mass analysis shows 24% [O+1 mod] along with bis-sulfoxide as a side product.

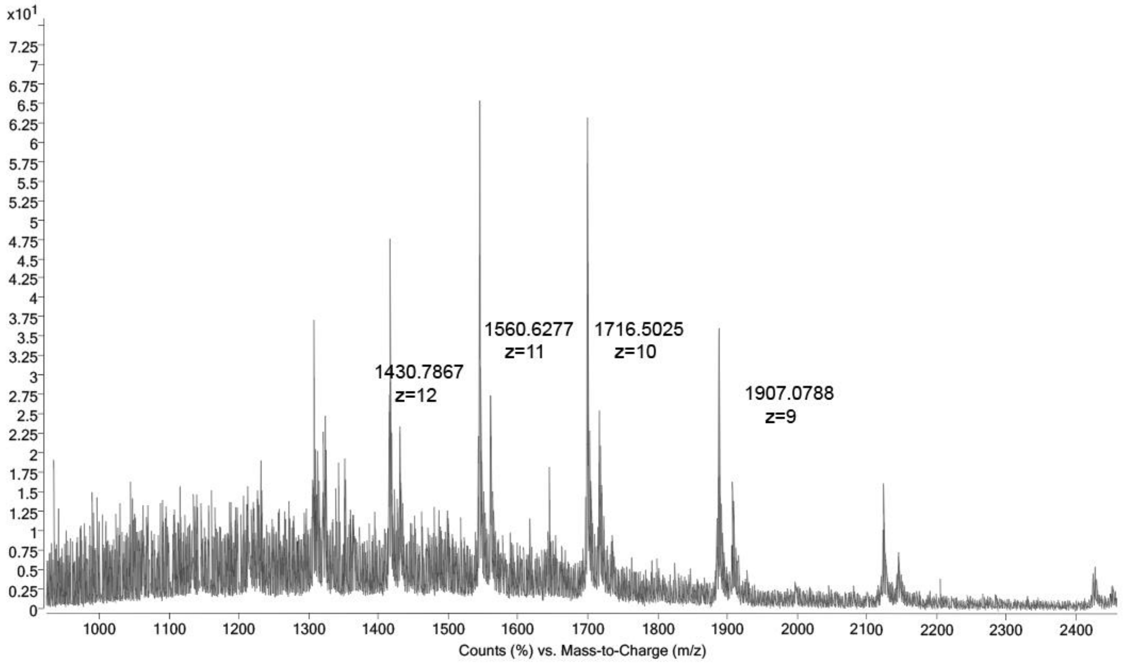

MS spectra of **1c** modified myoglobin

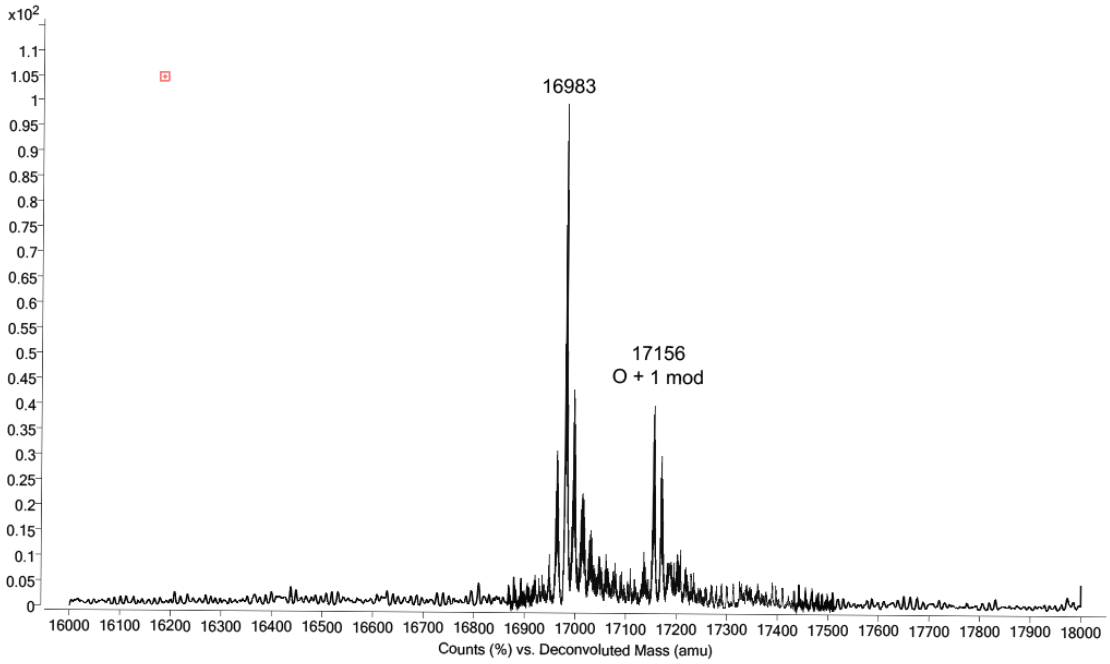

Deconvoluted MS spectra of **1c** modified myoglobin

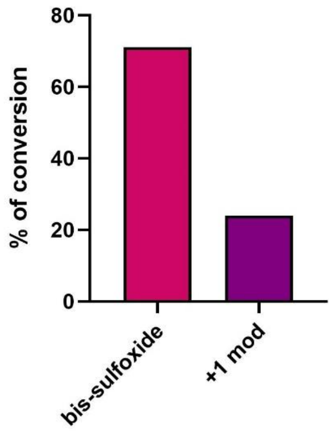

Labeling of Myoglobin with 1d using CuNiP

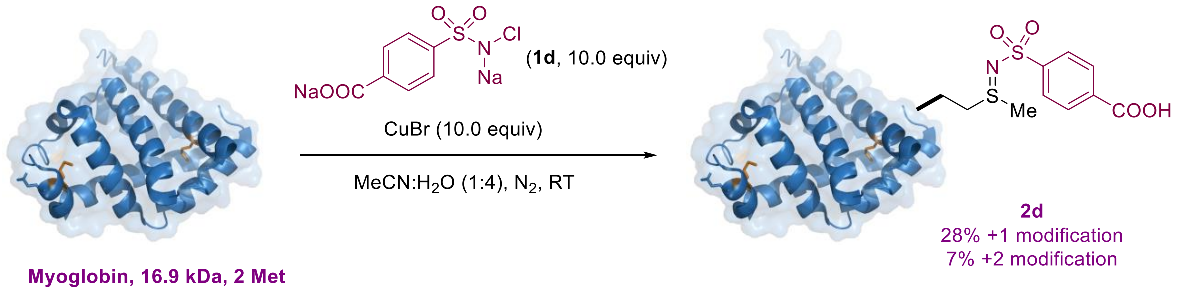

Myoglobin (2 mg, 0.12 µmol, 1.0 equiv) was dissolved in MeCN:H_2O (1:4, 800 µL) and CuBr (12 mM in MeCN, 100 µL, 1.2 µmol), **1d** (12 mM in H_2O, 100 µL, 1.2 µmol) were added sequentially. The reaction mixture was stirred at 25 °C for 2 h under nitrogen atmosphere

followed by the addition of 10 μL of 0.5 N HCl. The crude reaction mixture was passed through Amicon Ultra 3 kDa spin-concentrator and washed with H_2O (7×0.5 mL) to remove the small molecule impurities. Labeled protein was lyophilized, redissolved in 0.1% formic acid in H_2O and analyzed using LC-MS. The conversion was found to be >95%. Intact mass analysis shows [O+1 mod] as 28%, 7% +2 modification along with bis-sulfoxide as a side product.

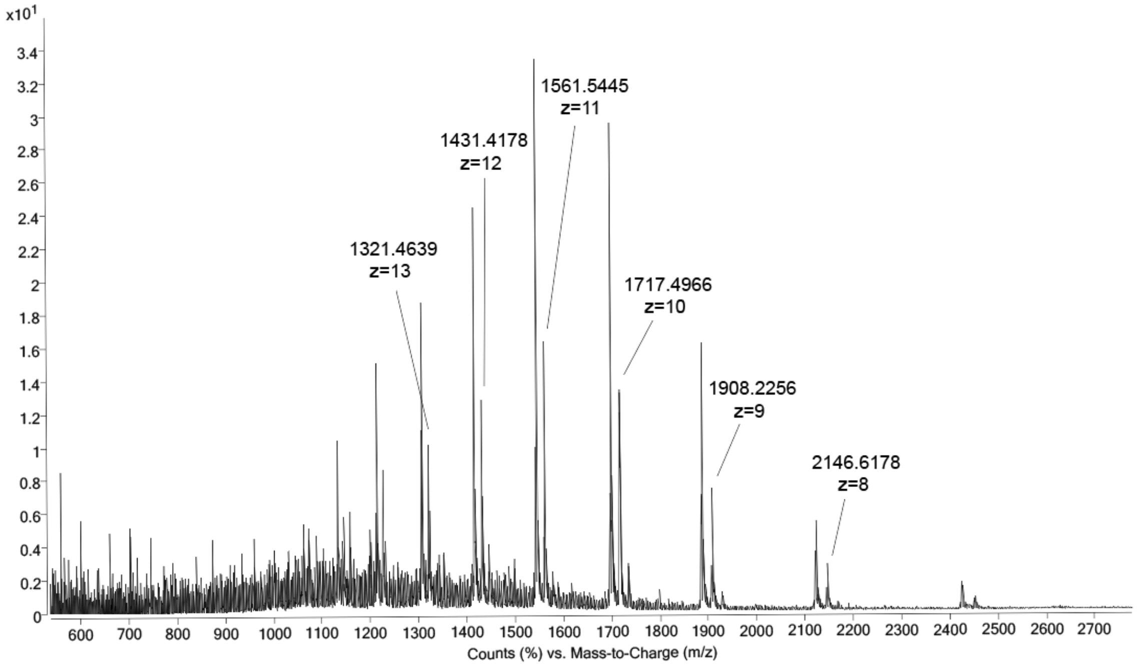

MS spectra of **1d** modified myoglobin

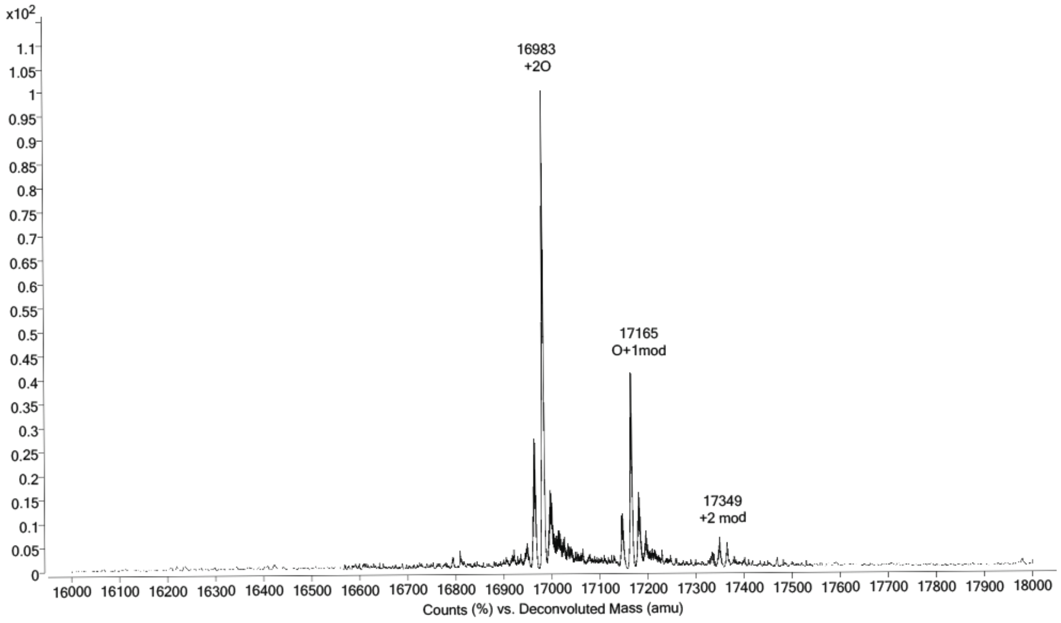

Deconvoluted MS spectra of **1d** modified myoglobin

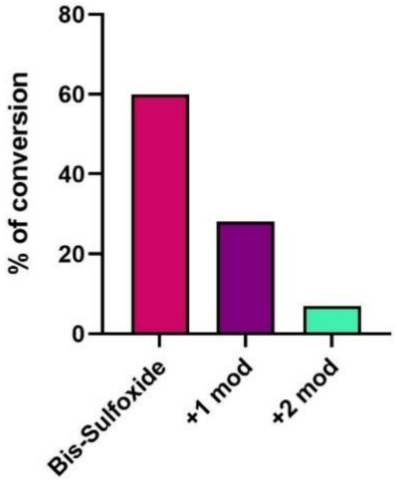

Labeling of Myoglobin with 1e using CuNiP

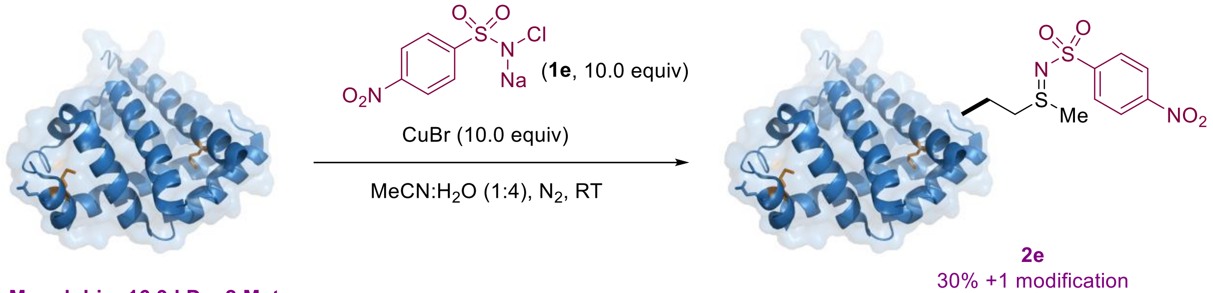

Myoglobin (2 mg, 0.12 µmol, 1.0 equiv) was dissolved in MeCN:H_2O (1:4, 800 µL) and CuBr (12 mM in MeCN, 100 µL, 1.2 µmol), **1e** (12 mM in H_2O, 100 µL, 1.2 µmol) were added sequentially. The reaction mixture was stirred at 25 °C for 2 h under nitrogen atmosphere followed by the addition of 10 µL of 0.5 N HCl. The crude reaction mixture was passed through Amicon Ultra 3 kDa spin-concentrator and washed with H_2O (7×0.5 mL) to remove the small molecule impurities. Labeled protein was lyophilized, redissolved in 0.1% formic acid in H_2O and analyzed using LC-MS. The conversion was found to be >95%. Intact mass analysis shows [O+1 mod] as 30% along with bis-sulfoxide as a side product.

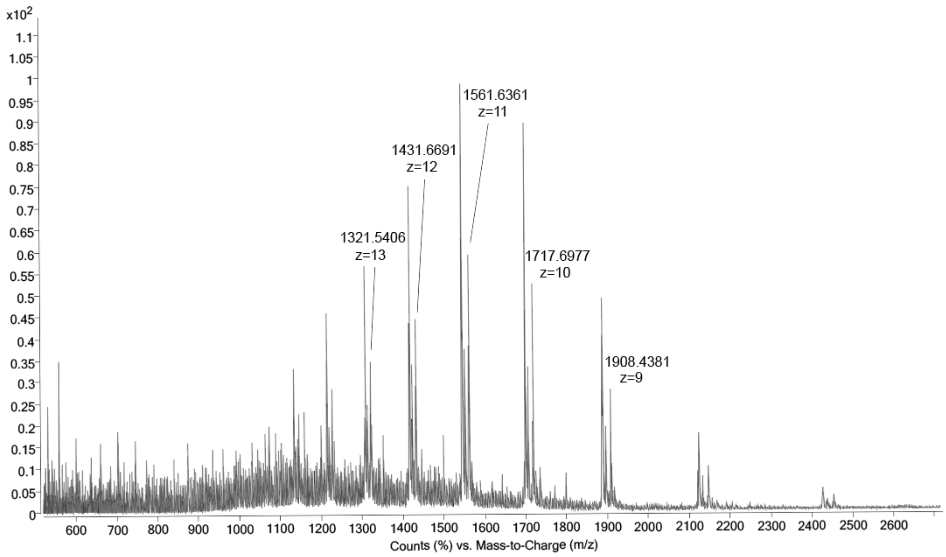

MS spectra of **1e** modified myoglobin

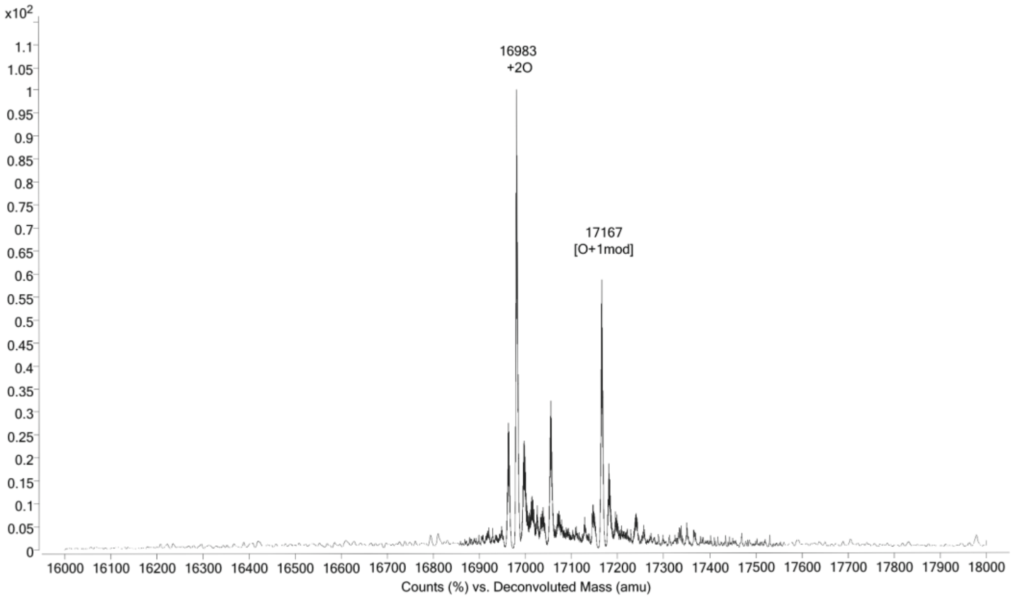

Deconvoluted MS spectra of **1e** modified myoglobin

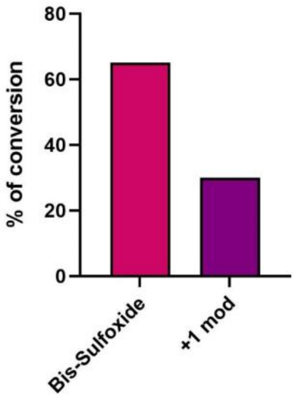

Labeling of Myoglobin with 1f using CuNiP:

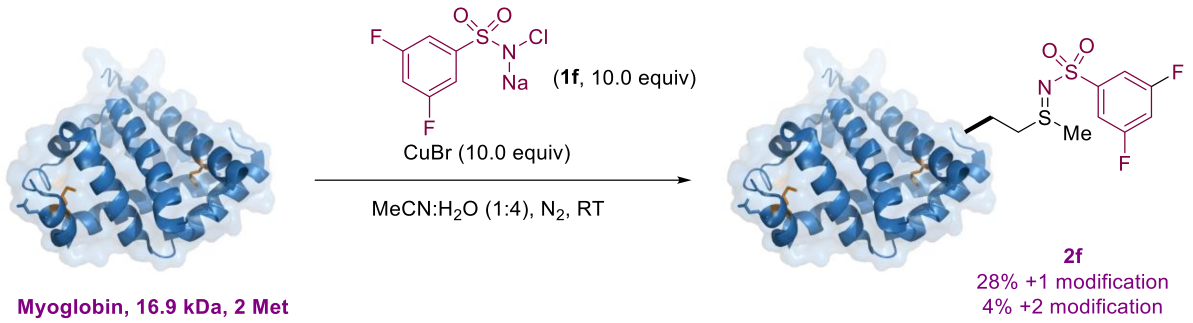

Myoglobin (2 mg, 0.12 μmol, 1.0 equiv) was dissolved in MeCN:H_2O (1:4, 800 μL) and CuBr (12 mM in MeCN, 100 μL, 1.2 μmol), **1f** (12 mM in H_2O, 100 μL, 1.2 μmol) were added sequentially. The reaction mixture was stirred at 25 °C for 2 h under nitrogen atmosphere followed by the addition of 10 μL of 0.5 N HCl. The crude reaction mixture was passed through Amicon Ultra 3 kDa spin-concentrator and washed with H_2O (7×0.5 mL) to remove the small molecule impurities. Labeled protein was lyophilized, redissolved in 0.1% formic acid in H_2O and analyzed using LC-MS. The conversion was found to be >95%. Intact mass analysis shows [O+1 mod] as 28%, +2 mod as 4% along with bis-sulfoxide as a side product.

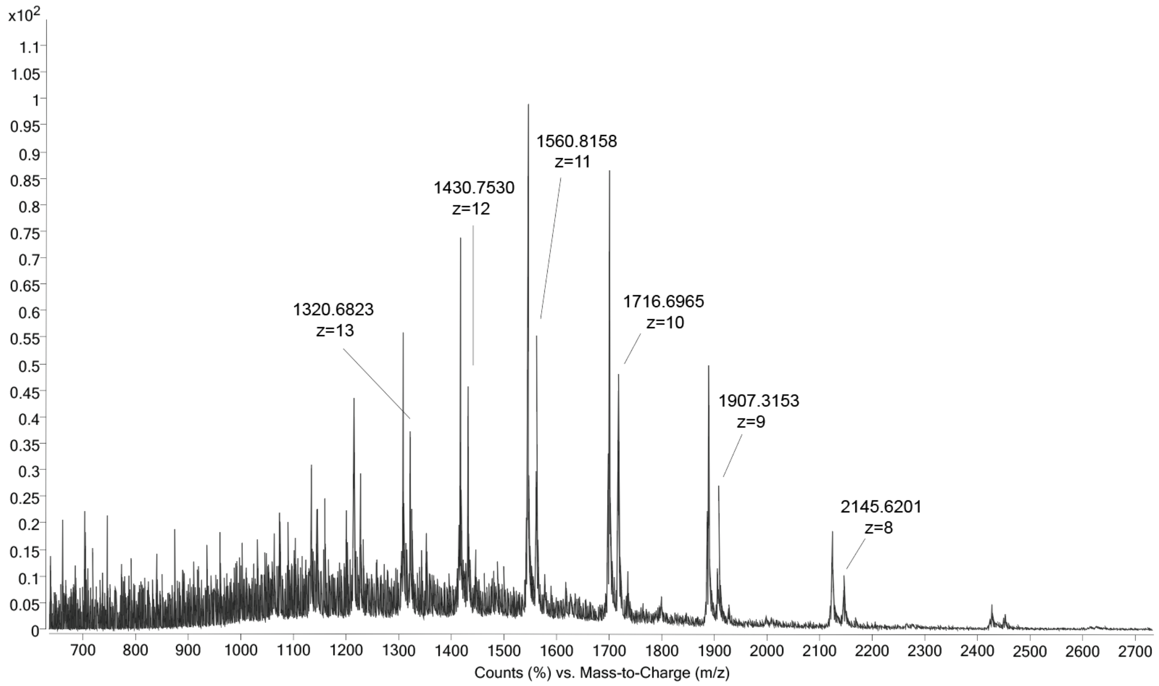

MS spectra of **1f** modified myoglobin

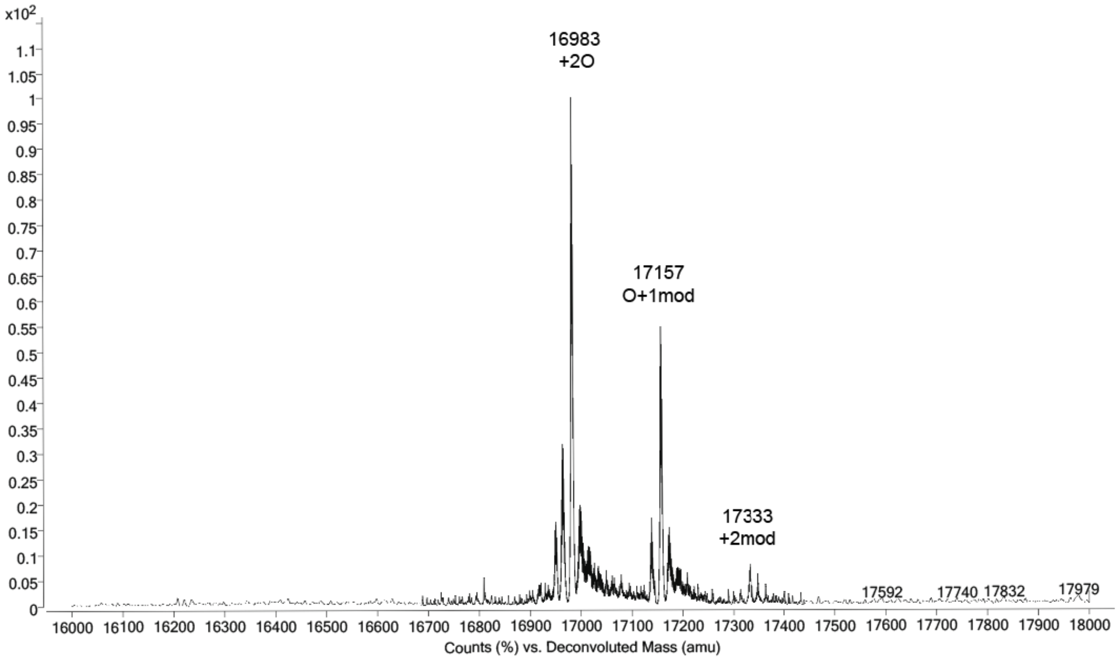

Deconvoluted MS spectra of **1f** modified myoglobin

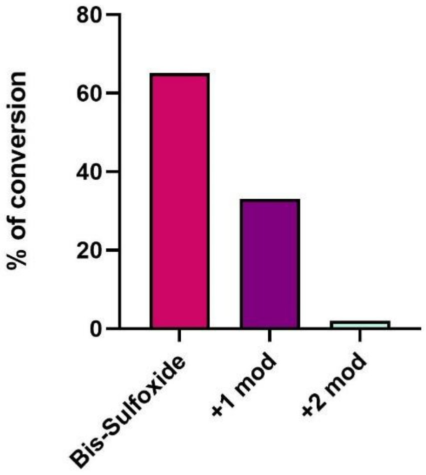

Labeling of Myoglobin with 1g using CuNiP

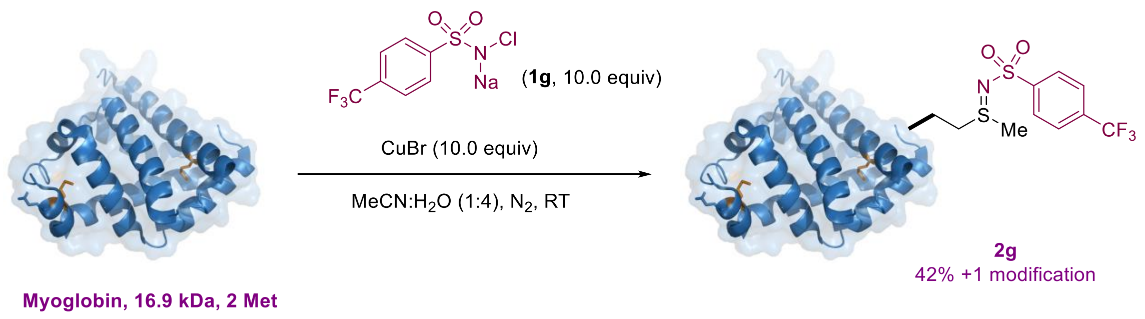

Myoglobin (2 mg, 0.12 μmol, 1.0 equiv) was dissolved in MeCN:H_2O (1:4, 800 μL) and CuBr (12 mM in MeCN, 100 μL, 1.2 μmol), **1g** (12 mM in H_2O, 100 μL, 1.2 μmol) were added sequentially. The reaction mixture was stirred at 25 °C for 2 h under nitrogen atmosphere

followed by the addition of 10 µL of 0.5 N HCl. The crude reaction mixture was passed through Amicon Ultra 3 kDa spin-concentrator and washed with H_2O (7×0.5 mL) to remove the small molecule impurities. Labeled protein was lyophilized, redissolved in 0.1% formic acid in H_2O and analyzed using LC-MS. The conversion was found to be >95%. Intact mass analysis shows [O+1 mod] as 42%, along with bis-sulfoxide as a side product.

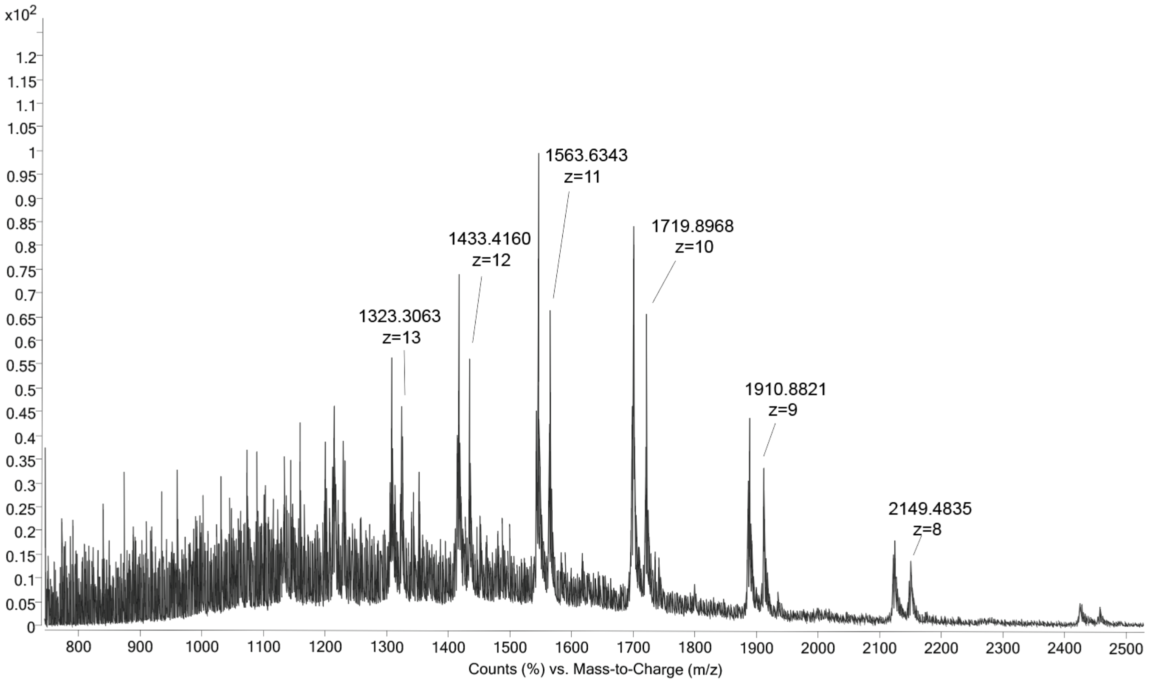

MS spectra of **1g** modified myoglobin

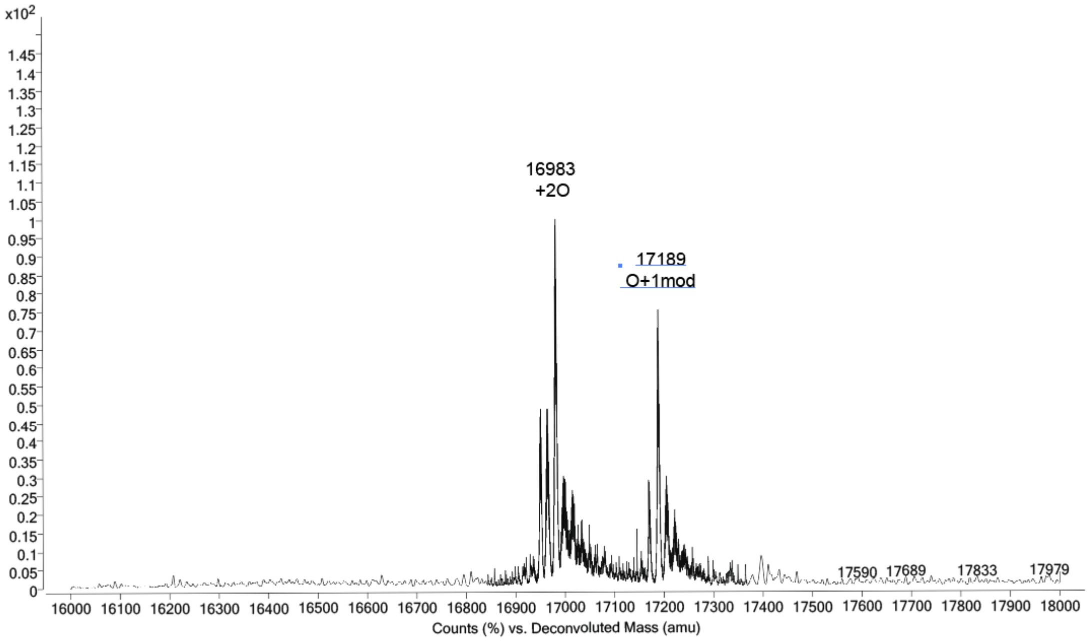

Deconvoluted MS spectra of **1g** modified myoglobin

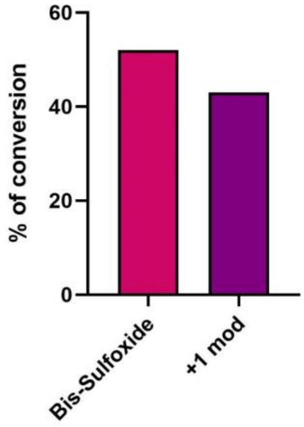

Supplementary Fig. 17. DFT evaluation of electronic effects on CuNiP reaction

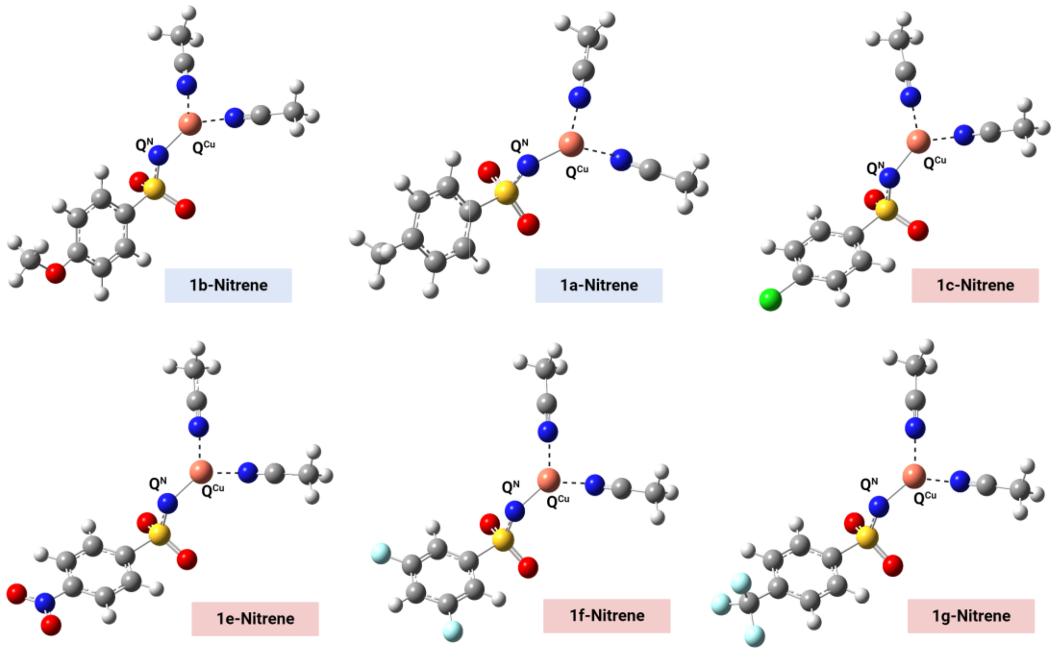

Electronic effect of nitrene probes

Nitrene probes	Mulliken Q_N ↑	Mulliken Q_{Cu} ↓	NBO Q_N ↑	NBO Q_{Cu} ↓
1b	-0.242	0.026	-1.119	1.011
1a	-0.230	0.103	-1.116	1.010
1c	-0.168	0.200	-1.114	1.016
1e	-0.160	0.201	-1.114	1.020
1f	-0.154	0.196	-1.114	1.024
1g	-0.131	0.194	-1.115	1.022

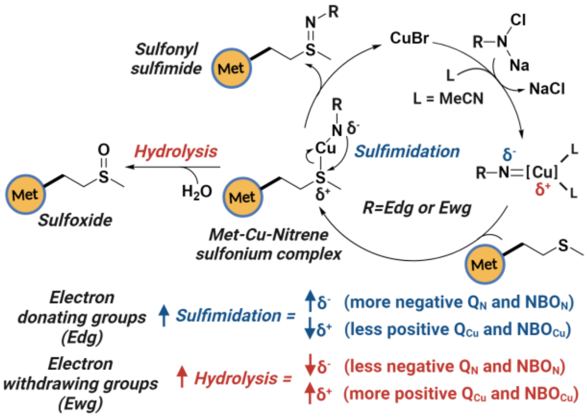

Mulliken population and Natural Bond Orbital (NBO) analysis revealed that the electron density on the nitrogen (Q^N) of the copper-nitrene complex undergoes an increase in the presence of electron-donating groups. This augmented electron density enhances the efficiency of capturing the methionine-Cu-nitrene sulfonium complex, leading to the formation of the sulfonyl sulfimide product. Conversely, when electron-withdrawing groups are employed, a decrease in electron density on the nitrogen (Q^N) of the copper-nitrene complex is observed.

Additionally, the electron density on Cu in the nitrene complex (Q^{Cu}) decreases with the presence of electron-withdrawing groups. This suggests that the initial reaction with methionine to form the methionine-Cu-nitrene sulfonium complex occurs more rapidly with electron-withdrawing probes. However, the subsequent trapping by nitrene nitrogen becomes less favorable, resulting in an increased formation of sulfoxide compared to electron-releasing probes. Calculations were done using the **general computational details** above.

To evaluate if the sulfur atom of methionine becomes electrophilic upon reaction with CuNiP probe, we optimized the geometries and calculated the electrostatic potential (ESP) maps of methionine and Met-Cu-Nitrene sulfonium complex. Analysis of the ESP map clearly shows the partial electrophilic nature of the sulfur atom in Met-Cu-Nitrene sulfonium complex (-CH_3 analog, **1a**) as compared to unreacted methionine.

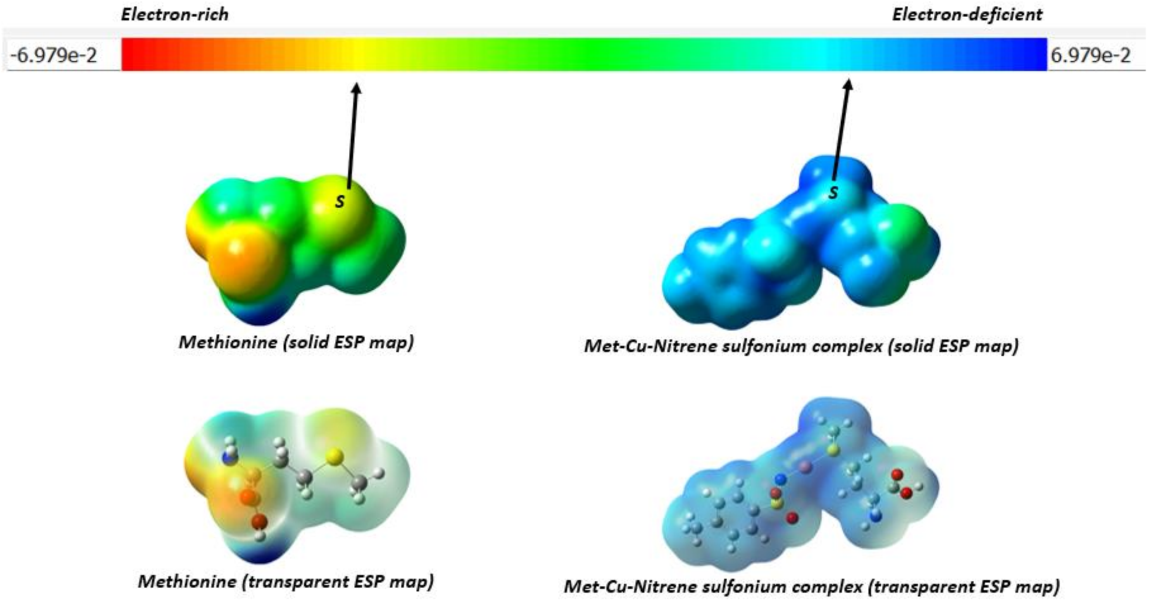

Supplementary Fig. 18. Labeling of methionine in Aprotinin by CuNiP (PDB: 1OA5).

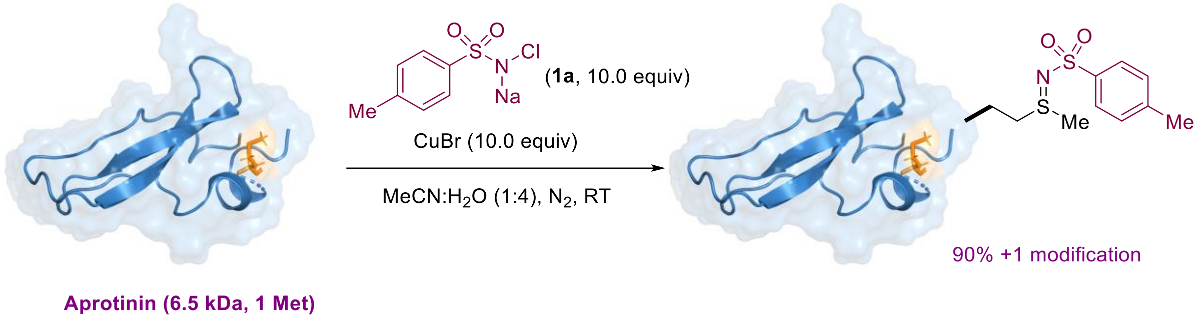

RPDFCLEPPYTGPCKARIIRYFYNAKAGLCQTFVYGGCRAKRNNFKSAEDCMRTCGGA

Aprotinin (0.78 mg, 0.12 µmol, 1.0 equiv) was dissolved in MeCN:H_2O (1:4, 800 µL) and CuBr (12 mM in MeCN, 100 µL, 1.2 µmol), **1a** (12 mM in H_2O, 100 µL, 1.2 µmol) were added sequentially. The reaction mixture was incubated at 25 °C for 2 h under nitrogen atmosphere followed by the addition of 10 µL of 0.5 N HCl. The crude reaction mixture was passed through Amicon Ultra 3 kDa spin-concentrator and washed with H_2O (7×0.5 mL) to remove the small molecule impurities This labeled protein was lyophilized, redissolved in 0.1% formic acid in H_2O and analyzed using LC-MS. The conversion was found to be >95%. Intact mass analysis shows 90% +1 modification.

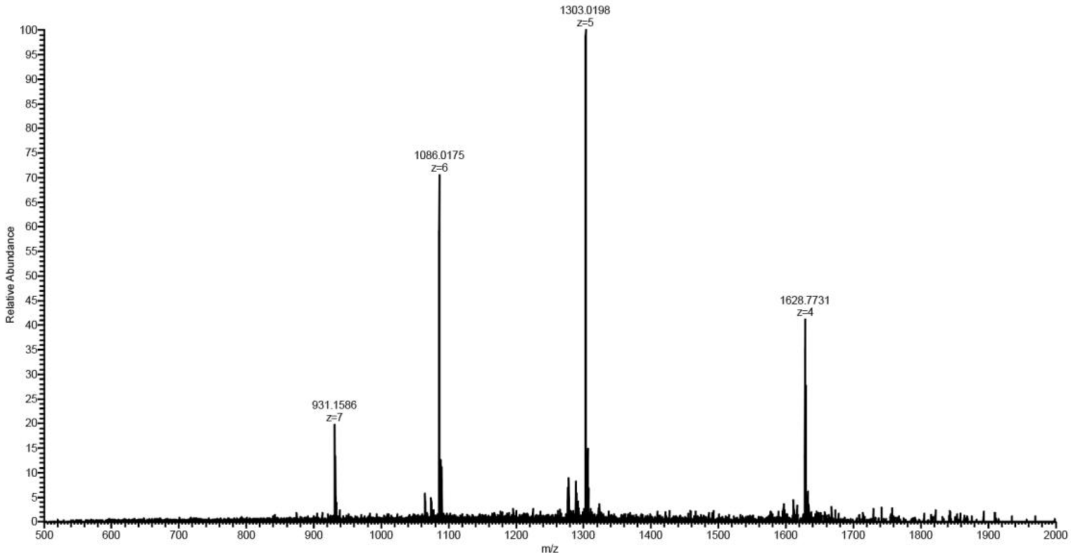

MS spectra of unmodified aprotinin

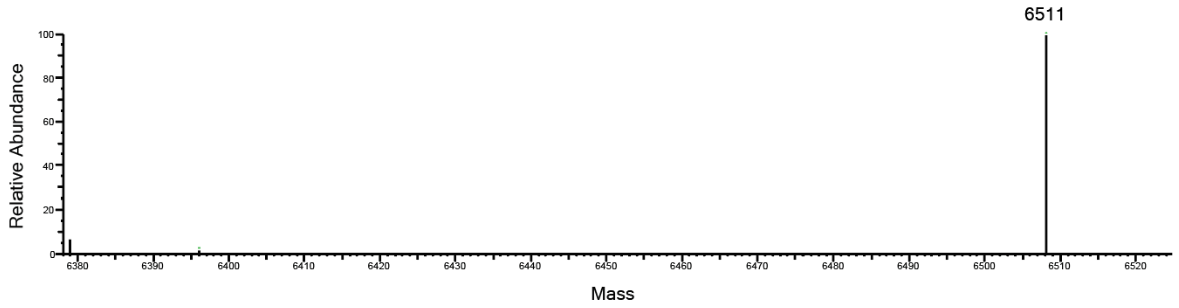

Deconvoluted MS spectra of unmodified aprotinin

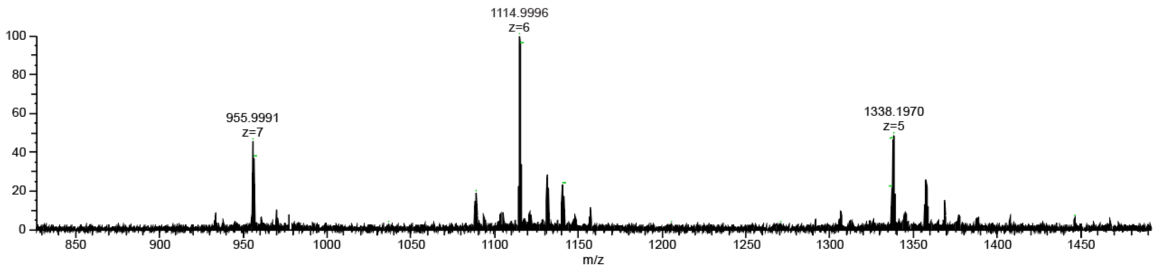

MS Spectra of **1a** modified aprotinin

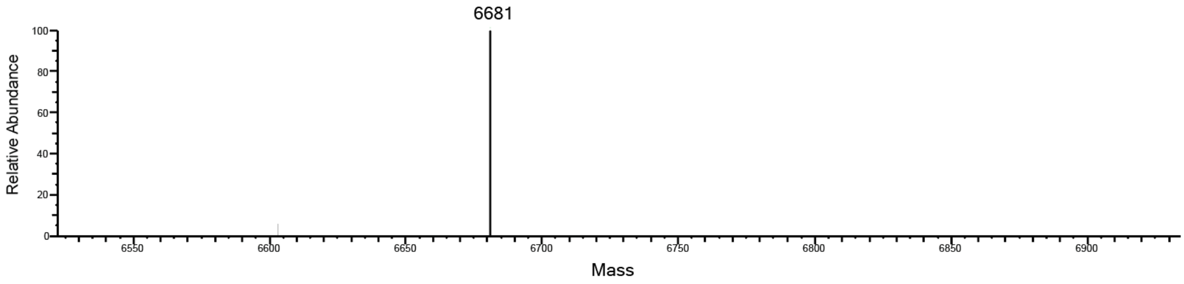

Deconvoluted MS spectra of **1a** modified Aprotinin

Supplementary Fig. 19. Labeling of methionine in Ubiquitin by CuNiP (PDB: 1UBQ).

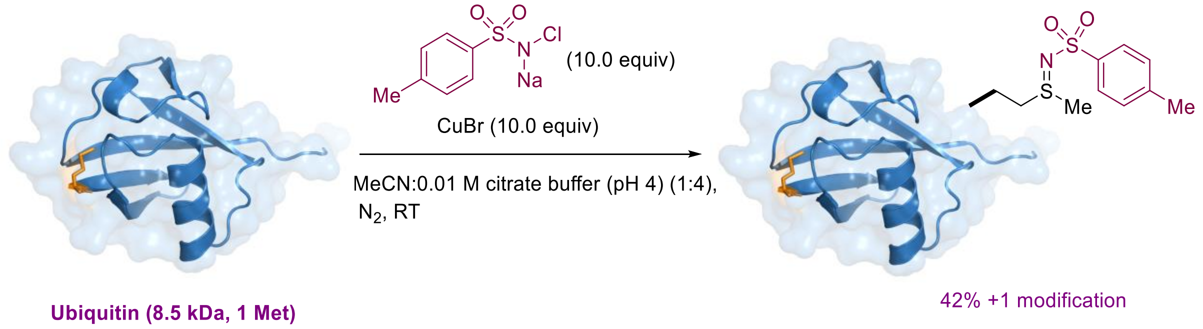

MQIFVKTLTGKTITLEVEPSDTIENVKAKIQDKEGIPPDQQRLIFAGKQLEDGRTLSDYNIQKESTLHLVLRLRGG

Ubiquitin (1 mg, 0.12 µmol, 1.0 equiv) was dissolved in MeCN: 0.01M citrate buffer (1:4, 800 µL) and CuBr (12 mM in MeCN, 100 µL, 1.2 µmol), **1a** (12 mM in 0.01M citrate buffer, 100 µL, 1.2 µmol) were added sequentially. The reaction mixture was incubated at 25 °C for 2 h under nitrogen atmosphere followed by the addition of 10 µL of 0.5 N HCl. The crude reaction mixture was passed through Amicon Ultra 3 kDa spin-concentrator and washed with H_2O (7×0.5 mL) to remove the small molecule impurities. This labeled protein was lyophilized, redissolved in 0.1% formic acid in H_2O, and analyzed using LC-MS. The conversion was found to be >95%. Intact mass analysis shows 42% +1 modification.

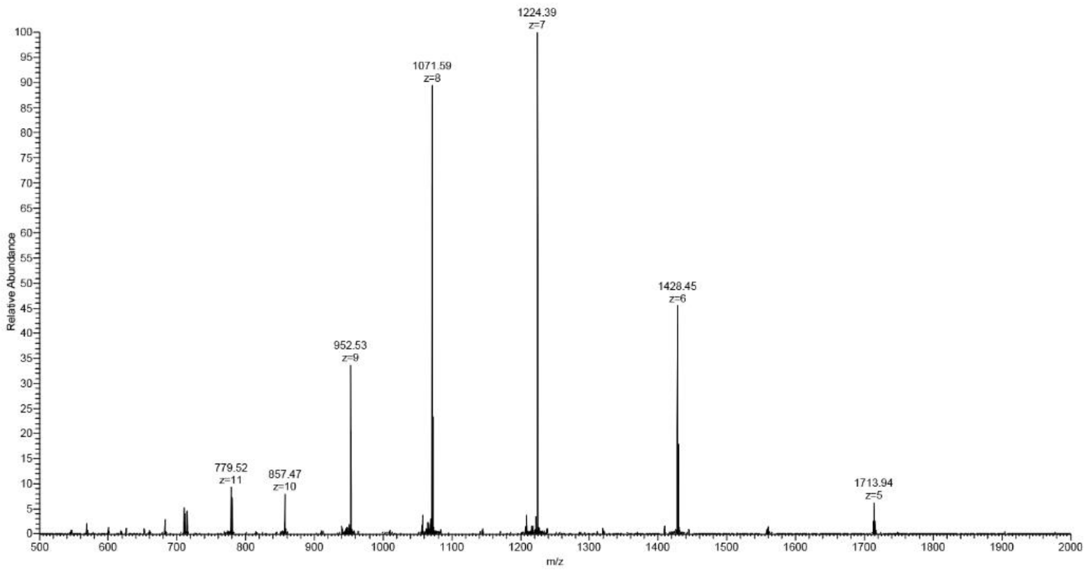

MS spectra of the unmodified ubiquitin

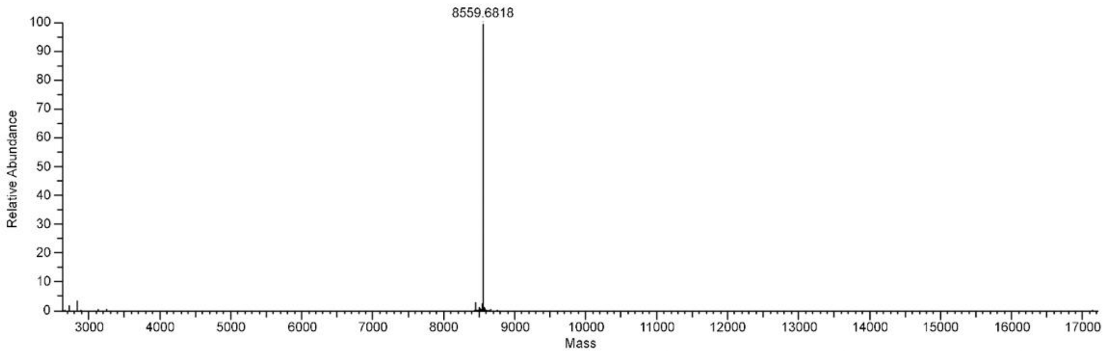

Deconvoluted MS spectra of unmodified ubiquitin

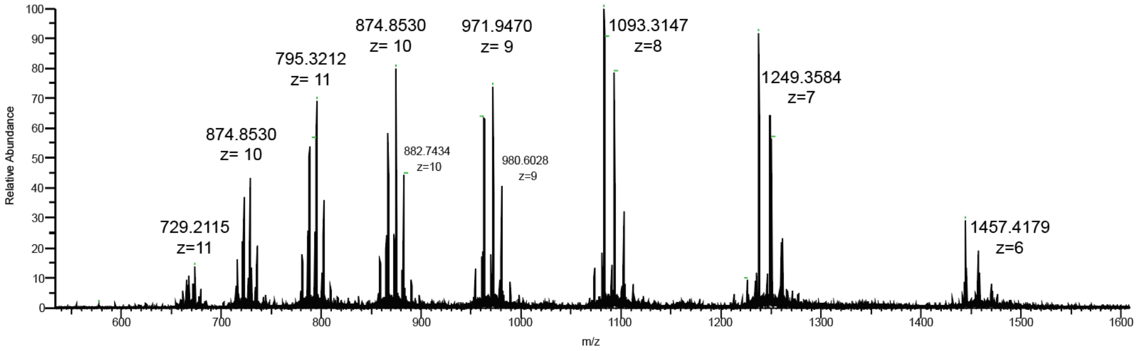

MS spectra of **1a** modified ubiquitin

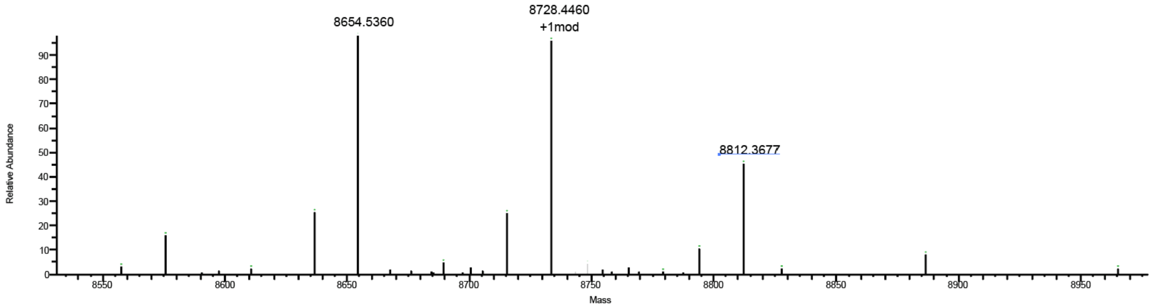

Deconvoluted MS spectra of **1a** modified ubiquitin

Supplementary Fig. 20. Labeling of methionine in Ribonuclease-A by CuNiP (PDB: 1KF5).

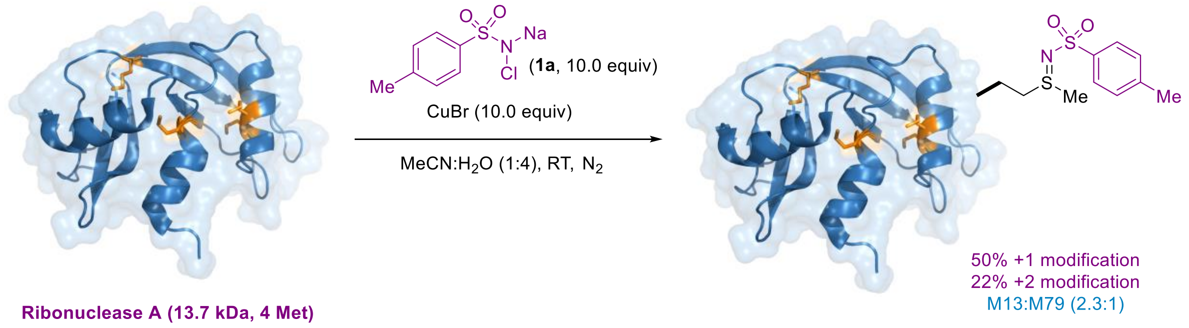

KETAAAKFERQHMDSSTSAASSSNYCNQMMKSRNLTKDRCKPVNTFVHESLADVQAVCSQ
KNVACKNGQTNCYQSYSTMSITDCRETGSSKYPNCAYKTTQANKHIIVACEGNPYVPVHFDASV

Ribonuclease A (1.6 mg, 0.12 µmol, 1.0 equiv) was dissolved in MeCN: H_2O (1:4, 800 µL) and CuBr (12 mM in MeCN, 100 µL, 1.2 µmol), **1a** (12 mM in H_2O, 100 µL, 1.2 µmol) were added sequentially. The reaction mixture was incubated at 25 °C for 2 h under nitrogen atmosphere followed by the addition of 10 µL of 0.5 N HCl. The crude reaction mixture was passed through Amicon Ultra 3 kDa spin-concentrator and washed with H_2O (7×0.5 mL) to remove the small molecule impurities. This labeled protein was lyophilized, redissolved in 0.1% formic acid in H_2O and analyzed using LC-MS. The conversion was found to be >95%.

Intact mass analysis of the protein shows 50% of +1 modification and 22% +2 modification. MS/MS analysis of the digested protein shows M13:M79 labeling ratio as 2.3:1.

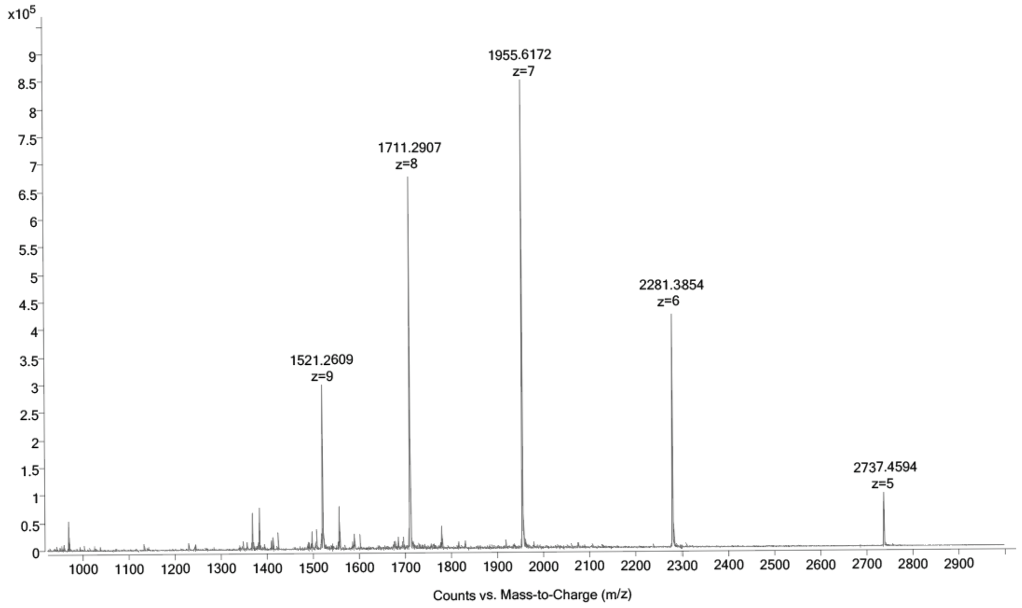

MS spectra of unmodified ribonuclease-A

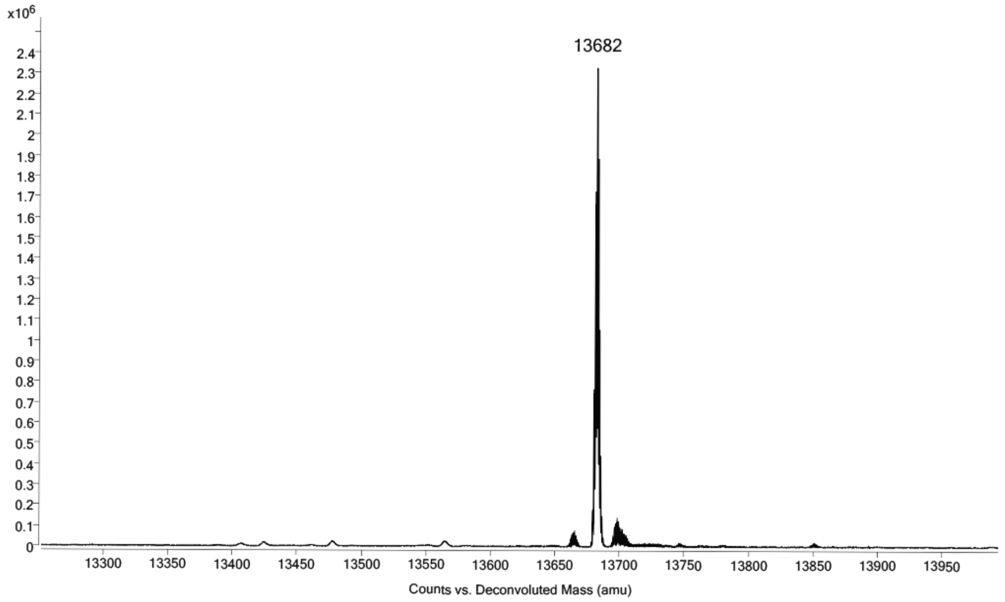

Deconvoluted MS spectra of unmodified ribonuclease-A

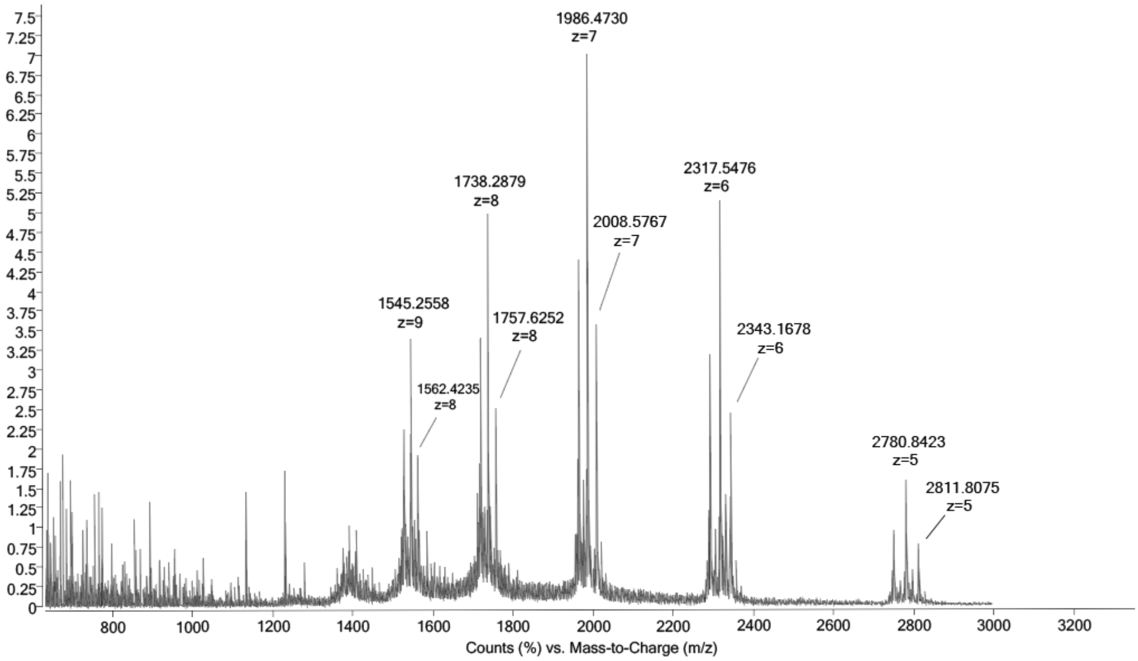

MS spectra of **1a** modified ribonuclease-A

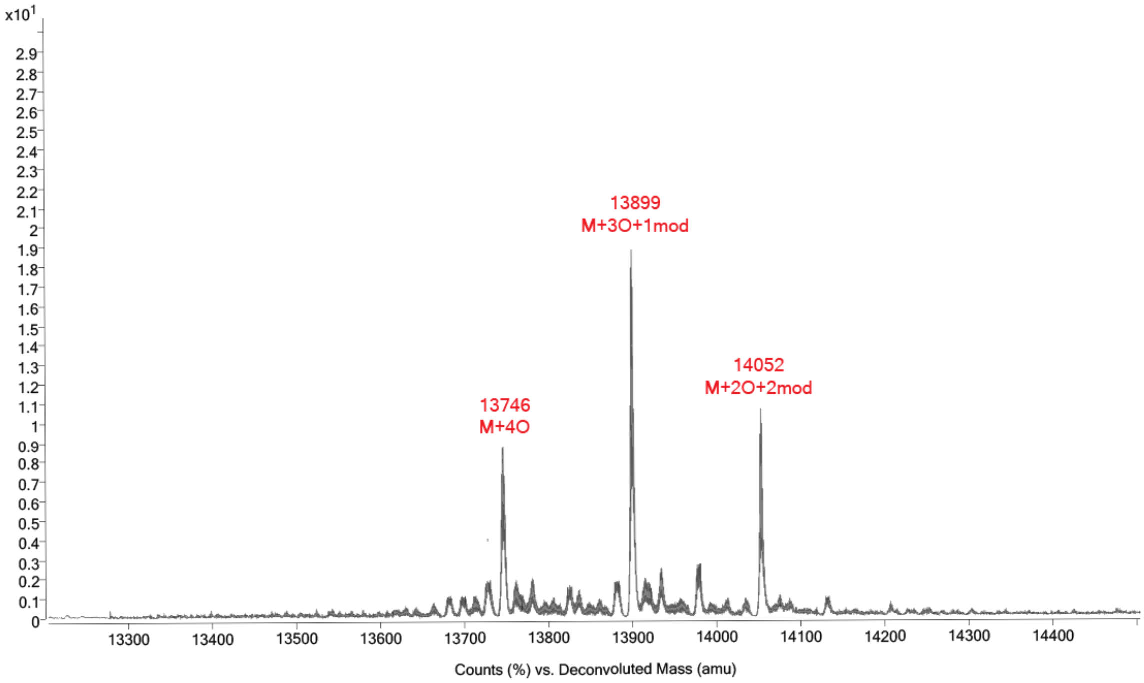

Deconvoluted MS spectra of **1a** modified ribonuclease-A

MS/MS Analysis of 1a modified ribonuclease A :

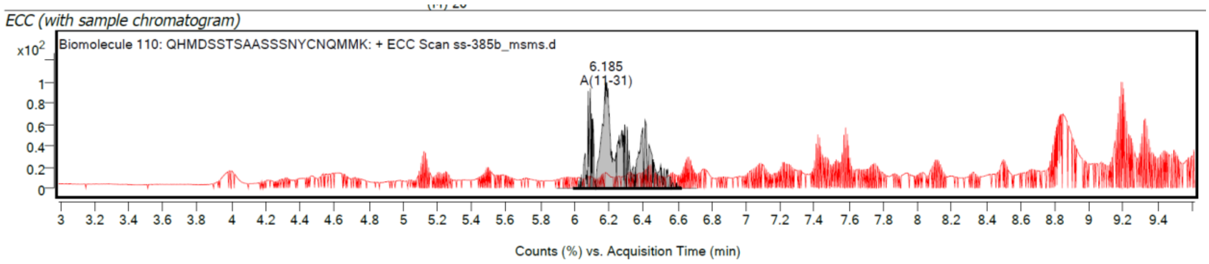

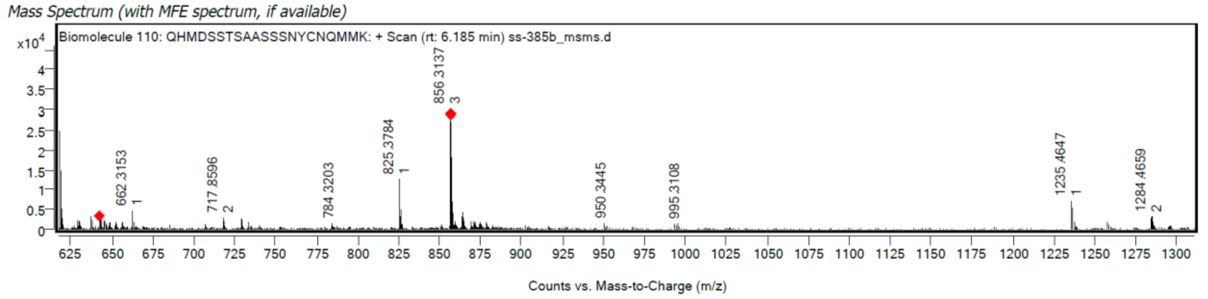

Identified Peptide Sequence: QHMDSSTSAASSSNYCNQMMK (AA11-AA31)

b^+	b^{++}		AA		y^+	y^{++}
129.065854	65.036565	1	Q	21		
266.124766	133.566021	2	H	20	2438.876335	1219.941806
566.184951	283.596113	3	**M+Mod**	19	2301.817424	1151.412350
681.211894	341.109585	4	D	18	2001.757239	1001.382258
768.243922	384.625599	5	S	17	1886.730296	943.868786
855.275950	428.141613	6	S	16	1799.698267	900.352772
956.323629	478.665453	7	T	15	1712.666239	856.836758
1043.355657	522.181467	8	S	14	1611.618560	806.312918
1114.392771	557.700024	9	A	13	1524.586532	763.796904
1185.429885	593.218581	10	A	12	1453.549418	727.278347
1272.461913	636.734595	11	S	11	1382.512304	691.759790
1359.493942	680.250609	12	S	10	1295.480276	648.243776
1446.525970	723.766623	13	S	9	1208.448248	604.727762
1560.568898	780.788087	14	N	8	1121.416219	561.211748
1723.632226	862.319751	15	Y	7	1007.373292	504.190284
1884.646911	942.827094	16	**C+IAA**	6	844.309963	422.658620
1998.689838	999.848557	17	N	5	683.295279	342.151277
2126.748416	1063.877846	18	Q	4	569.252351	285.129814
2273.788901	1137.398089	19	M+O	3	441.193774	221.100525
2420.829385	1210.918331	20	M+O	2	294.153289	147.580283
		21	K	1	147.112804	74.060040

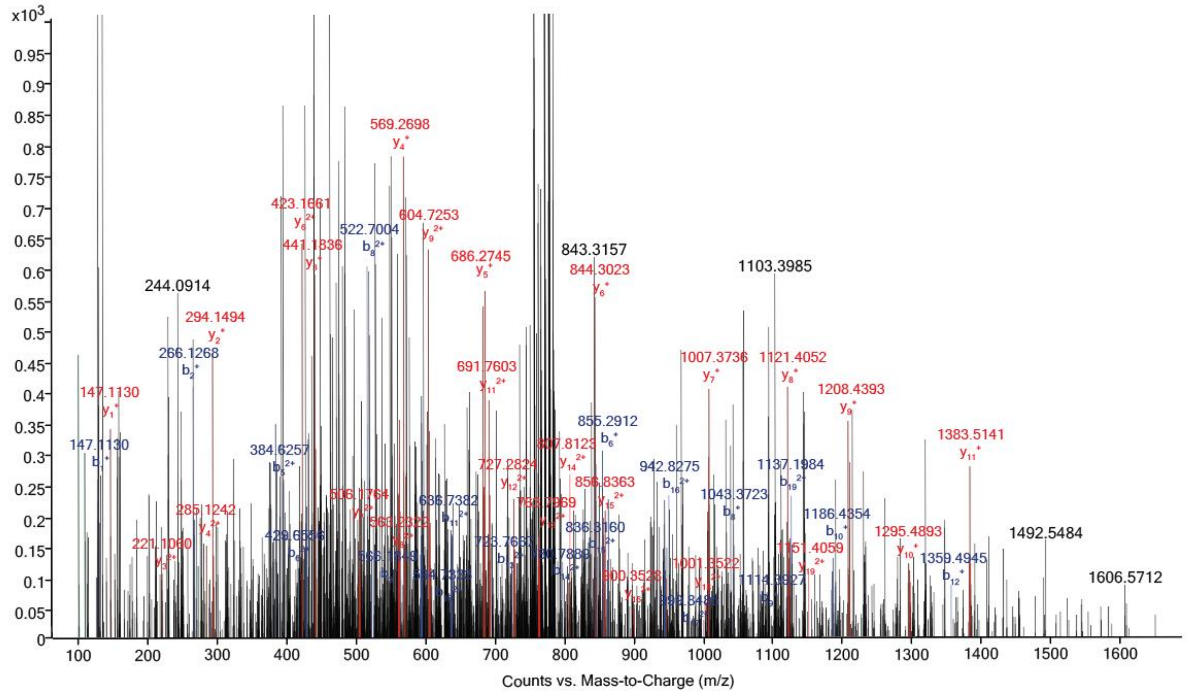

ECC (with sample chromatogram)

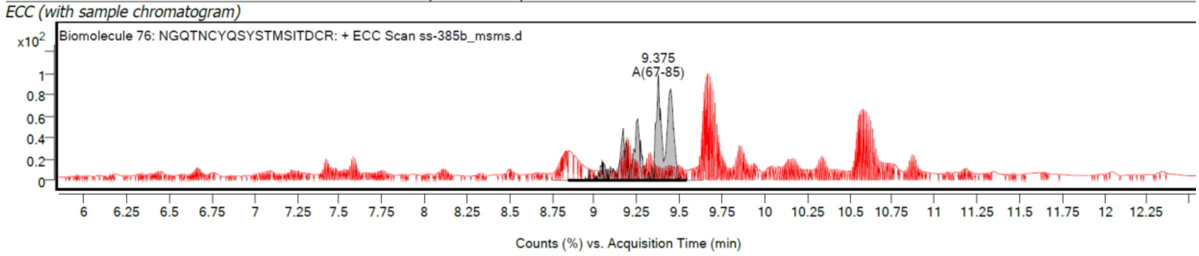

Mass Spectrum (with MFE spectrum, if available)

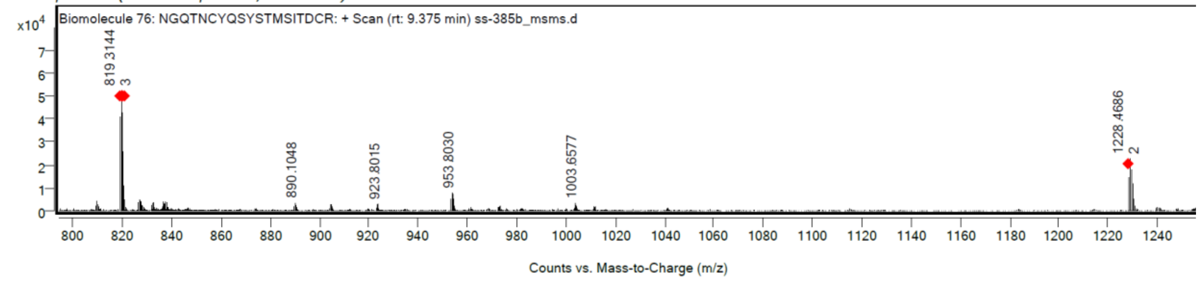

Identified Peptide Sequence: NGQTNCYQSYST**M**SITDCR (AA67-AA85)

b^+	b^{++}		AA		y^+	y^{++}
115.050204	58.028740	1	N	19		
172.071668	86.539472	2	G	18	2341.954837	1171.481057
301.114245	151.060761	3	**Q-Deamid**	17	2284.933373	1142.970325
402.161924	201.584600	4	T	16	2155.890796	1078.449036
516.204851	258.606064	5	N	15	2054.843117	1027.925197
676.235536	338.621406	6	**C+IAA**	14	1940.800190	970.903733
839.298864	420.153070	7	Y	13	1780.769505	890.888391
967.357442	484.182359	8	Q	12	1617.706177	809.356727
1054.389470	527.698373	9	S	11	1489.647599	745.327438
1217.452799	609.230038	10	Y	10	1402.615571	701.811424
1304.484827	652.746052	11	S	9	1239.552242	620.279759

1405.532506	703.269891	12	T	8	1152.520214	576.763745
1705.664690	853.335983	13	**M+Mod**	7	1051.472535	526.239906
1792.696719	896.851998	14	S	6	751.340351	376.173814
1905.780783	953.394030	15	I	5	664.308322	332.657799
2006.828461	1003.917869	16	T	4	551.224258	276.115767
2121.855404	1061.431340	17	D	3	450.176580	225.591928
2281.886089	1141.446683	18	**C+IAA**	2	335.149637	168.078457
		19	R	1	175.118952	88.063114

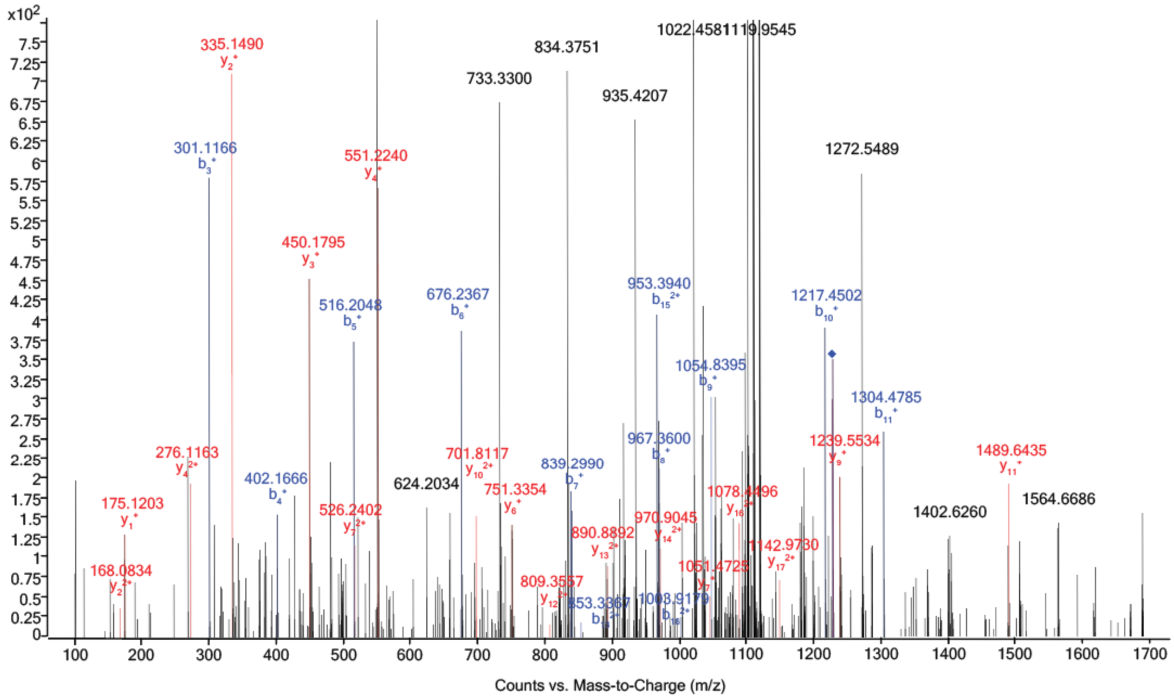

Supplementary Fig. 21. Labeling of methionine in Lysozyme Chicken Egg-White by CuNiP (PDB: 1DPX).

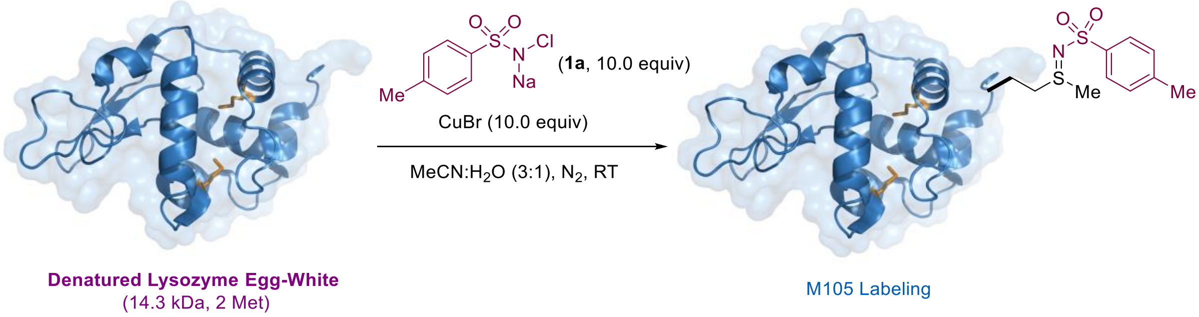

KVFGRCELAAAMKRHGLDNYRGYSLGNWVCAAKFESNFNTQATNRNTDGSTDYGILQINSRWWCN
DGRTPGSRNLCNIPCSALLSSDITASVNCAKKIVSDGNGMNAWVAWRNRCKGTDVQAWIRGCRL

Reaction with intact Lysozyme egg-white:

Lysozyme egg-white (1.71 mg, 0.12 μmol, 1.0 equiv) was dissolved in MeCN: H_2O (1:4, 800 μL) and CuBr (60 mM in MeCN, 100 μL, 6.0 μmol), **1a** (60 mM in H_2O, 100 μL, 6 μmol) were added sequentially. The reaction mixture was incubated at 25 °C for 2 h under nitrogen atmosphere followed by the addition of 10 μL of 0.5 N HCl. The crude reaction mixture was

passed through Amicon Ultra 3 kDa spin-concentrator and washed with H_2O (7×0.5 mL) to remove the small molecule impurities This labeled protein was lyophilized, redissolved in 0.1% formic acid in H_2O and analyzed using LC-MS. Intact mass analysis of the protein shows no labeling as well as no oxidation of any methionine residue.

Note: *Lysozyme egg-white was denatured using* ***method VI****. The intact mass of the denatured protein shows addition of +8 Da due to breakage of 4 di-sulfide bonds.*

Reaction with denatured Lysozyme egg-white:

Denatured lysozyme egg-white (1.71 mg, 0.12 µmol, 1.0 equiv) was dissolved in MeCN:H_2O (3:1, 800 µL) and CuBr (12 mM in MeCN, 100 µL, 1.2 µmol), **1a** (12 mM in H_2O, 100 µL, 1.2 µmol) were added sequentially. The reaction mixture was incubated at 25 °C for 2 h under nitrogen atmosphere followed by the addition of 10 µL of 0.5 N HCl. The crude reaction mixture was passed through Amicon Ultra 3 kDa spin-concentrator and washed with H_2O (7×0.5 mL) to remove the small molecule impurities. The labeled protein was digested and MS/MS analysis shows exclusive labeling of M105.

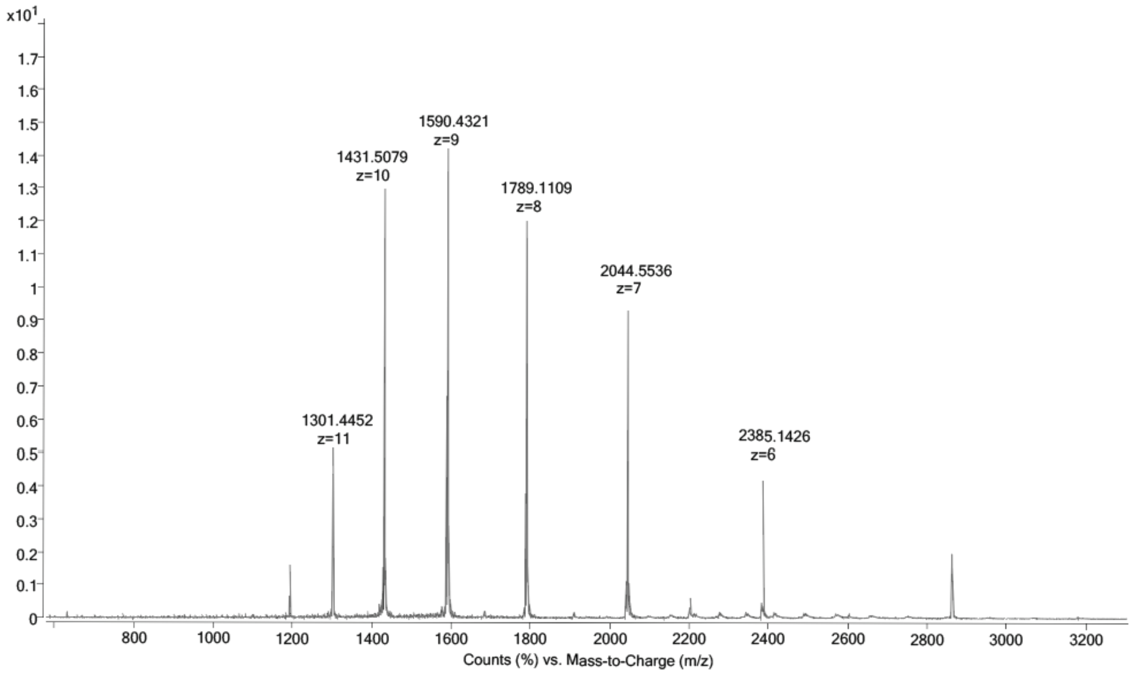

MS spectra of unmodified lysozyme egg white

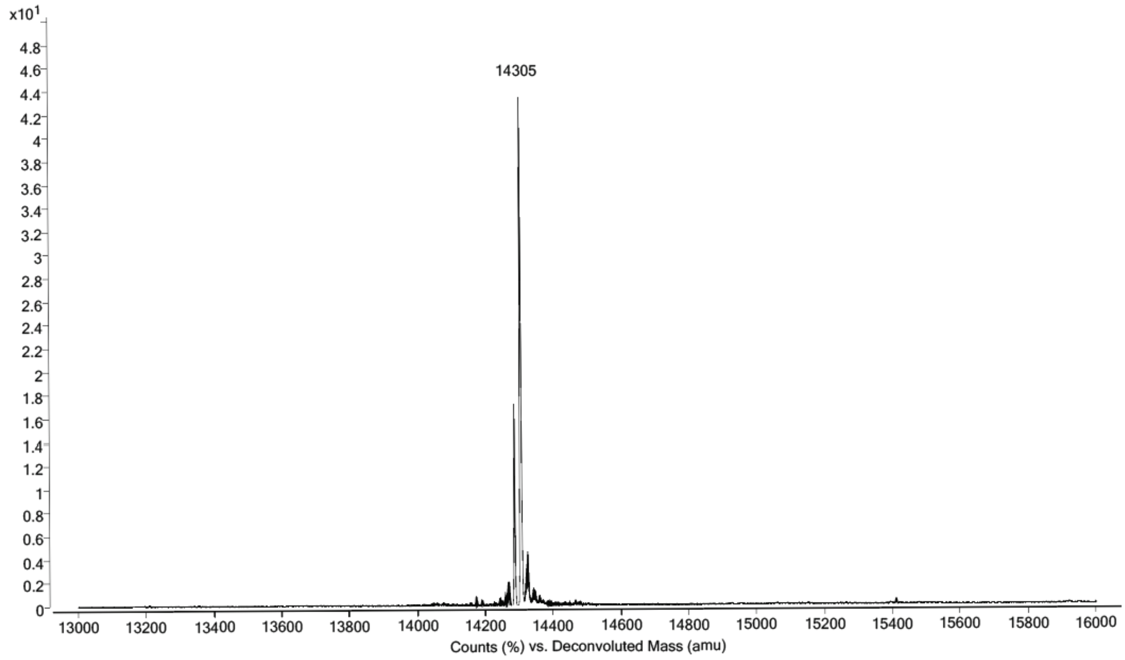

Deconvoluted MS spectra of unmodified lysozyme egg white

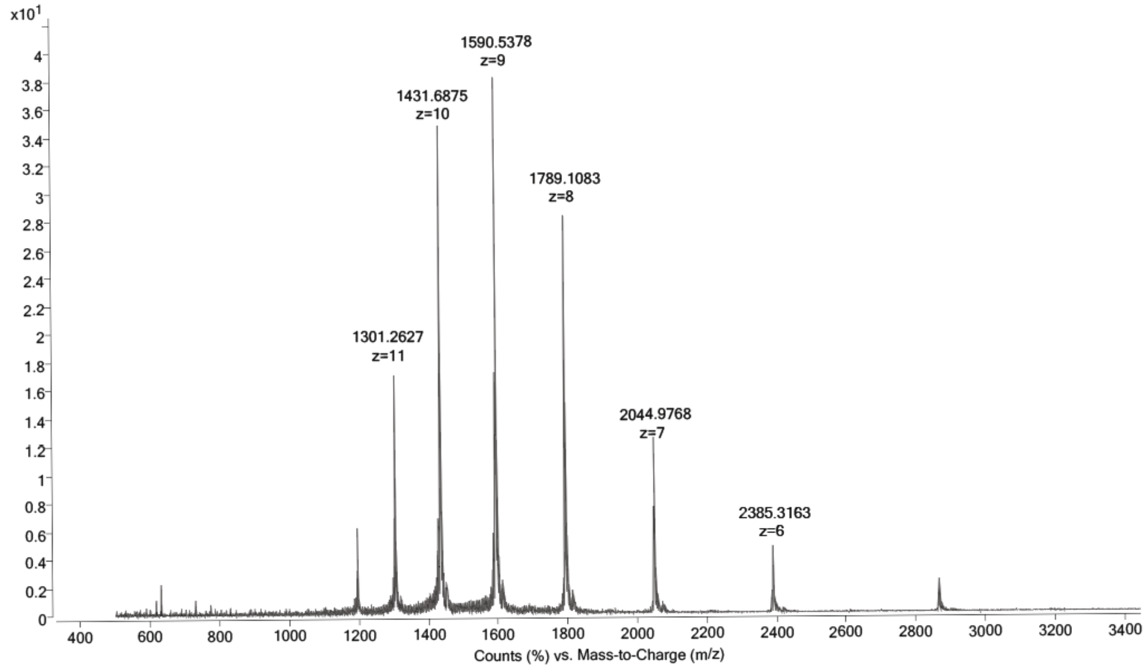

MS spectra of **1a** (50 equiv) modified intact lysozyme egg white shows no labeling of methionine

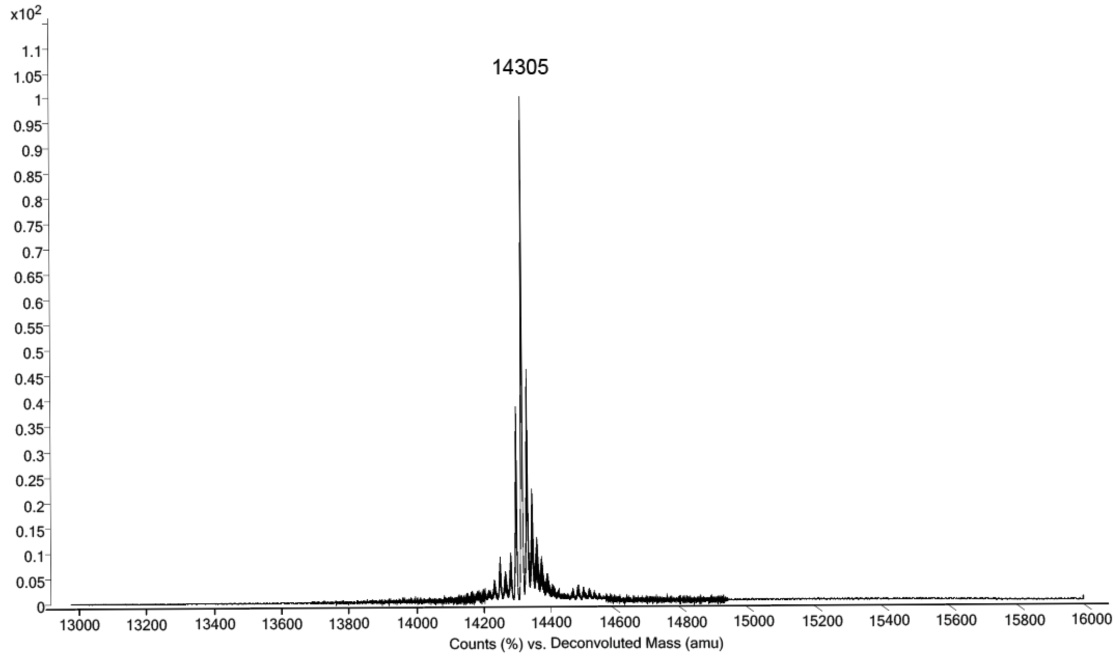

Deconvoluted MS spectra of **1a** (50 equiv) modified intact lysozyme egg white shows no labeling of methionine

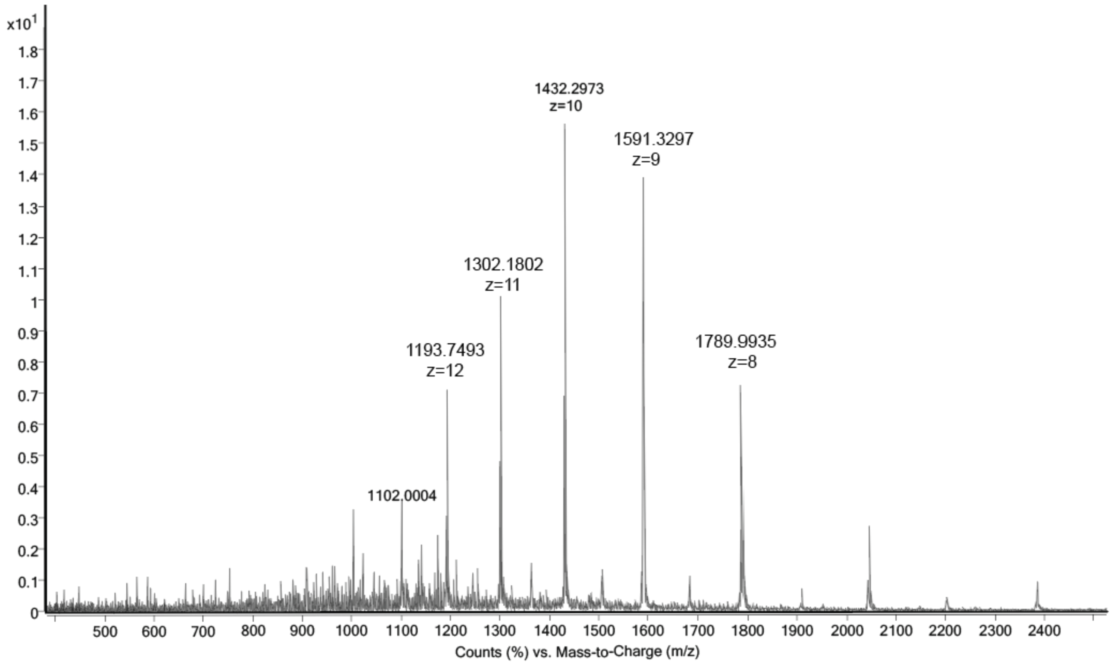

MS spectra of denatured lysozyme egg-white

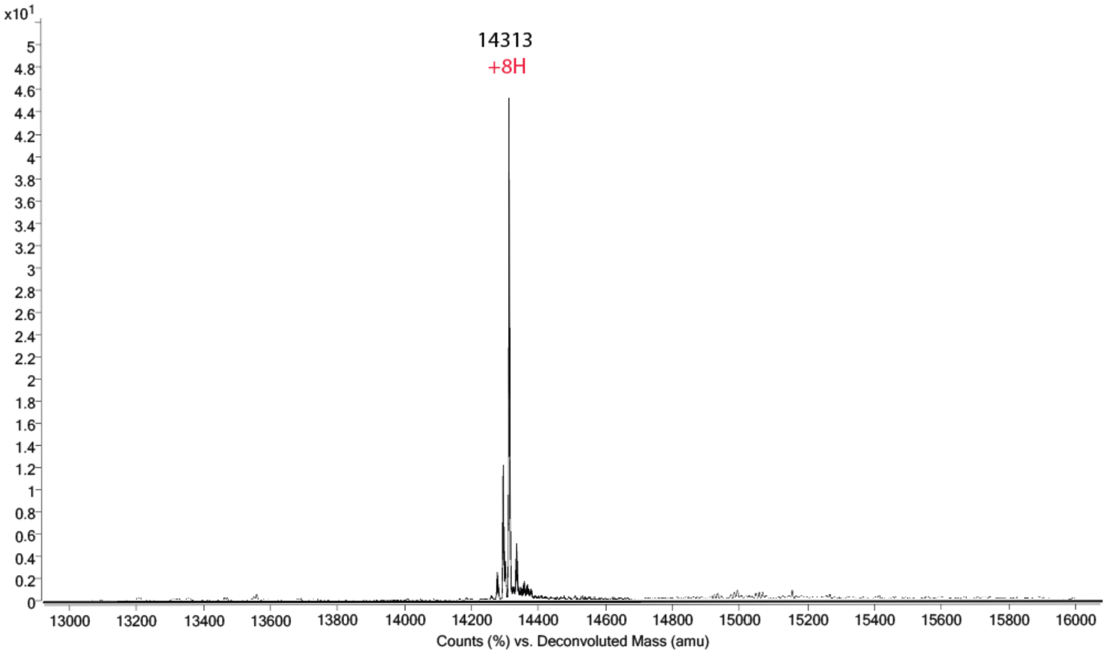

Deconvoluted MS spectra of denatured lysozyme egg-white

MS/MS Analysis 1a modified denatured lysozyme egg white:

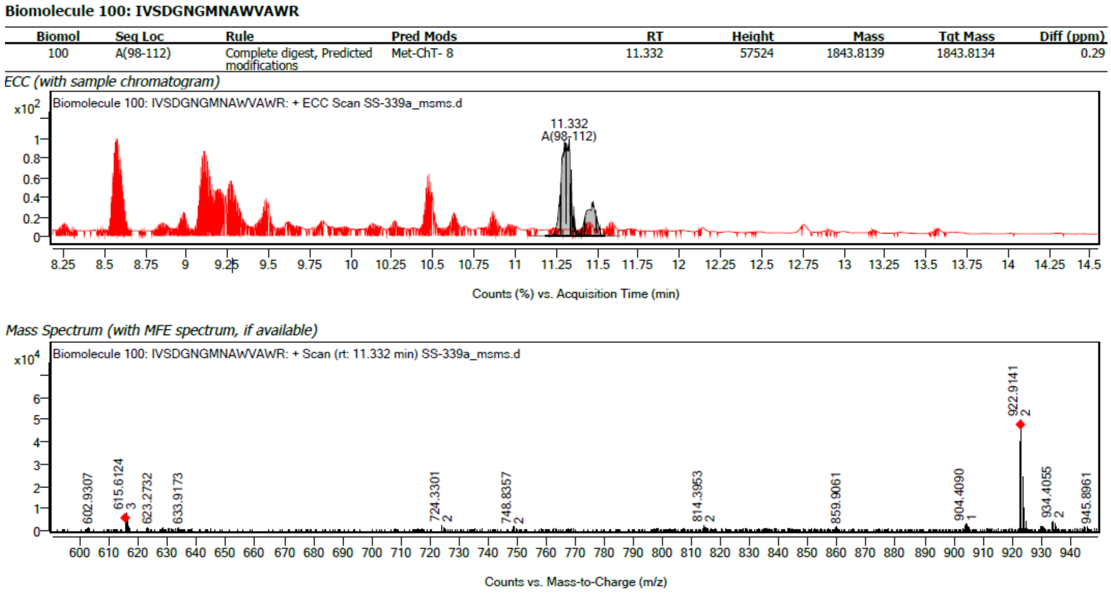

Identified Peptide Sequence: IVSDGNG**M**NAWVAWR (AA98-AA112)

b⁺	b²⁺	b³⁺		AA		y⁺	y²⁺	y³⁺
114.091340	57.549308	38.701964	1	I	15			
213.159754	107.083515	71.724769	2	V	14	1731.736622	866.371949	577.917058
300.191783	150.599530	100.735445	3	S	13	1632.668208	816.837742	544.894254
415.218726	208.113001	139.077760	4	D	12	1545.636180	773.321728	515.883578
472.240190	236.623733	158.084914	5	G	11	1430.609237	715.808257	477.541263
586.283117	293.645197	196.099223	6	N	10	1373.587773	687.297525	458.534109
643.304581	322.155929	215.106378	7	G	9	1259.544846	630.276061	420.519800
943.364816	472.186046	315.126456	8	**M+mod**	8	1202.523382	601.765329	401.512645
1057.407743	529.207510	353.140765	9	N	7	902.463147	451.735212	301.492567

1128.444857	564.726067	376.819803	10	A	6	788.420220	394.713748	263.478258
1314.524170	657.765723	438.846241	11	W	5	717.383106	359.195191	239.799220
1413.592584	707.299930	471.869046	12	V	4	531.303793	266.155535	177.772782
1484.629698	742.818487	495.548083	13	A	3	432.235379	216.621328	144.749977
1670.709011	835.858143	557.574521	14	W	2	361.198265	181.102771	121.070939
			15	R	1	175.118952	88.063114	59.044502

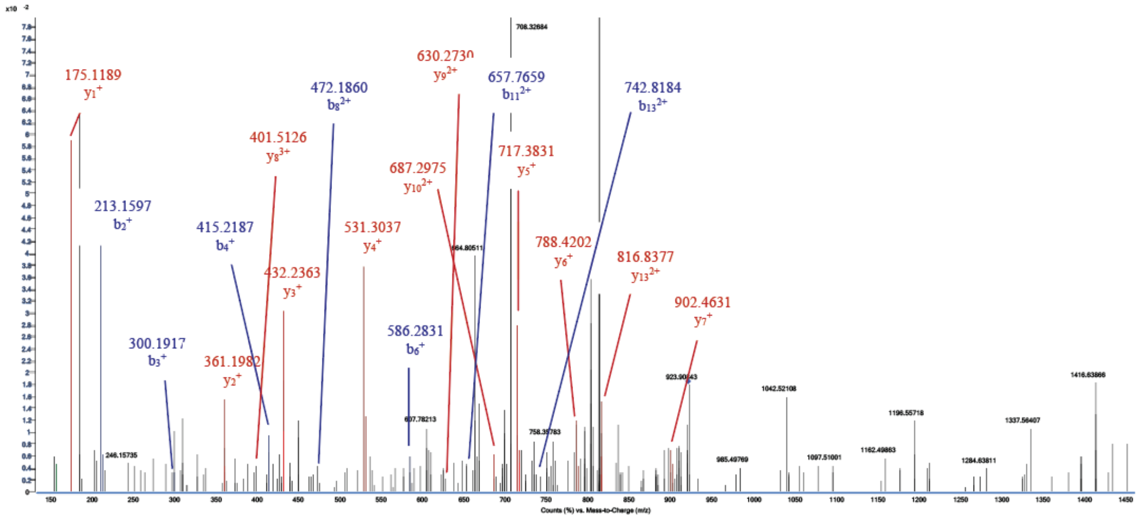

Supplementary Fig. 22. Labeling of methionine in Human Lysozyme by CuNiP (PDB: 1REX).

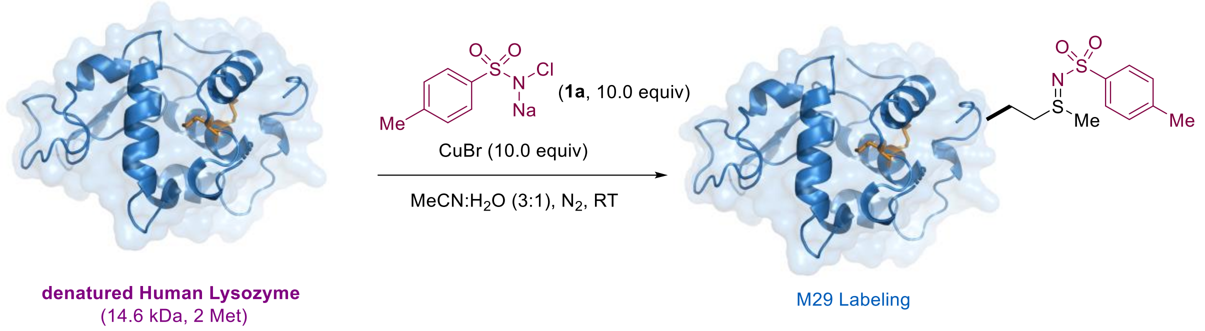

KVFERCELARTLKRLGMDGYRGISLANWMCLAKWESGYNTRATNYNAGDRSTDYGIFQINSRYWCND
GKTPGAVNACHLSCSALLQDNIADAVACAKRVVRDPQGIRAWVAWRNRCQNRDVRQYVQGCGV

Reaction with intact human Lysozyme: Human lysozyme (1.71 mg, 0.12 µmol, 1.0 equiv) was dissolved in MeCN: H_2O (1:4, 800 µL) and CuBr (60 mM in MeCN, 100 µL, 6.0 µmol), **1a** (60 mM in H_2O, 100 µL, 6 µmol) were added sequentially. The reaction mixture was incubated at 25 ^oC for 2 h under nitrogen atmosphere followed by the addition of 10 µL of 0.5 N HCl. The crude reaction mixture was passed through Amicon Ultra 3 kDa spin-concentrator and washed with H_2O (7×0.5 mL) to remove the small molecule impurities This labeled protein was lyophilized, redissolved in 0.1% formic acid in H_2O, and analyzed using LC-MS. Intact mass analysis of the protein shows no labeling as well as no oxidation of any methionine residue.

Note: *Human Lysozyme was denatured using* ***method VI****. The intact mass of the denatured protein shows addition of +8 Da due to breakage of 4 di-sulfide bonds.*

Reaction with denatured human Lysozyme: Denatured human lysozyme (1.71 mg, 0.12 µmol, 1.0 equiv) was dissolved in MeCN:H_2O (3:1, 800 µL) and CuBr (12 mM in MeCN, 100 µL, 1.2 µmol), **1a** (12 mM in H_2O, 100 µL, 1.2 µmol) were added sequentially. The reaction mixture was incubated at 25 ^oC for 2 h under nitrogen atmosphere followed by the addition of 10 µL of 0.5 N HCl. The crude reaction mixture was passed through Amicon Ultra 3 kDa spin-concentrator and washed with H_2O (7×0.5 mL) to remove the small molecule impurities. The labeled protein was digested and MS/MS analysis shows exclusive labeling of M29.

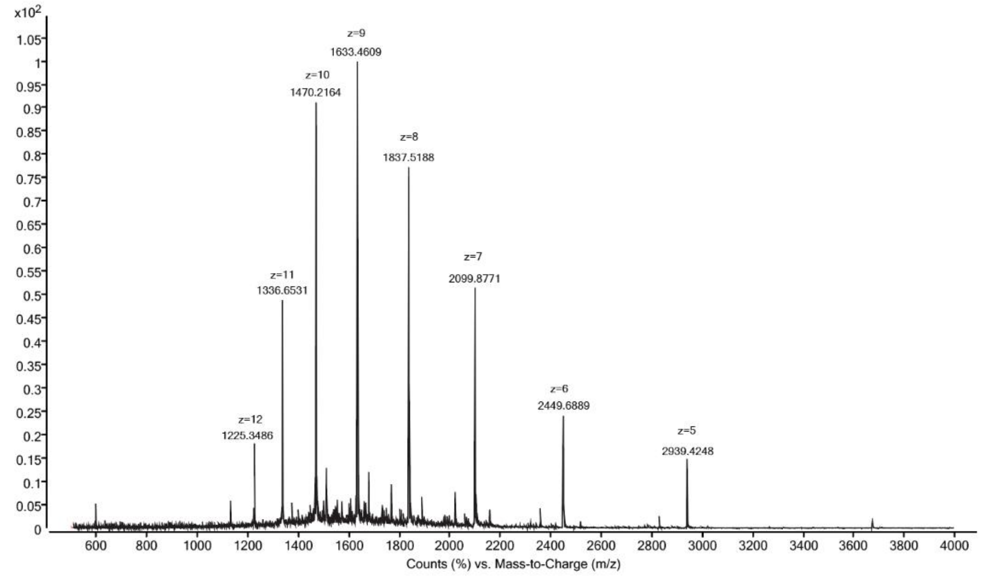

MS spectra of unmodified human lysozyme

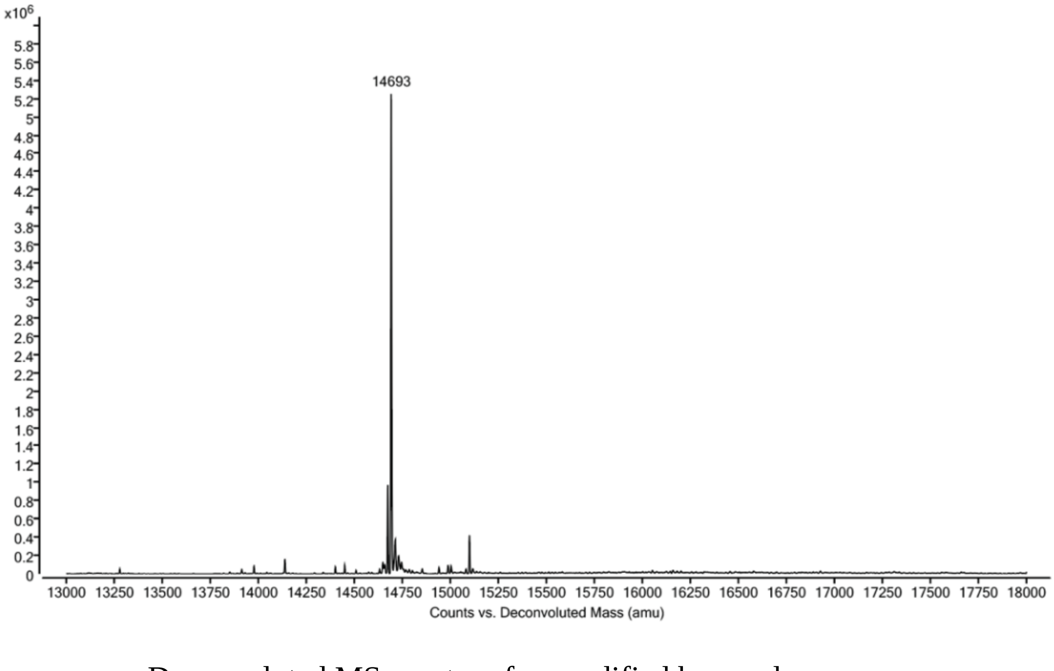

Deconvoluted MS spectra of unmodified human lysozyme

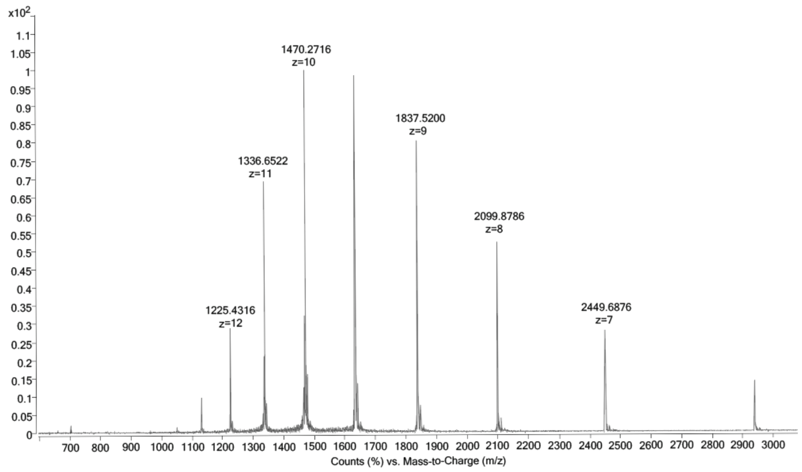

MS spectra of **1a** (50 equiv) modified human lysozyme shows no labeling

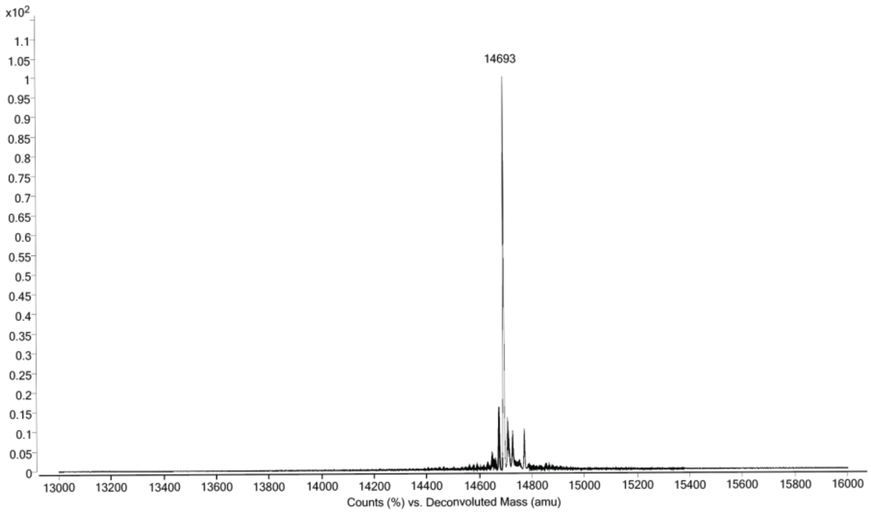

Deconvoluted MS spectra of **1a** (50 equiv) modified human lysozyme shows no labeling

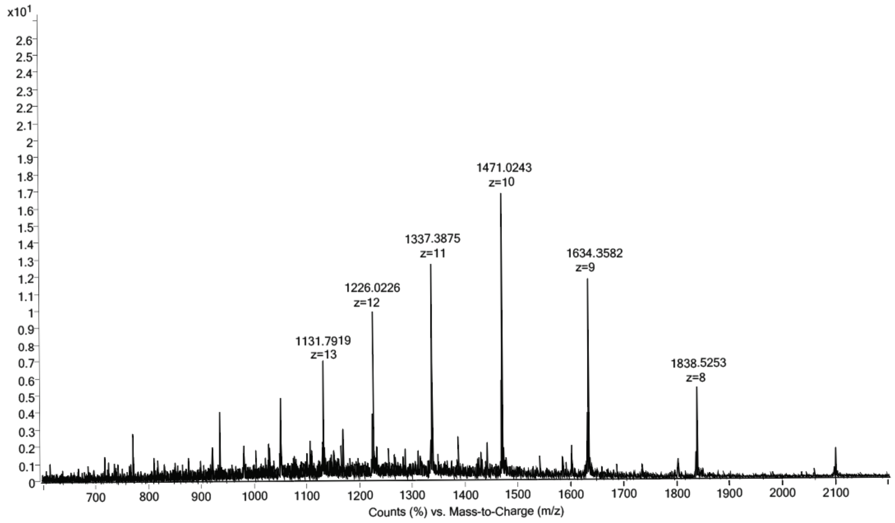

MS Spectra of unmodified denatured human lysozyme

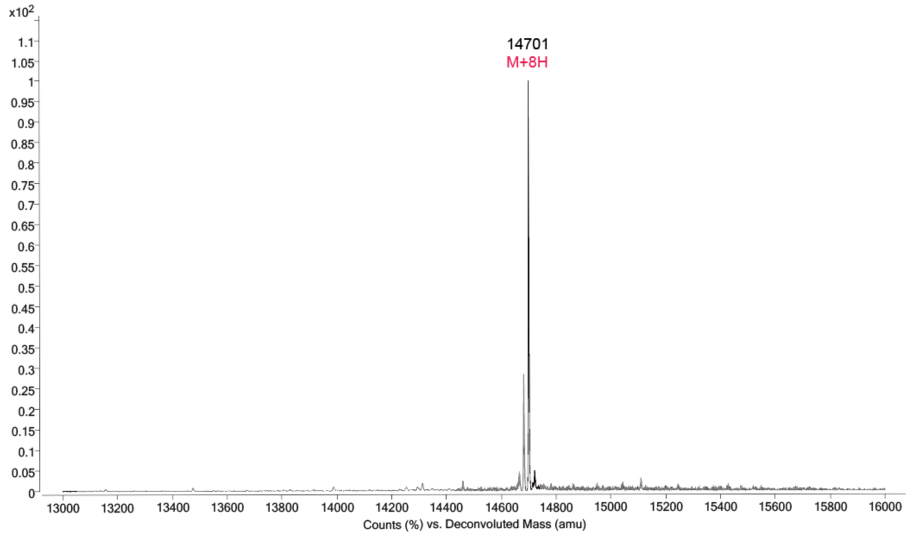

Deconvoluted MS spectra of denatured human lysozyme

MS/MS Analysis of 1a modified denatured human lysozyme :

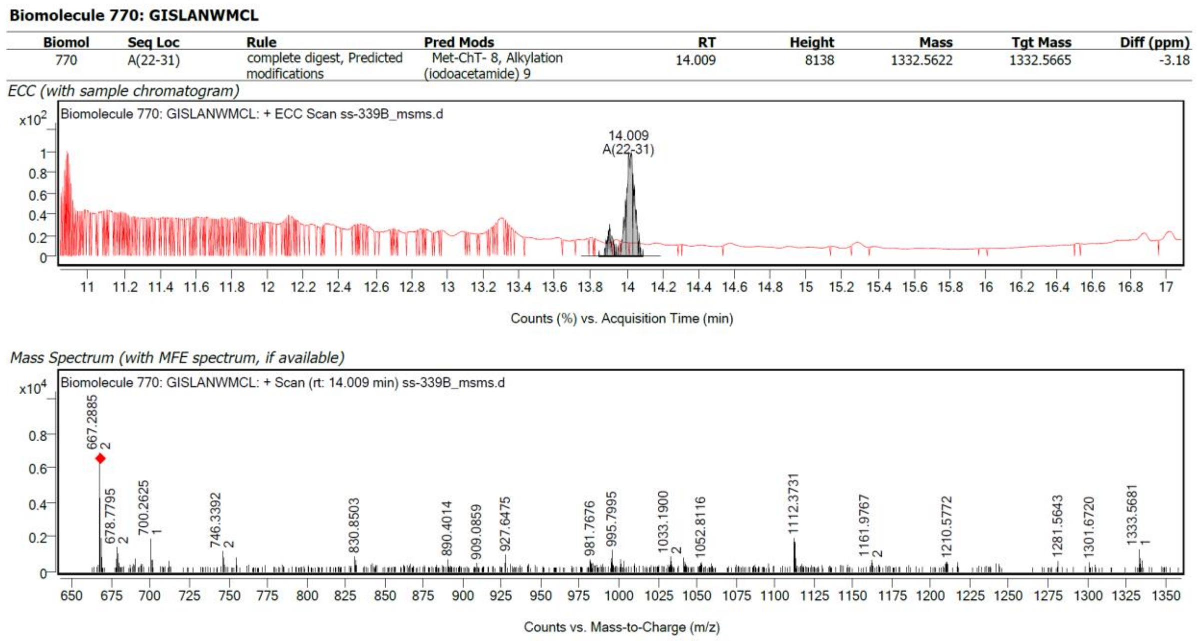

Identified Peptide Sequence: GISLANW**M**CL (AA22-AA31)

b^+	b^{2+}		AA		y^+	y^{2+}
58.028740	29.518008	1	G	10		
171.112804	86.060040	2	I	9	1276.551335	638.779306
258.144833	129.576055	3	S	8	1163.467271	582.237274
371.228897	186.118087	4	L	7	1076.435243	538.721260
442.266010	221.636643	5	A	6	963.351179	482.179228
556.308938	278.658107	6	N	5	892.314065	446.660671
742.388251	371.697764	7	W	4	778.271137	389.639207
1042.448486	521.727881	8	**M+mod**	3	592.191824	296.599550
1202.478170	601.742723	9	**C+IAA**	2	292.131590	146.569433
		10	L	1	132.101905	66.554591

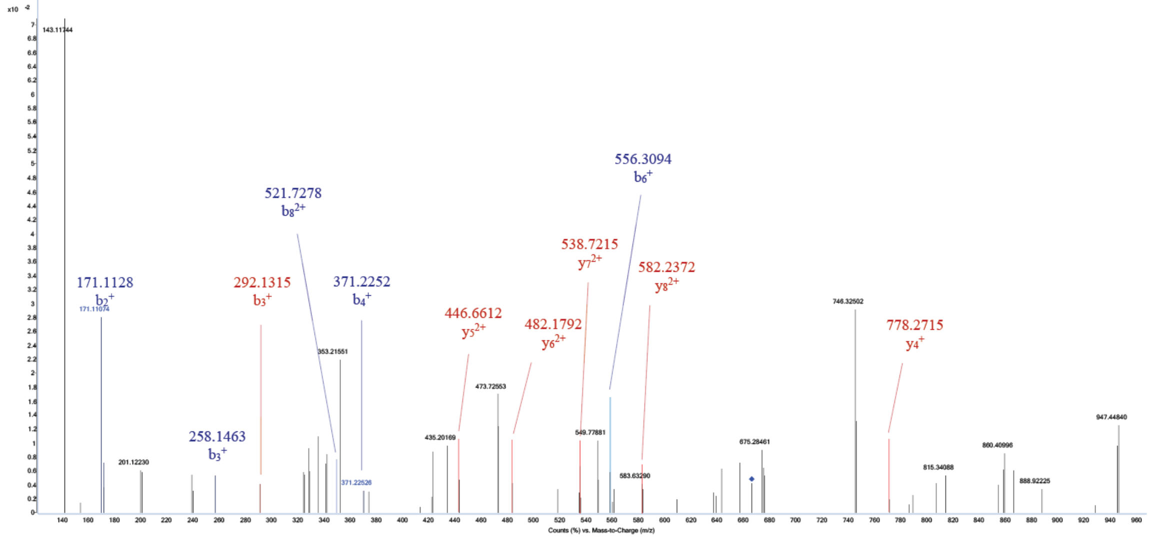

Supplementary Fig. 23. Labeling of methionine in α-Chymotrpsinogen A by CuNiP (PDB: 1EX3).

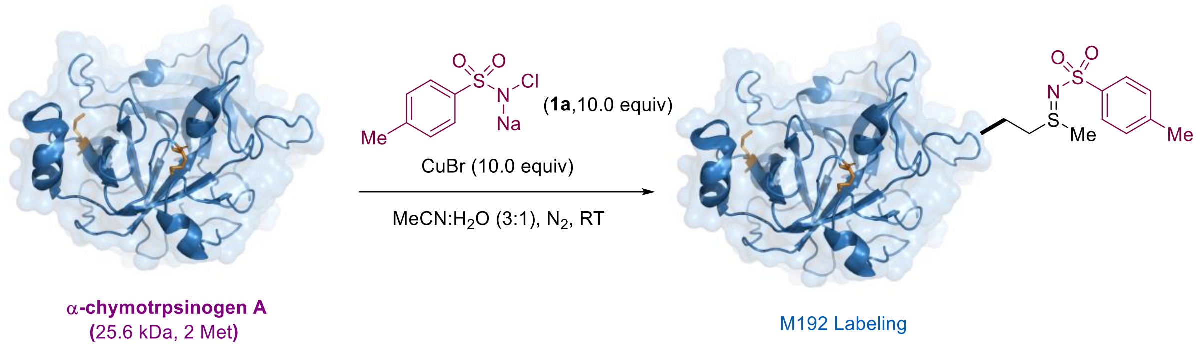

CGVPAIQPVLSGLSRIVNGEEAVPGSWPWQVSLQDKTGFHFCGGSLINENWVVTAAHCGVTTSDVVVAGEFDQGSSSEKIQKLK
IAKVFKNSKYNSLTINNDITLLKLSTAASFSQTVSAVCLPSASDDFAAGTTCVTTGWGLTRYTNANTPDRLQQASLPLLSNTNCKKY
WGTKIKDAMICAGASGVSSCMGDSGGPLVCKKNGAWTLVGIVSWGSSTCSTSTPGVYARVTALVNWVQQTLAAN

Reaction with intact α-Chymotrpsinogen A: α-Chymotrpsinogen A (3.0 mg, 0.12 µmol, 1.0 equiv) was dissolved in MeCN: H_2O (1:4, 800 µL) and CuBr (60 mM in MeCN, 100 µL, 6.0 µmol), **1a** (60 mM in H_2O, 100 µL, 6 µmol) were added sequentially. The reaction mixture was incubated at 25 ^{o}C for 2 h under nitrogen atmosphere followed by the addition of 10 µL of 0.5 N HCl. The crude reaction mixture was passed through Amicon Ultra 3 kDa spin-concentrator and washed with H_2O (7×0.5 mL) to remove the small molecule impurities This labeled protein was lyophilized, redissolved in 0.1% formic acid in H_2O, and analyzed using LC-MS. Intact mass analysis of the protein shows no labeling as well as no oxidation of any methionine residue.

Note: *α-Chymotrpsinogen A was denatured using* ***method VI****. The intact mass of the denatured protein shows addition of +10 Da due to breakage of 5 di-sulfide bonds*

Reaction with denatured α-Chymotrpsinogen A: α-Chymotrpsinogen A (3.0 mg, 0.12 µmol, 1.0 equiv) was dissolved in MeCN:H_2O (3:1, 800 µL) and CuBr (12 mM in MeCN, 100 µL, 1.2 µmol), **1a** (12 mM in H_2O, 100 µL, 1.2 µmol) were added sequentially. The reaction

mixture was incubated at 25 °C for 2 h under nitrogen atmosphere followed by the addition of 10 μL of 0.5 N HCl. The crude reaction mixture was passed through Amicon Ultra 3 kDa spin-concentrator and washed with H_2O (7×0.5 mL) to remove the small molecule impurities. The labeled protein was digested and MS/MS analysis shows exclusive labeling of M192.

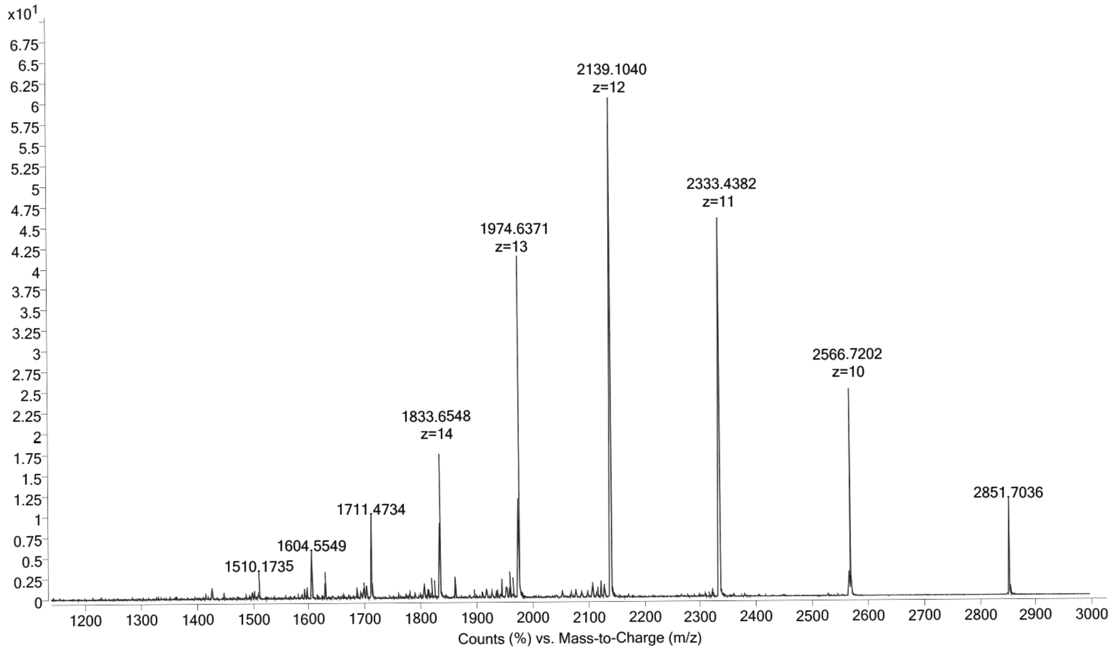

MS spectra of the unmodified α-chymotrpsinogen A

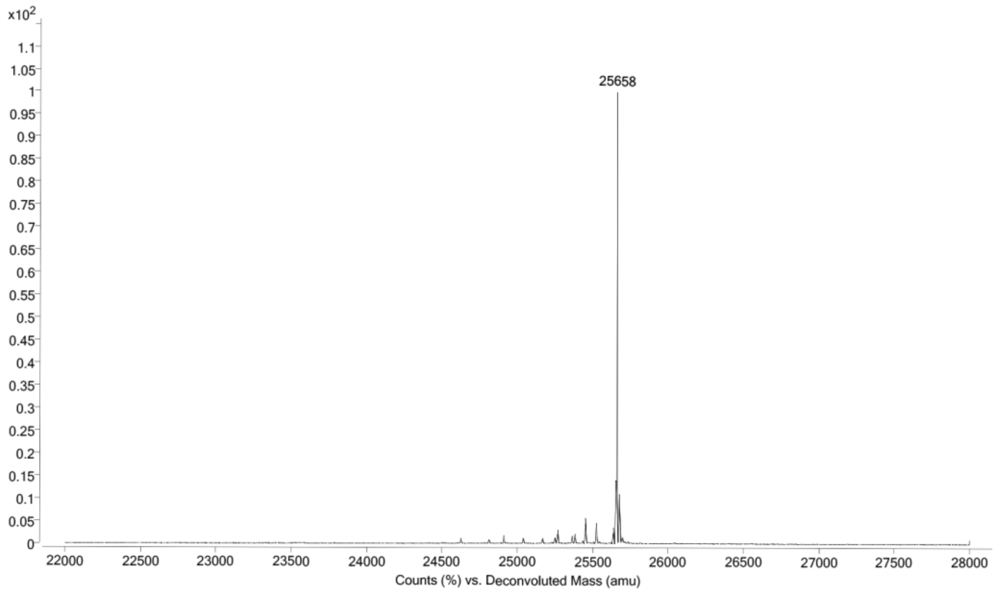

Deconvoluted MS spectra of unmodified α-chymotrpsinogen A

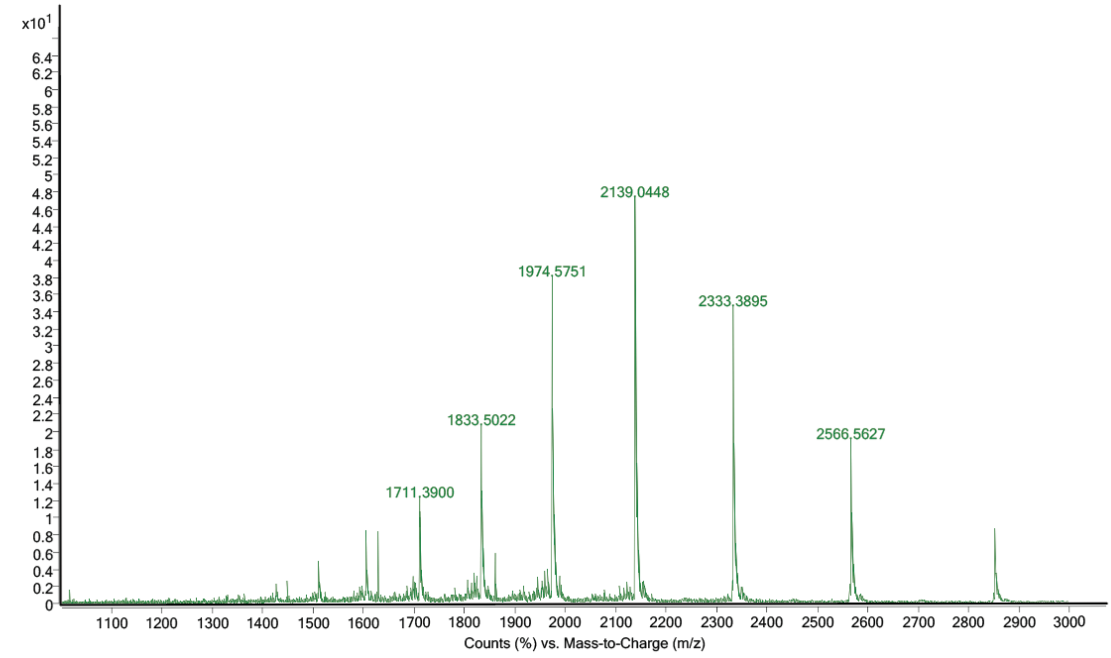

MS spectra of **1a** (50 equiv) modified α-chymotrpsinogen A shows no labelling

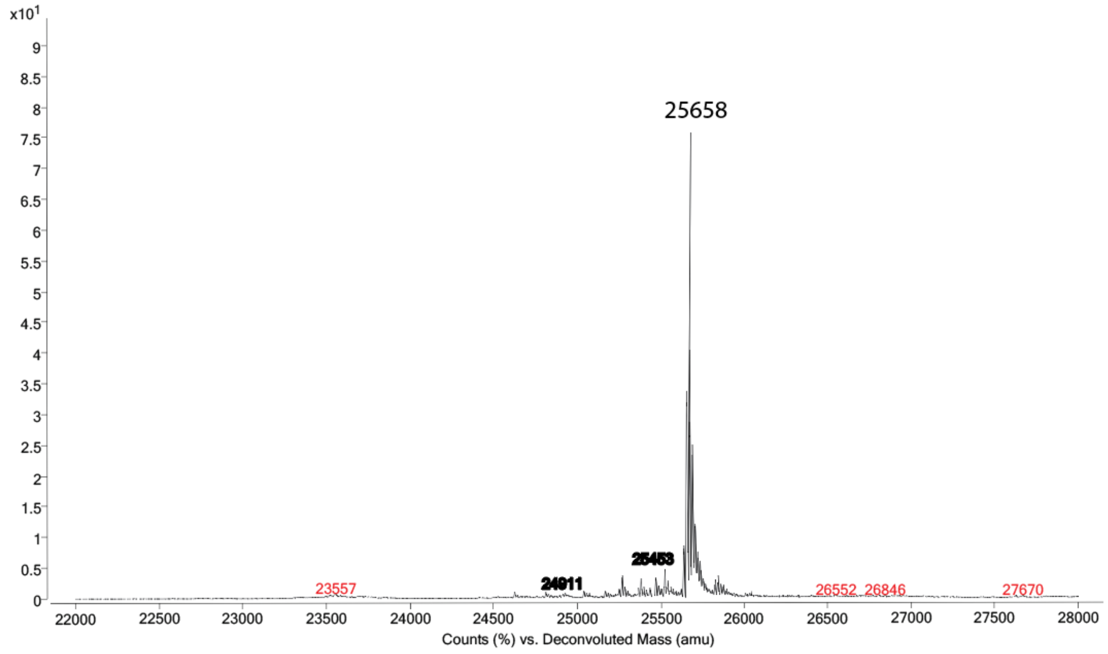

Deconvoluted MS spectra of **1a** (50 equiv) modified α-chymotrpsinogen A shows no labeling

MS/MS Analysis of 1a modified denatured α-chymotrpsinogen A:

Biomolecule 657: AMICAGASGVSSCMGDSGGPLVCK

Biomol	Seq Loc	Rule	Pred Mods	RT	Height	Mass	Tgt Mass	Diff (p
657	A(179-202)	complete digest, Predicted modifications	Oxidation (M) 2, Met-ChT- 14	9.217	6027	2384.9519	2384.9602	-

ECC (with sample chromatogram)

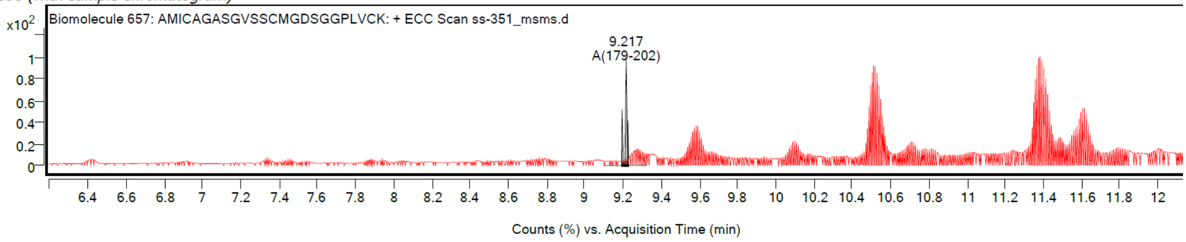

Mass Spectrum (with MFE spectrum, if available)

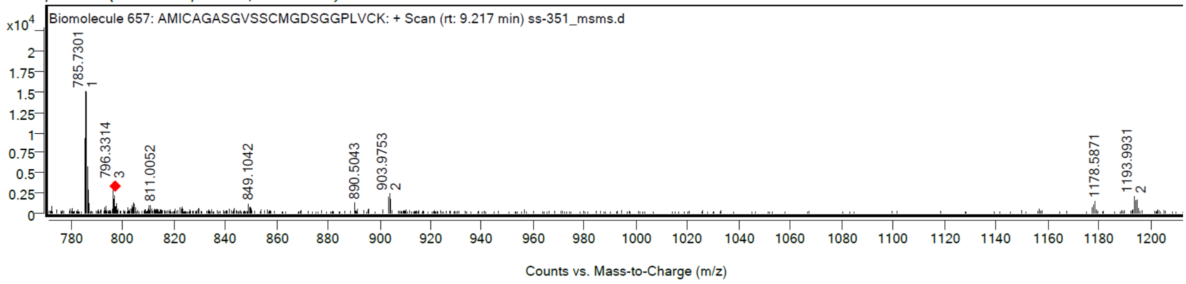

Identified Peptide Sequence: AMICAGASGVSSC**M**GDSGGPLVCK (AA179-AA202)

b^{+}	b^{++}		AA		y^{+}	y^{++}
72.044390	36.525833	1	A	24		
219.084875	110.046076	2	**M+O**	23	2314.935400	1157.971338
332.168939	166.588108	3	I	22	2167.894915	1084.451096
435.178123	218.092700	4	C	21	2054.810851	1027.909064
506.215237	253.611257	5	A	20	1951.801667	976.404472
563.236701	282.121989	6	G	19	1880.764553	940.885915
634.273815	317.640546	7	A	18	1823.743089	912.375183
721.305843	361.156560	8	S	17	1752.705975	876.856626
778.327307	389.667292	9	G	16	1665.673947	833.340612
877.395721	439.201499	10	V	15	1608.652483	804.829880
964.427749	482.717513	11	S	14	1509.584069	755.295673
1051.459778	526.233527	12	S	13	1422.552041	711.779659
1154.468962	577.738119	13	C	12	1335.520012	668.263644
1454.529197	727.768237	14	**M+Mod**	11	1232.510828	616.759052
1511.550661	756.278969	15	G	10	932.450593	466.728935
1626.577604	813.792440	16	D	9	875.429130	438.218203
1713.609632	857.308454	17	S	8	760.402186	380.704731
1770.631096	885.819186	18	G	7	673.370158	337.188717
1827.652560	914.329918	19	G	6	616.348694	308.677985
1924.705324	962.856300	20	P	5	559.327231	280.167254
2037.789388	1019.398332	21	L	4	462.274467	231.640872
2136.857801	1068.932539	22	V	3	349.190403	175.098840
2239.866986	1120.437131	23	C	2	250.121989	125.564633
		24	K	1	147.112804	74.060040

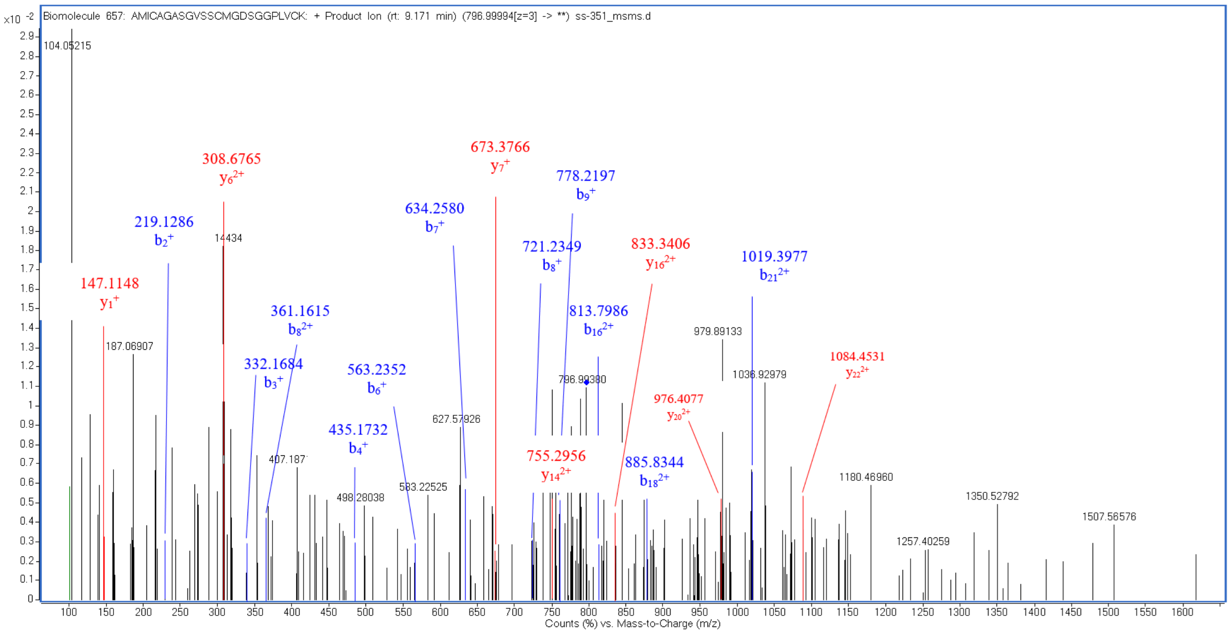

Supplementary Fig. 24. Labeling of methionine in Carbonic Anhydrase by CuNiP (PDB: 1V9E).

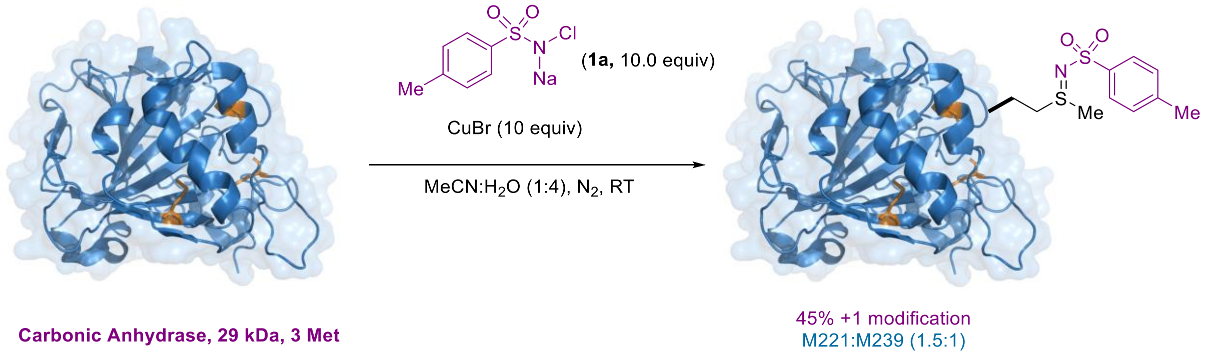

SHHWGYGKHNGPEHWHKDFPIANGERQSPVDIDTKAVVQDPALKPLALVYGEATSRR MVNNGHS
FNVEYDDSQDKAVLKDGPLTGTYRLVQFHFHWGSSDDQGSEHTVDRKKYAAELHLVHWNTKYG
DFGTAAQQPDGLAVVGVFLKVGDANPALQKVLDALDSIKTKGKSTDFPNFDPGSLLPNVLDYWTY
PGSLTTPPLLESVTWIVLKEPISVSSQQMLKFRTLNFNAEGEPELLMLANWRPAQPLKNRQVRGFPK

Carbonic anhydrase (3.5 mg, 0.12 µmol, 1.0 equiv) was dissolved in MeCN: H_2O (1:4, 800 µL) and CuBr (12 mM in MeCN, 100 µL, 1.2 µmol) and **1a** (12 mM in H_2O, 100 µL, 1.2 µmol) were added sequentially. The reaction mixture was incubated at 25 °C for 2 h under nitrogen atmosphere followed by the addition of 10 µL of 0.5 N HCl. The crude reaction mixture was passed through Amicon Ultra 3 kDa spin-concentrator and washed with H_2O (7×0.5 mL) to remove the small molecule impurities. This labeled protein was lyophilized, redissolved in 0.1% formic acid in H_2O and analyzed using LC-MS. The conversion was found to be >95%, and 45% of labeling carbonic anhydrase. MS/MS analysis of the digested protein shows labeling ratio of M221:M239 as 1.5:1.

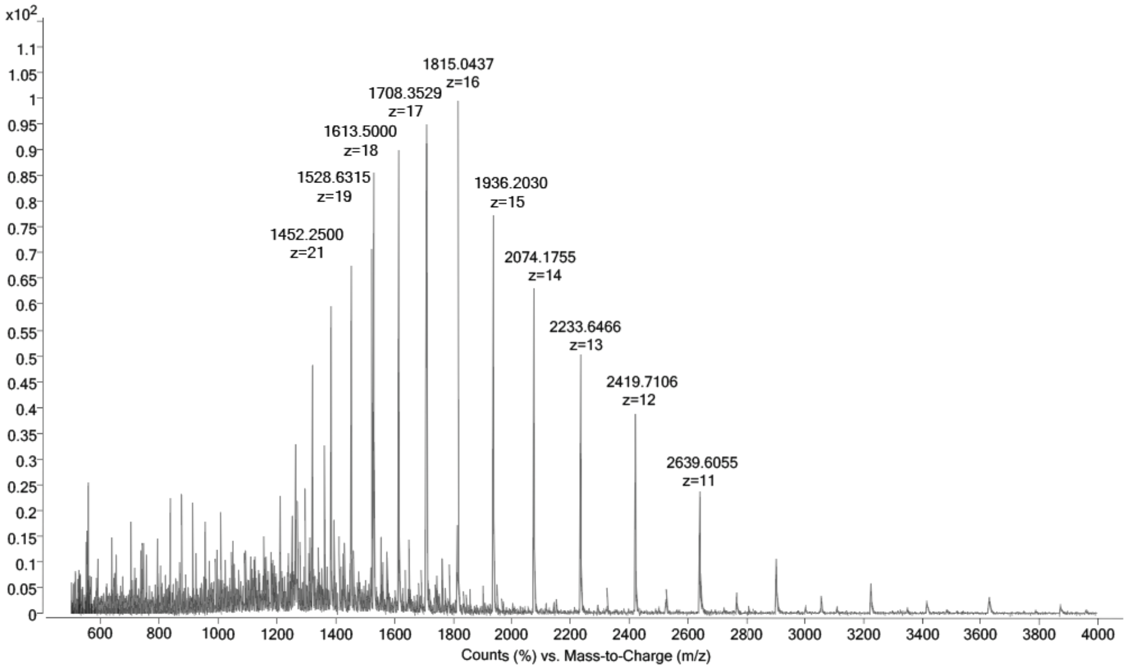

MS Spectra of unmodified carbonic anhydrase

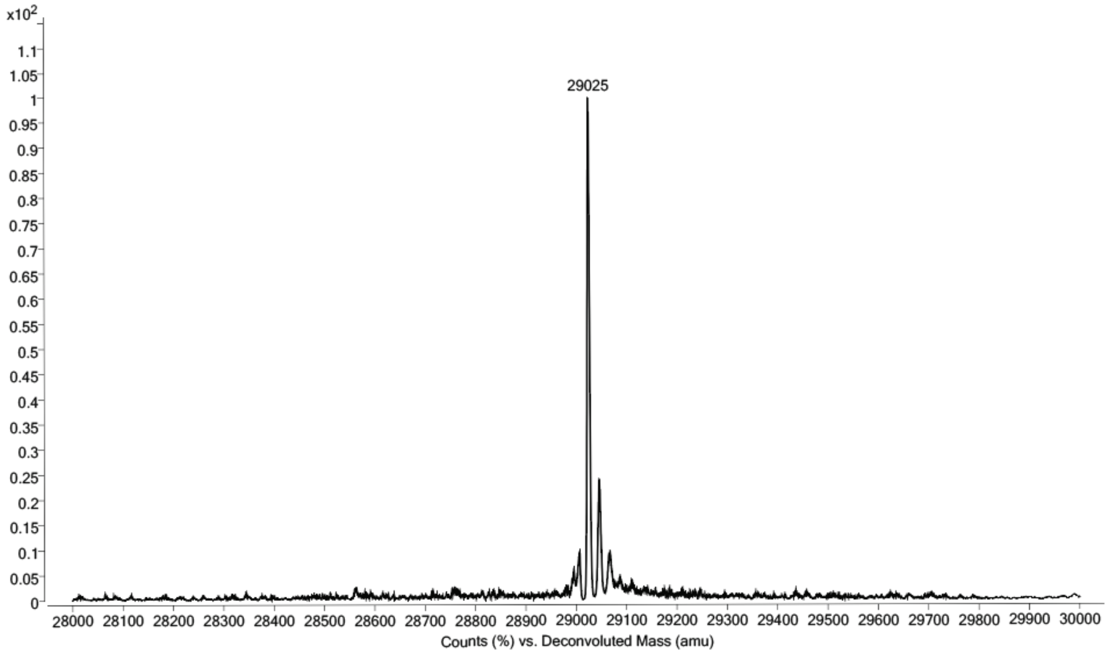

Deconvoluted MS spectra of unmodified carbonic anhydrase

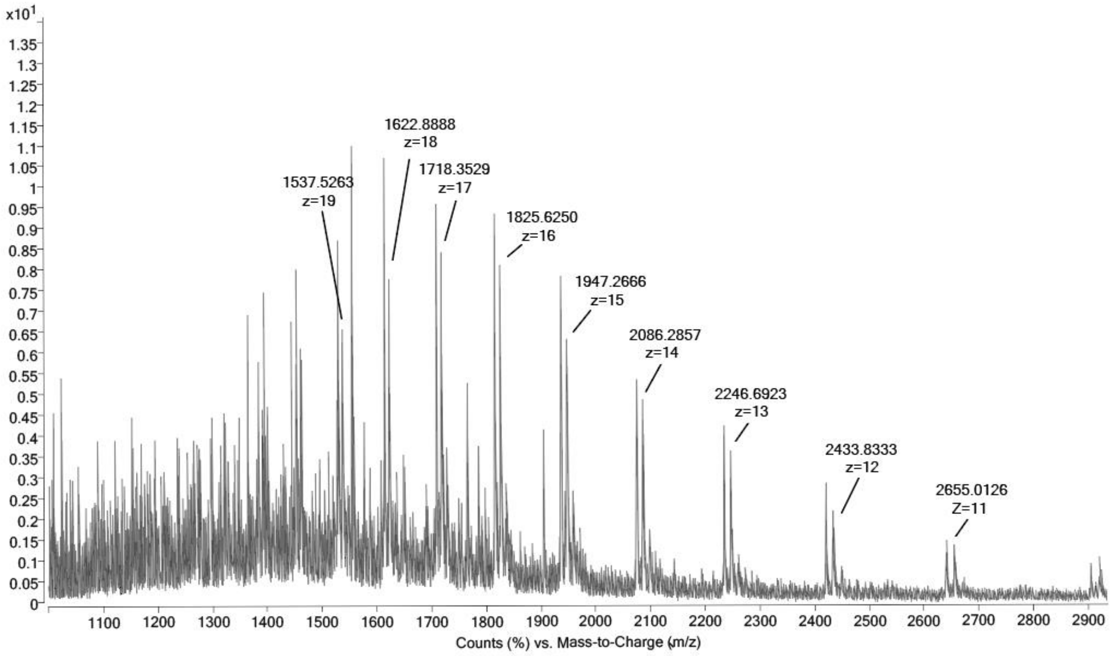

MS spectra of **1a** modified carbonic anhydrase

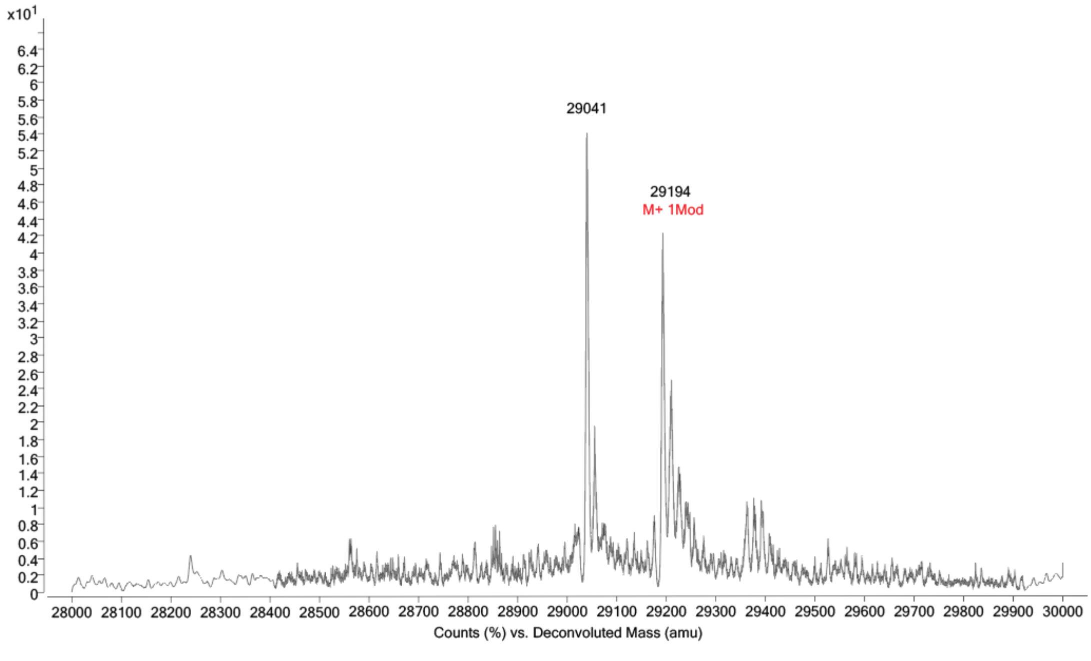

Deconvoluted MS spectra of **1a** modified carbonic anhydrase

MS/MS analysis of 1a modified carbonic anhydrase:

Biomolecule 55: EPISVSSQQMLK

Biomol	Seq Loc	Rule	Pred Mods	RT	Height	Mass	Tgt Mass	Diff (ppm)
55	A(212-223)	Complete digest, Predicted modifications	Met-ChT- 10	9.114	210490	1514.7178	1514.7109	4.55

ECC (with sample chromatogram)

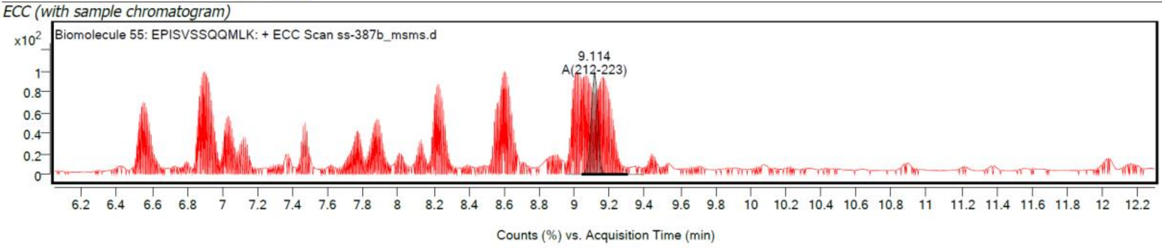

Mass Spectrum (with MFE spectrum, if available)

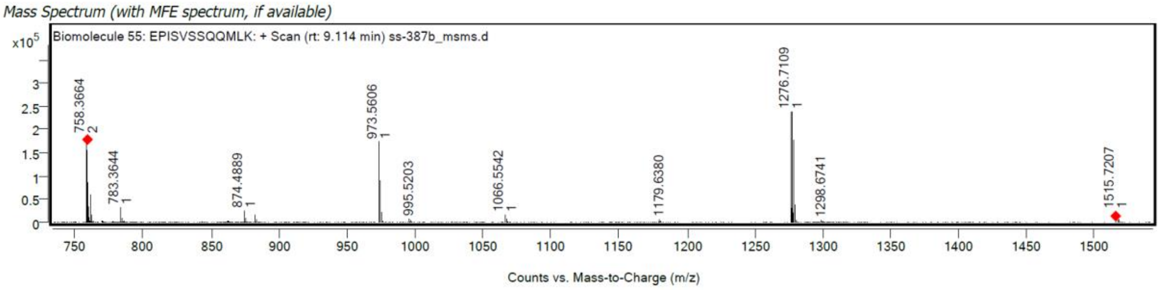

Identified Peptide Fragment Sequence: EPISVSSQQ**M**LK (AA212-AA223)

b^+	b^{++}		AA		y^+	y^{++}
130.049870	65.528573	1	E	12		
227.102633	114.054955	2	P	11	1386.675635	693.841456
340.186697	170.596987	3	I	10	1289.622871	645.315074
427.218726	214.113001	4	S	9	1176.538807	588.773042
526.287140	263.647208	5	V	8	1089.506779	545.257028
613.319168	307.163222	6	S	7	990.438365	495.722821
700.351197	350.679237	7	S	6	903.406336	452.206806
828.409774	414.708525	8	Q	5	816.374308	408.690792
956.468352	478.737814	9	Q	4	688.315730	344.661503
1256.528636	628.767956	10	**M+Mod**	3	560.257153	280.632215
1369.612700	685.309988	11	L	2	260.196868	130.602072
		12	K	1	147.112804	74.060040

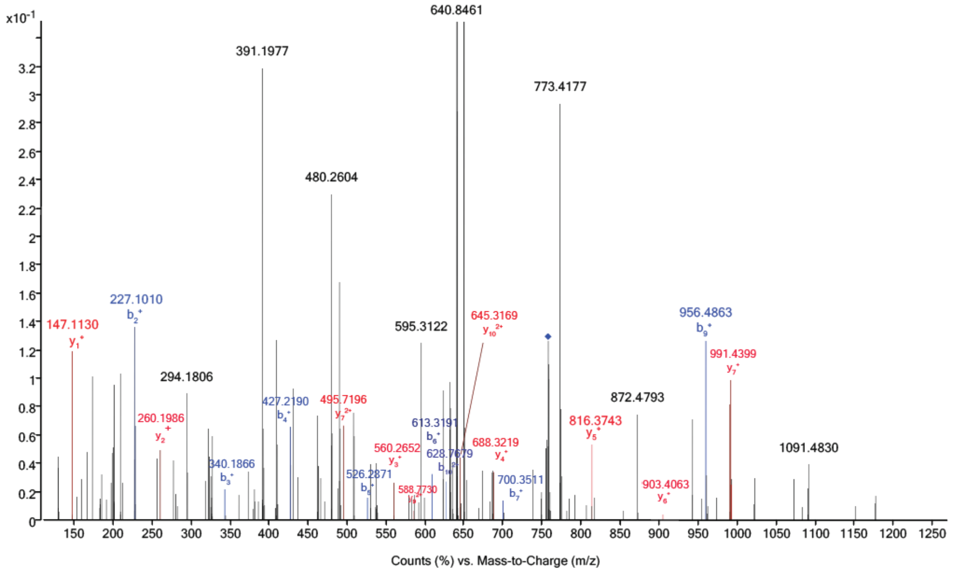

Biomolecule 69: TLNFNAEGEPELLMLANWRPAQPLK

Biomol	Seq Loc	Rule	Pred Mods	RT	Height	Mass	Tgt Mass	Diff (ppm)
69	A(226-250)	Complete digest, Predicted modifications	Met-ChT- 14	13.287	166798	3020.4938	3020.4888	1.63

ECC (with sample chromatogram)

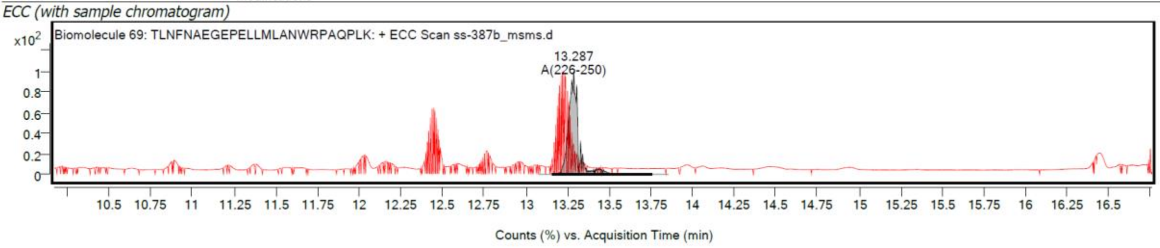

Mass Spectrum (with MFE spectrum, if available)

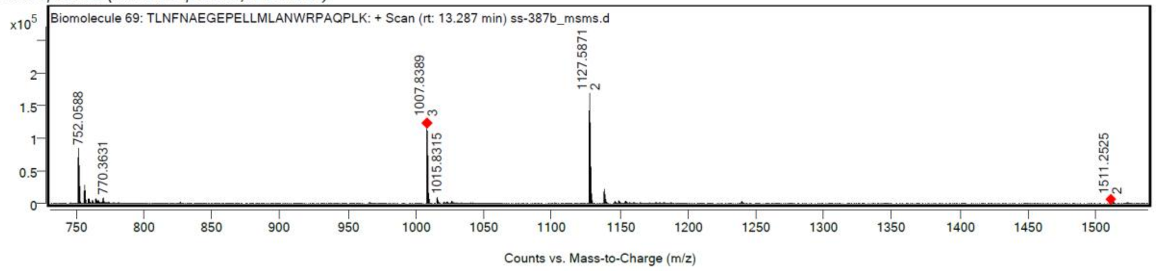

Identified Peptide Fragment Sequence: TLNFNAEGEPELL**M**LANWRPAQPLK (AA226-AA250)

b^+	b^{++}		AA		y^+	y^{++}
102.054955	51.531116	1	T	25		
215.139019	108.073148	2	L	24	2920.448483	1460.727880
329.181946	165.094611	3	N	23	2807.364419	1404.185848
476.250360	238.628818	4	F	22	2693.321492	1347.164384
590.293288	295.650282	5	N	21	2546.253078	1273.630177
661.330402	331.168839	6	A	20	2432.210150	1216.608713
790.372995	395.690136	7	E	19	2361.173036	1181.090156
847.394459	424.200868	8	G	18	2232.130443	1116.568860
976.437052	488.722164	9	E	17	2175.108979	1088.058128
1073.489816	537.248546	10	P	16	2046.066386	1023.536831
1202.532409	601.769843	11	E	15	1949.013622	975.010449
1315.616473	658.311875	12	L	14	1819.971029	910.489153
1428.700537	714.853907	13	L	13	1706.886965	853.947121
1728.760821	864.884049	14	**M+Mod**	12	1593.802901	797.405089
1841.844885	921.426081	15	L	11	1293.742617	647.374947
1912.881999	956.944638	16	A	10	1180.658553	590.832915
2026.924927	1013.966102	17	N	9	1109.621439	555.314358
2213.004240	1107.005758	18	W	8	995.578511	498.292894
2369.105351	1185.056314	19	R	7	809.499198	405.253237
2466.158115	1233.582696	20	P	6	653.398087	327.202682
2537.195228	1269.101252	21	A	5	556.345323	278.676300
2665.253806	1333.130541	22	Q	4	485.308210	243.157743
2762.306570	1381.656923	23	P	3	357.249632	179.128454
2875.390634	1438.198955	24	L	2	260.196868	130.602072
		25	K	1	147.112804	74.060040

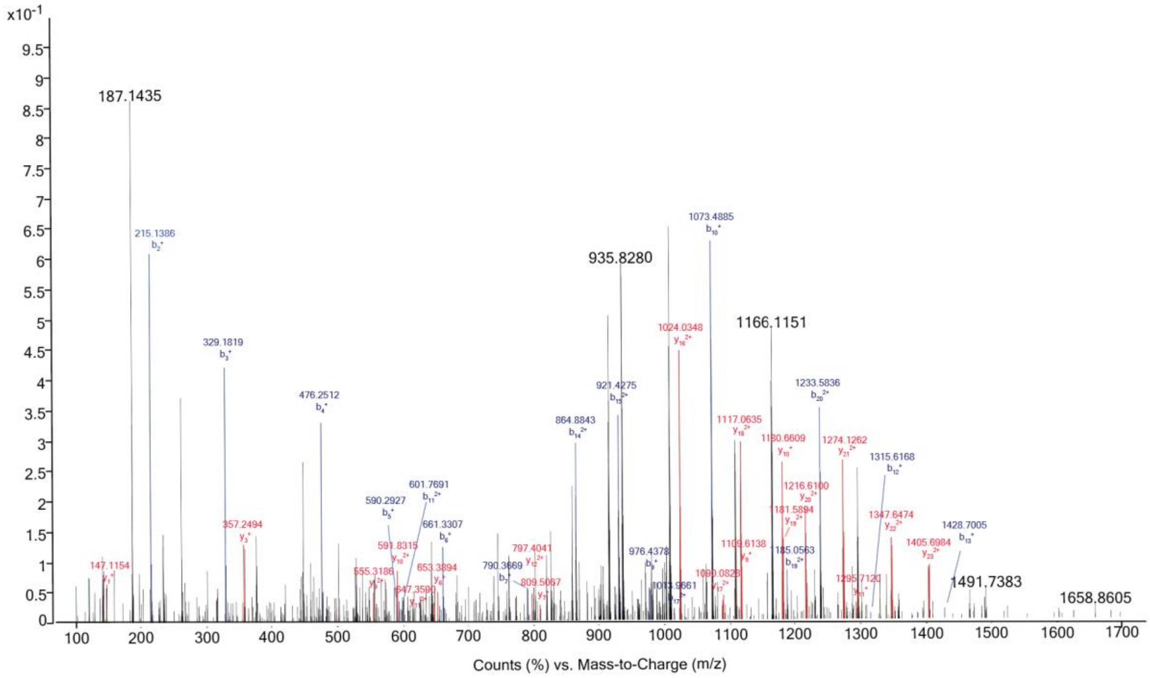

Supplementary Fig. 25. Labeling of methionine in Creatine Kinase by CuNiP (PDB: 2CRK).

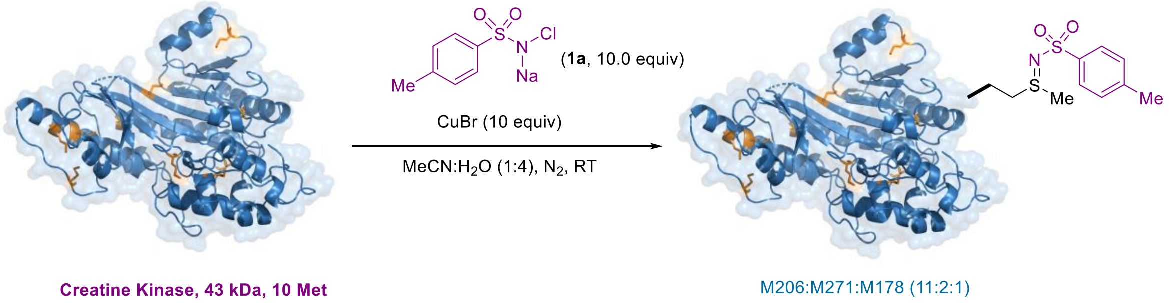

PFGNTHNKYKLNYKSEEEYPDLSKHNNHMAKVLTPDLYKKLRDKETPSGFTLDDVIQTGVDNP
GHPFIMTVGCVAGDEESYTVFKDLFDPIIQDRHGGFKPTDKHKTDLNHENLKGGDDLDPHYVLSS
RVRTGRSIKGYTLPPHCSRGERRAVEKLSVEALNSLTGEFKGKYYPLKS MTEQEQQQLIDDHFLFD
KPVSPLLLASGMARDWPDARGIWHNDNKSFLVWVNEEDHLRVIS MEKGGNMKEVFRRFCVGLQ
KIEEIFKKAGHPFMWNEHLGYVLTCPSNLGTGLRGGVHVKLAHLSKHPKFEEILTRLRLQKRGTGG
VDTAAVGSVFDISNADRLGSSEVEQVQLVVDGVKL MVEMEKKLEKGQSIDDMIPAQK

Creatine-kinase (5.15 mg, 0.12 µmol, 1.0 equiv) was dissolved in MeCN: H_2O (1:4, 800 µL) and CuBr (12 mM in MeCN, 100 µL, 1.2 µmol), **1a** (12 mM in H_2O, 100 µL, 1.2 µmol) were added sequentially. The reaction mixture was incubated at 25 °C for 2 h under nitrogen atmosphere followed by the addition of 10 µL of 0.5 N HCl. The crude reaction mixture was passed through Amicon Ultra 3 kDa spin-concentrator and washed with H_2O (7×0.5 mL) to remove the small molecule impurities. The labeled protein was digested and MS/MS analysis shows labeling ratio of M206:M271:M178 as 11:2:1.

MS/MS Analysis of 1a modified creatine kinase:

Biomolecule 60: PVSPLLLASGMAR

Biomol	Seq Loc	Rule	Pred Mods	RT	Height	Mass	Tgt Mass	Diff (ppm)
60	A(196-208)	Complete digest, Predicted modifications	Met-ChT- 11	12.032	282032	1479.7625	1479.7578	3.21

ECC (with sample chromatogram)

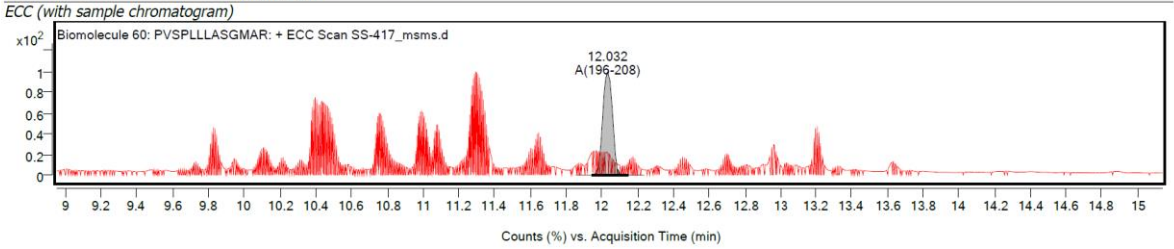

Mass Spectrum (with MFE spectrum, if available)

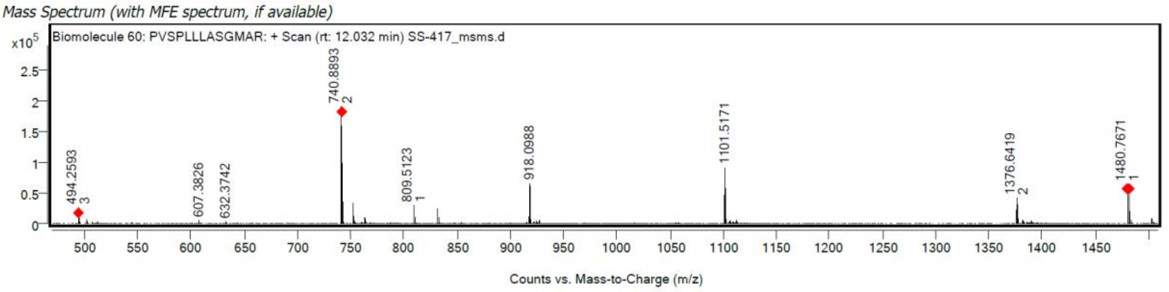

Identified Peptide Sequence: PVSPLLLASG**M**AR (AA196-AA208)

b+	b++		AA		y+	y++
98.060040	49.533658	1	P	13		
197.128454	99.067865	2	V	12	1383.712355	692.359816
284.160483	142.583880	3	S	11	1284.643941	642.825609
381.213247	191.110262	4	P	10	1197.611913	599.309595
494.297311	247.652294	5	L	9	1100.559149	550.783213
607.381375	304.194326	6	L	8	987.475085	494.241181
720.465439	360.736358	7	L	7	874.391021	437.699149
791.502552	396.254914	8	A	6	761.306957	381.157117
878.534581	439.770929	9	S	5	690.269843	345.638560
935.556045	468.281661	10	G	4	603.237814	302.122545
1235.616329	618.311803	11	**M+Mod**	3	546.216351	273.611814
1306.653443	653.830360	12	A	2	246.156066	123.581671
		13	R	1	175.118952	88.063114

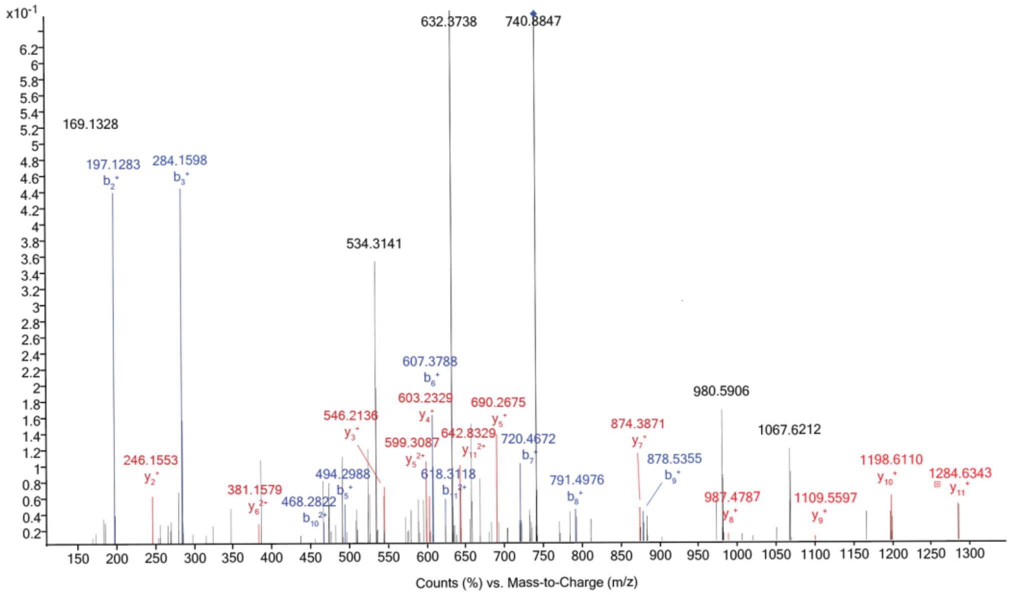

Biomolecule 185: AGHPFMWNEHLGYVLTCPSNLGTGL

Biomol	Seq Loc	Rule	Pred Mods	RT	Height	Mass	Tgt Mass	Diff (ppm)
185	A(266-290)	Protein truncation, Predicted modifications	Met-ChT- 6, Alkylation (iodoacetamide) 17	11.656	71957	2939.3430	2939.3193	8.05

ECC (with sample chromatogram)

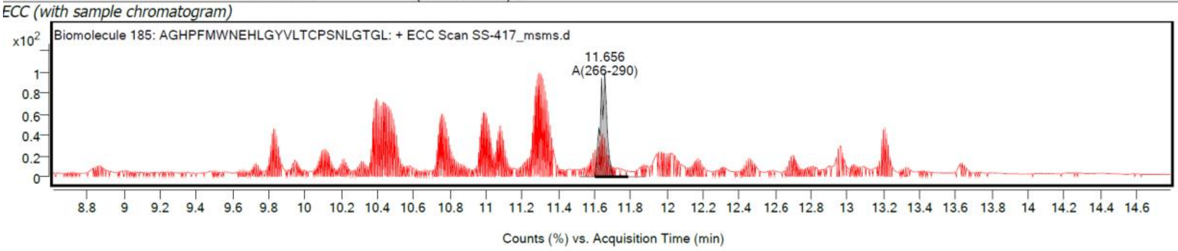

Mass Spectrum (with MFE spectrum, if available)

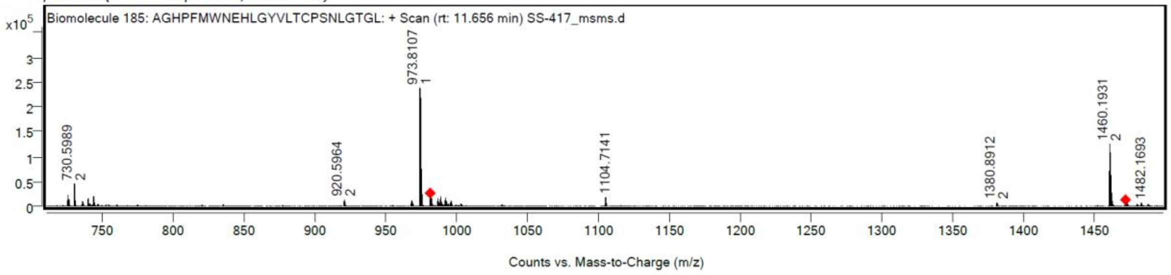

Identified Peptide Sequence: AGHPF**M**WNEHLGYVLTCPSNLGTGL (AA266-AA290)

b^{+}	b^{++}		AA		y^{+}	y^{++}
72.044390	36.525833	1	A	25		
129.065854	65.036565	2	G	24	2869.289576	1435.148426
266.124766	133.566021	3	H	23	2812.268112	1406.637694
363.177530	182.092403	4	P	22	2675.209200	1338.108238
510.245944	255.626610	5	F	21	2578.156436	1289.581856
810.306228	405.656752	6	**M+Mod**	20	2431.088022	1216.047649
996.385541	498.696409	7	W	19	2131.027738	1066.017507
1110.428469	555.717873	8	N	18	1944.948425	972.977851
1239.471062	620.239169	9	E	17	1830.905497	915.956387
1376.529974	688.768625	10	H	16	1701.862904	851.435090
1489.614038	745.310657	11	L	15	1564.803992	782.905634
1546.635502	773.821389	12	G	14	1451.719928	726.363602
1709.698830	855.353053	13	Y	13	1394.698465	697.852870
1808.767244	904.887260	14	V	12	1231.635136	616.321206
1921.851308	961.429292	15	L	11	1132.566722	566.786999
2022.898987	1011.953132	16	T	10	1019.482658	510.244967
2182.929671	1091.968474	17	**C+IAA**	9	918.434979	459.721128
2279.982435	1140.494856	18	P	8	758.404295	379.705786
2367.014463	1184.010870	19	S	7	661.351531	331.179404
2481.057391	1241.032334	20	N	6	574.319503	287.663390
2594.141455	1297.574366	21	L	5	460.276575	230.641926
2651.162919	1326.085098	22	G	4	347.192511	174.099894
2752.210597	1376.608937	23	T	3	290.171047	145.589162
2809.232061	1405.119669	24	G	2	189.123369	95.065323
		25	L	1	132.101905	66.554591

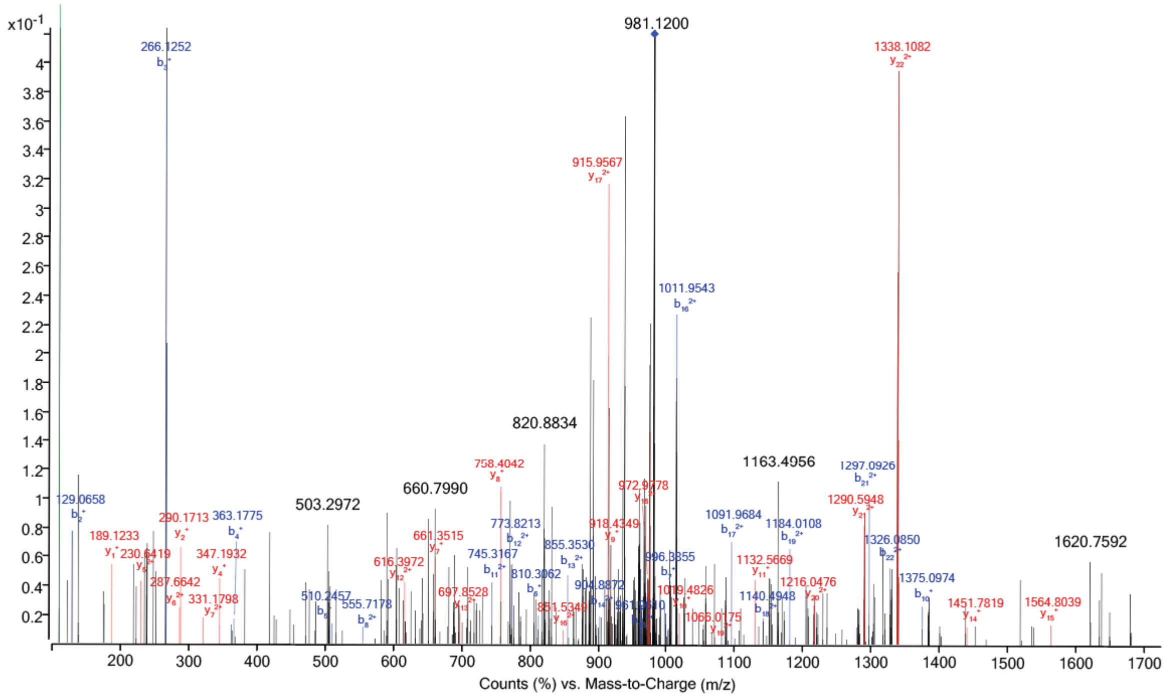

Biomolecule 236: SMTEQEQQQLIDDHFLFDK

Biomol	Seq Loc	Rule	Pred Mods	RT	Height	Mass	Tgt Mass	Diff (ppm)
236	A(177-195)	Complete digest, Predicted modifications	Met-ChT- 2	11.116	27434	2520.0912	2520.0937	-1.02

ECC (with sample chromatogram)

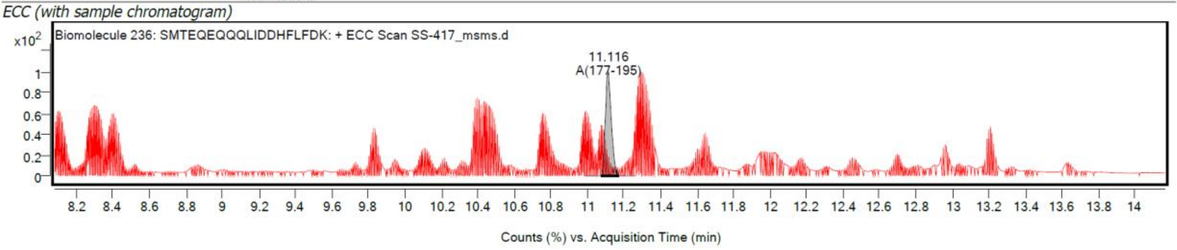

Mass Spectrum (with MFE spectrum, if available)

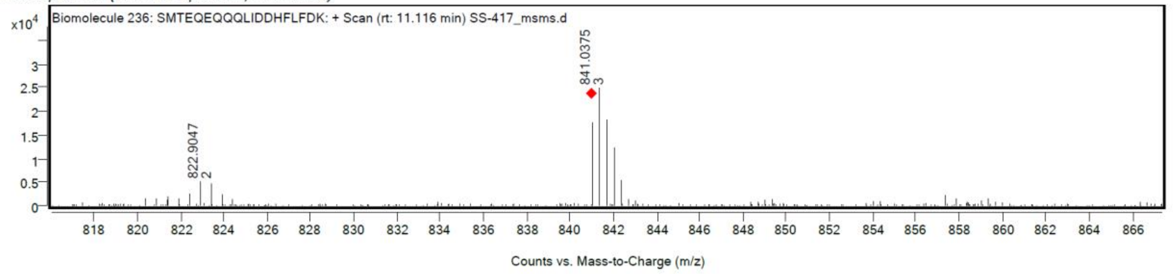

Identified Peptide Sequence: SMTEQEQQQLIDDHFLFDK (AA177-AA195)

b^{+}	b^{++}	AA			y^{+}	y^{++}
88.039305	44.523291	1	S	19		
388.099590	194.553433	2	**M+Mod**	18	2434.069025	1217.538151
489.147268	245.077272	3	T	17	2134.008740	1067.508008
618.189861	309.598569	4	E	16	2032.961062	1016.984169
746.248439	373.627858	5	Q	15	1903.918469	952.462873
875.291032	438.149154	6	E	14	1775.859891	888.433584
1003.349609	502.178443	7	Q	13	1646.817298	823.912287
1131.408187	566.207732	8	Q	12	1518.758720	759.882998
1259.466764	630.237020	9	Q	11	1390.700143	695.853710
1372.550829	686.779052	10	L	10	1262.641565	631.824421

1485.634893	743.321084	11	I	9	1149.557501	575.282389
1600.661836	800.834556	12	D	8	1036.473437	518.740357
1715.688779	858.348028	13	D	7	921.446494	461.226885
1852.747691	926.877484	14	H	6	806.419551	403.713414
1999.816104	1000.411690	15	F	5	669.360639	335.183958
2112.900169	1056.953722	16	L	4	522.292225	261.649751
2259.968582	1130.487929	17	F	3	409.208161	205.107719
2374.995526	1188.001401	18	D	2	262.139747	131.573512
		19	K	1	147.112804	74.060040

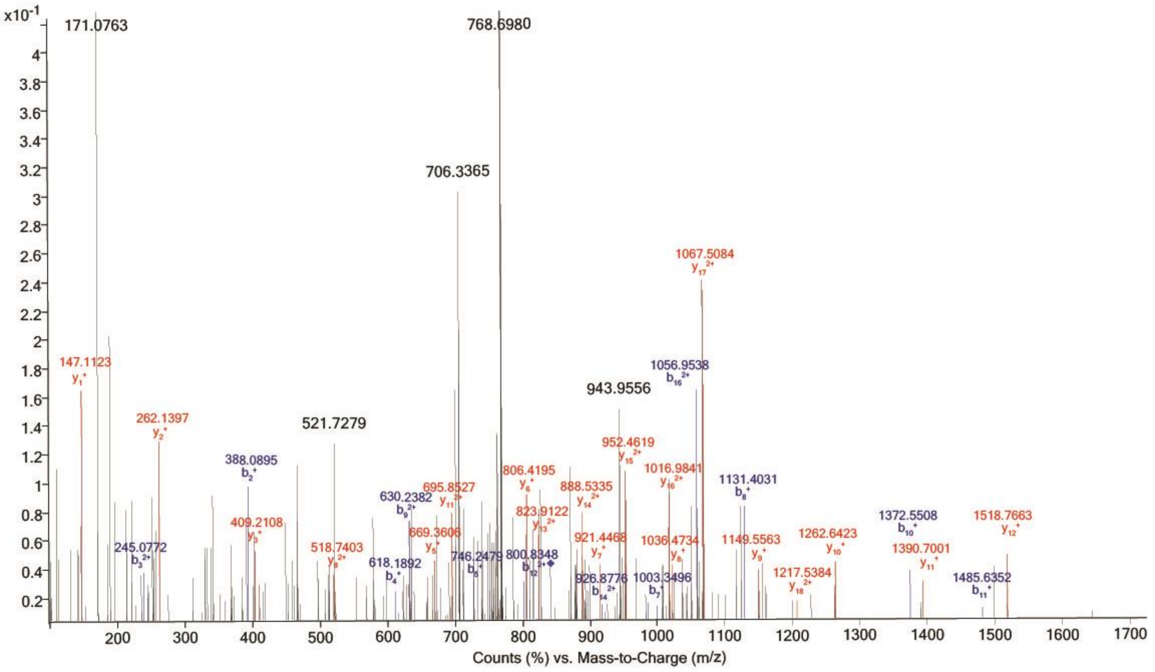

Supplementary Fig. 26: Labeling of methionine in Bovine Serum Albumin by CuNiP (PDB: 3V03).

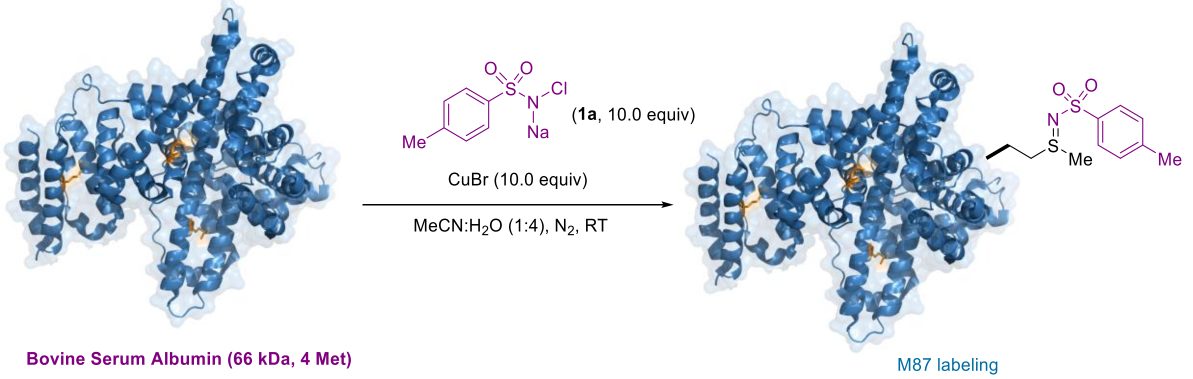

DTHKSEIAHRFKDLGEEHFKGLVLIAFSQYLQQCPFDEHVKLVNELTEFAKTCVADESHAGCEKSLHTLFG
DELCKVASLRETYGDMADCCEKQEPERNECFLSHKDDSPDLPKLKPDPNTLCDEFKADEKKFWGKYLY
EIARRHPYFYAPELLYYANKYNGVFQECCQAEDKGACLLPKIET MREKVLTSSARQRLRCASIQKFGER
ALKAWSVARLSQKFPKAEFVEVTKLVTDLTKVHKECCHGDLLECADDRADLAKYICDNQDTISSKLKE
CCDKPLLEKSHCIAEVEKDAIPENLPPLTADFAEDKDVCKNYQEAKDAFLGSFLYEYSRRHPEYAVSVL
LRLAKEYEATLEECCAKDDPHACYSTVFDKLKHLVDEPQNLIKQNCDQFEKLGEYGFQNALIVRYTRK
VPQVSTPTLVEVSRSLGKVGTRCCTKPESERMPCTEDYLSLILNRLCVLHEKTPVSEKVTKCCTESLVNR
RPCFSALTPDETYVPKAFDEKLFTFHADICTLPDTEKQIKKQTALVELLKHKPKATEEQLKTV MENFVA
FVDKCCAADDKEACFAVEGPKLVVSTQTALA

Bovine serum albumin (7.92 mg, 0.12 µmol, 1.0 equiv) was dissolved in MeCN: H_2O (1:4, 800 µL) and CuBr (12 mM in MeCN, 100 µL, 1.2 µmol), **1a** (12 mM in H_2O, 100 µL, 1.2 µmol) were added sequentially. The reaction mixture was incubated at 25 °C for 2 h under nitrogen atmosphere followed by the addition of 10 µL of 0.5 N HCl. The crude reaction mixture was passed through Amicon Ultra 3 kDa spin-concentrator and washed with H_2O (7×0.5 mL) to remove the small molecule impurities. The labeled protein was digested and MS/MS analysis shows exclusive labeling of M87.

MS/MS analysis of 1a modified bovine serum albumin:

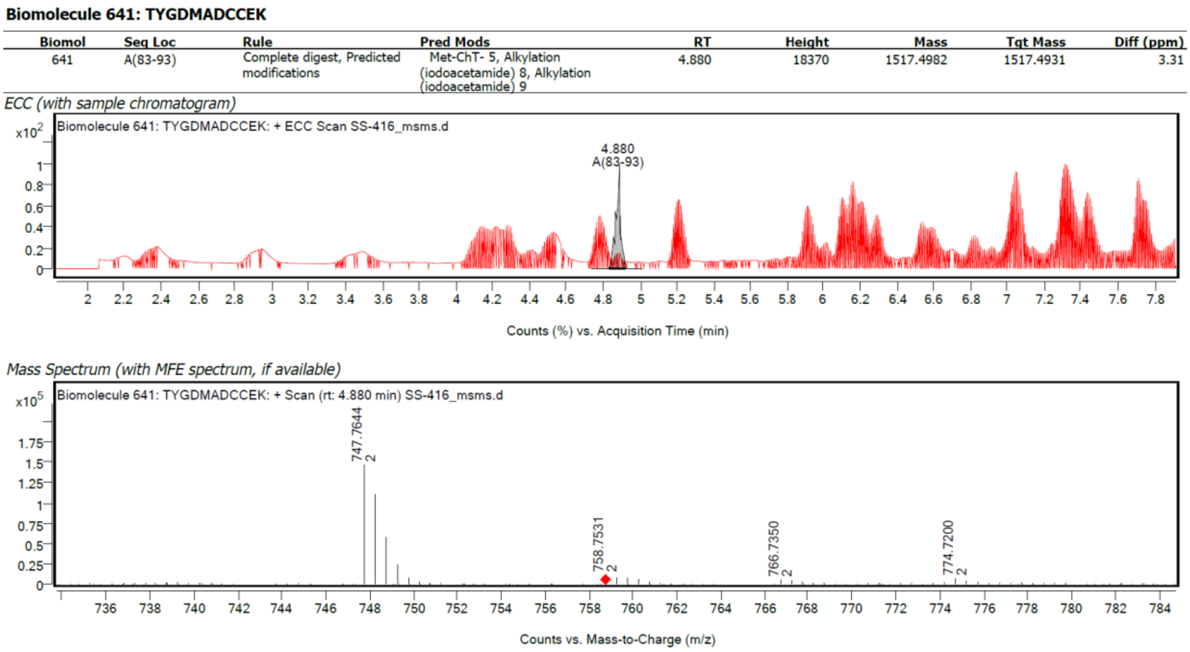

Identified Peptide Sequence: TYGD**M**ADCCEK (AA83-AA93)

b^+	b^{++}		AA		y^+	y^{++}
102.054955	51.531116	1	T	11		
265.118284	133.062780	2	Y	10	1417.452843	709.230060
322.139747	161.573512	3	G	9	1254.389515	627.698396
437.166690	219.086983	4	D	8	1197.368051	599.187664
737.226975	369.117126	5	**M+mod**	7	1082.341108	541.674192
808.264089	404.635683	6	A	6	782.280823	391.644050
923.291032	462.149154	7	D	5	711.243709	356.125493
1083.321716	542.164496	8	C	4	596.216766	298.612021
1243.352401	622.179839	9	C	3	436.186082	218.596679
1372.394994	686.701135	10	E	2	276.155397	138.581337
		11	K	1	147.112804	74.060040

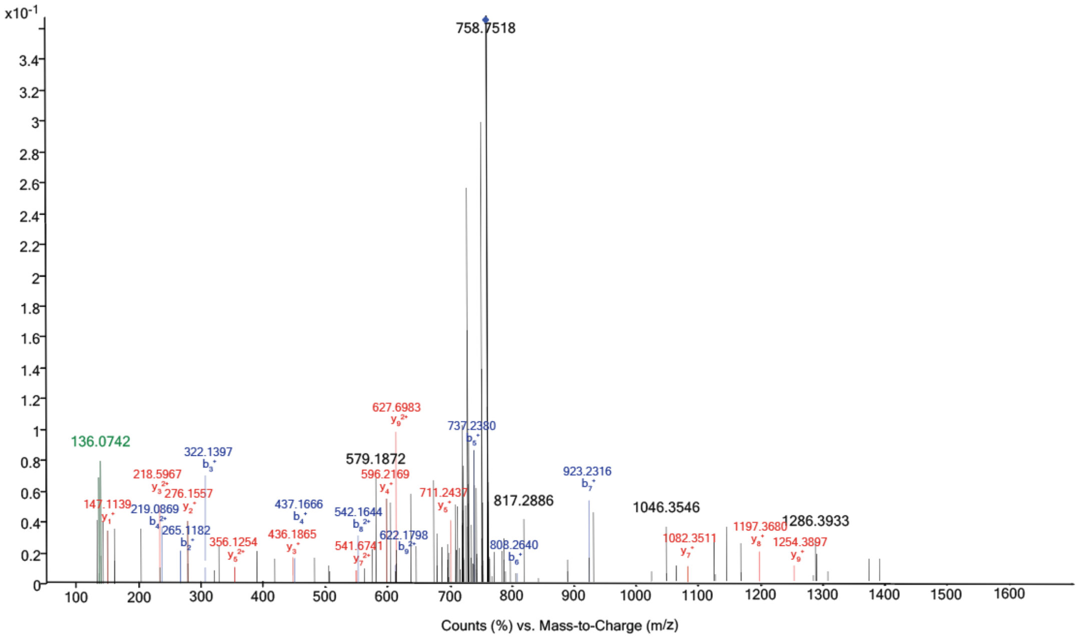

Supplementary Fig. 27. Labeling of methionine in Apo-Transferrin by CuNiP (PDB: 3V8X).

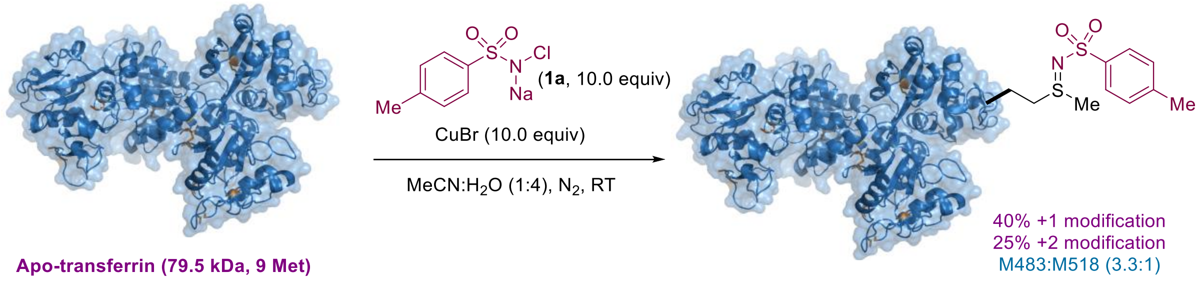

MRLAVGALLVCAVLGLCLAVPDKTVRWCAVSEHEATKCQSFRDH MKSVIPSDGPSVACVKKASYLDCIRAIAANEADAVTLDAGLVYDA
YLAPNNLKPVVAEFYGSKEDPQTFYYAVAVVKKDSGFQ MNQLRGKKSCHTGLGRSAGWNIPIGLLYCDLPEPRKPLEKAVANFFSGSCA
PCADGTDFPQLCQLCPGCGCSTLNQYFGYSGAFKCLKDGAGDVAFVKHSTIFENLANKADRDQYELLCLDNTRKPVDEYKDCHLAQVP
SHTVVARSMGGKEDLIWELLNQAQEHFGKDKSKEFQLFSSPHGKDLLFKDSAHGFLKVPPRMDAKMYLGYEYVTAIRNLREGTCPEAP
TDECKPVKWCALSHHERLKCDEWSVNSVGKIECVSAETTEDCIAKIMNGEADASLDGGFVYIAGKCGLVPVLAENYNKSDNCEDTPEA
GYFAIAVVKKSASDLTWDNLKGKKSCHTAVGRTAGWNIP MGLLYNKINHCRFDEFFSEGCAPGSKKDSSLCKLCMGSGLNLCEPNNK
EGYYGYTGAFRCLVEKGDVAFVKHQTVPQNTGGKNPDPWAKNLNEKDYELLCLDGTRKPVEEYANCHLARAPNHAVVTRKDKEACV
HKILRQQQHLFGSNVTDCSGNFCLFRSETKDLLFRDDTVCLAKLHDRNTYEKYLGEEYVKAVGNLRKCSTSSLLEACTFRRP

Apo-transferrin (9.5 mg, 0.12 μmol, 1.0 equiv) was dissolved in MeCN: H_2O (1:4, 800 μL) and CuBr (12 mM in MeCN, 100 μL, 1.2 μmol), **1a** (12 mM in H_2O, 100 μL, 1.2 μmol) were added sequentially. The reaction mixture was incubated at 25 °C for 2 h under nitrogen atmosphere followed by the addition of 10 μL of 0.5 N HCl. The crude reaction mixture was passed through Amicon Ultra 3 kDa spin-concentrator and washed with H_2O (7×0.5 mL) to remove the small molecule impurities. This labeled protein was lyophilized, redissolved in 0.1% formic acid in H_2O and analyzed using LC-MS. The conversion was found to be >95%. Intact mass analysis shows 40% +1 modification and 25% +2 modification. MS/MS analysis of the digested protein shows labeling ratio of M483:M518 is 3.3:1.

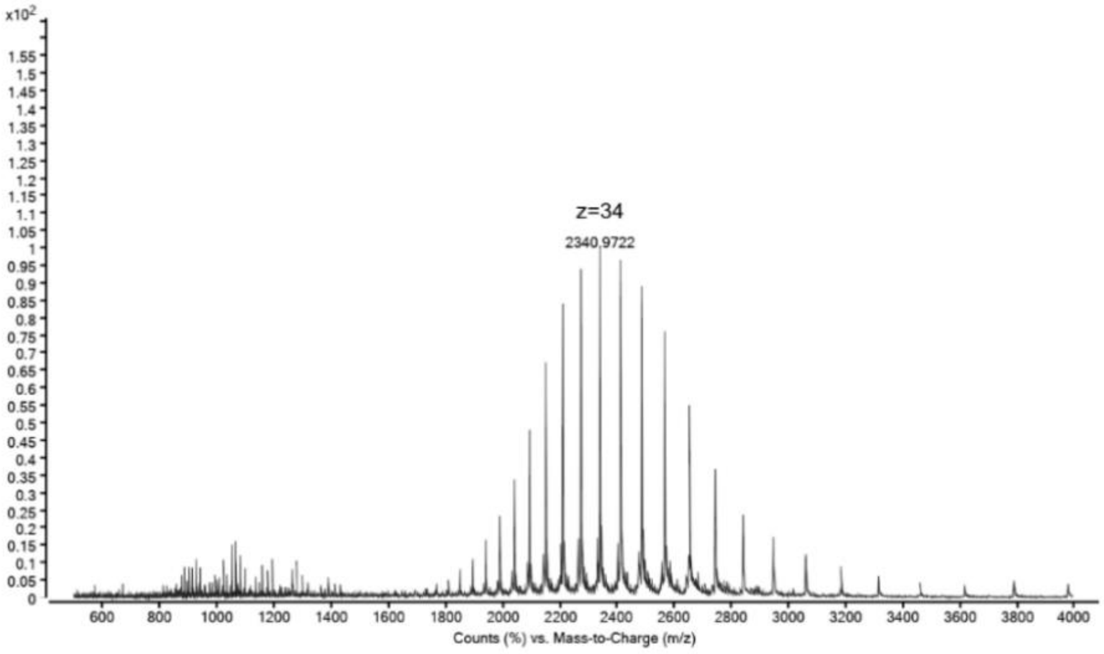

MS spectra of unmodified apo-transferrin

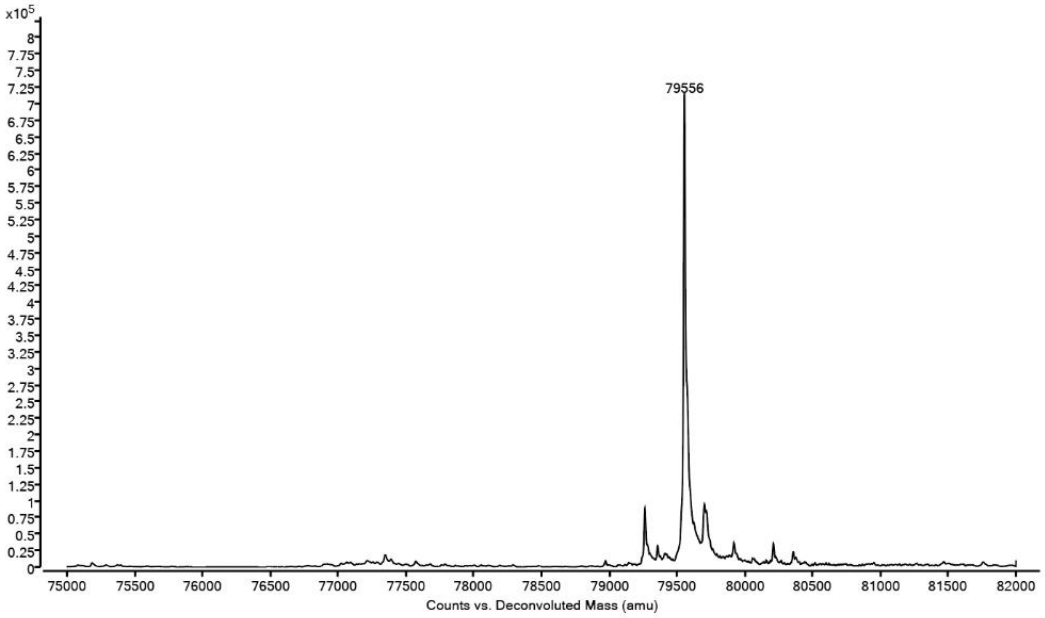

Deconvoluted MS spectra of unmodified apo-transferrin

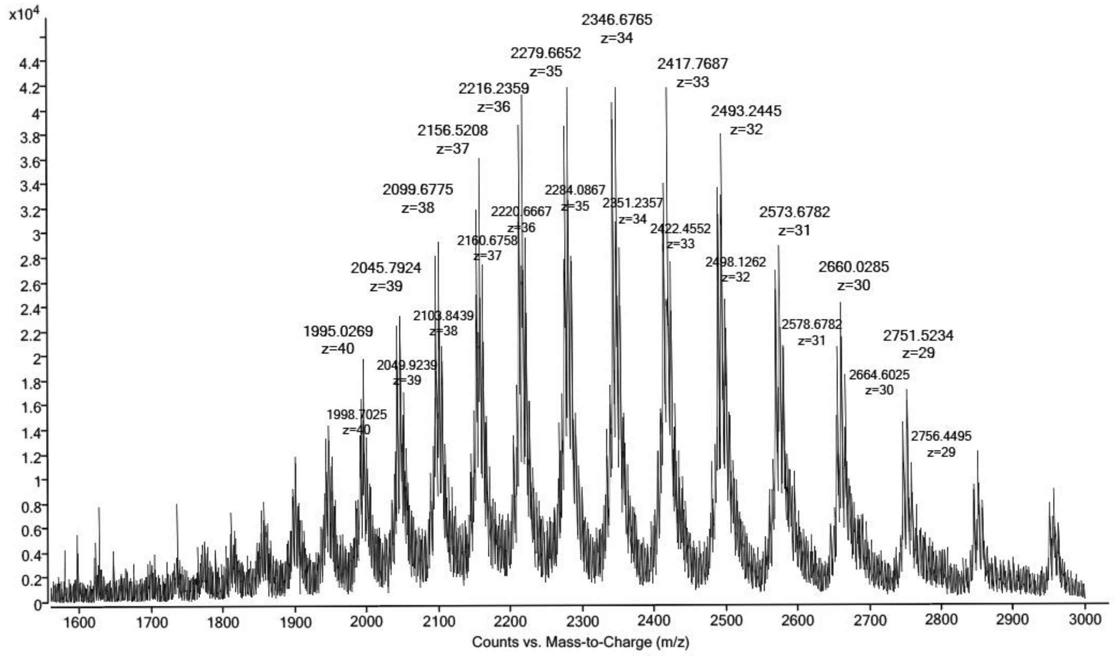

MS-Spectra of **1a** modified apo-transferrin

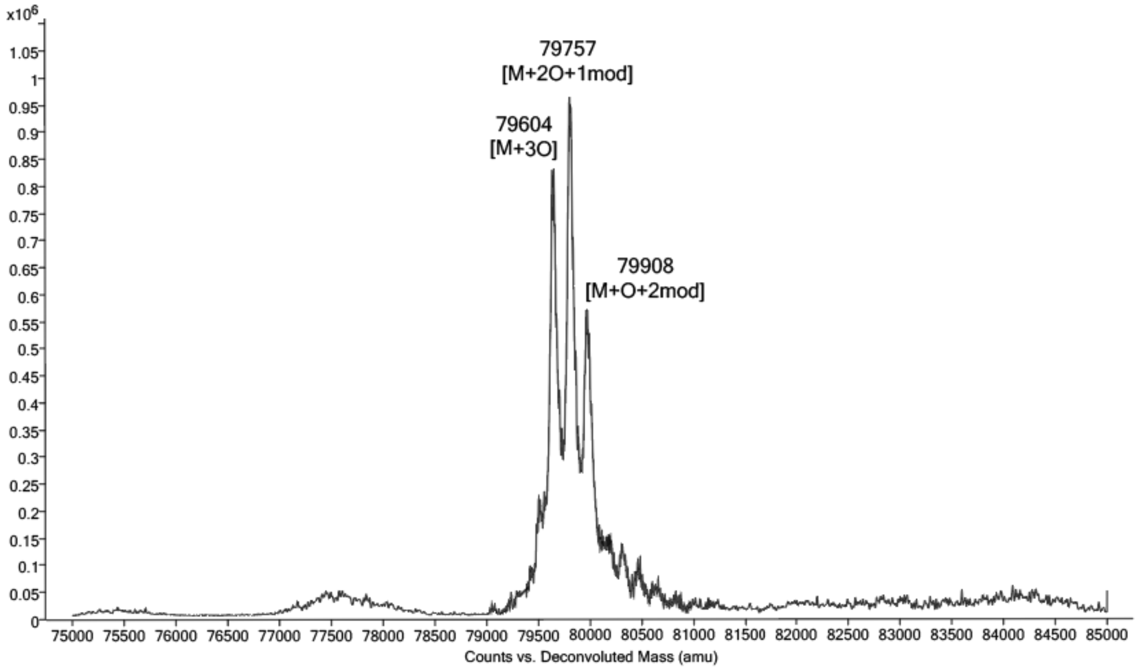

Deconvoluted Spectra of **1a** modified apo-transferrin

MS/MS Analysis of 1a modified Aprotinin

Biomolecule 193: TAGWNIPMGLLYNK

Biomol	Seq Loc	Rule	Pred Mods	RT	Height	Mass	Tgt Mass	Diff (p
193	A(476-489)	Complete digest, Predicted modifications	Met-ChT- 8	12.567	22622	1745.8260	1745.8269	

ECC (with sample chromatogram)

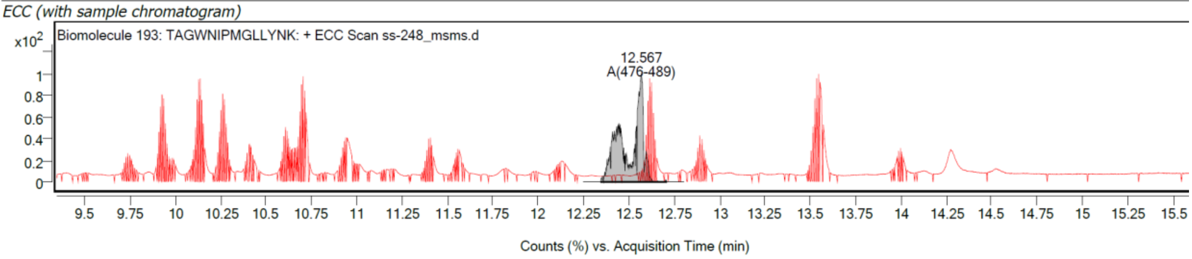

Mass Spectrum (with MFE spectrum, if available)

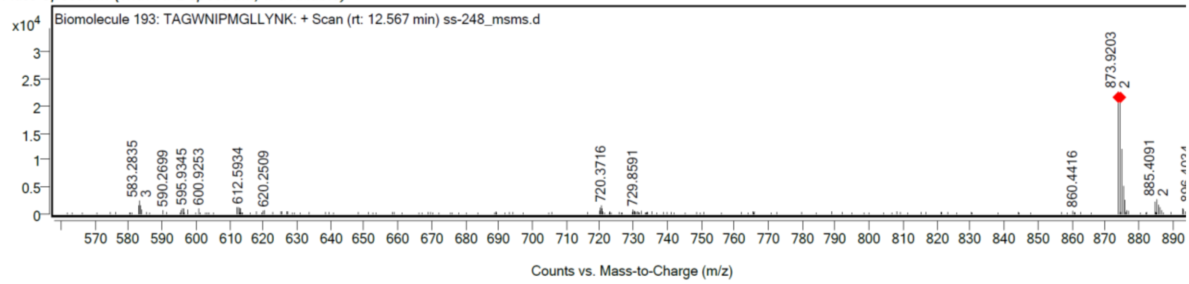

Identified Peptide Sequence: TAGWNIPMGLLYNK (AA476-AA489)

b+	b2+	b3+		AA		y+	y2+	y3+
102.054955	51.531116	34.689836	1	T	14			
173.092069	87.049673	58.368874	2	A	13	1645.786533	823.396905	549.267029
230.113533	115.560404	77.376028	3	G	12	1574.749419	787.878348	525.587991
416.192845	208.600061	139.402466	4	W	11	1517.727955	759.367616	506.580836
530.235773	265.621525	177.416775	5	N	10	1333.648642	666.327959	444.554398
643.319837	322.163557	215.111463	6	I	9	1217.605715	609.306496	406.540089
740.372601	370.689939	247.462385	7	P	8	1104.521651	552.764464	368.845401
1040.432836	520.720056	347.482463	8	M+mod	7	1007.468887	504.238082	336.494480
1097.454299	549.230788	366.489617	9	G	6	707.408652	354.207964	236.474402
1210.538363	605.772820	404.184305	10	L	5	650.387188	325.697232	217.467247
1323.622427	662.314852	441.878993	11	L	4	537.303124	269.155200	179.772559
1486.685756	743.846516	496.233436	12	Y	3	424.219060	212.613168	142.077871
1600.728683	800.867980	534.247745	13	N	2	261.155732	131.081504	87.723428
			14	K	1	147.112804	74.060040	49.709119

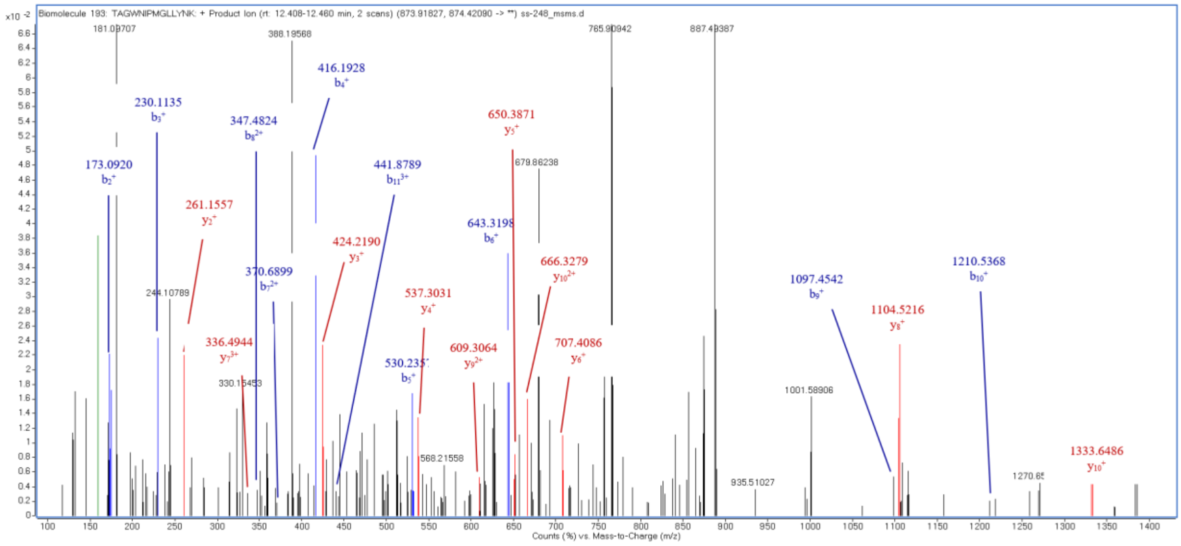

Biomolecule 184: LCMGSGLNLCEPNNK

Biomol	Seq Loc	Rule	Pred Mods	RT	Height	Mass	Tgt Mass	Diff (
184	A(516-530)	Complete digest, Predicted modifications	Alkylation (iodoacetamide) 2, Met-ChT- 3, Alkylation (iodoacetamide) 10	9.758	199506	1874.7849	1874.7784	

ECC (with sample chromatogram)

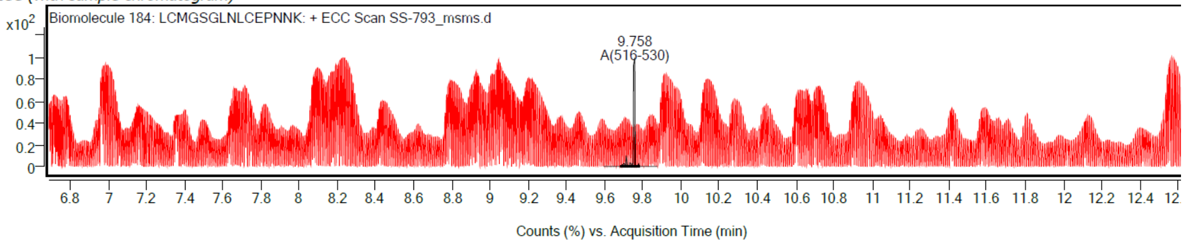

Mass Spectrum (with MFE spectrum, if available)

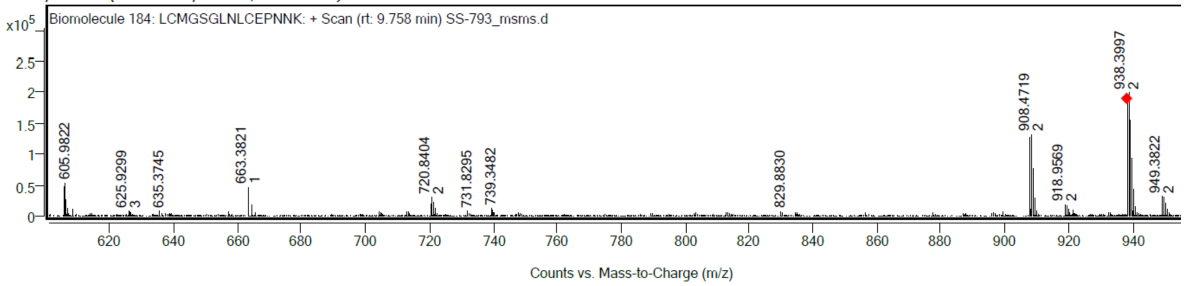

Identified Peptide Sequence: LC**M**GSGLNLCEPNNK (AA-516-AA530)

b^{+}	b^{2+}		AA		y^{+}	y^{2+}
114.091340	57.549308	1	L	15		
274.151825	137.579551	2	C+IAA	14	1762.761281	881.884279
574.212110	287.609693	3	**M+mod**	13	1602.700797	801.854037
631.233573	316.120425	4	G	12	1302.640512	651.823894
718.265602	359.636439	5	S	11	1245.619048	623.313162
775.287066	388.147171	6	G	10	1158.587020	579.797148
888.371130	444.689203	7	L	9	1101.565556	551.286416
1002.414057	501.710667	8	N	8	988.481492	494.744384
1115.498121	558.252699	9	L	7	874.438565	437.722921
1275.558606	638.282941	10	C+IAA	6	761.354501	381.180889
1404.601199	702.804238	11	E	5	601.294016	301.150646
1501.653963	751.330620	12	P	4	472.251423	236.629350
1615.696890	808.352083	13	N	3	375.198659	188.102968
1729.739818	865.373547	14	N	2	261.155732	131.081504
		15	K	1	147.112804	74.060040

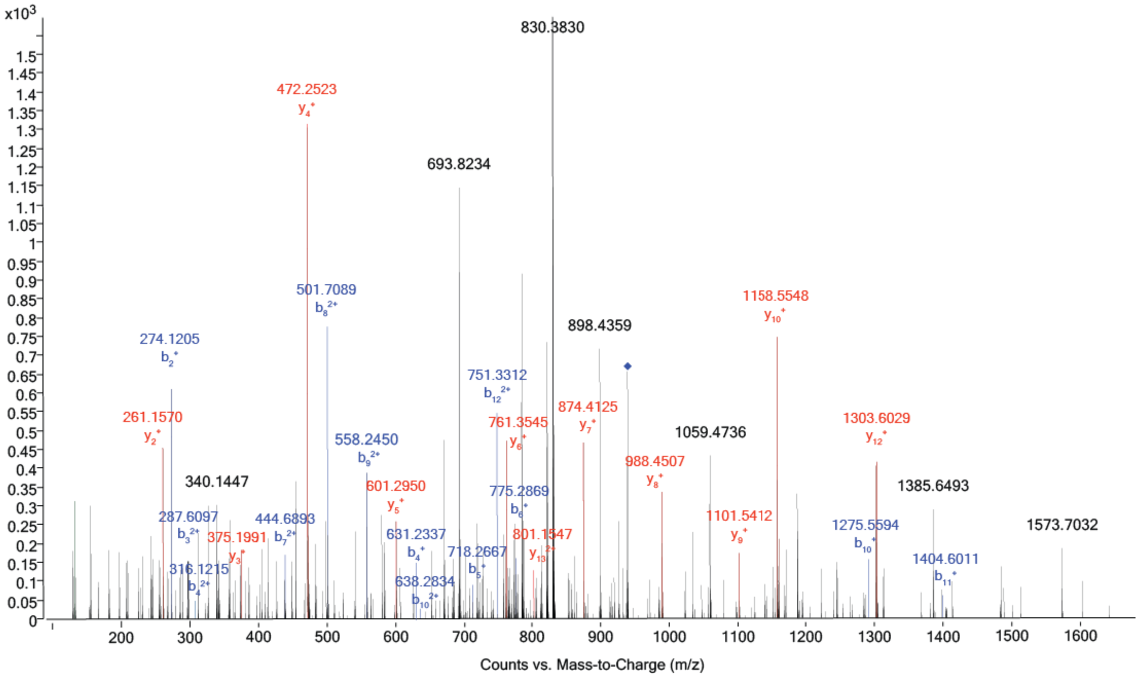

Supplementary Fig. 28. Installation of payloads in proteins and peptides:

Synthesis of the Sulfonamides:

4-(prop-2-yn-1-yloxy)benzenesulfonamide:

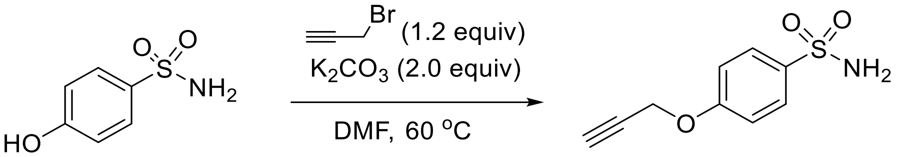

To a mixture of 4-hydroxybenzenesulfonamide (1.73 g, 10.0 mmol, 1.0 equiv) an K_2CO_3 (2.76 g, 20.0 mmol, 2.0 equiv) in dry DMF (30 mL) was added propargyl bromide (1.78 g, 80 wt% in toluene, 12.0 mmol, 1.2 equiv) at room temperature. The resulting mixture was heated at 60 °C overnight. Once the reaction was complete, it was cooled to room temperature. The crude reaction mixture was diluted with ethyl acetate and washed with ice-cold water (3×40 mL). The organic layer was dried under Na_2SO_4, concentrated, and purified on silica gel column chromatography using 10% ethyl acetate in hexane as eluent to get the titled compound as white solid (1.26 g, 60%). The analytical data matches with the literature reported data.[2]

4-((prop-2-yn-1-yloxy)methyl)benzenesulfonamide:

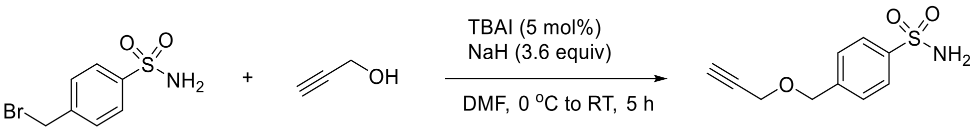

A 25 mL two-neck round bottom flask was charged with NaH (60% dispersion in mineral oil, 144 mg, 3.6 mmol, 3.6 equiv) and was backfilled with nitrogen thrice. Then dry DMF (3 mL) was added to it and the reaction mixture was cooled to 0 ^oC using an ice-bath. Then propargyl alcohol (168 mg, 3.0 mmol, 3.0 equiv) and tetrabutylammonium iodide (18.4 mg) was added dropwise. After 10 min it was brought to room temperature and stirred for 1 h. Then 4-(bromomethyl)benzenesulfonamide (250 mg, 1.0 mmol, 1.0 equiv) was added at once and the reaction stirred at RT for 5 h. Once the reaction is complete, as indicated by TLC, the reaction mixture was quenched with H_2O. The aqueous layer was extracted thrice with ethyl acetate (3×5 mL). The combined organic layer was dried with Na_2SO_4, concentrated and purified on silica by using 2% MeOH in DCM as eluent to give titled compound (142 mg, 63% yield) as white solid. **^{1}H NMR** (400 MHz, DMSO-d_6) δ = 7.82 (d, J = 8.2 Hz, 2H), 7.51 (d, J = 8.4 Hz, 2H), 7.33 (br s, 2H), 4.61 (s, 2H), 4.23 (d, J = 2.4 Hz, 1H), 3.48 (t, J = 2.4 Hz, 1H) ppm. **^{13}C NMR** (101 MHz, DMSO-d_6) δ = 143.30, 141.65, 127.68, 125.66, 79.95, 77.56, 77.54, 69.95, 57.21 ppm. **HRMS:** calcd. for $C_{10}H_{12}NO_3S$ [M+H$^+$] 226.0532; found 226.0537.

^{1}H NMR of 4-((prop-2-yn-1-yloxy)methyl)benzenesulfonamide:

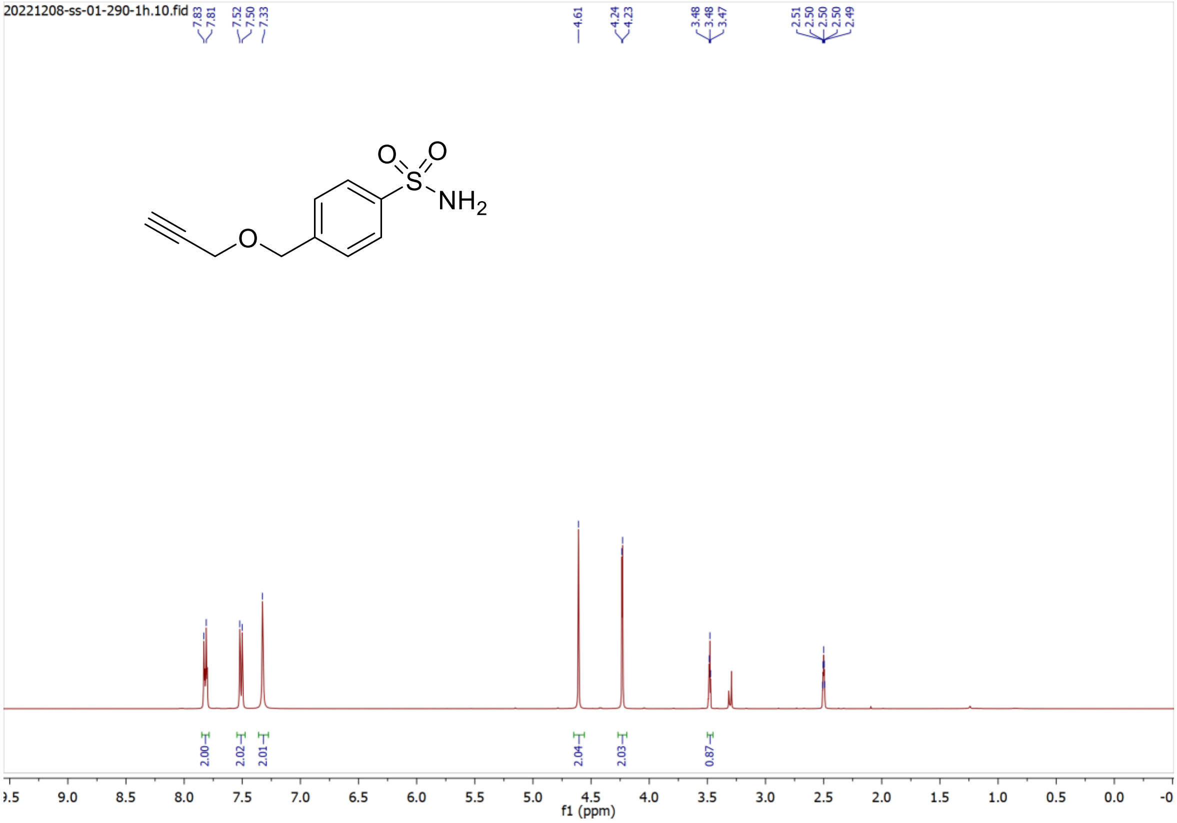

^{13}C NMR of 4-((prop-2-yn-1-yloxy)methyl)benzenesulfonamide

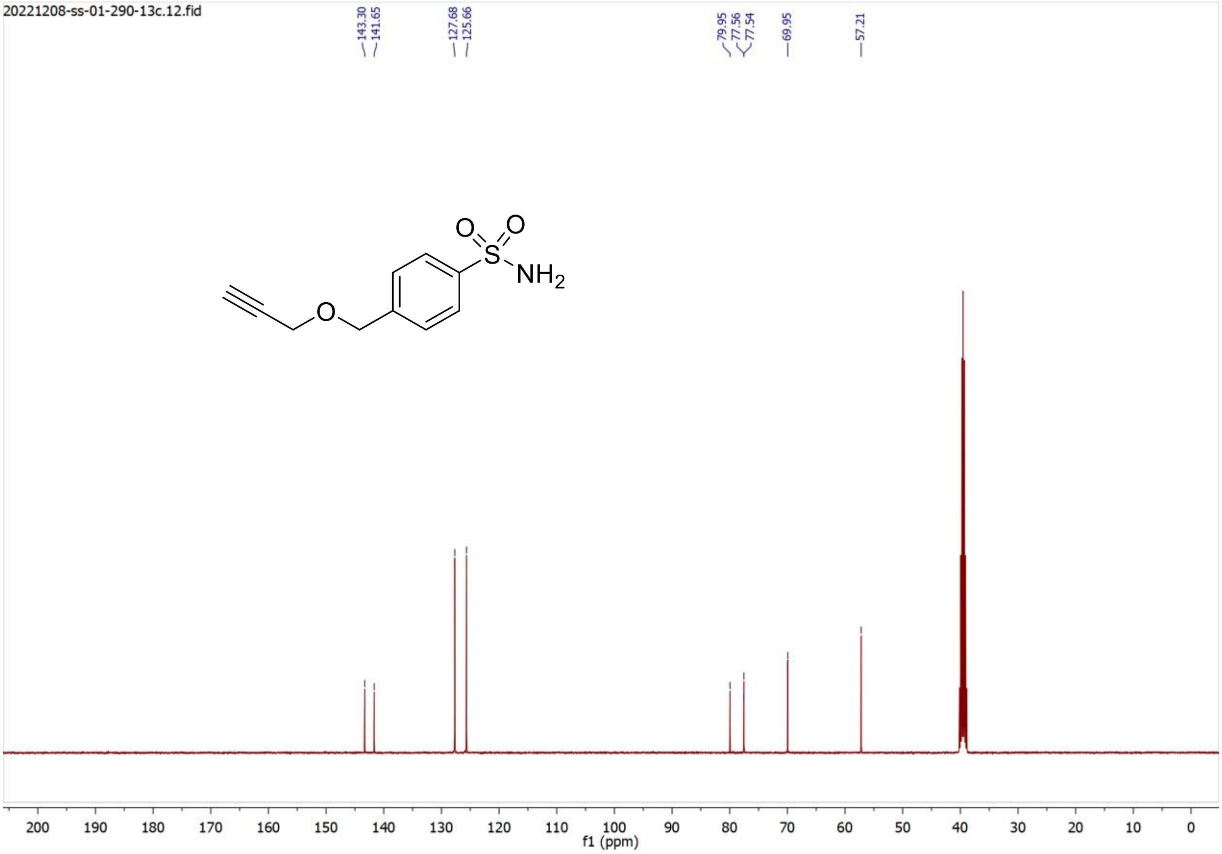

4-(2,2-diethoxyethoxy)benzenesulfonamide:

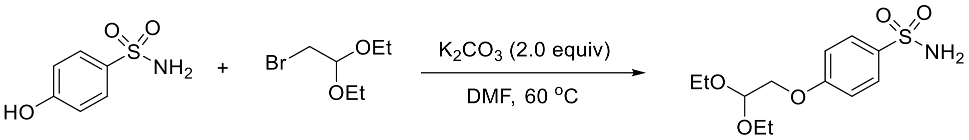

A 25 mL two round bottom flask was charged with 4-hydroxybenzenesulfonamide (865 mg, 5.0 mmol, 1.0 equiv), K_2CO_3 (1.38 g, 10 mmol, 2.0 equiv), bromoacetaldehyde diethyl acetal (1.48 g, 7.5 mmol, 1.5 equiv). Then it was backfilled with nitrogen thrice and 15 mL dry DMF was added. The resulting mixture was heated at 60 °C thereafter. After the completion of the reaction, it was cooled down to room temperature and was quenched with water. The aqueous was extracted thrice with ethyl acetate (3×15 mL). The combined organic layer was washed with brine, dried over Na_2SO_4, concentrated, and purified on silica by using 2% MeOH in DCM as eluent to give titled compound (1.05 g, 73% yield) as yellow solid. **^{1}H NMR** (400 MHz, DMSO-d_6) δ = 7.73 (d, J = 8.9 Hz, 2H), 7.22 (s, 2H), 7.11 (d, J = 8.9 Hz, 2H), 4.82 (t, J = 5.2 Hz, 1H), 4.03 (d, J = 5.2 Hz, 2H), 3.68 (dq, J = 9.6, 7.1 Hz, 2H), 3.57 (dq, J = 9.6, 7.0 Hz, 2H), 1.14 (t, J = 7.0 Hz, 6H) ppm. **^{13}C NMR** (101 MHz, DMSO-d_6) δ = 160.60, 136.46, 127.65, 114.62, 99.69, 68.36, 61.97, 15.31 ppm. **HRMS:** calcd. for $C_{13}H_{22}NO_5S$ [M+H$^+$] 304.1213; found 304.1211.

^{1}H NMR of 4-(2,2-diethoxyethoxy)benzenesulfonamide

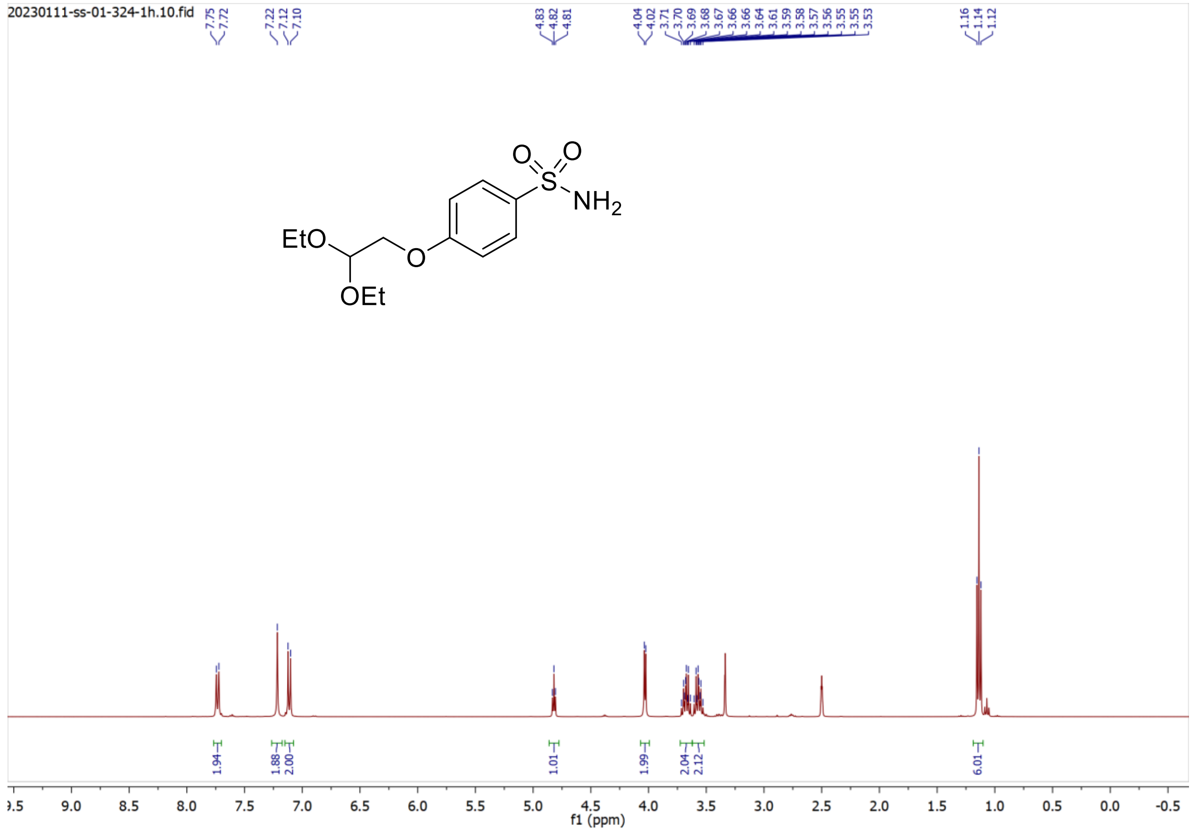

^{13}C NMR of 4-(2,2-diethoxyethoxy)benzenesulfonamide

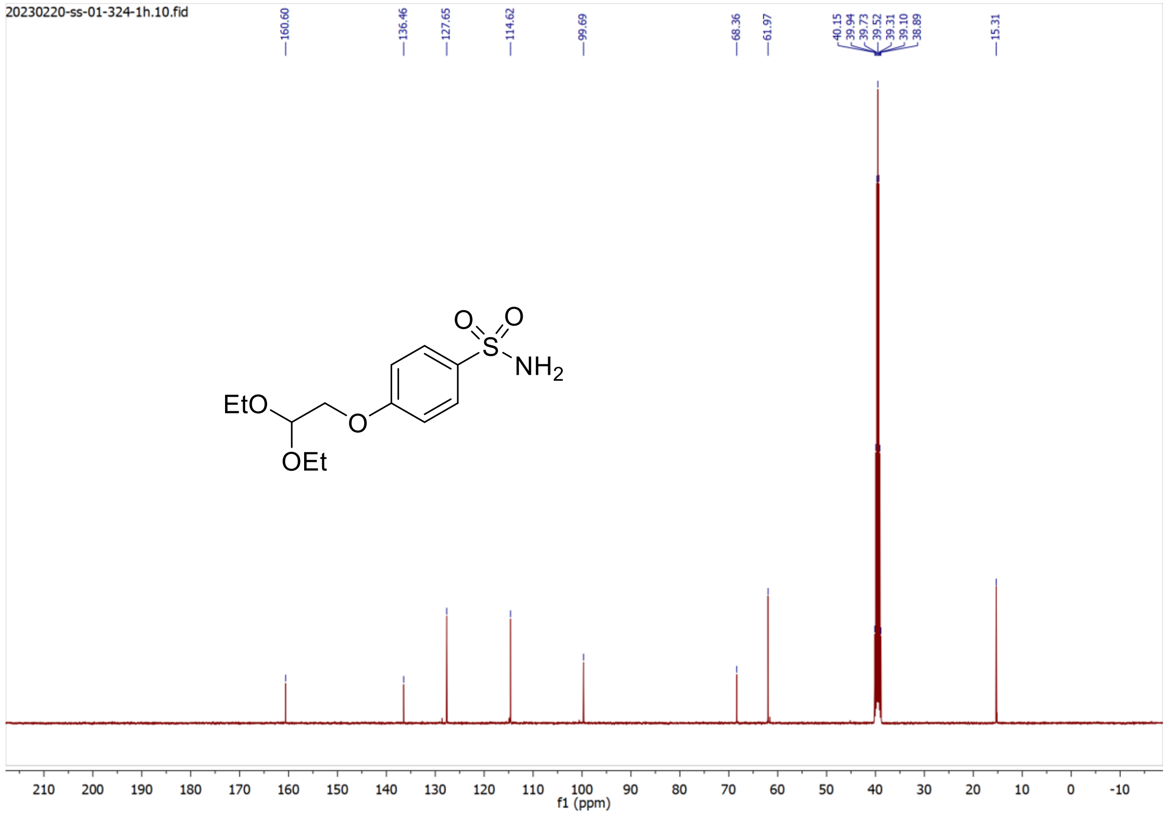

General Procedure for the Synthesis of Probes:

General Procedure I: To a solution of sulfonamide (1.0 equiv) in MeOH (0.2 M) was added trichloroisocyanuric acid (TCCA, 0.33 equiv). The reaction mixture was stirred at room temperature for 2 h and the solvent was removed under reduced pressure. Toluene was added to the resulting white precipitate and then filtered through a pad of celite. The filtrate was concentrated under reduced pressure and to the resulting liquid was subsequently added MeOH. The solution was then cooled to 0 °C and a solution of sodium hydroxide (1.0 equiv) in methanol (0.3 M) was slowly added. The reaction was allowed to stir at room temperature for 2 h. The solvent was removed under reduced pressure to afford the Chloramine-T analogues.

General Procedure II: To a solution of sulfonamide (3.0 mmol, 1.0 equiv) in H_2O (6 mL), was added crushed NaOH (120 mg, 3.0 mmol, 1.0 equiv) at 0 °C. After 5 min, once sulfonamide is dissolved then 15% NaOCl solution (2 mL, 3.15 mmol, 1.05 equiv) was dropwisely added over 10 min. The resulting reaction mixture was slowly allowed to come room temperature and stirred for 24 h. The appeared solid was filtered and washed with cold ether thrice. Finally, the resulting solid was dried under vacuum to give the Chloramine-T analogues.

Sodium chloro((4-(prop-2-yn-1-yloxy)phenyl)sulfonyl)amide (1i)

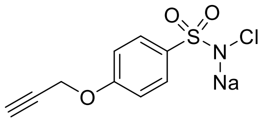

The titled compound was synthesized according to general procedure I by using general procedure A (422 mg, 2.0 mmol, 1.0 equiv), TCCT (172 mg, 0.74 mmol, 0.37 equiv), NaOH (80 mg, 2.0 mmol, 1.0 equiv). The resulting product **1i** was isolated as yellow solid (400 mg, 75% yield). **^{1}H NMR** (400 MHz, DMSO-d_6): δ = 7.55 (d, J = 9.1 Hz, 2H), 6.95 (d, J = 8.7 Hz, 2H), 4.81 (d, J = 2.4 Hz, 2H), 3.56 (t, J = 2.4 Hz, 1H) ppm. **^{13}C NMR** (101 MHz, DMSO-d_6) δ = 157.82, 138.40, 128.52, 113.82, 79.11, 78.39, 55.46 ppm. **HRMS:** calcd. for $C_9H_7ClNNa_2O_3S$ [M+Na$^+$] 289.9625; found 289.9628.

^{1}H NMR of Compound 1i

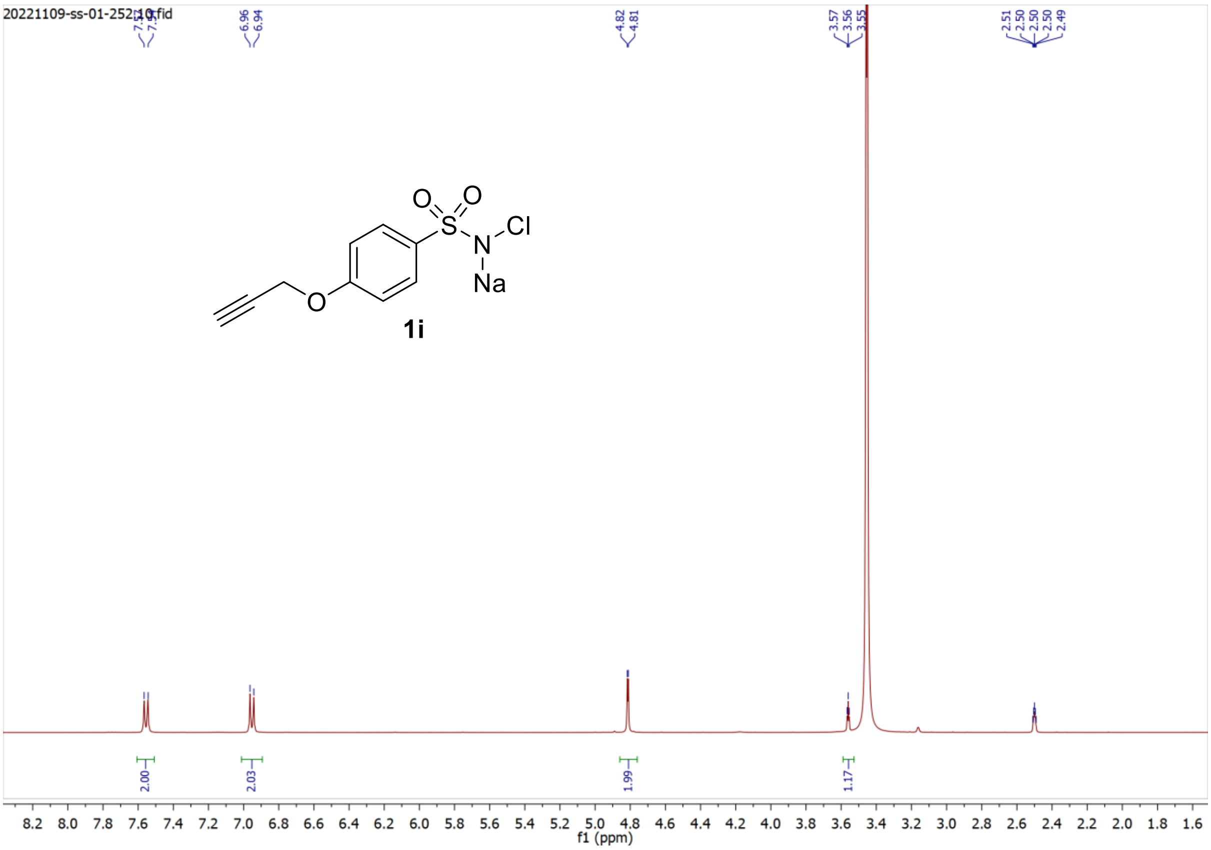

^{13}C NMR of Compound 1i

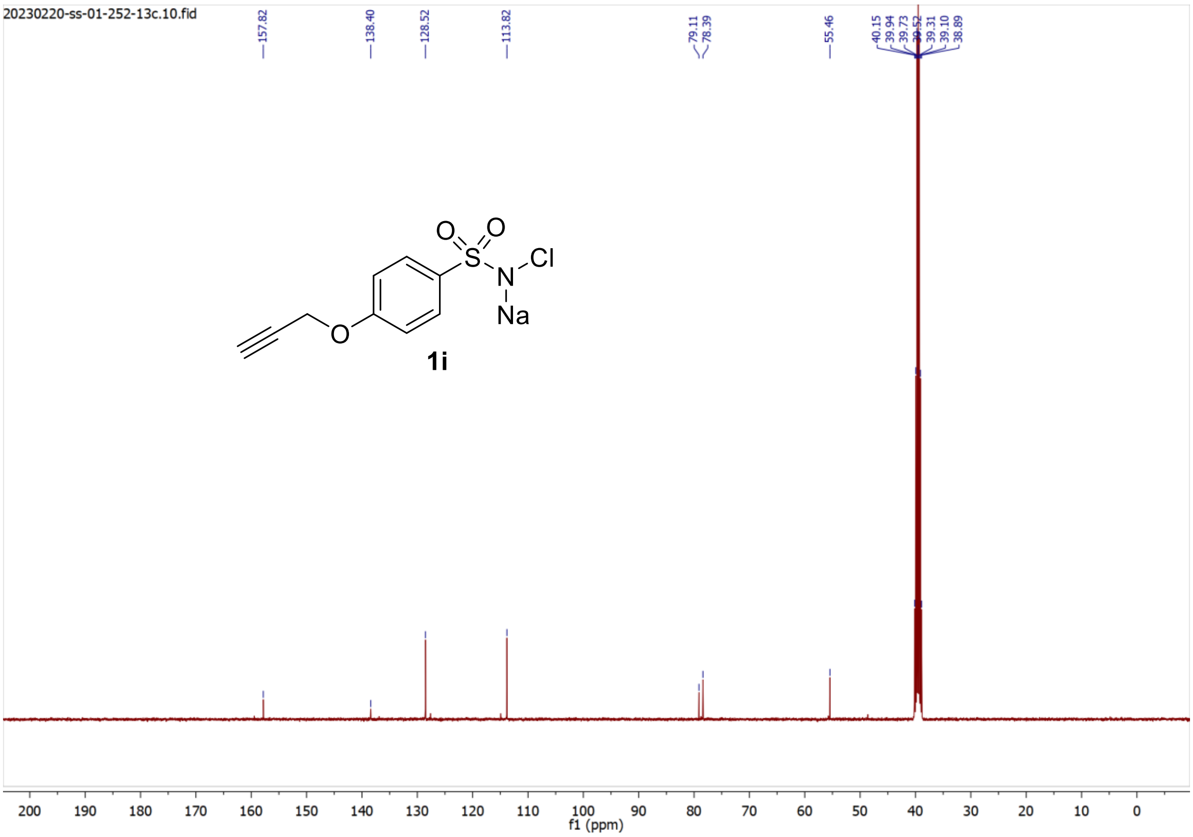

Sodium chloro((4-(2,2-diethoxyethoxy)phenyl)sulfonyl)amide (1j)

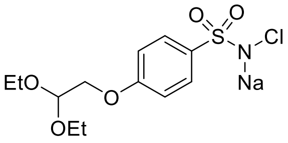

The titled compound was synthesized according to general procedure II by using 4-(2,2-diethoxyethoxy)benzenesulfonamide (714 mg, 2.17 mmol), NaOH (99 mg, 2.47 mmol), and 15% NaOCl solution (1.6 mL). The resulting solution was lyophilized to get the product **1j** as white solid (795 mg, 93%) as whitish solid. **^{1}H NMR** (400 MHz, DMSO-d_6) δ = 7.54 (d, J = 8.6 Hz, 2H), 6.92 (d, J = 8.8 Hz, 2H), 4.79 (t, J = 5.1 Hz, 1H), 3.96 (d, J = 5.1 Hz, 2H), 3.66 (dq, J = 8.7, 7.1 Hz, 2H), 3.55 (dq, J = 9.3, 6.2 Hz, 2H), 1.13 (t, J = 7.0 Hz, 6H) ppm. **^{13}C NMR** (101 MHz, DMSO-d_6) δ = 158.94, 137.86, 128.66, 113.56, 99.83, 68.20, 61.91, 15.34 ppm. **HRMS:** calcd. for $C_{12}H_{17}ClNNa_2O_5S$ [M+Na$^+$] 368.0306; found 368.0312.

^{1}H NMR of Compound 1j

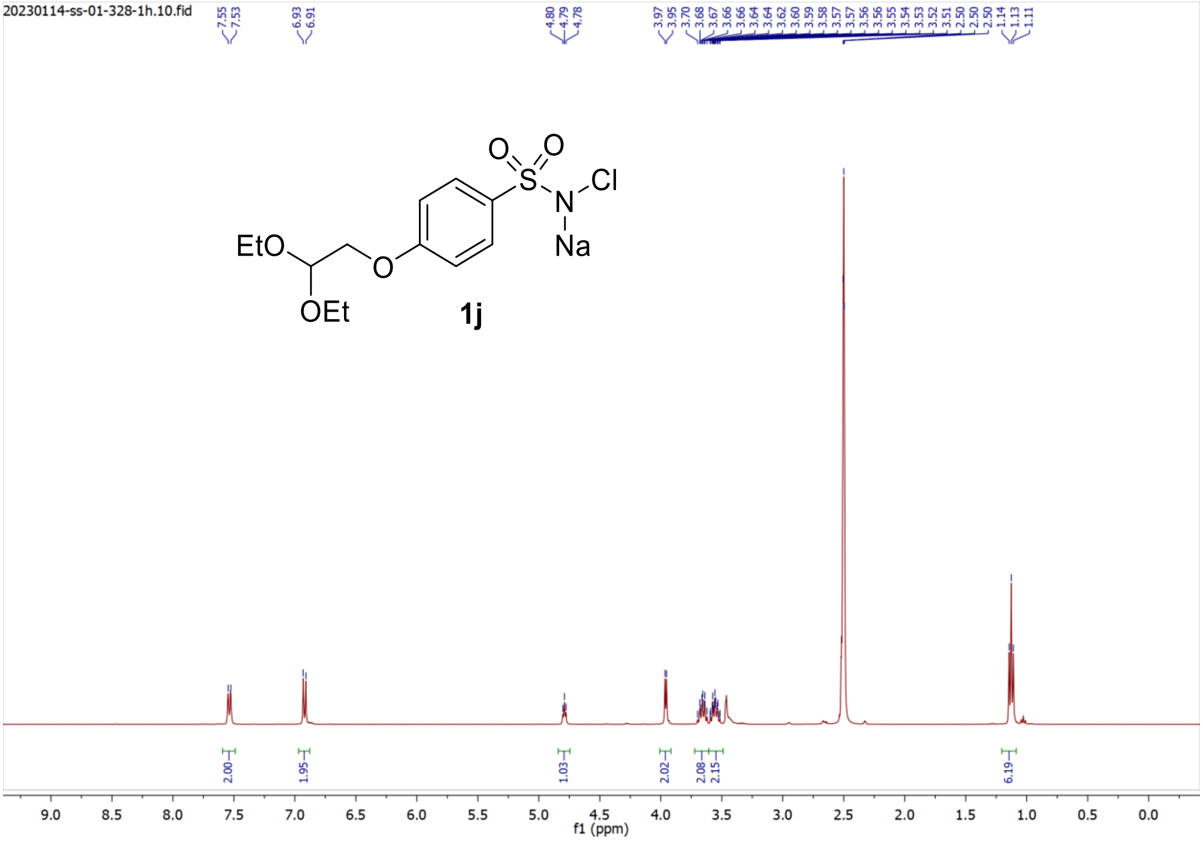

^{13}C NMR of Compound 1j

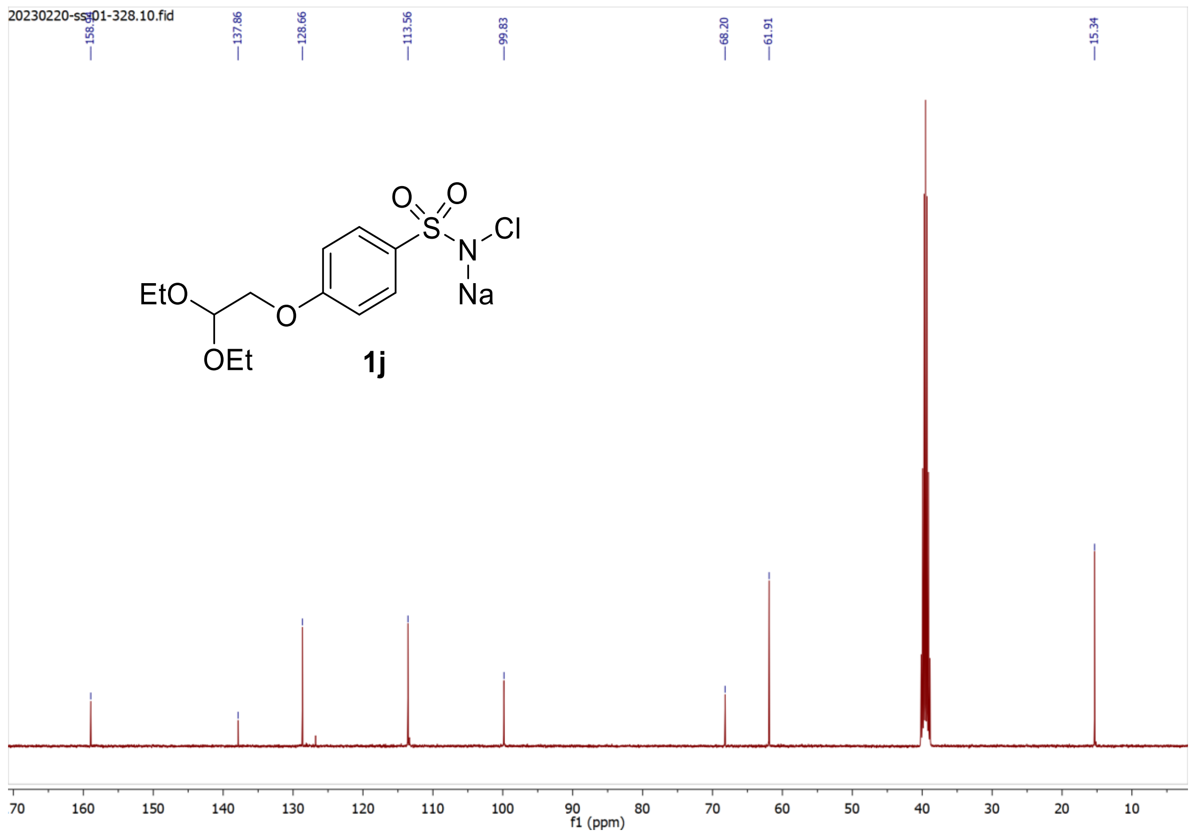

Labeling of myoglobin with 1h using CuNiP

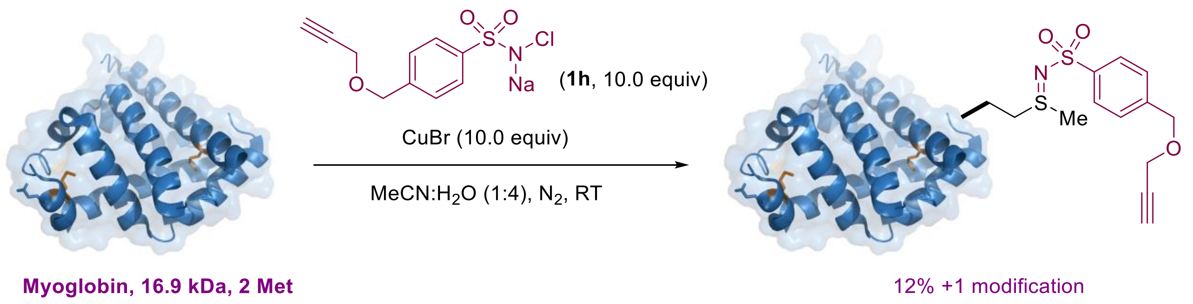

Myoglobin (2 mg, 0.12 µmol, 1.0 equiv) was dissolved in MeCN:H_2O (1:4, 800 µL) and CuBr (12 mM in MeCN, 100 µL, 1.2 µmol), **1h** (12 mM in H_2O, 100 µL, 1.2 µmol) were added sequentially. The reaction mixture was incubated at 25 °C for 2 h under nitrogen atmosphere followed by the addition of 10 µL of 0.5 N HCl. The crude reaction mixture was passed through Amicon Ultra 3 kDa spin-concentrator and washed with H_2O (7×0.5 mL) to remove the small molecule impurities. This labeled protein was lyophilized, redissolved in 0.1% formic acid in H_2O and analyzed using LC-MS. The conversion was found to be >95%. Intact mass analysis shows [O+1 mod] as 12%, along with mono-sulfoxide and bis-sulfoxide as a side product.

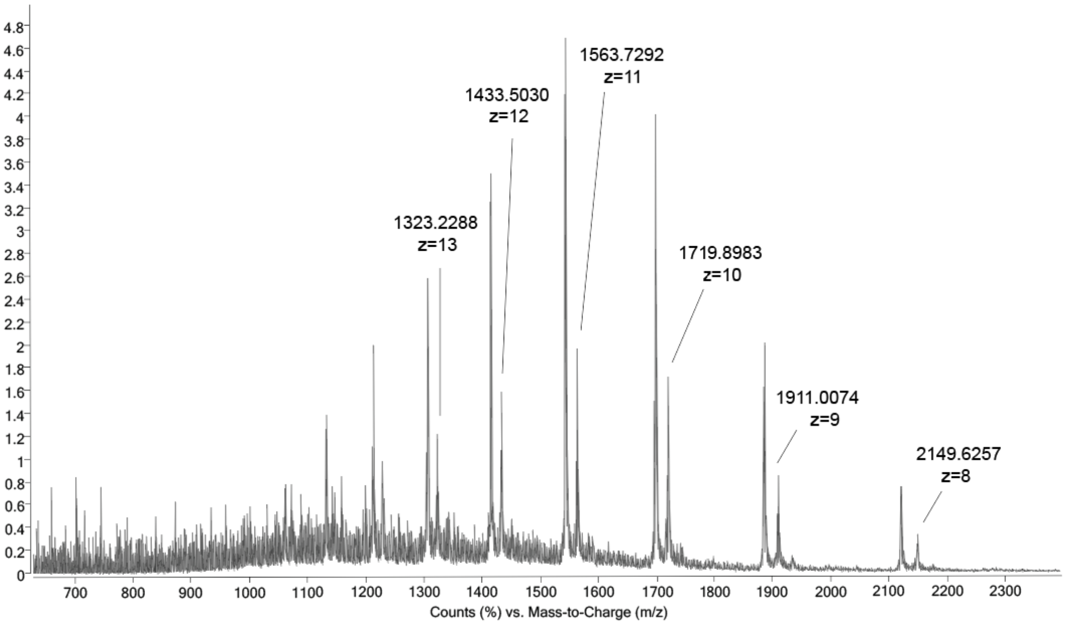

MS spectra of **1h** modified myoglobin

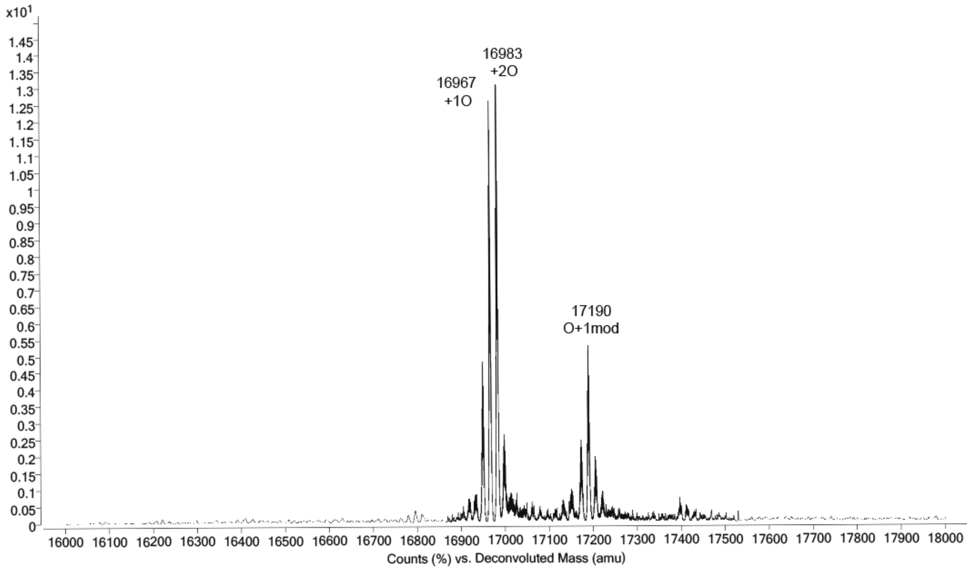

Deconvoluted MS spectra of **1h** modified Myoglobin

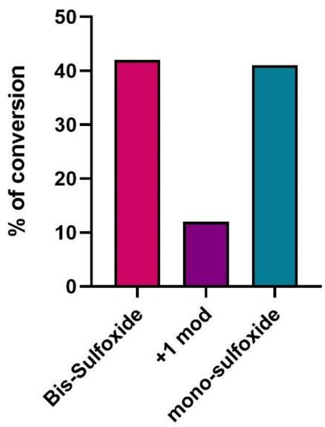

MS/MS Analysis of 1h modified myoglobin:

Biomolecule 21: HPGDFGADAQGAMTK

Biomol	Seq Loc	Rule	Pred Mods	RT	Height	Mass	Tgt Mass	Diff (ppm)
21	A(119-133)	Complete digest, Predicted modifications	Met-Cht-Alkyne-CH2O- 13	7.771	877695	1724.6945	1724.6923	1.26

ECC (with sample chromatogram)

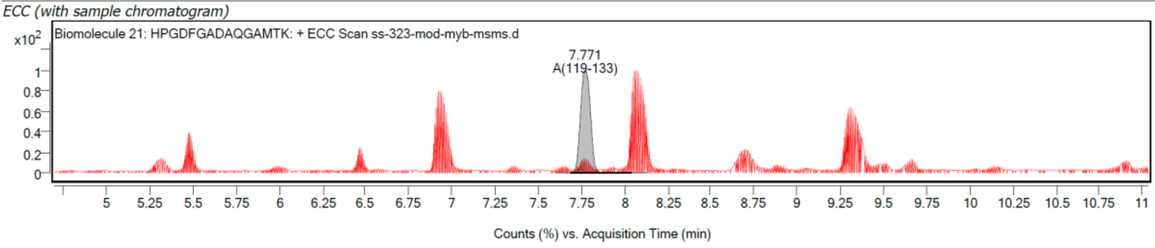

Mass Spectrum (with MFE spectrum, if available)

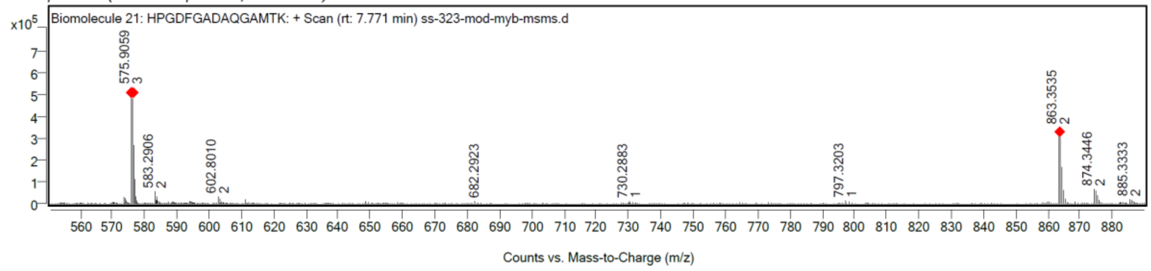

Identified Peptide Sequence: (AA199-AA133): HPGDFGADAQGA**M**TK

b$^+$	b^{2+}		AA		y$^+$	y^{2+}
138.066188	69.536732	1	H	15		
235.118952	118.063114	2	P	14	1588.864141	794.935709
292.140416	146.573846	3	G	13	1491.811378	746.409327
407.167359	204.087318	4	D	12	1434.789914	717.898595
554.235773	277.621525	5	F	11	1319.762971	660.385124
611.257237	306.132257	6	G	10	1172.694557	586.850917
682.294351	341.650813	7	A	9	1115.673093	558.340185
797.321294	399.164285	8	D	8	1044.635979	522.821628
868.358407	434.682842	9	A	7	929.609036	465.308156
996.416985	498.712131	10	Q	6	858.571922	429.789599
1053.438449	527.222863	11	G	5	730.513345	365.760311
1124.475562	562.741419	12	A	4	673.491881	337.249579
1478.769847	739.888562	13	**M+mod**	3	602.454767	301.731022
1579.817526	790.412401	14	T	2	248.160483	124.583880
		15	K	1	147.112804	74.060040

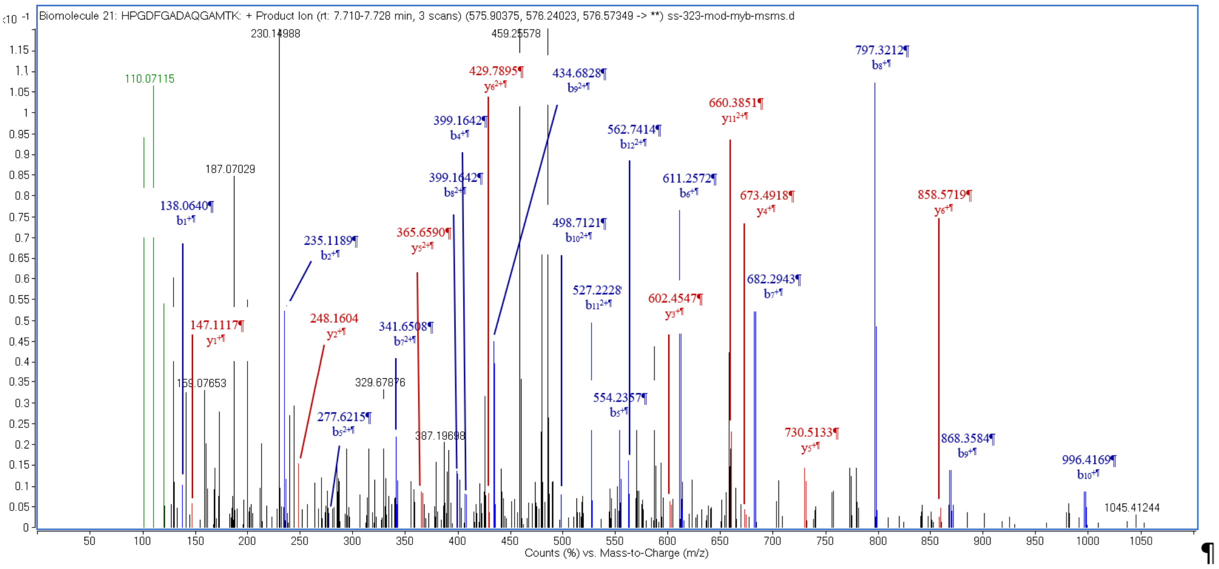

Labeling of myoglobin with 1i using CuNiP:

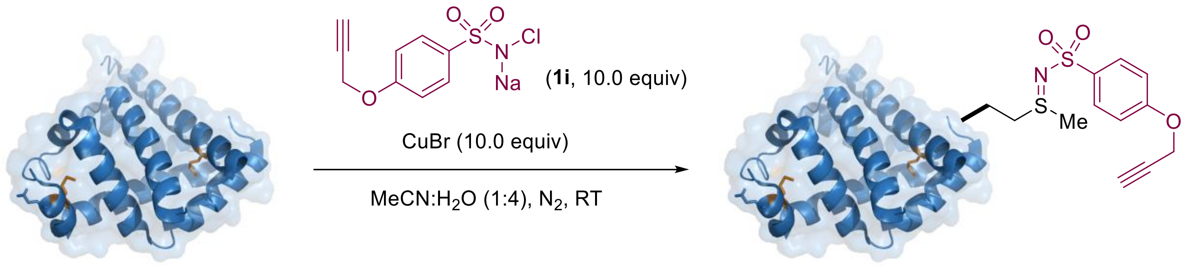

Myoglobin, 16.9 kDa, 2 Met

2i
44% +1 modification
3% +2 modification

Myoglobin (2 mg, 0.12 µmol, 1.0 equiv) was dissolved in MeCN:H_2O (1:4, 800 µL) and CuBr (12 mM in MeCN, 100 µL, 1.2 µmol), **1i** (12 mM in H_2O, 100 µL, 1.2 µmol) were added sequentially. The reaction mixture was incubated at 25 °C for 2 h under nitrogen atmosphere followed by the addition of 10 µL of 0.5 N HCl. The crude reaction mixture was passed through Amicon Ultra 3 kDa spin-concentrator and washed with H_2O (7×0.5 mL) to remove the small molecule impurities. This labeled protein was lyophilized, redissolved in 0.1% formic acid in H_2O and analyzed using LC-MS. The conversion was found to be >95%. Intact mass analysis shows [O+1 mod] as 47%, along with bis-sulfoxide as a side product.

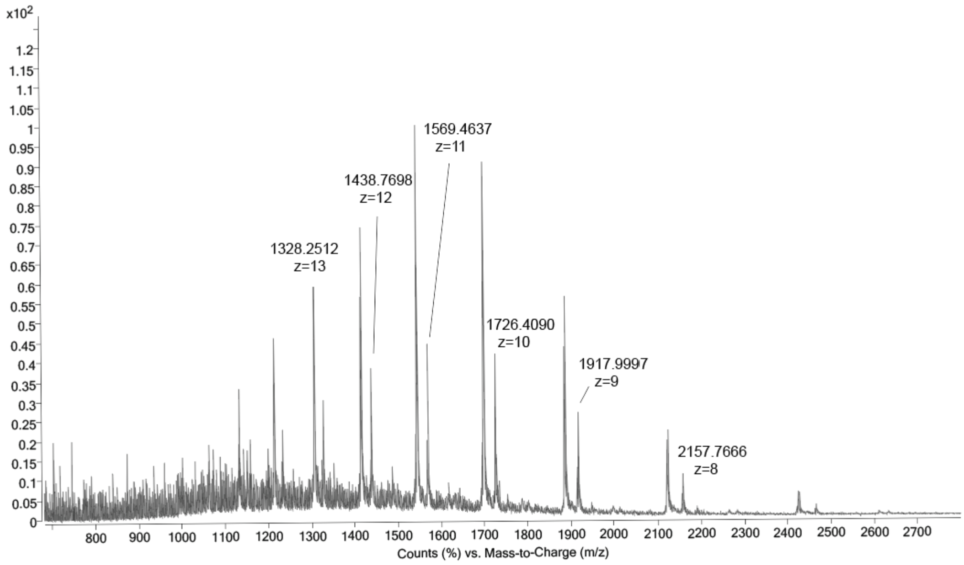

MS spectra of **1i** modified myoglobin

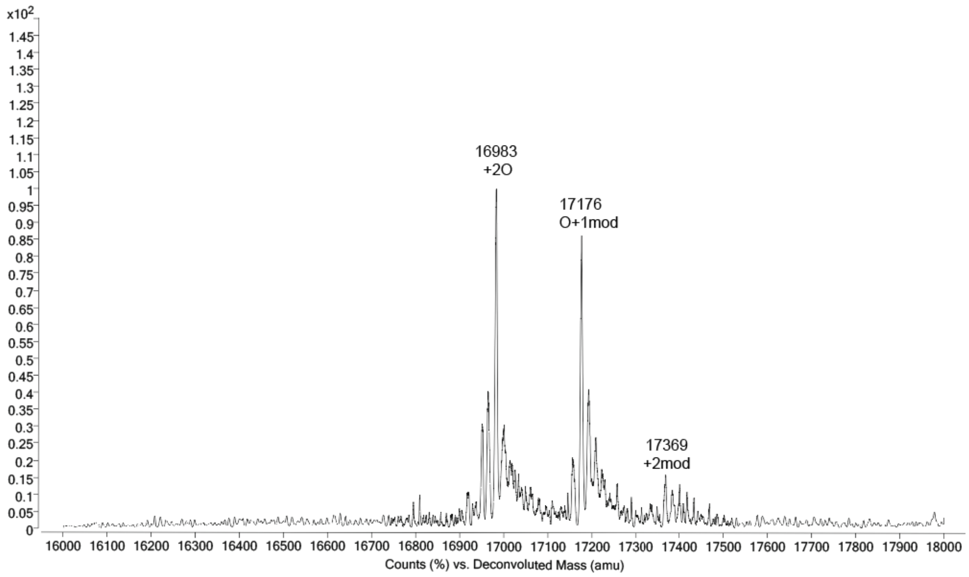

Deconvoluted MS spectra of **1i** modified myoglobin

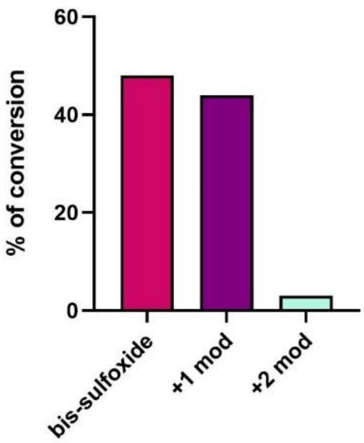

Labeling of Myoglobin with 1j using CuNiP:

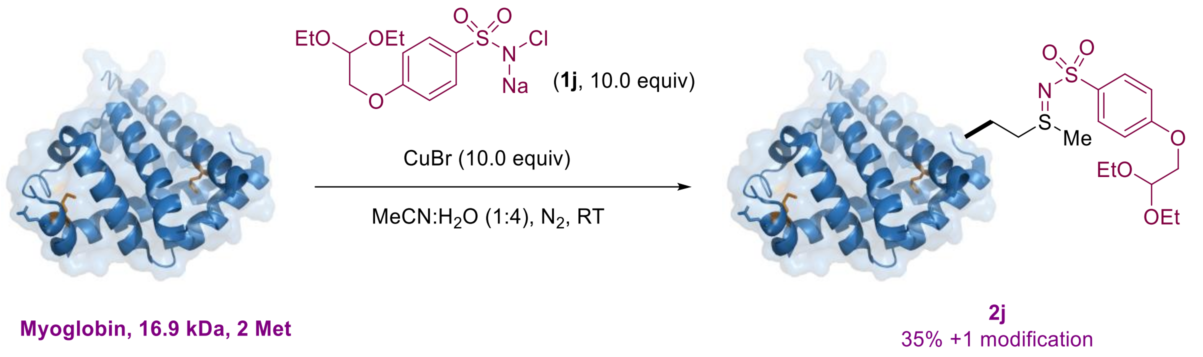

Myoglobin (2 mg, 0.12 µmol, 1.0 equiv) was dissolved in MeCN:H_2O (1:4, 800 µL) and CuBr (12 mM in MeCN, 100 µL, 1.2 µmol), **1j** (12 mM in H_2O, 100 µL, 1.2 µmol) were added sequentially. The reaction mixture was incubated at 25 °C for 2 h under nitrogen atmosphere followed by the addition of 10 µL of 0.5 N HCl. The crude reaction mixture was passed through Amicon Ultra 3 kDa spin-concentrator and washed with H_2O (7×0.5 mL) to remove the small molecule impurities This labeled protein was lyophilized, and redissolved in 0.1% formic acid in H_2O and was analyzed using LC-MS. The conversion was found to be >95%. Intact mass analysis shows [O+1 mod] as 35%, along with bis-sulfoxide as a side product.

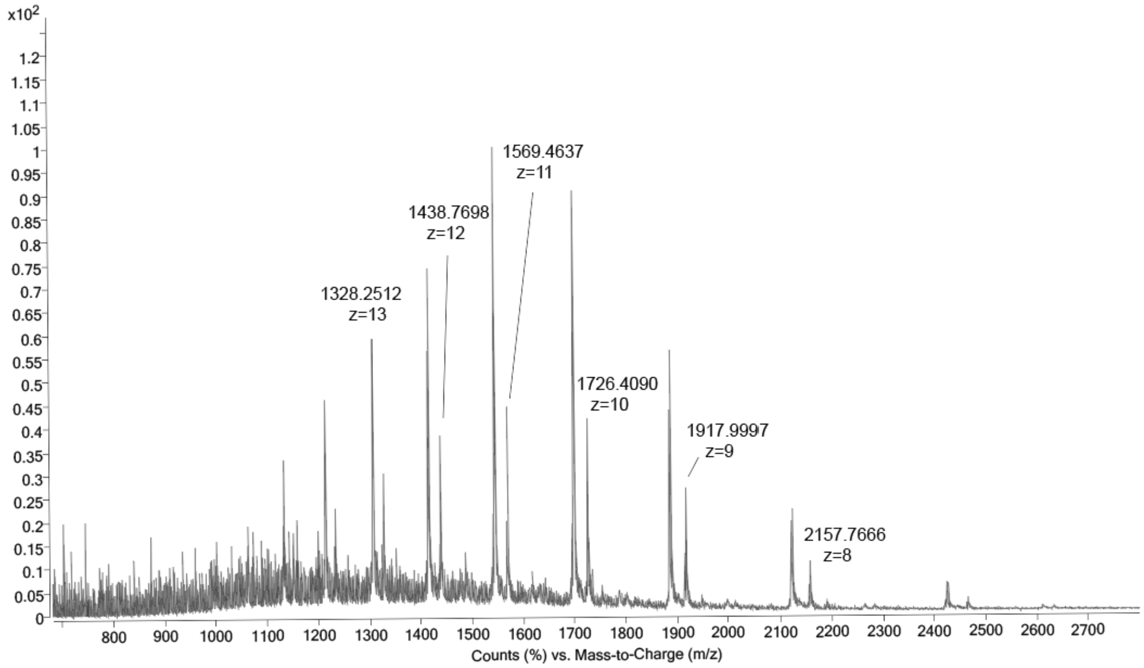

MS spectra of **1j** modified myoglobin

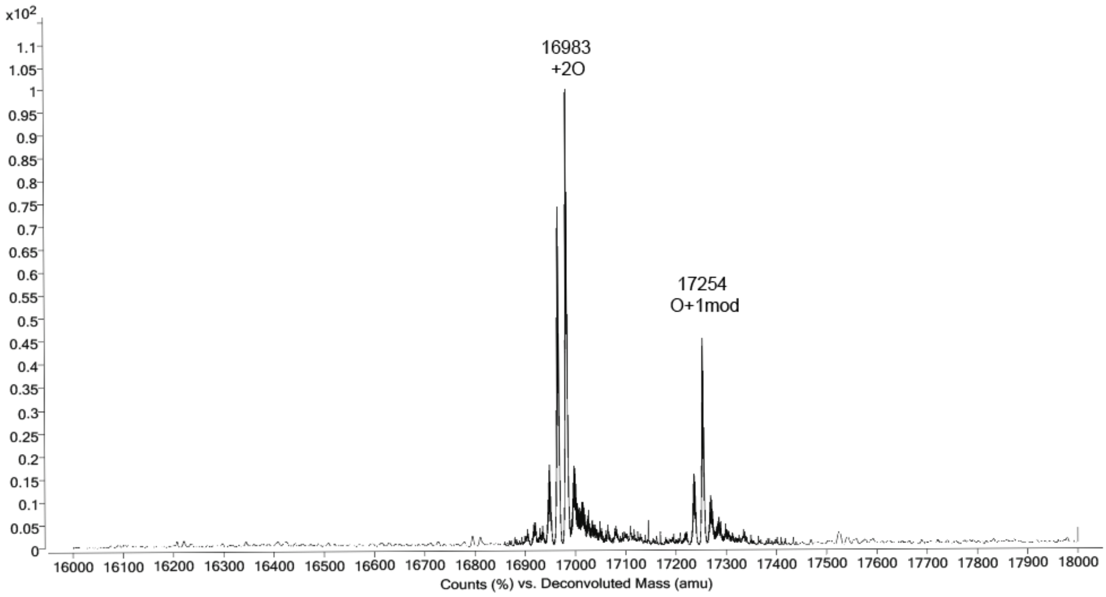

Deconvoluted MS spectra of **1j** modified myoglobin

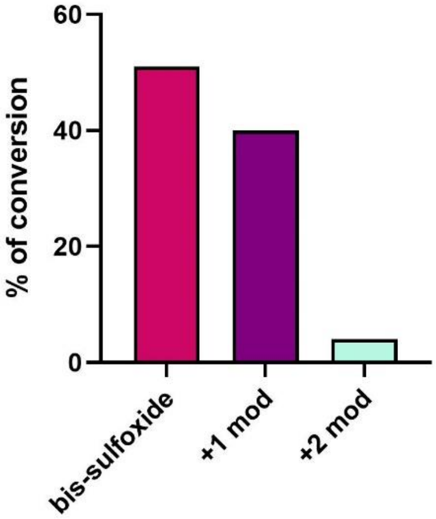

Supplementary Fig. 29. CD Spectra of the 1a Modified Myoglobin:

CD measurements were performed on a Jasco J-1500 CD spectropolarimeter using 0.20-mm thick quartz plates from Hellma USA Inc (Plainview, NY). Unmodified myoglobin and **1a** modified myoglobin were dissolved as 50 µM stock solution in water. Samples were micro-pipetted onto a 50 µL Hellma Analytics quartz cell with a 0.1 mm path length (Model # 106-0.10-40). Three spectras were collected and averaged in a wavelength range from 190 to 260 nm at a scanning range of 100 nm/min with a bandwidth of 2 nm and a data pitch of 0.2 nm.

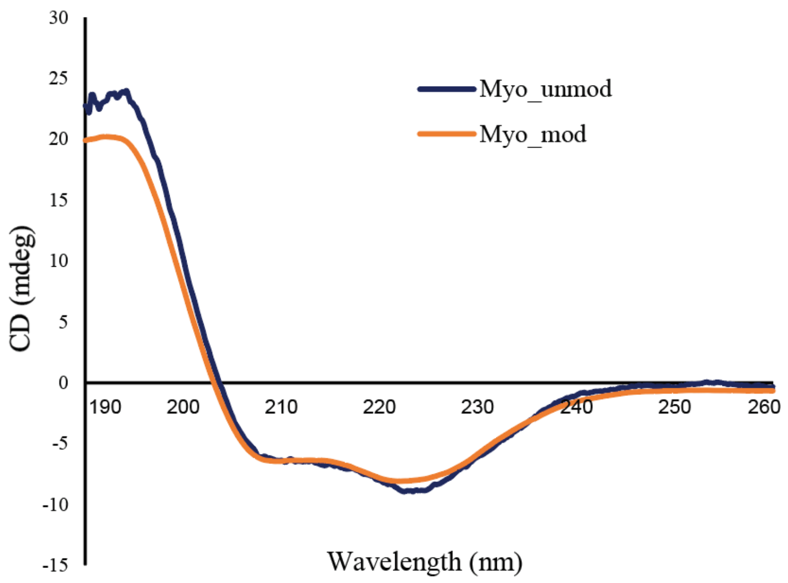

Supplementary Fig. 30. Installation of payloads onto bioactive peptides

Labeling of adrenomedullin with 1i using CuNiP

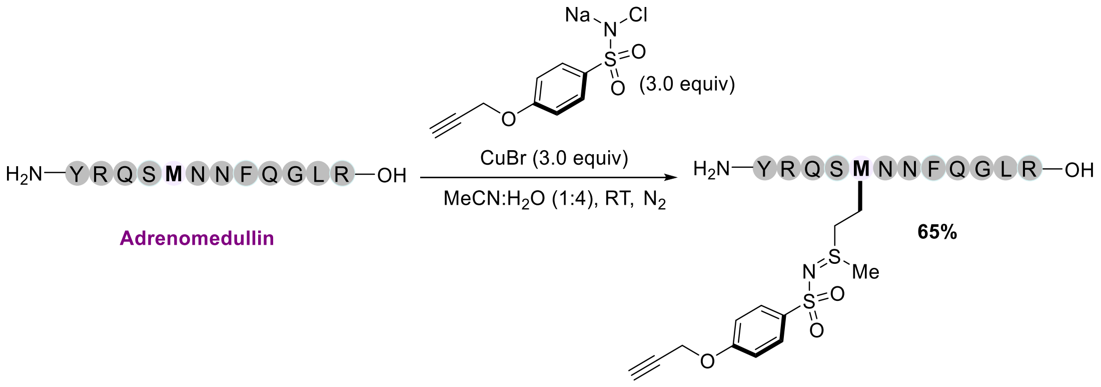

Adrenomedullin (1 mg, 0.7 µmol, 1.0 equiv), CuBr (0.3 mg, 2.1 µmol, 3.0 equiv), and **1i** (0.56 mg, 2.1 µmol, 3.0 equiv) were dissolved in MeCN:H_2O (1:4, 550 µL) and stirred at 25 °C for 3 h under nitrogen atmosphere followed by the addition of 10 µL of 0.5 N HCl. The crude mixture was analyzed on HPLC (Gradient: 0-70% solvent B over 30 min, solvent B: 0.1% formic acid in MeCN) and MS.

Unmodified Adrenomedullin NH_2-YRQSMNNFQGLR-OH: LCMS *m/z* 1514.5359 (calc. $[M+H^+]$ = 1514.5348), *m/z* 757.4369 (calc. $[M+2H^+]^{2+}$ = 757.7674), *m/z* 505.3256 (calc. $[M+3H^+]^{3+}$ 505.3298) Purity: > 99 % (HPLC analysis at 220 nm). Retention time in HPLC: 4.288 min.

1i modified Adrenomedullin NH_2-YRQSMNNFQGLR-OH: LCMS *m/z* 1722.7572 (calc. $[M+H^+]$ = 1722.7568), *m/z* 861.8775 (calc. $[M+2H^+]^{2+}$ = 861.8763), Purity: > 99 % (HPLC analysis at 220 nm). Retention time in HPLC: 4.773 min.

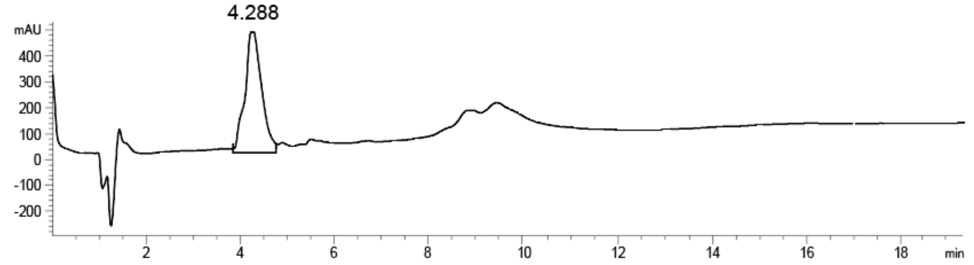

HPLC trace of unmodified Adrenomedullin

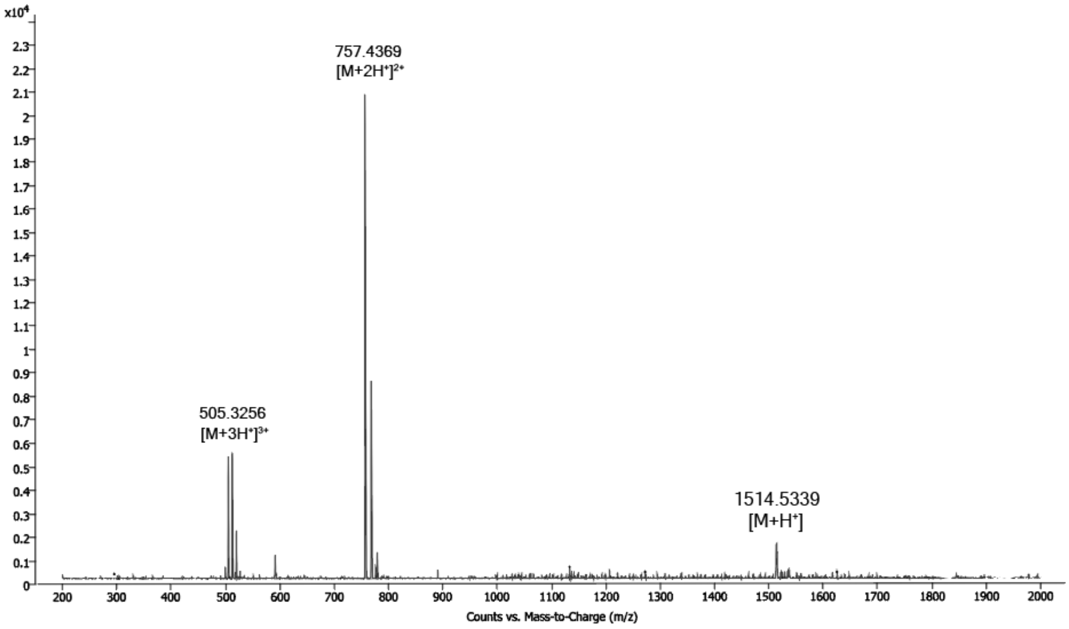

MS spectra of unmodified Adrenomedullin

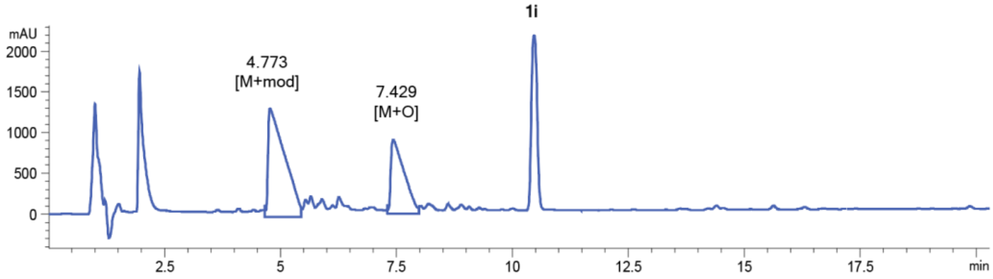

Peak #	RetTime [min]	Type	Width [min]	Area [mAU*s]	Height [mAU]	Area %
1	4.773	MM	0.4150	3.30201e4	1326.00842	64.6399
2	7.429	MM	0.3373	1.82852e4	903.41602	35.3601

HPLC trace of **1i** modified Adrenomedullin using CuNiP

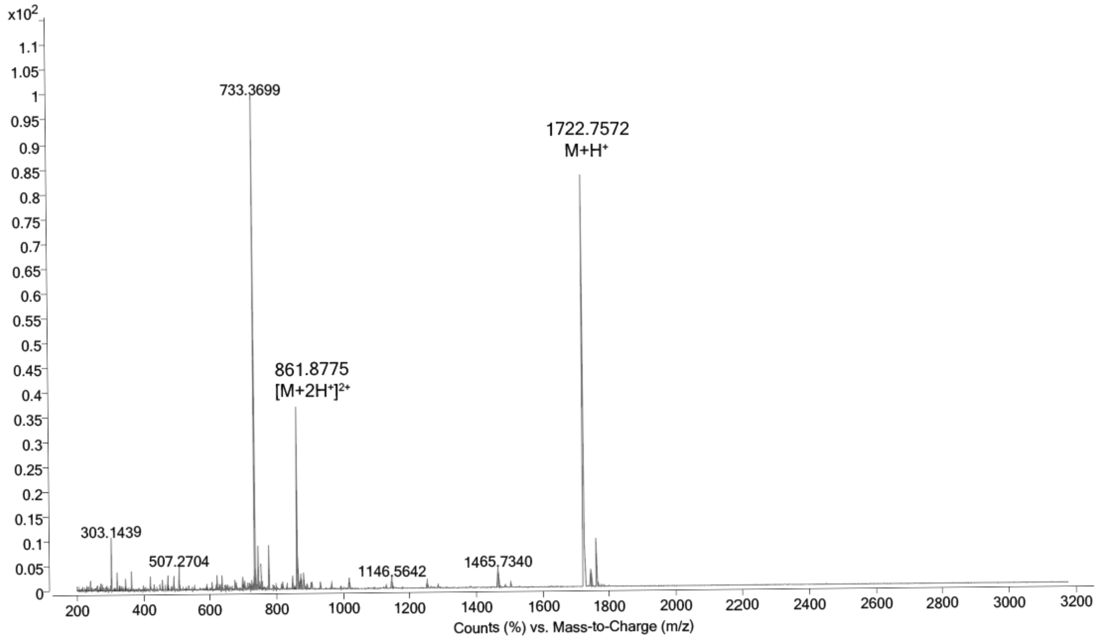

MS spectra of **1i** modified Adrenomedullin using CuNiP

Labeling of tetracosactide acetate with 1i using CuNiP

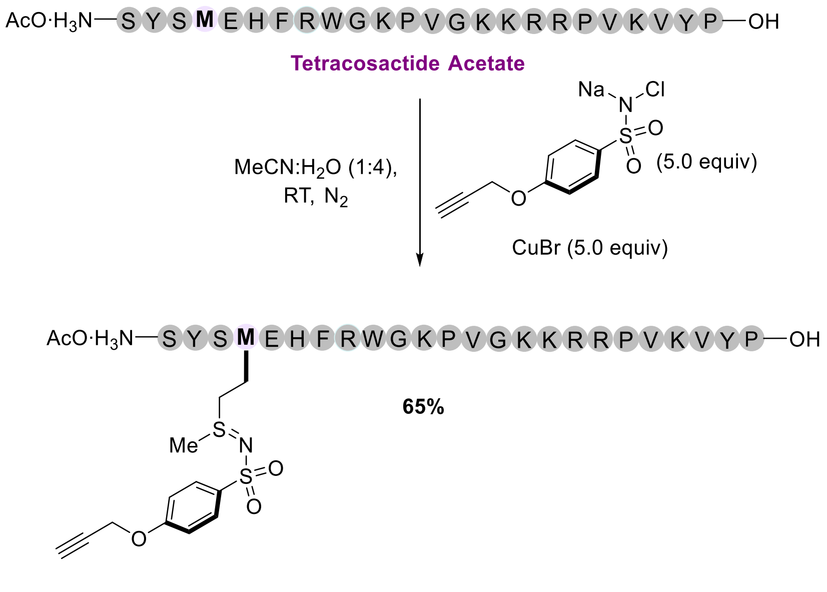

Tetracosactide acetate (2 mg, 0.7 µmol, 1.0 equiv), CuBr (0.5 mg, 3.5 µmol, 5.0 equiv), and **1i** (0.9 mg, 3.5 µmol, 5.0 equiv) were dissolved in MeCN:H_2O (1:4, 550 µL) and stirred at 25 °C for 3 h followed by the addition of 10 µL of 0.5 N HCl. The crude mixture was analyzed on HPLC (Gradient: 0-70% solvent B over 30 min, solvent B: 0.1% formic acid in MeCN) and MS.

Unmodified Tetracosactide Acetate NH2-SYSMEHFRWGKPVGKKRRPVKVYP-OH: LCMS *m/z* 1466.7954 (calc. 1466.7966 $[M+2H^+]^{2+}$), *m/z* 978.1572 (calc. $[M+3H^+]^{3+}$ = 978.2008), *m/z* 733.9035 (calc. $[M+4H^+]^{4+}$ = 733.9024), *m/z* 587.3240 (calc. $[M+5H^+]^{5+}$ =

587.3234) *m/z* 489.6048 (calc. $[M+6H^+]^{6+}$ = 489.6050) Purity: > 99 % (HPLC analysis at 220 nm). Retention time in HPLC: 8.247 min.

1i modified Tetracosactide Acetate NH_2-SYSMEHFRWGKPVGKKRRPVKVYP-OH: LCMS *m/z* 1570.8037 (calc. $[M+2H^+]^{2+}$ = 1571.3049), 1047.8753 (calc. $[M+3H^+]^{3+}$ = 1048.8723), *m/z* 786.1547 (calc. $[M+4H^+]^{4+}$ = 786.1553), *m/z* 629.1276 (calc. $[M+5H^+]^{5+}$ = 629.1263) Purity: > 99 % (HPLC analysis at 220 nm). Retention time in HPLC: 6.995 min.

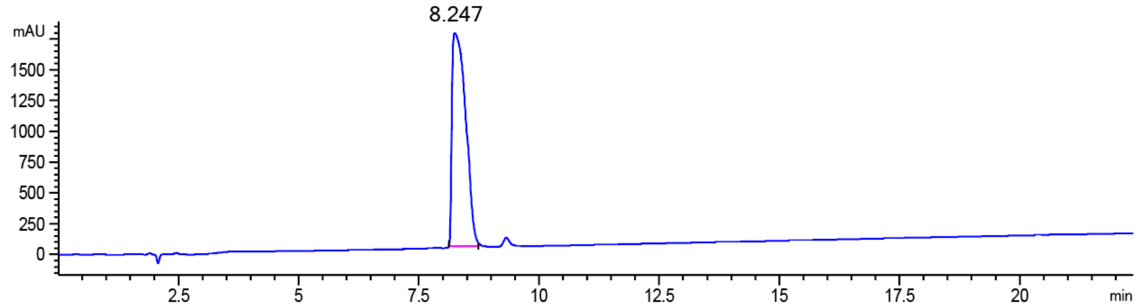

HPLC trace of unmodified tetracosactide

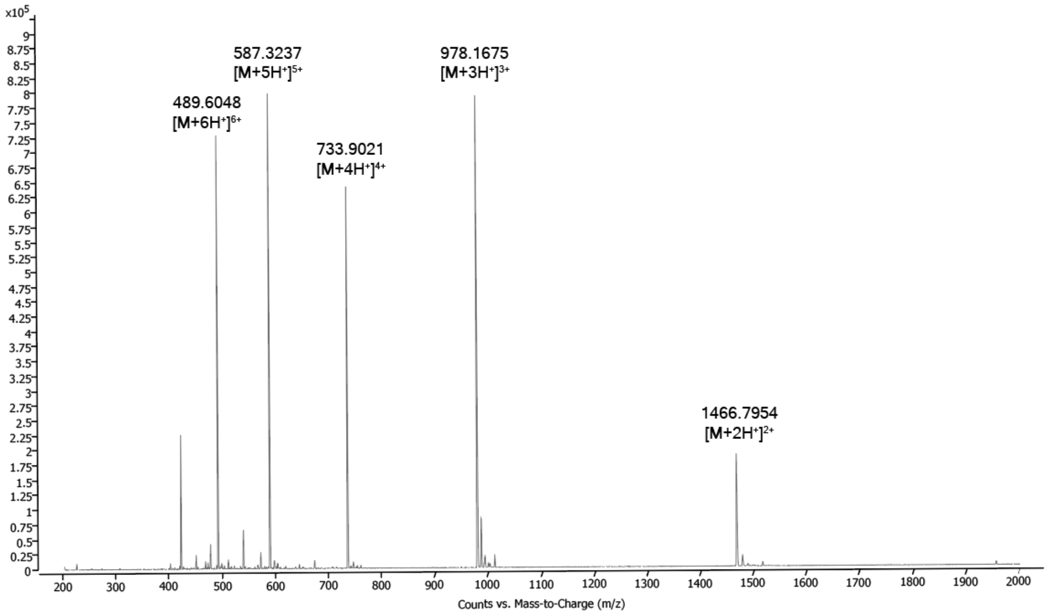

MS spectra of unmodified tetracosactide acetate

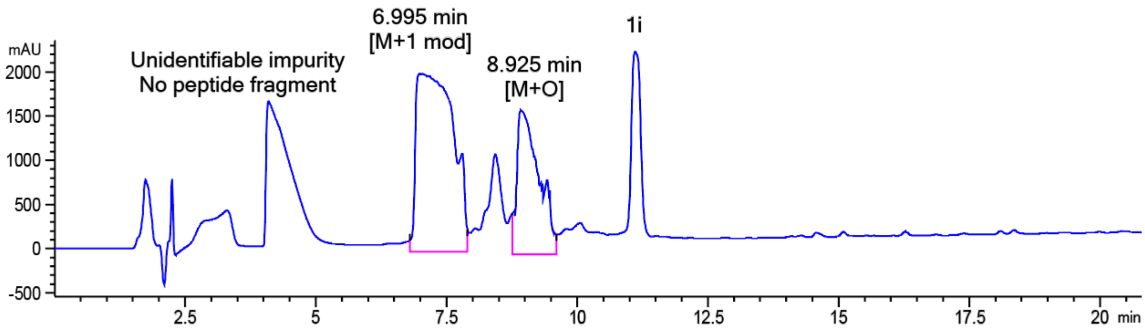

Signal 1: DAD1 A, Sig=220,4 Ref=off

Peak #	RetTime [min]	Type	Width [min]	Area [mAU*s]	Height [mAU]	Area %
1	6.995	MM	0.8261	9.97348e4	2012.19080	64.1673
2	8.925	MM	0.5254	6.60278e4	2094.67554	34.8327

HPLC trace of the **1i** modified tetracosactide acetate (***Note:*** *The peak at 4.08 min does not correspond to any peptide fragement, therefore we presume that it is coming from organic impurity*)

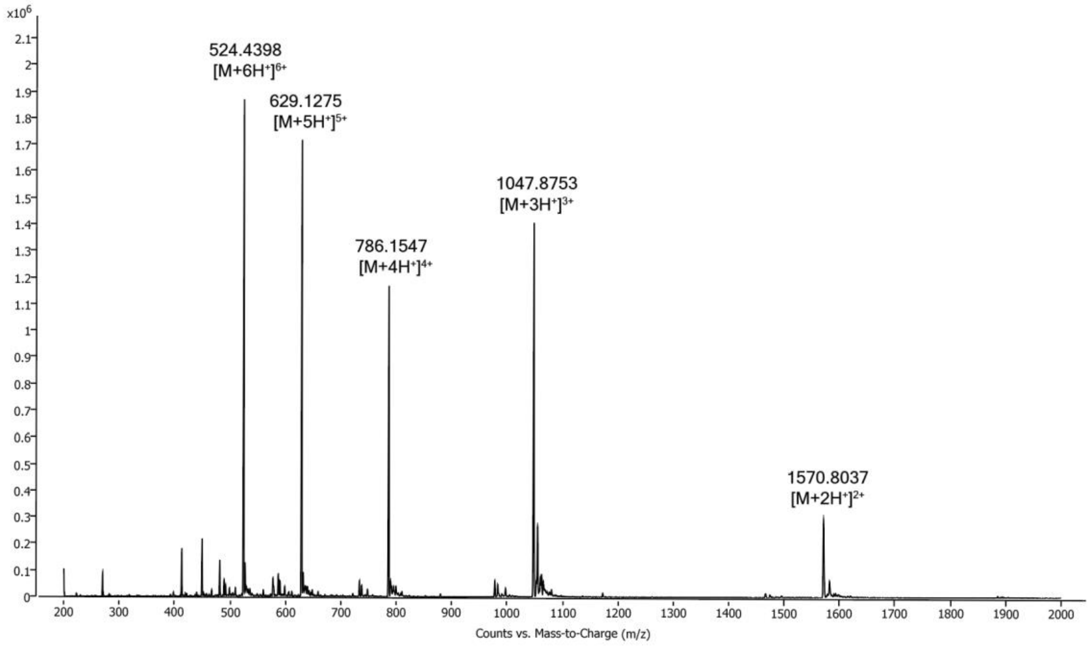

MS spectra of **1i** modified tetracosactide acetate using CuNiP

Labeling of Aviptadil Acetate with 1i using CuNiP:

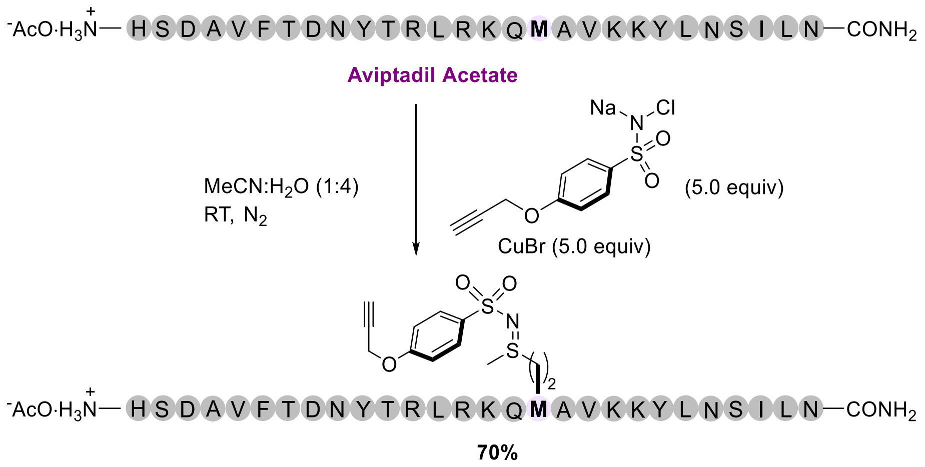

Aviptadil Acetate (2.36 mg, 0.7 µmol, 1.0 equiv), CuBr (0.5 mg, 3.5 µmol, 5.0 equiv), and **1i** (0.9 mg, 3.5 µmol, 5.0 equiv) were dissolved in MeCN:H_2O (1:4, 550 µL) and stirred at 25 °C for 3 h followed by the addition of 10 µL of 0.5 N HCl. The crude mixture was analyzed on

HPLC (Gradient: 0-70% solvent B over 30 min, solvent B: 0.1% formic acid in MeCN) and MS.

Unmodified NH_2-HSDAVFTDNYTRLRKQMAVKKYLNSILN-$CONH_2$: LCMS *m/z* 1109.6063 (calc. $[M+3H^+]^{3+}$ = 1109.6056), *m/z* 832.4547 (calc. $[M+4H^+]^{4+}$ = 832.4542), *m/z* 666.1639 (calc. $[M+5H^+]^{5+}$ = 666.1634) . Purity: > 95 % (HPLC analysis at 220 nm). Retention time in HPLC: 8.702 min.

1i modified NH_2-HSDAVFTDNYTRLRKQMAVKKYLNSILN-$CONH_2$: LCMS *m/z* 1109.2729 (calc. $[M+3H^+]^{3+}$ = 1179.2723), *m/z* 884.7057 (calc. $[M+4H^+]^{4+}$ = 884.7042), *m/z* 707.9645 (calc. $[M+5H^+]^{5+}$ = 707.9634) . Purity: > 95 % (HPLC analysis at 220 nm). Retention time in HPLC: 9.340 min.

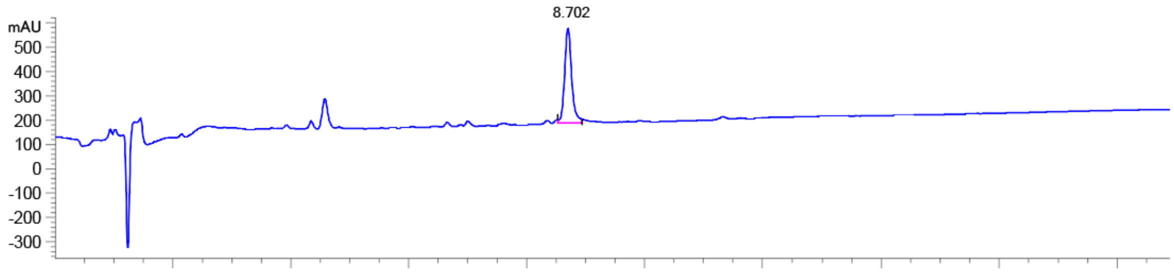

HPLC trace of aviptadil acetate

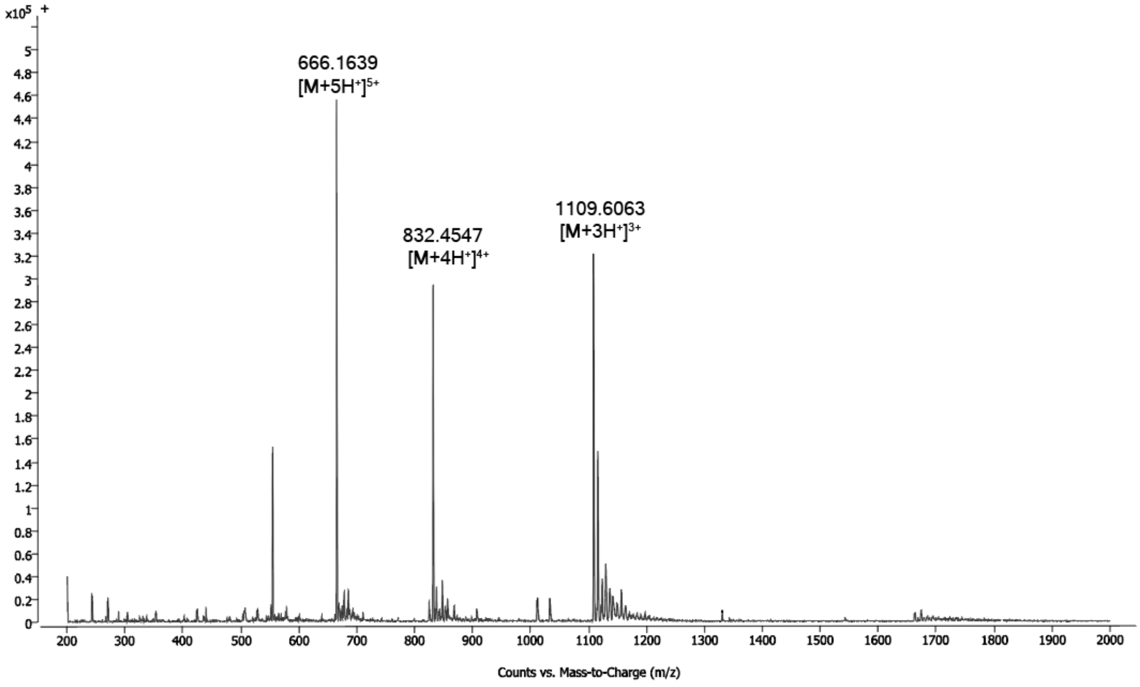

MS spectra of unmodified Aviptadil

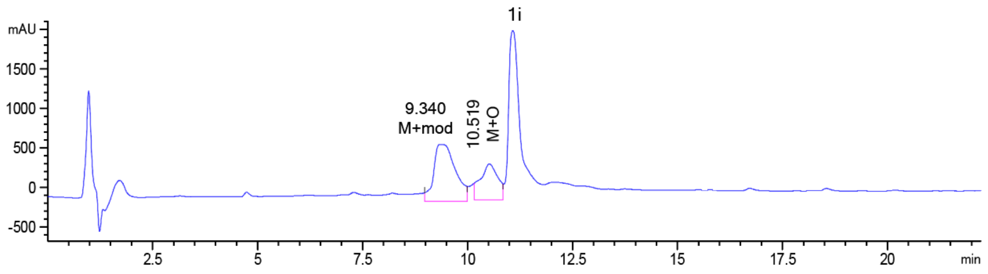

Peak #	RetTime [min]	Type	Width [min]	Area [mAU*s]	Height [mAU]	Area %
1	9.340	MM	0.6021	2.60936e4	722.32422	70.9999
2	10.519	MM	0.3917	1.06580e4	453.50769	29.0001

HPLC trace of **1i** modified Aviptadil using CuNiP

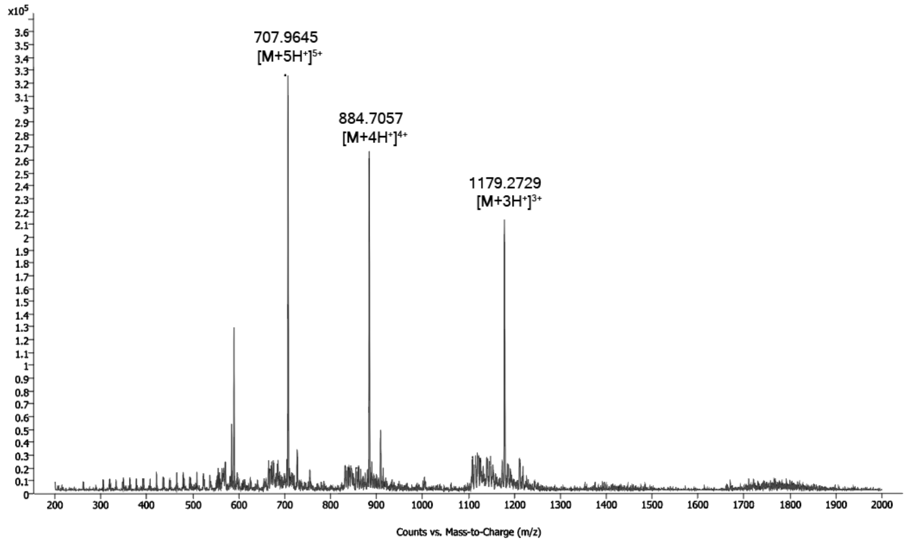

MS spectra of **1i** modified Aviptadil using CuNiP

Labeling of α-Endorphin with 1i using CuNiP:

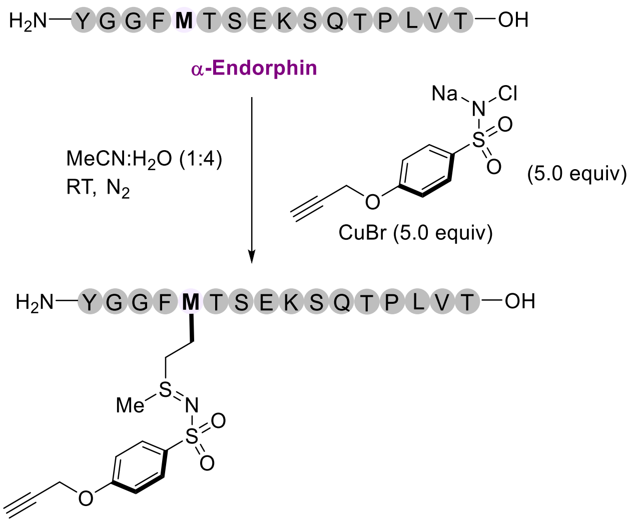

α-Endorphin (1.22 mg, 0.7 μmol, 1.0 equiv), CuBr (0.5 mg, 3.5 μmol, 5.0 equiv), and **1i** (0.9 mg, 3.5 μmol, 5.0 equiv) were dissolved in MeCN:H_2O (1:4, 550 μL) and stirred at 25 ^oC for 3 h followed by the addition of 10 μL of 0.5 N HCl. The crude mixture was analyzed on HPLC (Gradient: 0-70% solvent B over 30 min, solvent B: 0.1% formic acid in MeCN) and MS.

Unmodified α-Endorphin NH_2-YGGFMTSEKSQTPLVT-OH: LCMS *m/z* 873.4246 (calc. $[M+2H^+]^{2+}$ = 873.4243), *m/z* 1745.8149 (calc. $[M+H^+]^+$ = 1745.8415), Purity: > 99 % (HPLC analysis at 220 nm). Retention time in HPLC: 7.757 min.

1i modified α-Endorphin NH_2-YGGFMTSEKSQTPLVT-OH: LCMS *m/z* 977.9323 (calc. $[M+2H^+]^{2+}$ = 977.9317), Purity: > 99 % (HPLC analysis at 220 nm). Retention time in HPLC: 7.350 min.

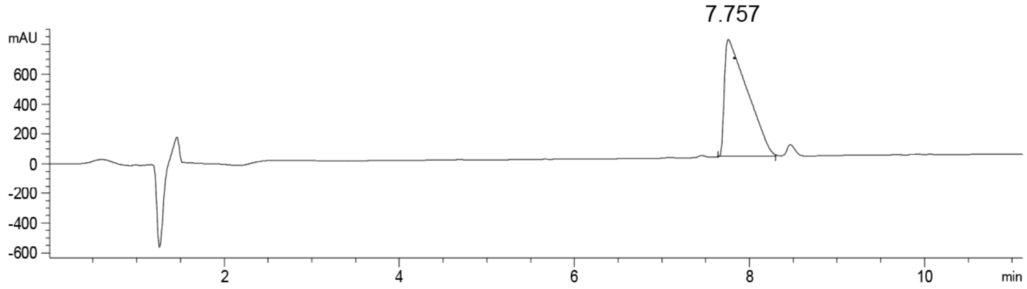

HPLC trace of unmodified α-Endorphin

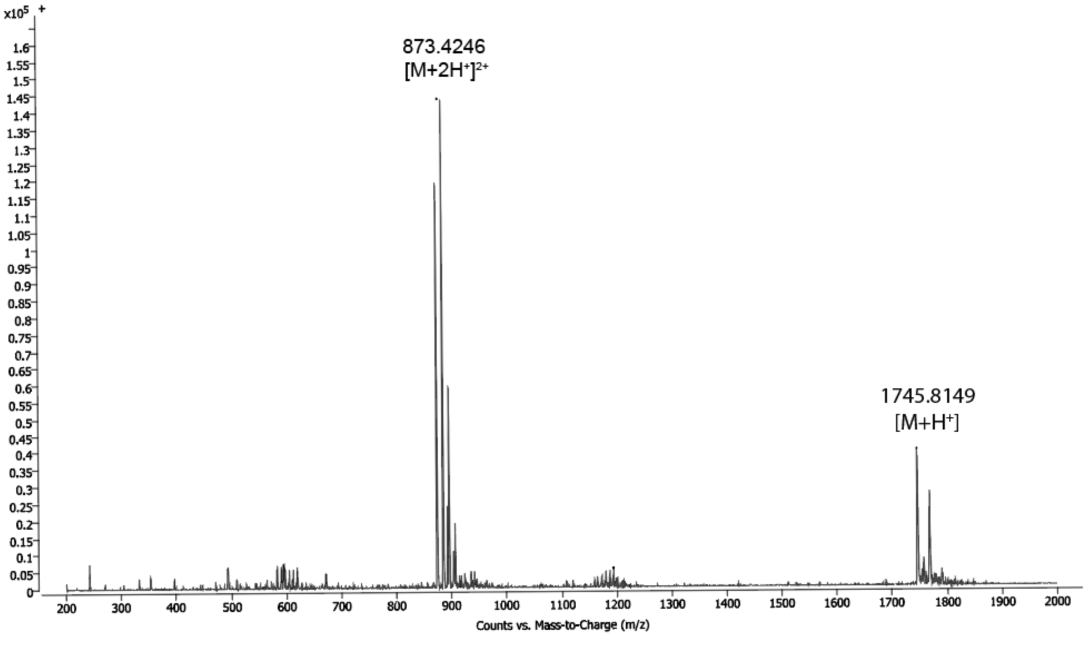

MS spectra of unmodified α-Endorphin

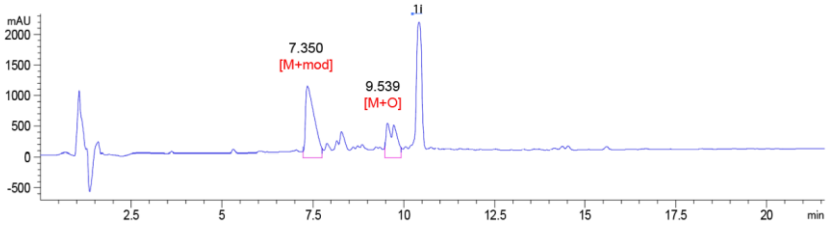

Peak #	RetTime [min]	Type	Width [min]	Area [mAU*s]	Height [mAU]	Area %
1	7.350	MM	0.2825	1.97146e4	1163.16040	65.9769
2	9.551	MM	0.3044	1.01665e4	556.55859	34.0231

HPLC trace of **1i** modified α-Endorphin using CuNiP

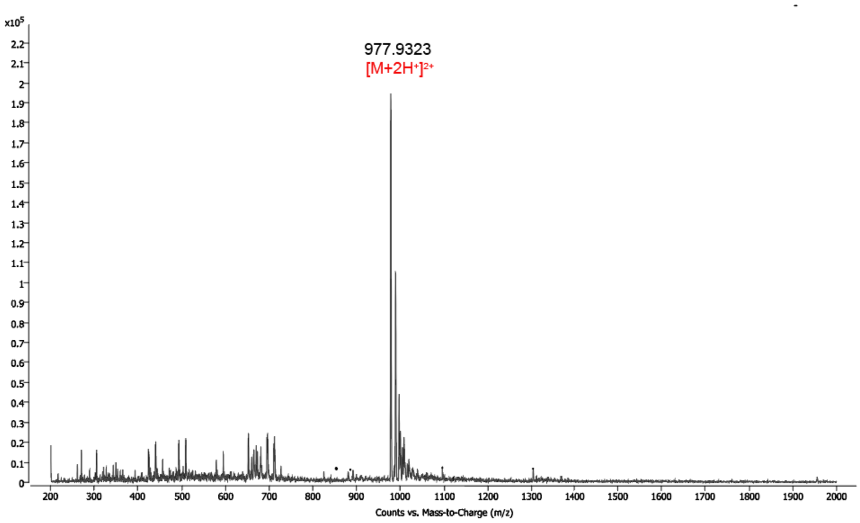

MS spectra of **1i** labeled α-Endorphin using CuNiP

Supplementary Fig. 31. CuNiP modification of proteins with azide fluorophore.

To 120 µM stock solution of proteins in MeCN:H_2O (1:4, 400 µL) was added 1.2 mM of probe **1i** and CuBr. The reaction was stirred at room temperature for 2 h. Samples were filtered using a 3 kDa molecular weight cut-off filter to obtain pure proteins. **1i** labeled proteins were dissolved in 100 µL of water, followed by the addition of 50 µL of 100 mM TBTA in water, 50 µL of 100 mM freshly prepared ascorbic acid in water, 50 µL of 50 mM of $CuSO_4$ in water, and 2 µL of 10 mM Cy5 azide in DMSO. The reaction was stirred for 1 h and filtered with a 3kDa filter, followed by analysis of proteins through in gel fluorescence imaging and Coomassie blue staining. Samples were loaded on a Novex WedgeWell 4-20% Tris-Glycine gel. Gel was run in Tris-glycine running buffer at 180V. The gel was then stained with Coomassie brilliant blue for 1 h and destained overnight. Uncropped gel data is attached as source data.

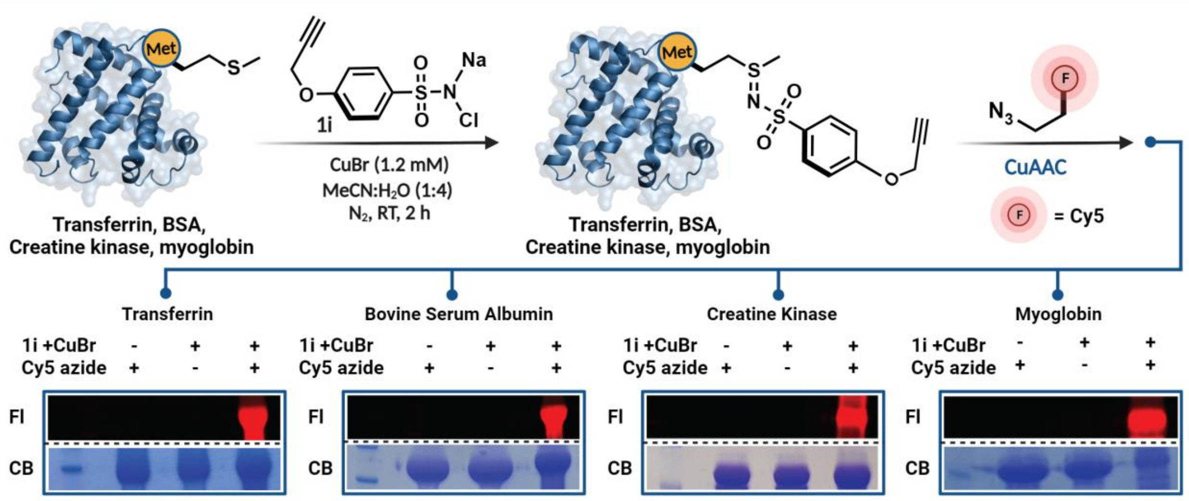

Supplementary Fig. 31 was created with BioRender.com, released under a Creative Commons Attribution-NonCommercial-NoDerivs 4.0 International license".

Supplementary Fig. 32. CuNiP modification of cell lysate with azide fluorophore

Cell Culture and Drugs. Cells were maintained at 37 °C and 5% CO_2. T47D cells were cultured in RPMI supplemented with 10% (V/V) fetal bovine serum (FBS) and 1% (V/V) penicillin/streptomycin (100 µg/mL).

Cell Lysis. Whole cell lysate was generated by lysing cells on ice in RIPA buffer (50 mM TrisHCl [pH 8], 150 mM NaCl, 1% NP-40, 0.5% sodium deoxycholate, 0.1% SDS) supplemented with protease and phosphatase inhibitors. Lysates were centrifuged 6,500 x g, 10 m at 4°C, and soluble lysate was collected. Whole cell lysate proteins were separated using 16% SDS-PAGE. SDS-PAGE gels were stained with Coomassie brilliant blue dye.

Dose-dependent CuNiP modification of lysates and conjugation with Cy5 azide fluorophore. To 4 tubes (individual reactions) of 100 µg of lysate in degassed MeCN:H_2O (1:4, 400 µL) were treated with freshly prepared 50 µM, 100 µM, 150 µM, and 200 µM CuBr that had been re-suspended in 50 µL of acetonitrile. To the 4 samples were added freshly prepared 50 µM, 100 µM, 150 µM, and 200 µM probe **1i** that had been re-suspended in 100 µL of NaP buffer pH 7. The reaction was stirred at room temperature for 3 h. Upon completion of reaction, samples were acetone precipitated, followed by Cy5 azide fluorophore labelling. Proteins were dissolved in 100 µL of water, followed by the addition of 50 µL of 100 mM TBTA in water, 50 µL of freshly prepared 100 mM ascorbic acid in water, 50 µL of 50 mM of CuSO4 in water, and 2 µL of 10 mM Cy5 azide in DMSO. The reaction was stirred for 1 h and acetone precipitated, followed by analysis of proteins through in gel fluorescence imaging and Coomassie blue staining. Samples were loaded on a Novex WedgeWell 4-20% Tris-Glycine gel. Gel was run in Tris-glycine running buffer at 180V. The gel was then stained with Coomassie brilliant blue for 1 h and destained overnight. Uncropped gel data is attached as source data.

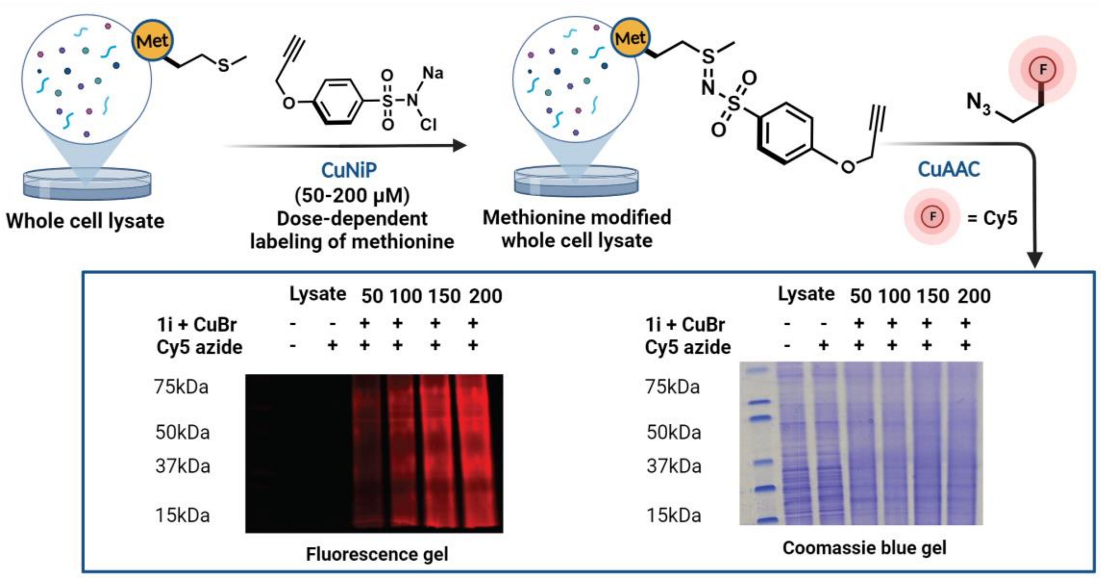

Supplementary Fig. 32 was created with BioRender.com, released under a Creative Commons Attribution-NonCommercial-NoDerivs 4.0 International license".

Supplementary Fig. 33. Optimization of Cell Lysate Chemoproteomics analysis using 1a

CuNiP reaction on cell lysate: Using the optimized reaction conditions describe above, whole cell lysate sample was modified using 150 µM of **1a** and CuBr. Upon completion of reaction, modified lysate samples were acetone precipitated and digested using SMART Digest™ Trypsin Kit by Thermo Scientific. This experiment was performed with (n= 1 biological sample).

LC-MS/MS. Digested sample was analyzed by nano LC-MS/MS with a Waters M-Class HPLC system interfaced to a ThermoFisher Fusion Lumos mass spectrometer. Peptides were loaded on a trapping column and eluted over a 75µM analytical column at 350nL/min; both columns were packed with Luna C18 resin (Phenomenex). A 1 h gradient was employed. The mass spectrometer was operated in data-dependent mode, with the Orbitrap operating at 60,000 FWHM and 15,000 FWHM for MS and MS/MS respectively. APD was enabled and the instrument was run with a 3s cycle for MS and MS/MS.

Database search (Proteome Discoverer). Mass spectrometry data was analyzed according to a published protocol.3 Spectra were searched using Proteome Discoverer 2.1 against 2020 human UniProtKB/Swiss-Prot database (20,379 target sequences). Searching parameters included fully tryptic restriction, precursor mass tolerance (± 20 ppm), and fragment mass tolerance (± 0.05 Da). Methionine oxidation (+15.99492 Da), asparagine and glutamine deamidation (+0.98402 Da) and protein N-terminal acetylation (+42.03670) were variable modifications (up to 3 allowed per peptide); cysteine was assigned a fixed carbamidomethyl modification (+57.021465 Da). Percolator was used to filter the peptide spectra matches (PSMs) to a false discovery rate of 1%.

Excel sheet of analysis is attached supplementary data 1. The mass spectrometry proteomics data (Data 1) generated in this study have been deposited to the ProteomeXchange Consortium via the PRIDE partner repository with the dataset identifier **PXD051224.**

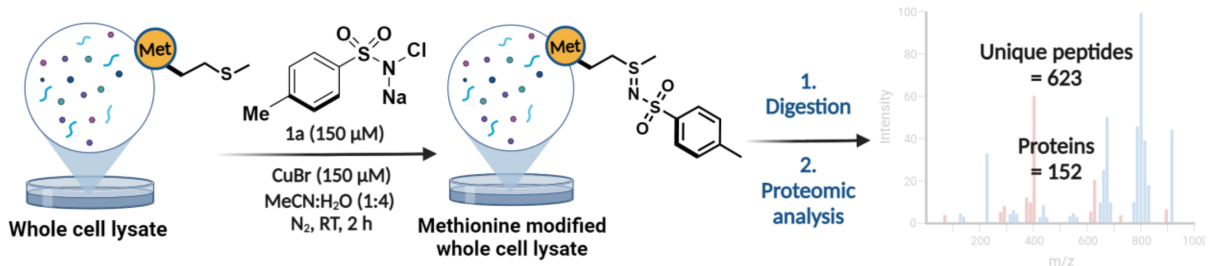

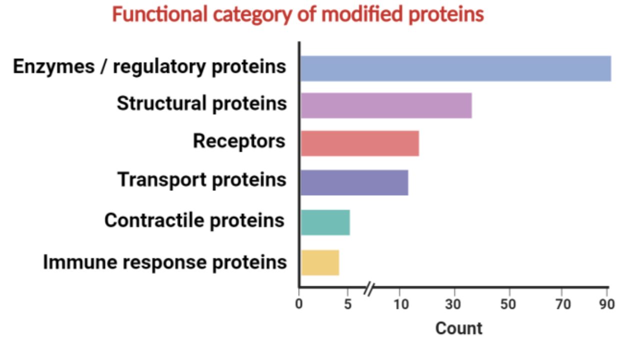

Most significant modifications

Accession	Proteins names
P60709	Actin, cytoplasmic 1
P68104	Elongation factor 1-alpha 1
Q09666	Neuroblast associated protein
P07900	Heat shock protein HSP 90-alpha
P49327	Fatty acid synthase
P06733	Alpha-enolase
P08238	Heat shock protein HSP 90-beta
Q5VTE0	Elongation factor 1-alpha-like 3
Q71U36	Tubulin alpha-1A chain
P07437	Tubulin beta chain

Supplementary Fig. 33 was created with BioRender.com, released under a Creative Commons Attribution-NonCommercial-NoDerivs 4.0 International license".

Supplementary Fig. 34. Dose-dependent Cell Lysate Chemoproteomics analysis using probe 1i

Dose-dependent CuNiP modification of lysates and proteomics analysis. To 4 tubes (individual reactions) of 100 µg of lysate in 400 µL of MeCN:H_2O (1:4) were treated with freshly prepared solution of CuBr and **1i** (1 µM for super low dose, 10 µM for low dose, 50 µM for medium dose, and 250 µM for high dose). The reaction was stirred at room temperature for 2 h. The proteins were acetone precipitated, followed by digestion using SMART Digest™ Trypsin Kit by Thermo Scientific. This experiment was performed with (n= 1 biological sample).

LC-MS/MS. Digested samples were resuspended in Buffer A (0.1% FA in water) and the peptide amount was determined by Pierce™ Quantitative Peptide Assays & Standards (Thermo Fisher Scientific) according to manufactures instructions. Samples were injected into a nanoElute UPLC autosampler (Bruker Daltonics) coupled to a timsTOF Pro2 mass-spectrometer (Bruker Daltonics). The peptides were loaded on a 25 cm Aurora ultimate CSI C18 column (IonOpticks) and chromatographic separation was achieved using a linear gradient starting with a flow rate of 250 nl/min from 2% Buffer B (0.1% FA in MeCN) and increasing to 13% in 42 min, followed by an increase to 23% B in 65 min, 30% B in 70 min, then the flow rate was increased to 300 nl/min and 80% B in 85 min, this was kept for 5 min.

The mass-spectrometer operated in positive polarity for data collection using a data-dependent acquisition (ddaPASEF) mode. The cycle time was 1.17 s and consisted of one full scan followed by 10 PASEF/MSMS scans. Precursors with intensity of over 2500 (arbitrary units) were picked for fragmentation and precursors over the target value of 20,000 were dynamically excluded for 1 min. Precursors below 700 Da were isolated with a 2 Th window and ones above with 3 Th. All spectra were acquired within an m/z range of 100 to 1700 and fragmentation energy was set to 20 eV at 0.6 1/K0 and 59 eV at 1.60 1/K0.

Database search (MSFragger).

MS raw files were searched FragPipe GUI version 20 with MSFragger (version 3.8) as the search algorithm. Protein identification was performed with the human Swisspot database (20′456 entries) with acetylation (N-terminus), and oxidation on methionine was set variable modification. To account for the mass shift introduced by the different chemical handles a variable mass shift of 209.0147 Da or 169.0197 Da on Methionine with a maximal occurrence of 3 for the Alkyne or Methyl, respectively, was set. Carbamidomethylation of cysteine residues was considered a fixed modification. Trypsin was set as the enzyme with up to two missed cleavages. The peptide length was set to 7–50, and the peptide mass range of 500–5000 Da. For MS2-based experiments, the precursor tolerance was set to 20 ppm and fragment tolerance to 20 ppm. Peptide spectrum matches (PSMs) were adjusted to a 1% false discovery rate using Percolator as part of the Philosopher toolkit (v5). For label-free quantification, match-between-runs were enabled. All downstream analysis was performed in R (version 2023.03.0). Individual samples were normalized to the mean of all quantified peptides.

Excel sheet of analysis is attached supplementary data 2. The mass spectrometry proteomics data (Data 2) generated in this study have been deposited to the ProteomeXchange Consortium via the PRIDE partner repository with the dataset identifier **PXD051224**

Chemoselectivity of CuNiP on a proteome-wide scale

To assess the selectivity for methionine, we performed a database search with the modification on Met, Lys, Gln, Tyr, Cys, Arg, Asp, His, and Ser. Although a limited number of modified peptides can be identified when searching for other amino acids, the minimum specificity at 250 µM is 3.95-fold (554 peptides for Met compared to 140 peptides for Ser). We believe these observed modifications are artifacts as we do not observe any reactivity of CuNiP with cysteine or other reactive residues at peptide and recombinant protein examples. This experiment was performed with (n= 1 biological sample).

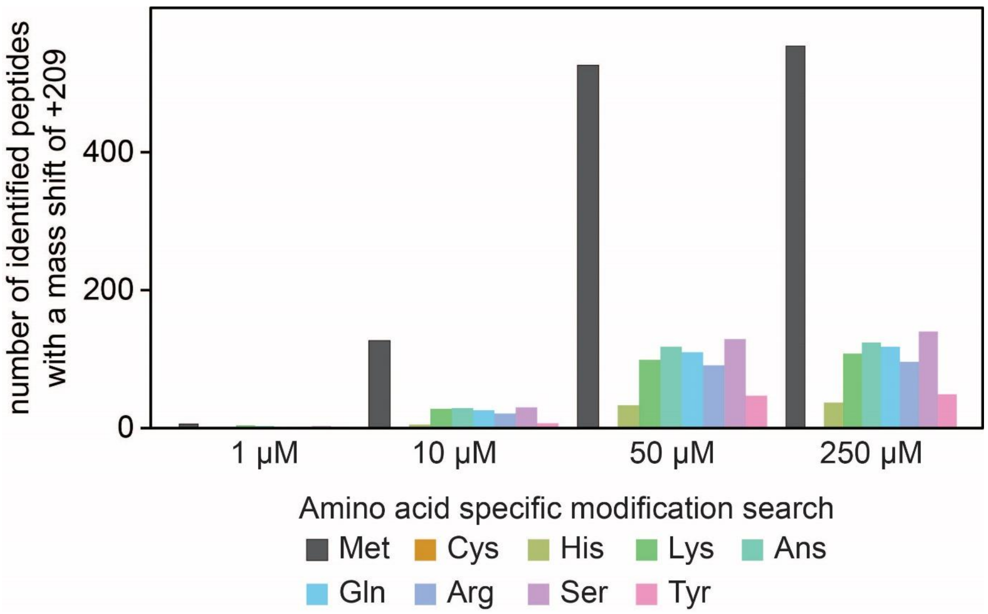

Heatmap analysis of hyperreactive methionine sites

Heatmap analysis of the dose-dependent labelling of lysates with CuNiP reagent identified peptides within cluster 11 to possess hyperreactive methionine residues as intensity of modified peptides remained relatively saturated as concentration of CuNiP reagent increased.

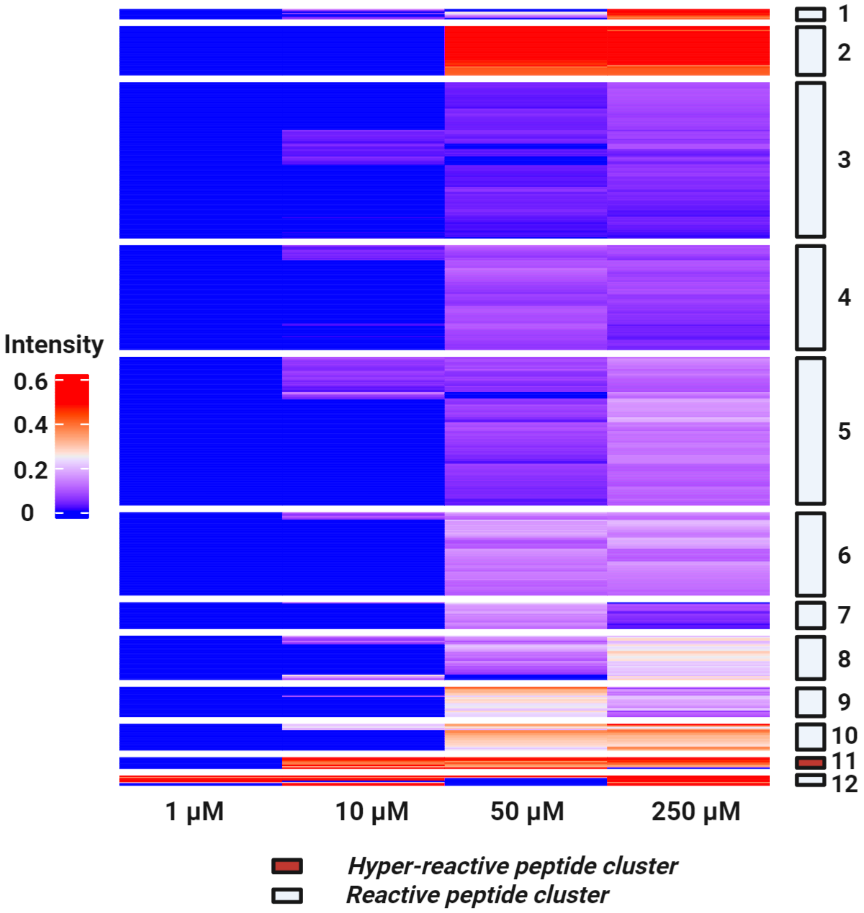

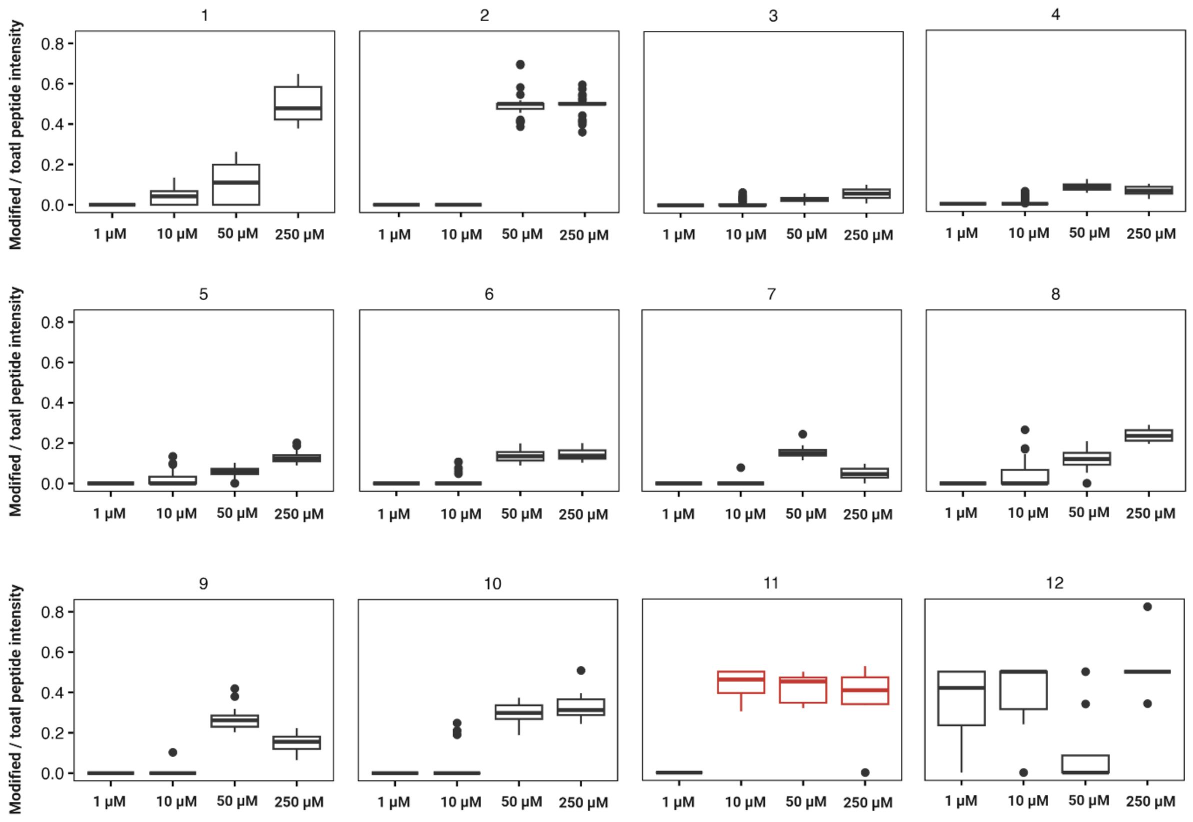

Center line—median; box limits contain 50% of data; upper and lower quartiles, 75 and 25%; maximum—greatest value excluding outliers; minimum—least value excluding outliers; outliers—more than 1.5 times of the upper and lower quartiles

Solvent-Accessible Surface Area (SASA) of modified methionine sites

200 randomized peptide sequences were taken from the high-dose (250 µM) sample . Solvent accessible surface area was calculated using Geterea online platform (https://curie.utmb.edu/getarea.html). For proteins that does not have PDBs, a similar AlphaFold structure were chosen for SASA calculation. Radius of the water probe was chosen as 1.4 Å. Gradient in calculation was set to n. Desired level of output was kept as Area/energy per residue. The excel sheet is attached along with the SASA calculation.

Gene Ontology (GO) analysis of hyperreactive methionine sites

To evaluate the biological processes and localization of modified proteins, Gene ID of CuNiP modified protein targets were extracted followed by GO analysis using ShinyGO 0.77. FDR cut-off was set at 0.05%.

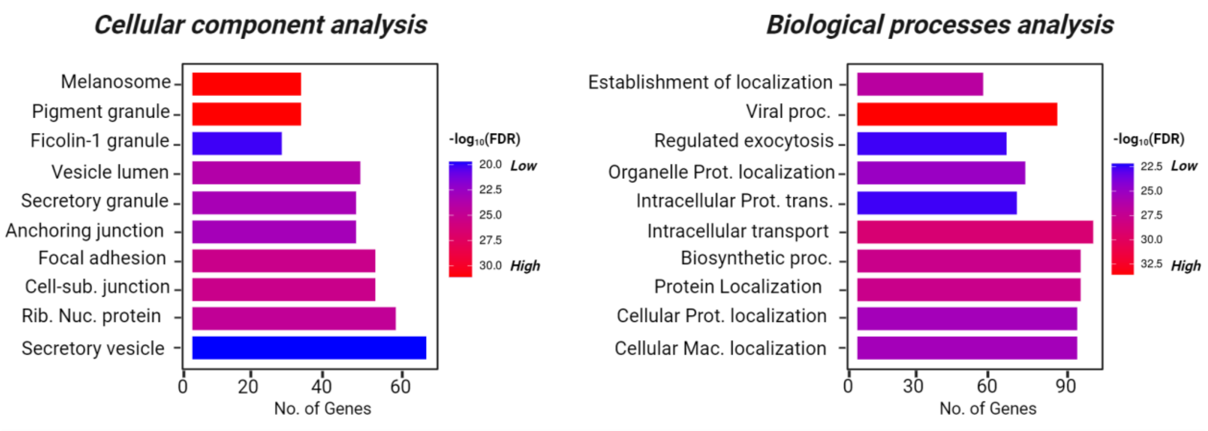

Sequence Motif analysis

Sequence motif of modified methionine sites: To identify the sequence motif of modified methionine sites, Excel list containing the CuNiP modified methionine sites were utilized. Sequences containing 4 residues from the left and 4 residues from the right of modified methionine sites were utilized, with methionine as the fixed positions. Sequence motif was generated using "probability logo generator for biological sequence motif " plogo v1.2.0

Supplementary Fig. 35. CuNiP mediated profiling of oxidation sensitive methionine.

Dose-dependent H_2O_2 treatment and CuNiP modification of lysates and in-gel fluorescence analysis. To 4 tubes (individual reactions) of 100 µg of lysate in 100 µL of PBS were treated with freshly prepared solution of H_2O_2 (0.5 mM, 1 mM, 2 mM). The reaction was stirred at room temperature for 1 h. The proteins were acetone precipitated, followed by resuspension of proteins into 400 µL of MeCN:H_2O (1:4). Proteins were incubated with 250 µM of 1i and CuBr at room temperature for 2 h. Control samples without H_2O_2 was generated by not t incubating lysates with H_2O_2. CuNip modified proteins were treated with Cy5 azide using CUAAC to attach cy5 azide followed by in-gel fluorescence analysis. This experiment was performed with (n= 1 biological sample). Uncropped gel data is attached as source data.

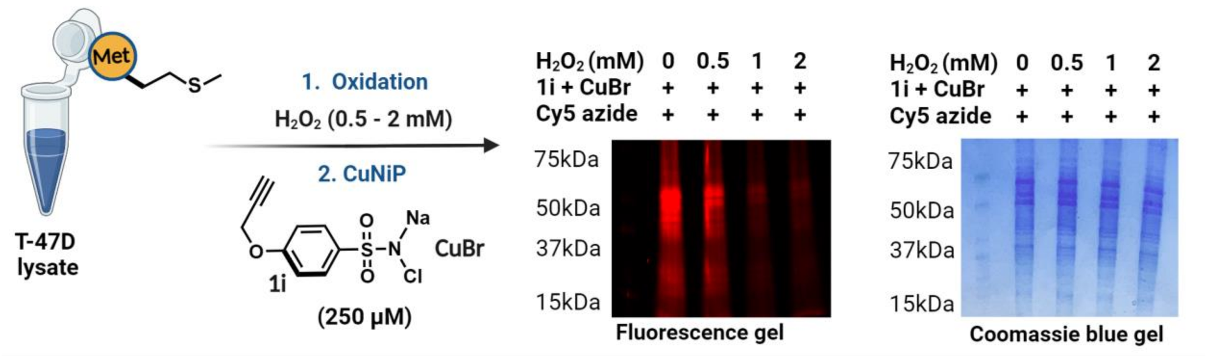

Supplementary Fig. 35 was created with BioRender.com, released under a Creative Commons Attribution-NonCommercial-NoDerivs 4.0 International license".

Dose-dependent H_2O_2 treatment and CuNiP modification of lysates and proteomics analysis.

Digestion of 50 µg (H_2O_2 + **1i**) treated and 50 µg of **1i** treated control samples (above) and proteomics analysis showed a dose-dependent decrease in **1i** labelling as H_2O_2 concentration increases. Furthermore, evaluating the effect of H_2O_2 concentration on modified methionine sites in control samples clearly showed a reduction in **1i** modified peptides as H_2O_2 concentration increased (356 peptides-control; 267 peptides-0.5 mM; 178 peptides- 1 mM; 115 peptides- 2 mM) with (0.5 mM of H_2O_2, 88 PSMs < control; 1 mM of H_2O_2, 177 PSMs < control; 0.5 mM of H_2O_2, 240 PSMs < control). This experiment was performed with (n= 1 biological sample).

Excel sheet of analysis is attached supplementary data 3. The mass spectrometry proteomics data (Data 4) generated in this study have been deposited to the ProteomeXchange Consortium via the PRIDE partner repository with the dataset identifier **PXD051224**

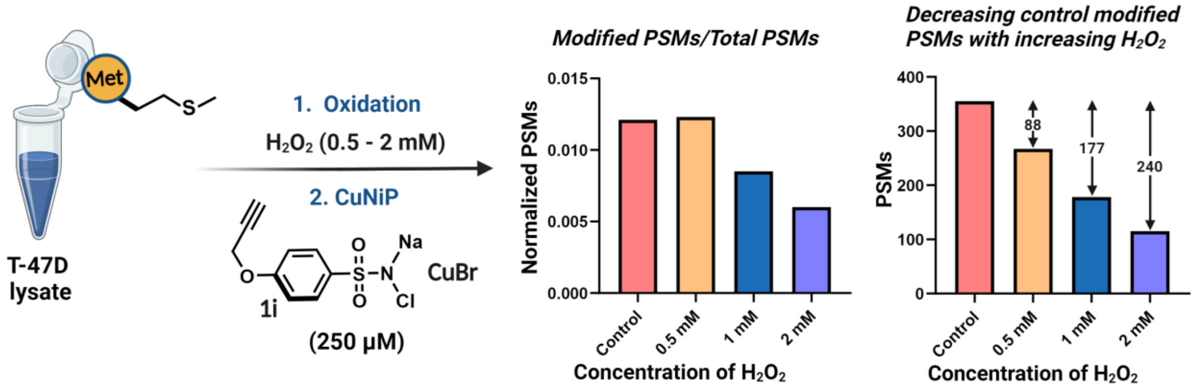

Supplementary Fig. 35 was created with BioRender.com, released under a Creative Commons Attribution-NonCommercial-NoDerivs 4.0 International license".

GO analysis (biological processes)

GO analysis of oxidation sensitive methionine residues showed a significant enrichment of nucleic acid metabolism and regulatory proteins. GO analysis was done using ShinyGO 0.77. FDR cut-off was set at 0.05%.

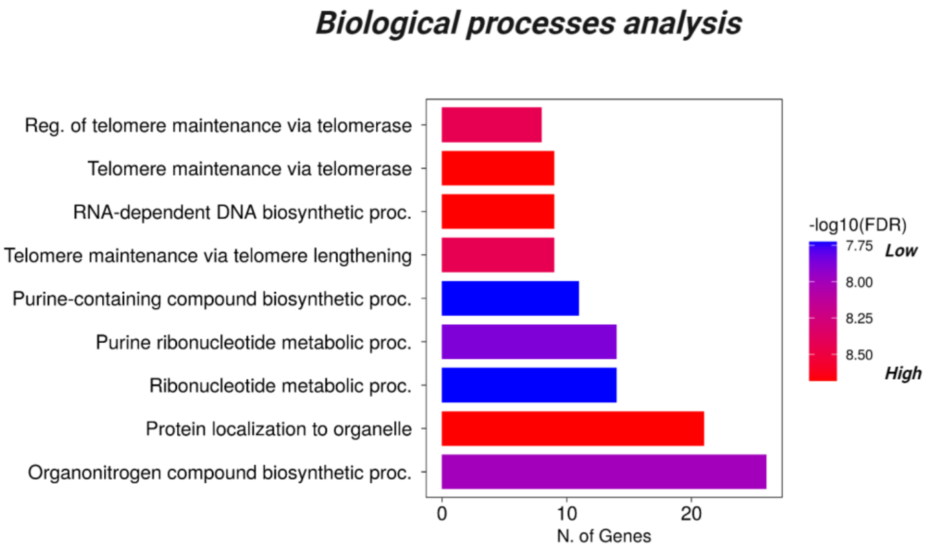

Supplementary Fig. 36. Cell viability studies.

Gating Strategy

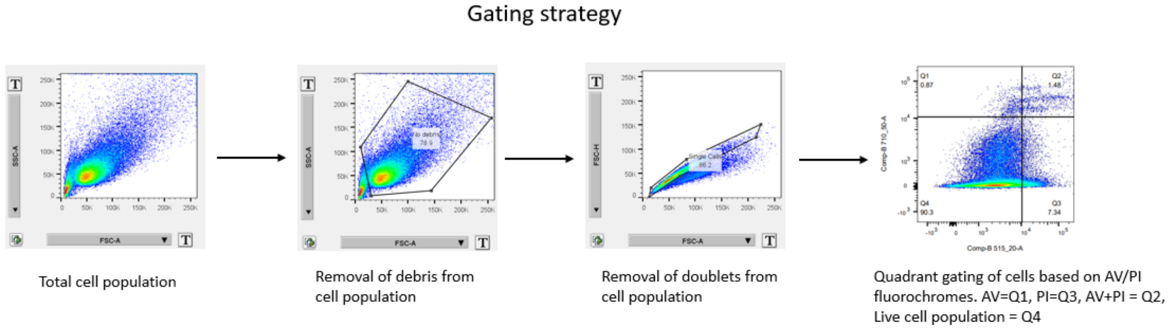

Flow cytometry analysis of cell death by probe 1i: T47D cells were treated with probe 1i (100 µM to 2 mM) for 2 h. After incubation, cells were washed with PBS, detached with trypsin, and stained with Annexin V/PI, according to manufacturer's protocol. Annexin V (AV) conjugated to FITC was used to determine apoptosis and propidium iodide (PI) was used to determine necrosis within the cell population. Cells were analyzed via flow cytometry within 1 h to quantify cell death. FlowJo software (version 10.8.1) was used to analyze data collected on the cytometer. PI and AV controls were used to determine quadrant placement. All the experiments were performed duplicates (n= 2 biological replicates). Data are represented as mean ± SD.

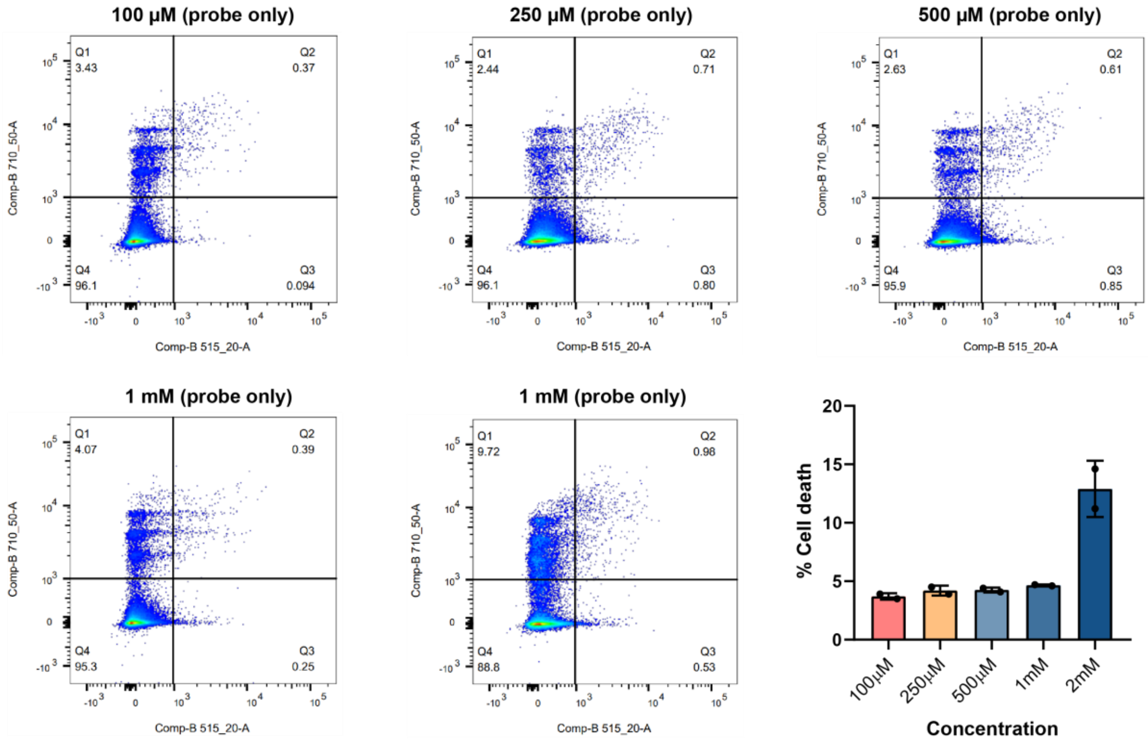

Flow cytometry analysis of cell death by CuBr: T47D cells were treated with CuBr (100 µM to 2 mM) for 2 h. After incubation, cells were washed with PBS, detached with trypsin, and stained with Annexin V/PI, according to manufacturer's protocol. Annexin V (AV) conjugated to FITC was used to determine apoptosis and propidium iodide (PI) was used to determine necrosis within the cell population. Cells were analyzed via flow cytometry within 1 h to quantify cell death. FlowJo software (version 10.8.1) was used to analyze data collected on the cytometer. PI and AV controls were used to determine quadrant placement. All the experiments were performed duplicates (n= 2 biological replicates). Data are represented as mean ± SD.

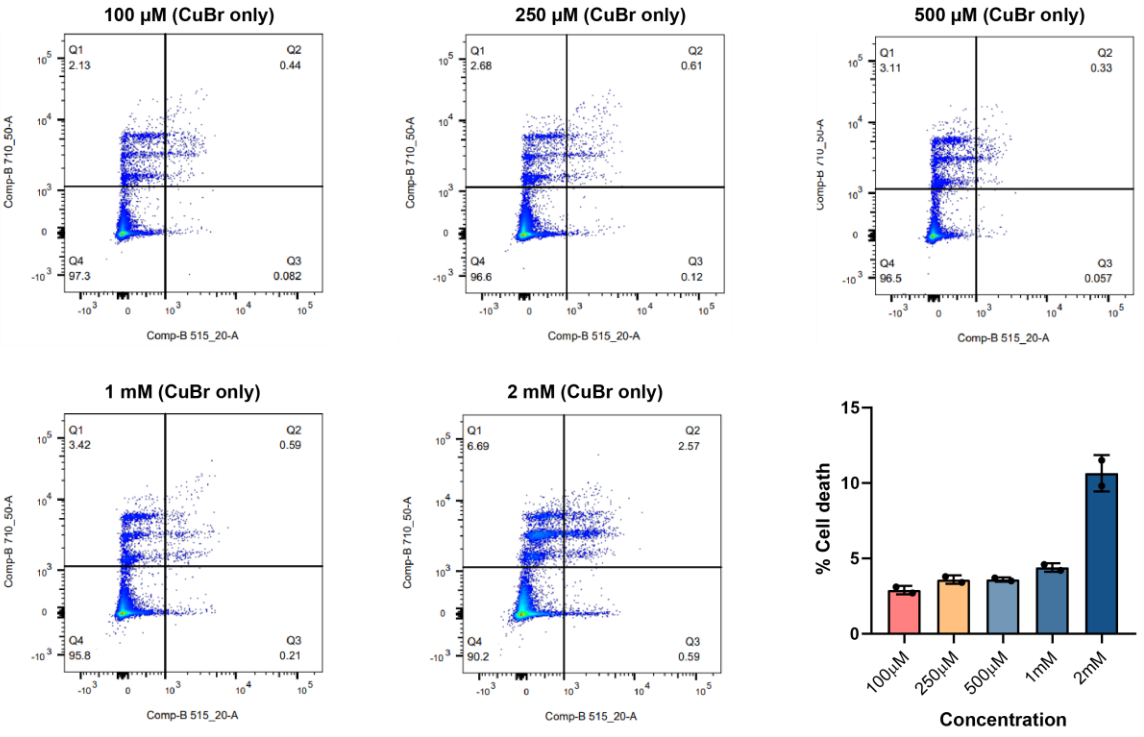

Flow cytometry analysis of cell death by Acetonitrile (MeCN): T47D cells were treated with MeCN (0.1% to 2%) for 2 h. After incubation, cells were washed with PBS, detached with trypsin, and stained with Annexin V/PI, according to manufacturer's protocol. Annexin V (AV) conjugated to FITC was used to determine apoptosis and propidium iodide (PI) was used to determine necrosis within the cell population. Cells were analyzed via flow cytometry within 1 h to quantify cell death. FlowJo software (version 10.8.1) was used to analyze data collected on the cytometer. PI and AV controls were used to determine quadrant placement. All the experiments were performed in duplicates (n= 2 biological replicates). Data are represented as mean ± SD.

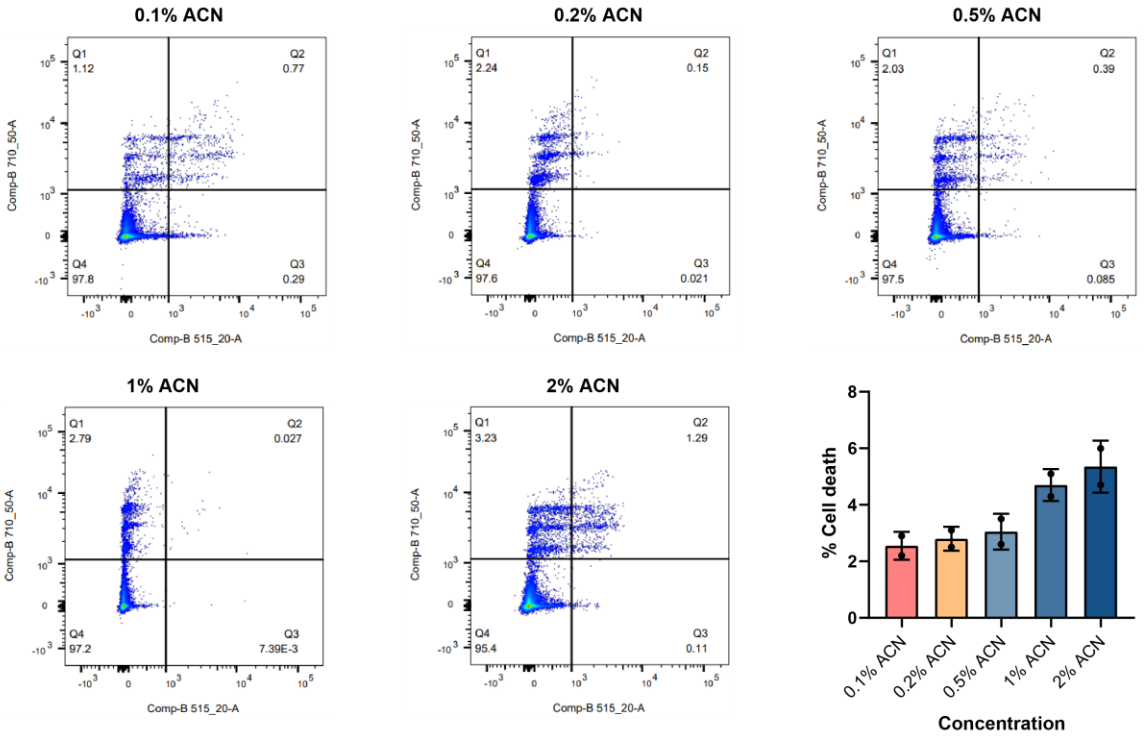

Flow cytometry analysis of cell death by Probe 1i, CuBr, and 1% MeCN: T47D cells were treated with probe **1i** (100 µM to 2 mM), CuBr (100 µM to 2 mM), and 1% acetonitrile for 2 h. After incubation, cells were washed with PBS, detached with trypsin, and stained with Annexin V/PI, according to manufacturer's protocol. Annexin V (AV) conjugated to FITC was used to determine apoptosis and propidium iodide (PI) was used to determine necrosis within the cell population. Cells were analyzed via flow cytometry within 1 h to quantify cell death. FlowJo software (version 10.8.1) was used to analyze data collected on the cytometer. PI and AV controls were used to determine quadrant placement. All the experiments were performed in duplicates (n= 2 biological replicates). Data are represented as mean ± SD.

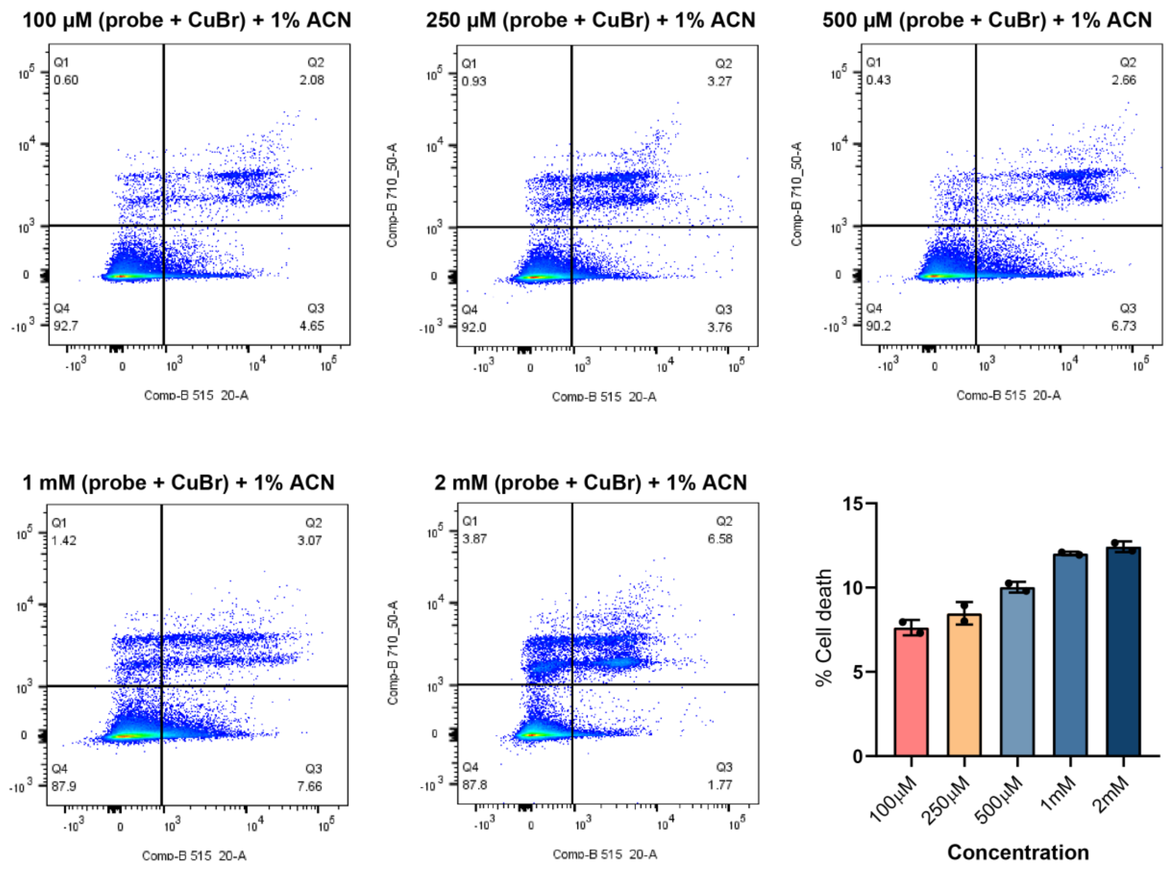

Supplementary Fig. 37. CuNiP reaction on live cells

Dose-dependent fluorophore labelling and proteomic analysis of live cells.

Live T47D cells were plated on 6 cm petri dishes supplemented with RPMI media and incubated for 24 h. Cells were then treated with probe **1i** (100 µM to 2 mM), CuBr (100 µM to 2 mM), and 1% acetonitrile for 2 h. After 2 h, cells were washed 3 times with cold PBS and lysed using RIPA buffer (50 mM Tris HCl [pH 8], 150 mM NaCl, 1% NP-40, 0.5% sodium deoxycholate, 0.1% SDS) supplemented with protease and phosphatase inhibitors. Lysates were centrifuged 6,500 x g, 10 m at 4°C, and soluble lysate was collected. To 100 µg of lysate in 100 µL of PBS buffer were treated with 50 µL of 100 mM TBTA in water, 50 µL of freshly prepared 100 mM ascorbic acid in water, 50 µL of 50 mM of $CuSO_4$ in water, and 2 µL of 10 mM Cy5 azide in DMSO. The reaction was stirred for 1 h and acetone precipitated, followed by analysis of proteins through in gel fluorescence imaging and Coomassie blue staining. Samples were loaded on a Novex WedgeWell 4-20% Tris-Glycine gel. Gel was run in Tris-glycine running buffer at 180V. The gel was then stained with Coomassie brilliant blue for 1 h and destained overnight. For proteomics analysis, 100 µg of live-cell derived lysates were digested using SMART Digest™ Trypsin Kit by Thermo Scientific. Proteomics analysis was done according to the general protocol described above. This experiment was repeated (n= 3 biological sample) with similar results. Uncropped gel data is attached in the source data section below.

Excel sheet of analysis is attached supplementary data 4. The mass spectrometry proteomics raw data generated in this study (Data 3) have been deposited to the ProteomeXchange Consortium via the PRIDE partner repository with the dataset identifier **PXD051224**

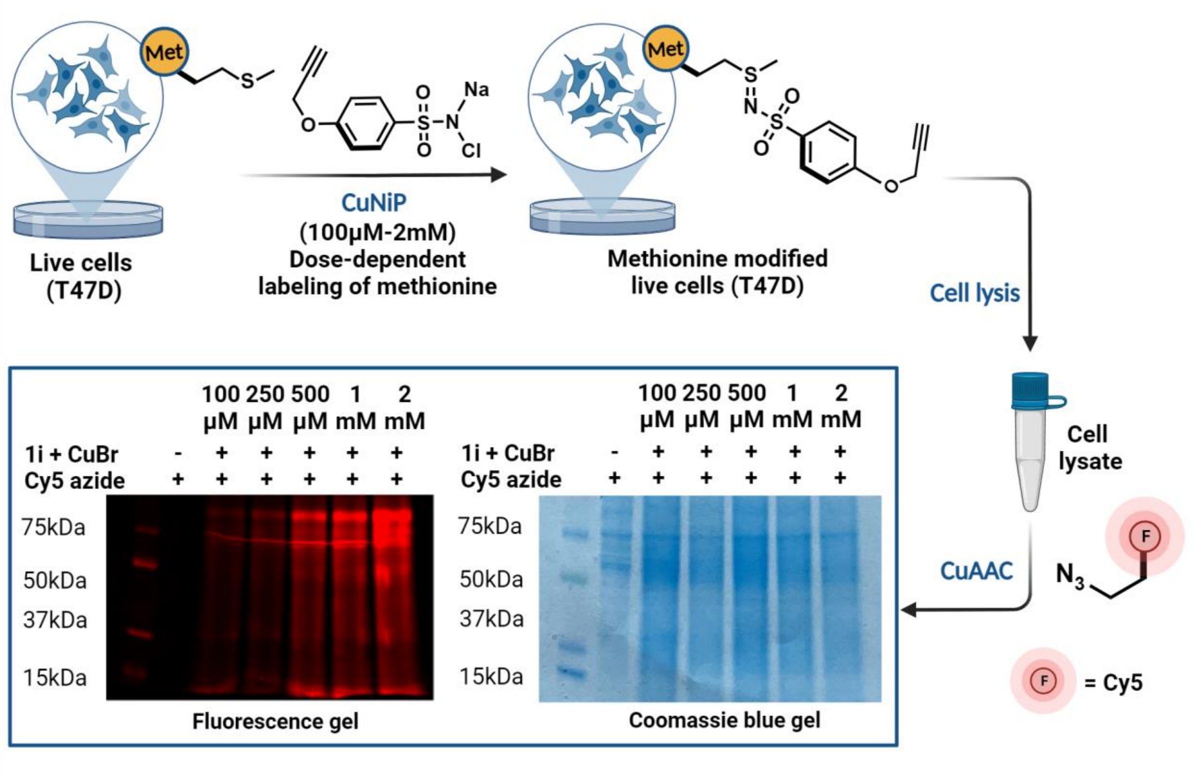

Supplementary Fig. 37 was created with BioRender.com, released under a Creative Commons Attribution-NonCommercial-NoDerivs 4.0 International license".

Supplementary Fig. 38. Confocal microscopy imaging of CuNiP labeled T47D cells.

Live T47D cells were plated on microscope slides in a 6 cm petri dish supplemented with RPMI media and incubated for 24 h. Cells were then treated with probe **1i** (100 µM to 2 mM), CuBr (100 µM to 2 mM), and 1% acetonitrile for 2 h. After 2 h, cells were washed 3 times with cold PBS and fixed in 4% formaldehyde solution for 10 min. Cells were subsequently washed 3 times with PBS (5 min) and permeabilized with freshly prepared 0.1% Triton-X solution in PBS. Alkyne labeled proteins within cell were labeled with cy5 azide fluorophore using click chemistry. For labelling through click chemistry, fixed and permeabilized cells were incubated in 5 mL of PBS followed by the addition of 50 µL of 100 mM TBTA in water, 50 µL of freshly prepared 100 mM ascorbic acid in water, 50 µL of 50 mM of $CuSO_4$ in water, and 1 µL of 10 mM Cy5 azide in DMSO. The reaction was stirred for 1 h and washed 3 times with PBS. Nuclear staining of cells was done with Fluoroshield-DAPI mounting media and subsequently imaged on a Leica SP8 confocal microscope. The images were processed and analyzed using ImageJ software to determine relative labelling of cells. For quantification >50 cells were used for control and experimental samples. A z-stack image of median intensity image from 22 slices

of CuNiP modified cells are reported below. From these recent images, we can observe that most of the modifications were at the cell membrane while few modifications were within the nuclear region of the cells. This experiment was repeated (n= 2 biological sample) with similar results.

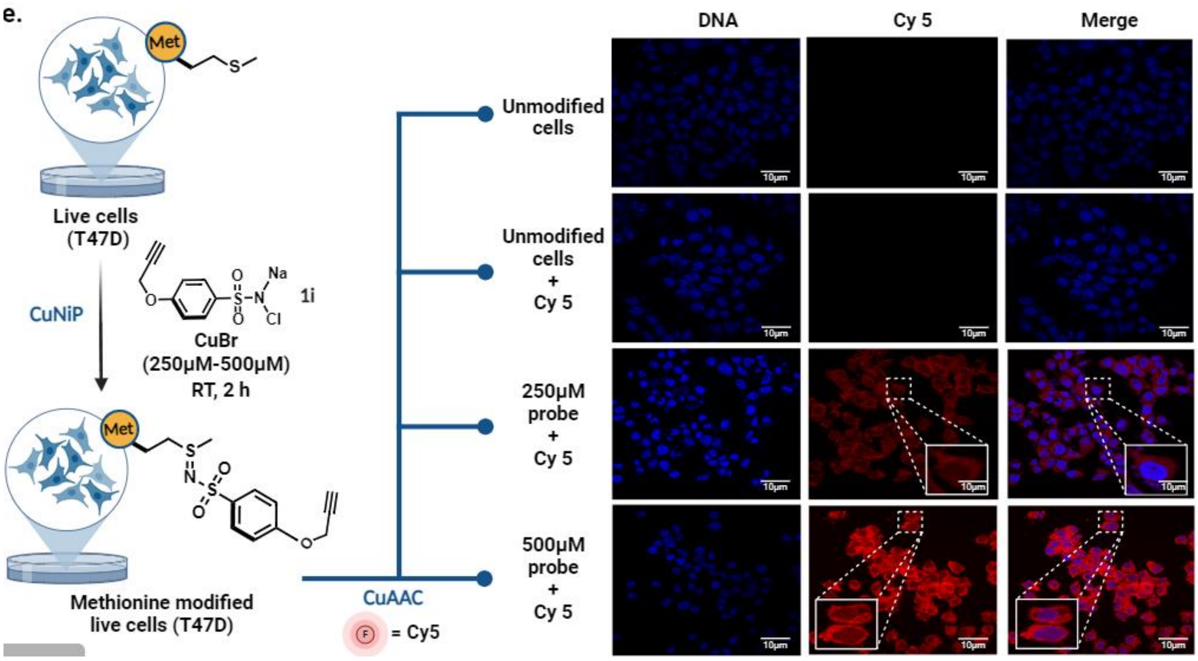

Supplementary Fig. 38 was created with BioRender.com, released under a Creative Commons Attribution-NonCommercial-NoDerivs 4.0 International license".

Supplementary Notes.

Cartesian coordinates and energy properties of computational studies.

Cartesian coordinates, Thermal correction to Gibbs Free Energies (TCG), and single-point electronic energies (E) of reactants, intermediates, and transition states.

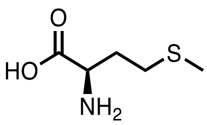

L-METHIONINE

N	2.56031500	-1.59695200	-0.28958500
H	2.67159400	-1.74499900	0.71003300
C	1.52892900	-0.58537000	-0.52630700
H	1.45001800	-0.41875200	-1.60227100
C	0.13389700	-0.97129600	0.02536200
C	1.98895100	0.70986700	0.12027100
H	-0.12919900	-1.93088900	-0.42886300
H	0.22041100	-1.13449400	1.10406400
C	-0.95809800	0.05365100	-0.26918800
O	2.50363500	0.78208300	1.21284400

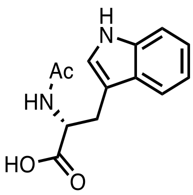

N-ACETYL-TRYPTOPHAN

N	-3.06903500	2.07564200	0.00900100
H	-3.69994200	1.72532200	0.72542200
H	-3.59953600	2.10984200	-0.85647100
C	-1.92618100	1.17563400	-0.12587300
H	-1.32824600	1.49815800	-0.97903700
C	-1.06208400	1.23844400	1.15557000
C	-2.33194100	-0.27806800	-0.35763500
H	-0.75768600	2.28133600	1.28144100

H	-0.76158500	0.99975000	0.23863000
H	-1.02593900	0.24727700	-1.34221100
S	-2.58074400	-0.57547600	0.31169800
C	-3.62963400	0.84993800	-0.13580700
H	-3.60295100	1.02383100	-1.21213500
H	-4.64854900	0.60385000	0.16262700
H	-3.30675200	1.74634500	0.39499600
H	2.26469500	-2.48199200	-0.69018400
O	1.72856500	1.78699000	-0.64130000
H	1.99609300	2.58302800	-0.15310000

TCG = 0.126479
E = -800.630022

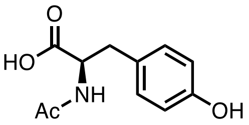

N-ACETYL-TYROSINE

N	-3.13940700	-1.69253000	-0.14348600
H	-3.51501300	-1.15926800	-0.92265100
H	-3.80193400	-1.60911200	0.62173000
C	-1.82943000	-1.16548400	0.24500000
H	-1.49930300	-1.69766000	1.13881100
C	-0.81256600	-1.40347700	-0.88941700
C	-1.85670000	0.31286600	0.63264400
H	-0.76263200	-2.48266100	-1.05275900
H	-1.20276600	-0.95048300	-1.80432000
C	0.55512200	-0.84480700	-0.58048800
O	-1.53881100	0.75311800	1.71689700
C	0.96809600	0.38547400	-1.09710700
C	1.42988700	-1.52409100	0.27574900
H	0.31055900	0.93384500	-1.76231400
C	2.21118500	0.92767300	-0.77493000
C	2.67342700	-0.99844500	0.60704400
H	1.13619600	-2.48313800	0.68915400
H	2.51350500	1.88372800	-1.18914300
C	3.06589700	0.23363300	0.08063000
H	3.34695600	-1.53240400	1.26660900
O	4.30169400	0.70836600	0.43583400
H	4.46322200	1.55781000	0.00819200
O	-2.28731000	1.07786700	-0.38078700
C	-2.35404200	2.50011400	-0.14320900
H	-3.03378600	2.71285800	0.68164800
H	-1.36125400	2.88727400	0.08630800
H	-2.72773500	2.93138400	-1.06791500

TCG = 0.178693
E = -669.402783

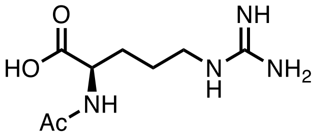

N-ACETYL-ARGININE

N	-3.09969200	-2.17733000	0.26366700
H	-3.20449300	-2.31384300	-0.73840400
H	-4.02853900	-1.99104300	0.63018900
C	-2.21362600	-1.04117400	0.51691100
H	-2.20316900	-0.84599800	1.59097700
C	-0.78843800	-1.38498500	0.05299500
C	-2.67903400	0.23267400	-0.18775500
H	-1.69950300	0.98224600	2.00703700
C	0.12783100	0.33208100	1.11932900
O	-3.18807900	-0.85488700	0.28008100
C	0.27719700	-0.85914400	1.78557100
C	1.31370600	0.50308800	0.31776400
H	-0.39047100	-1.34709200	2.47835700
N	1.48482600	-1.44011900	1.44788100
C	2.14518700	-0.62840100	0.55100700
C	1.75311600	1.49897800	-0.56936000
H	1.83346900	-2.30902500	1.81953700
C	3.38635000	-0.78045100	-0.07236600
C	2.98595600	1.35062600	-1.19102100
H	1.14132400	2.37246800	-0.76548400
H	4.00809900	-1.64785400	0.11659000
C	3.79357600	0.22141800	-0.94503400
H	3.33647100	2.11260800	-1.87756600
H	4.75057600	0.13313900	-1.44605000
O	-1.61934900	-0.85238900	-1.33395200
C	-1.85403000	-2.25941200	-1.56487000
H	-2.89355600	-2.42796800	-1.84491700
H	-1.18849500	-2.53425400	-2.37856700
H	-1.61570300	-2.82813800	-0.66599100

TCG = 0.202921
E = -725.745656

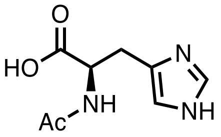

N-ACETYL-HISTIDINE

N	-2.16890800	-2.07288800	0.41151100
H	-1.97362500	-2.87488500	1.00279200
H	-2.23302100	-2.41512200	-0.54367400
C	-1.07921700	-1.09692500	0.52146500
H	-0.98276800	-0.82571200	1.57296200
C	0.27529900	-1.59697000	-0.01699900
C	-1.51599100	0.14359700	-0.24958600
H	0.53694400	-2.50920200	0.52918700
H	0.14577000	-1.87908900	-1.06583200
C	1.36539200	-0.57872100	0.09862700
O	-1.63096600	0.17819300	-1.45603200
N	2.39344400	-0.47837400	-0.81783200
C	1.62484500	0.41030700	1.01850900
H	2.51389700	-1.05438200	-1.63830800
C	3.21388300	0.53591600	-0.42565200
N	2.77749800	1.09830100	0.68368800
H	1.04934600	0.66921400	1.89284100
H	4.09571400	0.81511200	-0.98067500
O	-1.77829900	1.17708000	0.55502900
C	-2.20974400	2.40041400	-0.08464600
H	-1.43761400	2.75657200	-0.76626400
H	-3.13734600	2.23089300	-0.63069500
H	-2.36421500	3.10944300	0.72381100

TCG = 0.147236
E = -588.151730

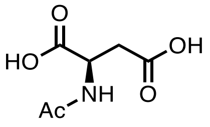

H	-0.50329100	-2.30894500	0.56344600
H	-0.81887800	-1.60714600	-1.01976000
C	0.24677400	-0.29418200	0.33066500
O	-3.06039900	0.27574800	-1.33845300
H	-0.02424900	0.62763100	-0.19639900
H	0.25800000	-0.05494200	1.39906200
C	1.64449500	-0.72330600	-0.10430300
H	1.95387200	-1.61752800	0.43978300
H	1.64414800	-0.98315300	-1.17122000
N	2.60738900	0.33540500	0.17390400
H	2.27080400	1.27478700	0.01431600
C	3.94973900	0.14612100	-0.05604900
N	4.41659900	-1.02173500	-0.35133600
N	4.69615800	1.32011500	0.03245300
H	5.43203100	-0.99097600	-0.40871000
H	4.34971900	2.01433500	0.68183300
H	5.69238200	1.18060200	0.12438000
O	-2.59896100	1.30729200	0.60795800
C	-2.95554500	2.57917000	0.01935000
H	-2.30179400	2.79682800	-0.82516100
H	-3.99364700	2.56185200	-0.31145300
H	-2.81738400	3.31212300	0.80937500

TCG = 0.203781
E = -645.905875

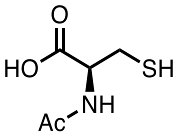

N-ACETYL-CYSTEINE

N	-0.36967300	2.41581800	0.38843100
H	-1.21285900	2.82190900	0.78209500
H	-0.32523000	2.69431800	-0.58809200
C	-0.42342300	0.95729100	0.49029700
H	-0.44990400	0.68381400	1.54600900
C	-1.64448100	0.35100600	-0.22524500
C	0.85561700	0.39911600	-0.12011300
H	-2.54844800	0.78311500	0.20238400
H	-1.59762400	0.58475100	-1.28917000
S	-1.70144700	-1.48650800	-0.01115500
O	1.28543600	0.73505500	-1.20166800
H	-2.82371700	-1.67853800	-0.73132600
O	1.42789500	-0.51663000	0.66500200
C	2.61003400	-1.16860500	0.14414200
H	2.36884400	-1.69085700	-0.78137400
H	3.39332200	-0.43327500	-0.03642800
H	2.91335100	-1.87208300	0.91420200

TCG = 0.098493
E = -761.313110

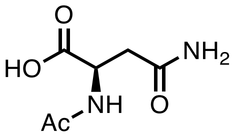

N-ACETYL-ASPARAGINE

N	0.89628000	2.49655600	0.24451000
H	0.25611200	3.25231200	0.46658200
H	1.17977700	2.61269700	-0.72439200
C	0.23043100	1.21136500	0.41941400

N-ACETYL-ASPARTIC ACID

N	0.97252600	2.45403800	0.05341400
H	1.77922600	2.57203800	0.65781900
H	1.31683000	2.50837300	-0.90134800
C	0.34916600	1.16675300	0.29890600
H	0.06492000	1.12339600	1.35499300
C	-0.93318800	1.04153300	-0.55257000
C	1.27622400	-0.03291000	0.05448800
H	-1.46066100	1.99848900	-0.49600700
H	-0.70364700	0.85000200	-1.60326700
C	-1.88621300	0.00141500	-0.06804900
O	2.44899600	0.04765300	-0.22666300
O	-2.21958700	-0.16090300	1.13522500
O	-2.51570500	-0.79169400	-0.81939100
O	0.61352700	-1.18519300	0.20184900
C	1.36069400	-2.40839000	0.00252600
H	1.75141400	-2.44371500	-1.01385000
H	2.17867500	-2.46528200	0.71978800
H	0.64761400	-3.21087300	0.16800800

TCG = 0.097957
E = -551.058461

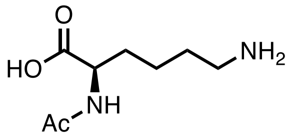

N-ACETYL-LYSINE

N	-2.21557600	-2.28271200	0.18600500
H	-2.29483100	-2.40081100	-0.82077000
H	-3.16037400	-2.16273400	0.53960200
C	-1.40465800	-1.10026500	0.48117700
H	-1.41238900	-0.93926700	1.56096300
C	0.04229200	-1.33377100	0.01691000
C	-1.94945900	0.16205800	-0.18479900
H	0.39192800	-2.24478200	0.51079400
H	0.03034200	-1.53818700	-1.06015200
C	1.00078100	-0.17898100	0.31310300
O	-2.31041300	0.22313100	-1.34149900
H	0.66957300	0.72685100	-0.20706000
H	0.97131100	0.05262100	1.38436100
C	2.43924000	-0.49612500	-0.10016200
H	2.78761700	-1.39324500	0.42336800
H	2.46577000	-0.72811800	-1.17251500
C	3.40387200	0.65113000	0.18600300
H	3.03355700	1.56254500	-0.30726100
H	3.41678200	0.85572600	1.26130700
N	4.77468200	0.30107000	-0.22229700
H	4.81340500	0.18939800	-1.23208900
H	5.40976900	1.06157800	0.00114100
O	-1.96862000	1.20716000	0.65351800
C	-2.40962700	2.47063000	0.10637900
H	-1.75502700	2.77378600	-0.71069400
H	-3.43498000	2.38697100	-0.25296400
H	-2.34857000	3.17878400	0.92819000

TCG = 0.196190
E = -536.358397

H	0.00359700	1.06869800	1.47637600
C	-1.09564500	1.07098200	-0.38375000
C	1.18130800	0.10943900	-0.02188500
H	-1.73696600	1.90882500	-0.10301000
H	-0.88069200	1.15015500	-1.45179600
C	-1.84973500	-0.20548600	-0.04800500
O	1.95338000	0.20792200	-0.95009800
O	-2.34622800	-0.38131800	1.06377300
N	-1.92320700	-1.12741900	-1.03308500
H	-2.39407800	-2.00328400	-0.85971500
H	-1.51893300	-0.97396100	-1.94263200
O	1.01749500	-1.00129800	0.70241600
C	1.77377500	-2.16276600	0.28885700
H	1.50557900	-2.43691300	-0.73128500
H	2.84167400	-1.95610500	0.35016100
H	1.49443100	-2.95003700	0.98322600

TCG = 0.124533
E = -531.843197

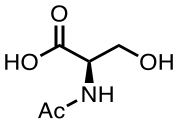

N-ACETYL-SERINE

N	0.85903600	-1.97734000	-0.04328100
H	1.79029500	-2.37938800	0.00206000
H	0.54667600	-2.04419700	-1.00781800
C	0.89855100	-0.57504800	0.38357300
H	1.32805000	-0.53205000	1.38533200
C	1.71585200	0.32637800	-0.54727100
C	-0.52873700	-0.05853900	0.51445800
H	2.74855800	-0.03937800	-0.56752500
H	1.30338000	0.27178300	-1.56046600
O	1.65337300	1.66219300	-0.04208700
O	-1.02700400	0.36056600	1.53520400
H	2.11077100	2.24655000	-0.65575100
O	-1.17563700	-0.13354100	-0.65557500
C	-2.55210400	0.30468700	-0.67152000
H	-3.14554800	-0.29930500	0.01461200
H	-2.61376900	1.35524700	-0.38847100
H	-2.88889000	0.16349800	-1.69478200

TCG = 0.105324
E = -438.330725

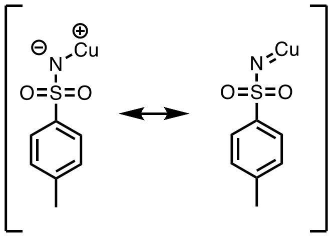

Cu-NITRENE

C	-1.61205000	-1.19723800	0.22709800
C	-2.95955300	-1.21701100	-0.11783900
C	-3.66045400	-0.03130100	-0.36940500
C	-2.97259100	1.18358300	-0.26641100
C	-1.62498700	1.22129400	0.07753400
C	-0.95432400	0.02645700	0.32587400
C	-5.12732800	-0.05875600	-0.70938000
S	0.79926900	0.05762300	0.69831000

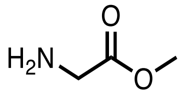

N-TERMINUS (GLYCINE METHYL ESTER)

N	2.16289700	-0.59093400	-0.15579600
C	0.73699000	-0.78167400	0.07504900
H	2.65985500	-1.44874500	0.05966700
H	2.52238700	0.13082400	0.46097800
C	-0.00008300	0.54009400	0.00312400
H	0.33630100	-1.46427400	-0.67836700
H	0.49095000	-1.22345700	1.05367600
O	0.56055900	1.61318100	-0.00494200
O	-1.34535000	0.51627400	-0.00979400
C	-2.07635000	-0.72980800	-0.01689100
H	-3.12406600	-0.44569500	0.03133500
H	-1.82185800	-1.33750200	0.85183900
H	-1.88886400	-1.28193200	-0.93836200

TCG = 0.076080
E = -323.778693

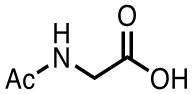

C-TERMINUS (N-ACETYL GLYCINE)

N	-0.53769800	-0.41682700	-0.00001900
H	-0.23588400	-1.38366500	-0.00009400
C	0.50582200	0.58498500	-0.00006100
C	1.85821900	-0.09110800	-0.00001800
H	0.45785600	1.23338900	-0.88063000
H	0.45780200	1.23348800	0.88043000
O	2.03193100	-1.28695500	-0.00012200
O	2.84673200	0.81213500	0.00015400
H	3.69822000	0.34599500	0.00011200
C	-1.87494400	-0.18739000	0.00004500
O	-2.67934600	-1.12143400	0.00012700
C	-2.31324700	1.25937200	-0.00007100
H	-3.39988400	1.30093900	-0.00002100
H	-1.93197100	1.78115400	-0.88166400
H	-1.93188900	1.78136500	0.88135900

TCG = 0.081441
E = -437.188428

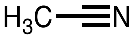

ACETONITRILE

C	0.27823900	-0.00017000	-0.00008600
N	1.43187500	0.00007000	0.00003500
C	-1.17535500	0.00003500	0.00001300
H	-1.54705200	-0.89039200	-0.50947100
H	-1.54668300	0.88665300	-0.51631700
H	-1.54669800	0.00406000	1.02597700

TCG = 0.021137
E = -132.788607

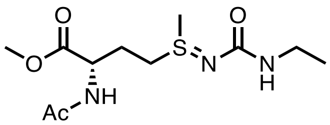

UREA ETHYL

O	1.08840300	1.39048700	1.27632400
O	1.08015600	-1.12596200	1.54474400
N	1.54566000	-0.10878700	-0.73028100
Cu	3.38616300	-0.05642100	-0.73112400
H	-1.08012400	-2.11901000	0.42373600
H	-3.47419000	-2.16841100	-0.19241200
H	-3.49738900	2.11305300	-0.45707800
H	-1.10167700	2.16489500	0.15958200
H	-5.39704700	0.76989700	-1.36694800
H	-5.40423600	-0.99503400	-1.19717400
H	-5.73272600	0.03199000	0.19853200

TCG = 0.087295
E = -2514.873532

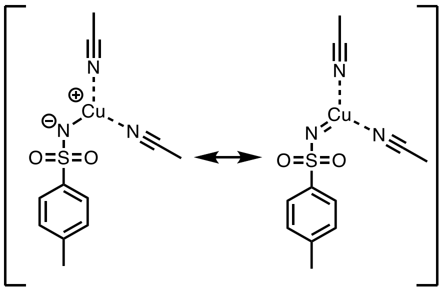

Cu-NITRENE-MeCN

C	-2.91936200	-0.90232700	-0.74856500
C	-4.23330200	-0.83651600	-1.19315000
C	-5.15187200	0.05005800	-0.61161100
C	-4.71341400	0.86614200	0.43394600
C	-3.39714800	0.80801000	0.89258200
C	-2.50543900	-0.07682500	0.29764800
C	-6.56817600	0.12681200	-1.11970800
S	-0.81098400	-0.19962800	0.89742900
O	-0.63633300	0.94935500	1.82725800
O	-0.69405000	-1.54637800	1.53060900
N	0.12459300	-0.12254700	-0.38285700
Cu	1.95473000	0.01995100	-0.29938200
H	-2.21728500	-1.58315300	-1.21374800
H	-4.55284000	-1.47949500	-2.00616500
H	-5.40701400	1.55786800	0.89916500
H	-3.06330100	1.44375300	1.70175300
H	-7.20513800	0.68909900	-0.43515800
H	-6.60371700	0.62282800	-2.09489800
H	-6.99544500	-0.87077200	-1.24826200
N	3.31747200	-1.42316500	-0.25779900
N	3.08748300	1.64952900	-0.37270100
C	4.52301600	3.81347200	-0.42842700
H	3.87590000	4.67993600	-0.57362500
H	5.24058400	3.75628200	-1.24857200
H	5.06180200	3.91924000	0.51463800
C	5.03781400	-3.36565600	-0.14390000
H	4.52768600	-4.31711100	-0.30227100
H	5.51636200	-3.37146000	0.83681900
H	5.79811100	-3.22975100	-0.91480600
C	3.72305600	2.60723400	-0.39726000
C	4.07967600	-2.28245700	-0.20840400

TCG = 0.162752
E = -2780.485848

C	-2.32366600	1.48491500	0.56597400
O	-2.02428800	1.81710100	1.68988200
N	-2.81822700	-0.84709200	0.12110300
C	-1.83940600	0.20734500	-0.12746300
C	-0.44709500	-0.19280800	0.36736700
C	0.06527000	-1.39418500	-0.41207600
S	1.73740600	-2.05078400	0.00179300
C	1.55675400	-2.37033700	1.79489400
O	-3.16516300	2.16047300	-0.22245800
C	-3.77872700	3.34292500	0.34326100
N	2.89592700	-0.94326000	-0.32230500
C	3.01247100	0.18383900	0.45514000
O	-4.62512100	-2.11646300	-0.31105000
C	-3.81587500	-1.27602400	-0.70362600
C	-3.86937200	-0.69696000	-2.09826500
O	2.35291400	0.47099700	1.47346800
C	4.95544800	0.75985200	-1.04264200
H	-2.85017100	-1.22970600	1.05867600
H	-1.80906300	0.40028200	-1.19783200
H	-0.49497100	-0.40616500	1.43589400
H	0.22741800	0.65298400	0.24748100
H	0.14718200	-1.17618000	-1.47878100
H	-0.58604200	-2.26437200	-0.29856900
H	2.50465400	-2.79684500	2.11926200
H	1.34439700	-1.45110800	2.32747000
H	0.76194600	-3.10979200	1.89476400
H	-4.41506800	3.74023100	-0.44228300
H	-4.36943700	3.07289200	1.21821300
H	-3.01137600	4.06502300	0.62003700
H	-4.70088800	-1.15384300	-2.62965300
H	-4.00936900	0.38538700	-2.06307300
H	-2.94292600	-0.89879600	-2.64161000
H	4.21085700	1.77630400	0.66413300
H	5.63760600	-0.03974600	-0.72770600
H	4.42178800	0.39438400	-1.92108600
N	3.98015700	1.04730300	0.00470500
C	5.74606400	2.01703100	-1.38509400
H	6.48082200	1.79956600	-2.16320700
H	5.08531600	2.80881100	-1.74769700
H	6.28590300	2.39418100	-0.51120300

TCG = 0.272021
E = -1295.273027

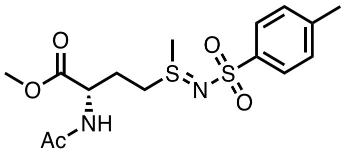

N-ACETYL METHIONINE CH_3-SULFONYL-SULFIMIDE

N	2.49646700	1.15740400	0.38509000
H	2.96381100	1.49233200	-0.44848300
C	2.26188600	-0.27133400	0.46357500
H	2.31826900	-0.59989300	1.50157100
C	0.87482100	-0.64404500	-0.11491900
C	3.37378100	-0.97369400	-0.30822400
H	0.15369800	0.01912900	0.36150100
H	0.86020000	-0.42215500	-1.18404700
C	0.47223400	-2.08935400	0.14686900

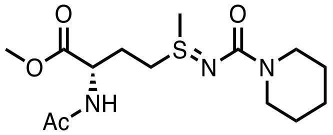

UREA CYCLOPENTYL

C	2.49740200	1.73392600	-0.34864100
O	2.03242300	2.06968500	-1.41391600
N	3.50054600	-0.47418600	-0.32796000
C	2.41063400	0.31948400	0.23174100
C	1.05249900	-0.31662300	-0.07866000
C	0.92029800	-1.65386900	0.63199000
S	-0.62641100	-2.61425100	0.32800800
C	-0.35523600	-3.12856300	-1.41095600
O	3.19808900	2.53777300	0.45700800
C	3.42947100	3.88748600	-0.01261900
N	-1.93380000	-1.66233400	0.52716300
C	-2.25006300	-0.71208800	-0.42616000
O	5.55821700	-1.38296300	-0.39275900
C	4.70546400	-0.76145200	0.24130200
C	4.93338800	-0.30619600	1.66432100
O	-1.64189500	-0.51654200	-1.49761200
C	-3.76275500	1.13057500	-0.98065300
H	3.40741900	-0.76018200	-1.29543300
H	2.55289800	0.38800400	1.30787700
H	0.93826300	-0.42293000	-1.15652300
H	0.26515000	0.36060300	0.25374500
H	0.93101700	-1.52644600	1.71595300
H	1.72089100	-2.34787900	0.36585800
H	-1.27379600	-3.62013300	-1.72736700
H	-0.14175400	-2.27603400	-2.04416000
H	0.46247200	-3.84913100	-1.38425100
H	4.00934500	4.36846800	0.76986100
H	3.98746500	3.86520600	-0.94819100
H	2.47882600	4.39944600	-0.15792500
H	5.93699300	-0.59900800	1.96369000
H	4.82867900	0.77729000	1.74982900
H	4.20966700	-0.76815900	2.34076200
H	-2.87879700	1.48047700	-1.51053500
H	-4.47447700	0.77579700	-1.73892800
N	-3.35560300	0.02900400	-0.10156500
C	-4.38888100	2.25323400	-0.15579700
H	-4.61879700	3.09654600	-0.81160300
H	-3.64178900	2.60632900	0.56207500
C	-5.65364600	1.77876500	0.58941900
H	-6.54289000	1.97407500	-0.01652800
H	-5.76974700	2.35566600	1.51124100
C	-5.57686100	0.27306900	0.91181200
H	-6.00809300	-0.30793000	0.09044200
H	-6.16249900	0.03787900	1.80392300
C	-4.13745300	-0.19383600	1.12197400
H	-3.68577500	0.34045900	1.96902100
H	-4.10635800	-1.25487300	1.35938800

TCG = 0.332450
E = -1411.978497

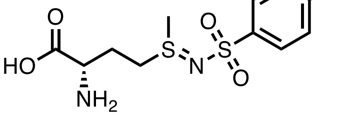

O	4.04184500	-0.44577100	-1.16770100
H	1.04629000	-2.80543900	-0.44034100
H	0.53467100	-2.34938800	1.20507400
S	-1.27525900	-2.32883400	-0.33976700
C	-1.50300400	-4.05845700	0.14612500
H	-1.26453200	-4.15761500	1.20387300
H	-2.54800700	-4.29462900	-0.04317500
H	-0.85335600	-4.67497800	-0.47442300
O	3.47979600	-2.24896900	0.06114700
C	4.44846800	-3.05720300	-0.65305800
H	4.19579800	-3.08858500	-1.71217700
H	5.44694600	-2.64388600	-0.51772000
H	4.37706000	-4.04597500	-0.20978300
C	2.17884700	2.09371100	1.31635700
C	1.50561400	1.61491700	2.58315800
O	2.44883300	3.28106500	1.12991500
H	2.20141600	1.01742800	3.17879300
H	1.20207700	2.48315700	3.16327700
H	0.62908000	0.99921100	2.37526100
C	-0.50788200	2.98508100	-1.15442700
C	-1.01825400	2.04171100	-2.05053800
C	-1.88778600	1.03964100	-1.62585500
C	-2.24945100	0.98131000	-0.28369800
C	-1.76999900	1.92038100	0.62973300
C	-0.90790800	2.91500100	0.18794000
H	-0.72757900	2.08380500	-3.09402200
H	-2.27271300	0.30907900	-2.32514100
H	-2.06125100	1.86201200	1.67052800
H	-0.52649300	3.64165400	0.89563400
C	0.44748200	4.05638900	-1.60535600
H	0.74620200	3.91026200	-2.64433000
H	-0.01224400	5.04584000	-1.52015900
H	1.34110500	4.05622800	-0.97602400
S	-3.19065800	-0.42146800	0.31427700
O	-3.92698000	0.00680600	1.51563900
O	-3.97818200	-0.92911500	-0.83097600
N	-2.08666200	-1.51014800	0.85946200

TCG = 0.304272
E = -1866.955810

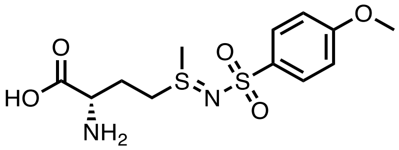

METHIONINE OCH_3-SULFONYL SULFIMIDE PRODUCT

C	-2.55591100	-0.41375000	-0.76635100
C	-3.89826100	-0.35517600	-1.13848100
C	-4.84844800	0.10911700	-0.22326700
C	-4.44546300	0.51297900	1.05823600
C	-3.11008000	0.45190900	1.42054800
C	-2.16167400	-0.01221600	0.50424000
S	-0.44359800	-0.16383600	1.01077300
O	-0.20641800	0.97993100	1.93553100
O	-0.31270800	-1.50867800	1.64773500
N	0.42639400	-0.09923600	-0.31308600
Cu	2.26060500	0.01241500	-0.32030400
H	-1.81188600	-0.75913400	-1.47201600

METHIONINE CH_3-SULFONYL SULFIMIDE PRODUCT

C	-2.91936200	-0.90232700	-0.74856500
C	-4.23330200	-0.83651600	-1.19315000
C	-5.15187200	0.05005800	-0.61161100
C	-4.71341400	0.86614200	0.43394600
C	-3.39714800	0.80801000	0.89258200
C	-2.50543900	-0.07682500	0.29764800
C	-6.56817600	0.12681200	-1.11970800
S	-0.81098400	-0.19962800	0.89742900
O	-0.63633300	0.94935500	1.82725800
O	-0.69405000	-1.54637800	1.53060900
N	0.12459300	-0.12254700	-0.38285700
Cu	1.95473000	0.01995100	-0.29938200
H	-2.21728500	-1.58315300	-1.21374800
H	-4.55284000	-1.47949500	-2.00616500
H	-5.40701400	1.55786800	0.89916500
H	-3.06330100	1.44375300	1.70175300
H	-7.20513800	0.68909900	-0.43515800
H	-6.60371700	0.62282800	-2.09489800
H	-6.99544500	-0.87077200	-1.24826200
N	3.31747200	-1.42316500	-0.25779900
N	3.08748300	1.64952900	-0.37270100
C	4.52301600	3.81347200	-0.42842700
H	3.87590000	4.67993600	-0.57362500
H	5.24058400	3.75628200	-1.24857200
H	5.06180200	3.91924000	0.51463800
C	5.03781400	-3.36565600	-0.14390000
H	4.52768600	-4.31711100	-0.30227100
H	5.51636200	-3.37146000	0.83681900
H	5.79811100	-3.22975100	-0.91480600
C	3.72305600	2.60723400	-0.39726000
C	4.07967600	-2.28245700	-0.20840400

TCG = 0.162752
E = -2780.485848

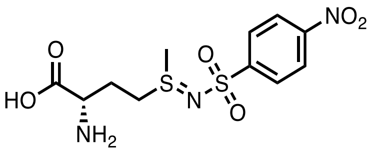

METHIONINE NO_2-SULFONYL SULFIMIDE PRODUCT

C	-2.58280900	-1.21201900	0.38619000
C	-3.88847500	-1.21934500	-0.08531400
C	-4.51908900	-0.00054800	-0.32181300
C	-3.88923800	1.22057400	-0.09533400
C	-2.58360100	1.21794700	0.37623400
C	-1.93947000	0.00410400	0.61242700
S	-0.20870000	0.00693900	1.14055800
O	-0.03744000	1.26977500	1.90410500
O	-0.03581600	-1.25097400	1.91178500
N	0.63195100	0.00403500	-0.20018400
Cu	2.46333200	0.00013400	-0.29461500
H	-2.06901100	-2.14221100	0.58695500
H	-4.41254400	-2.14655200	-0.26567900
H	-4.41390100	2.14592700	-0.28333500
H	-2.07042000	2.15009800	0.56937600
N	3.70238200	-1.54211900	-0.43003600
N	3.71147100	1.53460400	-0.43206800
H	-4.18428800	-0.66502200	-2.13341300
H	-5.19407600	0.87488000	1.75223800
H	-2.80001200	0.77091700	2.40717400
N	3.59755500	-1.45583500	-0.34337000
N	3.41902300	1.62027300	-0.43951900
C	4.89224900	3.75721300	-0.53771200
H	4.25863600	4.63355500	-0.68337200
H	5.59768600	3.67886200	-1.36655800
H	5.44533200	3.86304100	0.39704600
C	5.28085100	-3.43382000	-0.32902500
H	4.74854300	-4.36926700	-0.50838200
H	5.77899500	-3.48225900	0.64069600
H	6.02799600	-3.28679900	-1.11061500
C	4.07146700	2.56589000	-0.48311900
C	4.34362300	-2.33055700	-0.33749200
O	-6.17795500	0.20917100	-0.48493900
C	-6.64964800	-0.19106600	-1.77458200
H	-6.43794300	-1.24778200	-1.95842000
H	-7.72522200	-0.03142000	-1.75800300
H	-6.20010200	0.41881800	-2.56268800

TCG = 0.167628
E = -2855.713446

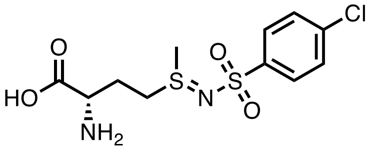

METHIONINE Cl-SULFONYL SULFIMIDE PRODUCT

C	-2.84094400	-1.21034400	0.28105800
C	-4.13889800	-1.21554900	-0.22439300
C	-4.77040700	-0.00090000	-0.47515000
C	-4.13922800	1.21451900	-0.22730200
C	-2.84126900	1.21088400	0.27814600
C	-2.19741100	0.00065900	0.53040200
S	-0.48059600	0.00159800	1.11059500
O	-0.32918400	1.26422400	1.88327400
O	-0.32956700	-1.25793200	1.88837900
N	0.43167500	-0.00098800	-0.18799300
Cu	2.26992600	-0.00095100	-0.26190200
H	-2.33914500	-2.14663300	0.48918600
H	-4.64963300	-2.15028200	-0.41638500
H	-4.65022900	2.14864400	-0.42153700
H	-2.33968300	2.14778300	0.48401900
N	3.51439200	-1.55202800	-0.39853700
N	3.51324900	1.55115000	-0.39955200
C	5.08842900	3.61253700	-0.55134400
H	4.49560100	4.52159100	-0.66593500
H	5.75317700	3.50426300	-1.41004100
H	5.68597400	3.68435300	0.35907400
C	5.09115800	-3.61220000	-0.55030700
H	4.49904000	-4.52172700	-0.66480400
H	5.68883700	-3.68349700	0.36006400
H	5.75574800	-3.50344400	-1.40906700
C	4.21020100	2.46351000	-0.46692300
C	4.21203800	-2.46385900	-0.46588200
Cl	-6.41325000	-0.00187500	-1.11290600

TCG = 0.124889
E = -3200.757376

C	5.32227100	3.56574800	-0.58669400
H	4.75798400	4.49268400	-0.47304200
H	5.82104300	3.56268800	-1.55727400
H	6.07166300	3.49935600	0.20378300
C	5.30169200	-3.58264700	-0.58061100
H	4.73245300	-4.50610600	-0.46335400
H	6.05250900	-3.51791900	0.20863800
H	5.79919800	-3.58533100	-1.55184300
C	4.42405000	2.43403200	-0.50003400
C	4.40986400	-2.44568700	-0.49632100
N	-5.90062200	-0.00306200	-0.82014900
O	-6.44554400	1.07836200	-1.02298400
O	-6.44543000	-1.08650300	-1.01223700

TCG = 0.135940
E = -2945.751932

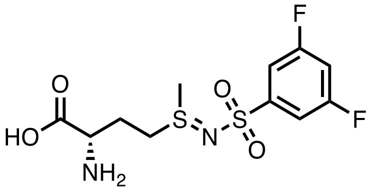

METHIONINE CF_2-SULFONYL SULFIMIDE PRODUCT

C	-3.01559500	-1.21189600	0.11458000
C	-4.27730500	-1.16622600	-0.45619700
C	-4.92136600	0.01674200	-0.78421700
C	-4.23624800	1.19137300	-0.51216600
C	-2.97393000	1.22035300	0.05707500
C	-2.37523300	-0.00009500	0.36393400
S	-0.68565800	-0.01173900	1.03567600
O	-0.58113400	1.23917000	1.83185700
O	-0.58576300	-1.28223400	1.80043600
N	0.28133300	0.00309400	-0.21764700
Cu	2.11948100	-0.00080200	-0.22379700
H	-2.55566600	-2.15807700	0.36456700
H	-2.48267200	2.16183700	0.26144900
N	3.36440200	-1.55193900	-0.33395000
N	3.37021600	1.54808600	-0.29458100
C	4.95779300	3.60418200	-0.36332200
H	4.37049400	4.52080500	-0.43928600
H	5.62062200	3.52714900	-1.22687800
H	5.55704500	3.63442100	0.54830400
C	4.94787400	-3.60902900	-0.45070900
H	4.35897900	-4.52199200	-0.55376500
H	5.54297000	-3.66495800	0.46242900
H	5.61469100	-3.51046300	-1.30898200
C	4.07257400	2.45817400	-0.32511400
C	4.06493300	-2.46247000	-0.38579100
H	-5.90932200	0.02347500	-1.22426900
F	-4.92138600	-2.33409200	-0.70191900
F	-4.83961700	2.36765200	-0.81450300

TCG = 0.120361
E = -2939.674564

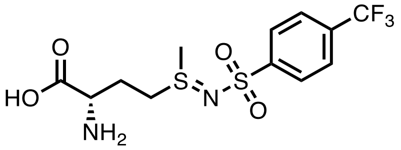

METHIONINE CF_3-SULFONYL SULFIMIDE PRODUCT

C	-2.25146900	-1.24444700	0.48005700
C	-3.56629000	-1.21754300	0.02714800
C	-4.20704200	0.00780900	-0.16950400
C	-3.53827700	1.20575900	0.08569700
C	-2.22312500	1.17878000	0.54019400
C	-1.58575900	-0.04547100	0.73119100
C	-5.61024900	0.02907500	-0.70533900
S	0.15472600	-0.07708900	1.24778500
O	0.33998800	1.15326900	2.06232700
O	0.32439200	-1.36613800	1.96975600
N	1.00667600	-0.03793100	-0.08775600
Cu	2.83650300	-0.00034100	-0.26188800
H	-1.75113800	-2.18937900	0.64722000
H	-4.08948000	-2.14653300	-0.16198800
H	-4.03896300	2.15440400	-0.05850200
H	-1.70167800	2.10267700	0.75398400
N	4.10898800	-1.51425800	-0.49878500
N	4.02735700	1.58757900	-0.44323800
C	5.52492000	3.69963600	-0.66492900
H	4.89747800	4.58345000	-0.79208100
H	6.18163600	3.59515100	-1.53025400
H	6.13140300	3.81460100	0.23507200
C	5.73989300	-3.51706300	-0.78432200
H	5.17200000	-4.44006700	-0.91258000
H	6.37190700	-3.60284700	0.10125100
H	6.36929000	-3.35548600	-1.66108100
C	4.69034800	2.52213800	-0.54134100
C	4.83017200	-2.40106200	-0.62541900
F	-6.36147900	-0.99316800	-0.23001900
F	-5.63924500	-0.08160700	-2.06287200
F	-6.26723000	1.17220800	-0.40484700

TCG = 0.138160
E = -3078.271167

Supplementary References:

1. Chan, W. C.; White, P. D. Fmoc solid phase peptide synthesis: A practical approach (Oxford Univ. Press, New York, 2000).
2. Nocentini, A. et al. Benzenesulfonamides Incorporating Flexible Triazole Moieties Are Highly Effective Carbonic Anhydrase Inhibitors: Synthesis and Kinetic, Crystallographic, Computational, and Intraocular Pressure Lowering Investigations. *J. Med. Chem.* **59**, 10692-10704 (2016).
3. Christian, A. H. *et al.* A Physical Organic Approach to Tuning Reagents for Selective and Stable Methionine Bioconjugation. *J. Am. Chem. Soc.* **141**, 12657-12662 (2019).
4. Kong, A. T., Leprevost, F. V., Avtonomov, D. M., Mellacheruvu, D. & Nesvizhskii, A. I. MSFragger: ultrafast and comprehensive peptide identification in mass spectrometry–based proteomics. *Nature Methods* **14**, 513-520 (2017).
5. Yu, F. *et al.* Fast Quantitative Analysis of timsTOF PASEF Data with MSFragger and IonQuant. *Mol. Cell. Proteomics.* **19**, 1575-1585 (2020).
6. Yu, F., Haynes, S. E. & Nesvizhskii, A. I. IonQuant Enables Accurate and Sensitive Label-Free Quantification With FDR-Controlled Match-Between-Runs. *Mol. Cell. Proteomics* **20**, (2021)
