# Supplementary material for: Copper(I)-nitrene platform for chemoproteomic profiling of methionine
Source: Nat Commun. 2024 May 18;15:4243. doi: 10.1038/s41467-024-48403-0 (PMC11102537; doi:10.1038/s41467-024-48403-0)
Supplement: Supplementary file 3 — Description of Additional Supplementary Files [file 41467_2024_48403_MOESM3_ESM.pdf]

<u>Description of Additional Supplementary Files</u>

Supplementary Data 1. Lysate chemoproteomics with Chloramine-T probe 1a

Supplementary Data 2. Lysate Chemoproteomics using Alkyne Probe 1i (dose-dependent labeling)

Supplementary Data 3. Oxidation sensitive methionine residue chemoproteomics

Supplementary Data 4. Live Cell chemoproteomics
